# Manganese‐Catalyzed Electrochemical Diazidation of Dehydroalanine Peptides

**DOI:** 10.1002/advs.202502711

**Published:** 2025-05-11

**Authors:** Xinwei Hu, Chengwei Zheng, Xiaotong Song, Balati Hasimujiang, Mu Chen, Zhixiong Ruan

**Affiliations:** ^1^ Guangzhou Municipal and Guangdong Provincial Key Laboratory of Molecular Target and Clinical Pharmacology and the State Key Laboratory of Respiratory Disease School of Pharmaceutical Sciences Guangzhou Medical University Guangzhou 511436 China

**Keywords:** dha‐containing peptides, diazidation, electrochemical, manganese, regioselectivity

## Abstract

Dehydroalanine (Dha), a readily introducible non‐canonical amino acid, is widely used for protein post‐translational modification (PTM) and site‐specific labeling of peptides and proteins. Despite its growing applications in biological systems, the precise modification of unnatural amino acid peptides and their conversion into functionalized endogenous peptides remain underdeveloped. To address this challenge, a novel peptide modification strategy is developed based on a manganese‐catalyzed electrochemical radical relay process between dehydroalanine peptides and sodium azide. This method demonstrates excellent functional group tolerance, accommodating amino acids with sensitive groups and even drug molecules, achieving yields of up to 99%. Mechanistic studies reveal that the reaction proceeds via a Mn(II)‐mediated electrochemical generation of azidyl radicals (N_3_⚫), which undergo regioselective addition to the Dha moiety, enabling efficient and site‐selective diazidation. The rapid diazidation strategy presented here provides a robust and efficient approach for converting unnatural amino acids into functionalized endogenous peptides, offering significant potential for applications in peptide chemistry and bioconjugation.

## Graphical abstract

A Mn(II)‐catalyzed electrochemical method achieves site‐selective diazidation of dehydroalanine peptides with NaN₃. This radical relay process tolerates diverse functional groups, delivers high yields, and enables precise peptide modification, offering a powerful tool for bioconjugation and therapeutic development.

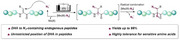

## 1 Introduction

Endogenous peptides hold significant potential for therapeutic applications in the treatment of various diseases.^[^ ^1^ ^]^ Existing peptide modification methods largely rely on traditional peptide condensations^[^ ^2^ ^]^ (amides) and nucleophilic residues, such as cysteine (─SH),^[^ ^3^ ^]^ lysine (─NH_2_),^[^ ^4^ ^]^ tryptophan (─NH),^[^ ^5^ ^]^ and tyrosine (phenol),^[^ ^6^ ^]^ which limits the diversity of peptides. Notably, advancing strategies that facilitate the direct modification of peptides into their endogenous forms is of paramount importance.

**Figure 1:**
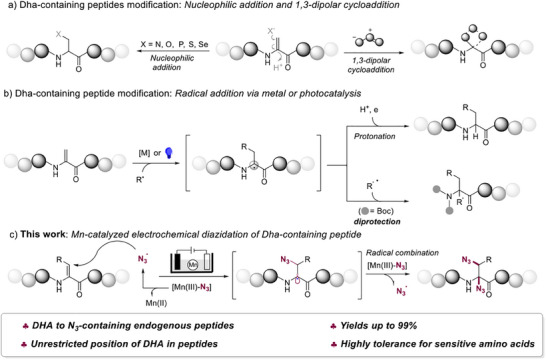
Late‐stage functionalization of Dha‐containing peptides.

Dehydroalanine (Dha), a non‐coding amino acid featuring a distinctive olefinic motif, contains an *α*, *β*‐unsaturated structure that enables precise late‐stage modification of peptides.^[^ ^7^ ^]^ Various Dha modification strategies have been developed, such as nucleophilic additions^[^ ^8^ ^]^ and 1,3‐dipolar cycloadditions,^[^ ^9^ ^]^ which readily undergo protonation to afford monosubstituted *α*‐amino acids (**Figure** **1**a). Additionally, photocatalytic and metal‐catalyzed dehydroalanine functionalization reactions have attracted considerable attention,^[^ ^7^, ^10^ ^]^ despite their difunctionalization reactions^[^ ^11^ ^]^ presenting certain limitations in terms of substrates (Figure 1b). However, the advancement of functionalization in the modification of Dha and their subsequent conversion into functionalized endogenous peptides remains highly attractive. Particularly noteworthy is the limited success achieved by only a few research groups in introducing azide groups into the distal modification of peptide compounds.^[^ ^12^ ^]^ While numerous methods for simple olefin azidation have been widely reported,^[^ ^13^ ^]^ including the elegant electrochemical diazidation of alkenes,^[^ ^14^ ^]^ electrochemical strategies for azide incorporation into dehydroalanine peptides remain remarkably underexplored. Building upon our previous investigations in electrochemical peptide modifications,^[^ ^15^ ^]^ we have developed an innovative electrochemical strategy for the synthesis of azidated dehydroalanine peptides via manganese‐stabilized N_3_ radicals (Figure 1c). Notable features of this strategy include: 1) excellent regioselectivity and high functional group tolerance, preserving sensitive groups and drug scaffolds with yields of up to 99%; 2) no positional restriction on dehydroalanine within the peptide sequence; and 3) the catalytic amount of Mn (II) facilitating the efficiency of the reaction.

## 2 Results and Discussion

This electrochemical strategy was successfully implemented, achieving an 87% yield of the desired product **2a** using methyl (2‐((*tert*‐butoxycarbonyl)amino)acryloyl)‐*L*‐leucinate (**1a**) as the model substrate and NaN_3_ as the nitrogen source (**Table** 1 and Table S1, S2). The reaction was carried out in the presence of MnF_2_, 1,10‐phenanthroline (1,10‐Phen), and LiClO_4_ at a constant current of 8.0 mA in a mixed solvent system of MeCN:DCE:AcOH (1.5:1.25:0.25, 3.0 mL) at room temperature for 5.0 h under an argon atmosphere (entry 1). The yields experienced a slight decline when alternative metallic catalysts were employed, such as MnCl_2_·4H_2_O, CuSO_4_·5H_2_O, CpFe_2_, and NiCl_2_·glyme (entries 2—5). Control experiments provided critical mechanistic insights into reaction optimization. The solvent composition emerged as a pivotal factor: the complete elimination of AcOH abolished product formation, while the omission of DCE significantly diminished the yield (entries 6–7), highlighting the synergistic effects of the ternary solvent system. Substituting the platinum (Pt) cathode with graphite felt (GF) resulted in a decreased yield of 79% (entry 8). A similar outcome was also obtained when increasing the electric current to 40 mA, suggesting that this electrochemical diazidation process holds considerable potential for industrial‐scale applications (entry 9). A notable contrast emerged when TMSN_3_ was employed as the azide source under identical conditions, yielding no diazidation product (entry 10), thereby confirming the unique reactivity profile of the NaN_3_/MnF_2_ system.

**Table 1:** Optimization of reaction conditions.

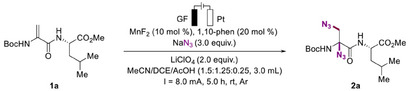		
Entry^a)^	Deviation from standard conditions	Yield [%]^b)^
1	None	87%
2	MnCl_2_·4H_2_O instead of MnF_2_	56%
3	CuSO_4_·5H_2_O instead of MnF_2_	45%
4	CpFe_2_ instead of MnF_2_	0%
5	NiCl_2_·glyme instead of MnF_2_	53%
6	No AcOH	N.D.
7	No DCE	69%
8	GF(+)|GF(−) instead of GF(+)|Pt(−)	79%
9	40 mA instead of 8.0 mA	80%
10	TMSN_3_ instead of NaN_3_	trace

^a)^ Reaction conditions: undivided cell, graphite felt anode and Pt cathode (1.5 cm × 1.0 cm × 0.01 cm), **1** (0.20 mmol), MnF_2_ (0.020 mmol), 1,10‐phen (0.040 mmol), NaN_3_ (0.60 mmol), LiClO_4_ (0.40 mmol) and MeCN:DCE:AcOH (1.5:1.25:0.25, 3.0 mL), constant current = 8.0 mA, 5.0 h (3.7 *F*), rt, under Ar;

^b)^ Yields of isolated products.

**Figure 2:**
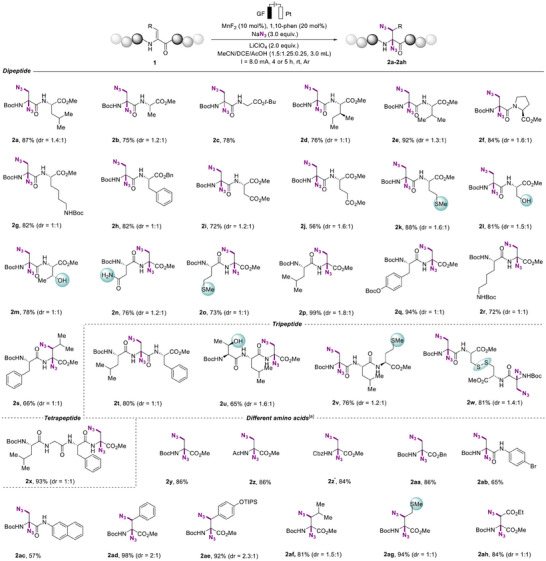
Scope of dehydroalanine derivatives. Isolated yields were reported. ^[a]^ 4 h (3.0 *F*).

With optimized reaction conditions in hand, we systematically explored the diversity of Dha side chains through electrochemical azidation. As shown in **Figure** **2**, the reaction exhibited high regioselectivity, yielding products with good yields regardless of whether the azide was introduced at the C‐ or N‐terminus of dehydroalanine. A broad array of dipeptide substrates incorporating diverse amino acid residues—including Ala, Gly, Leu, Val, Pro, Lys, Phe, Asp, Glu, Met, Ser, and Thr were subjected to electrooxidation, resulting in favorable yields. To our delight, the desired diazidation product could also be obtained from the reactive groups of methylthio and hydroxyl with moderate to good yields. We further extended the reaction to study the substrate scope at the N‐terminus of Dha featuring residues such as Asp, Met, Leu, Tyr, Lys, and Phe. All substrates reacted favorably with NaN_3_, affording the corresponding N_3_‐containing endogenous peptide products with excellent yields. Notably, the diazidation product derived from 1p was obtained with an almost equivalent yield, underscoring the reaction's robust transformation. Moreover, the versatility of this electrochemical oxidative azidation strategy extends beyond dipeptides. Tripeptides bearing sensitive functional groups (**2u**–**2w**) and a tetrapeptide (**2w**) were successfully modified, with yields ranging from 65% to 81%. Remarkably, a symmetrical double dehydroalanine containing a disulfide bond also showed excellent compatibility. Subsequently, we extended our investigation to the diazidation of dehydroalanine derivatives. Gratifyingly, dehydroalanine substrates containing Boc (**2y**), Ac (**2z**), Cbz (**2z** ^'^), Bn (**2aa**), aryl (**2ab**—**2ae**), alkyl (**2af** and **2ag**), and ester (**2ah**) groups at the terminal position underwent smooth reactions, delivering the corresponding diazide products in excellent yields.

**Figure 3:**
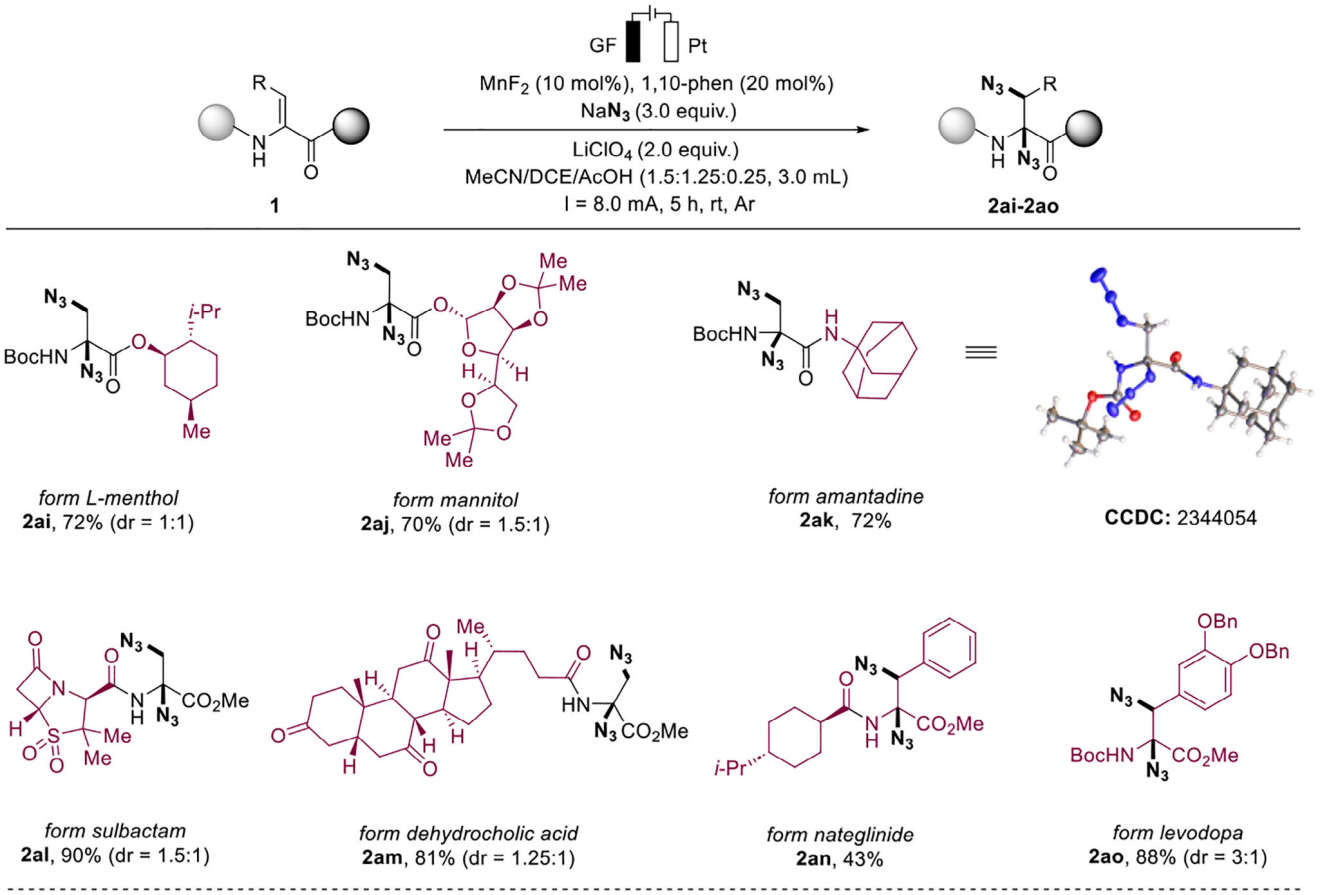
Late‐stage modifications of Dha‐containing biomolecules by electrolysis.

Considering the remarkable advantages of significant functional group compatibility, the electrochemical diazidation of dehydroalanine ultimately establishes a robust platform for the efficient late‐stage diversification of complex bioactive molecules (**Figure** **3**). Encouragingly, the methodology demonstrated exceptional versatility when applied to dehydroalanine derivatives of commercially available pharmaceuticals and biologically relevant compounds. Substrates derived from L‐menthol (**2ai**), mannitol (**2aj**), amantadine (**2ak**), sulbactam (**2al**), and dehydrocholic acid (**2am**) underwent smooth electrochemical diazidation, delivering products in moderate to excellent yields. Particularly noteworthy is the application of this strategy to the diazidation of the hypoglycemic drug nateglinide (**2an**) and the neuropharmaceutical levodopa (**2ao**), demonstrating its potential for modifying structurally complex drug molecules. Furthermore, the structure of **2ak** was unambiguously confirmed by single‐crystal X‐ray diffraction studies (For details see the Supporting Information). As a result, our development of a straightforward diazide amino acid synthesis method enables precise late‐stage azide installation on bioactive molecules, facilitating click chemistry applications, targeted drug delivery, and structure‐activity studies across medicinal chemistry, chemical biology, and materials science.

**Figure 4:**
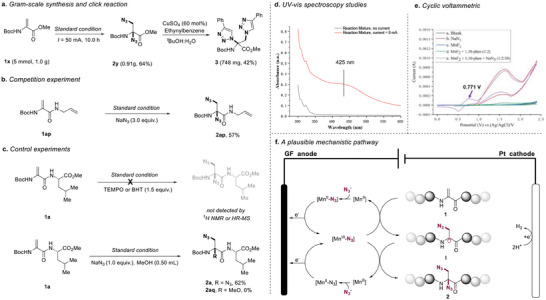
Synthetic application and mechanistic studies: a) Gram‐scale synthesis. b) Competition experiment. c) Control experiments. d) UV–vis spectroscopy studies. e) Cyclic voltametric. f) A plausible mechanistic pathway.

The practical utility of the electrochemical diazidation approach was further demonstrated by the gram‐scale synthesis of **2y** (64% yield), in which a higher constant current (50 mA) was employed to ensure the conversion in 10 h. Building on this, the coupling of **2y** with phenylacetylene was successfully achieved via a click reaction, demonstrating the potential of this methodology in bioactivity‐related applications (**Figure** **4**a). To elucidate the reaction mechanism, we conducted a series of systematic investigations. Competitive experiments between Dha and simple olefins revealed remarkable selectivity for Dha's double bonds (Figure 4b). Radical scavenging experiments using TEMPO or BHT completely inhibited product formation, suggesting radical involvement (Figure 4c). Furthermore, methanol addition experiments excluded the possibility of both ionic nucleophilic attack pathways and direct anodic oxidation of Dha.^[^ ^12b^ ^]^ Intriguingly, UV–vis spectroscopy revealed a characteristic peak at 425 nm, indicating valence changes in the Mn–N_3_ complex under electrochemical conditions (Figure 4d).^[^ ^14^, ^16^ ^]^ This observation was corroborated by cyclic voltammetry studies (Figure 4e and Figure S4,S5), which demonstrated distinct electrochemical behavior: while neither MnF_2_ nor MnF_2_/1,10‐phen showed redox activity between 0–2.5 V. Notably, the complete system (MnF_2_/1,10‐phen/NaN_3_) showed an oxidation peak at 0.771 V. Based on these findings, we propose a catalytic cycle involving redox‐active Mn(II) complexation with N_3_^–^, followed by anodic oxidation from Mn(II)–N_3_ to Mn(III)–N_3_.

Based on comprehensive mechanistic investigations, we propose a plausible reaction pathway (Figure 4f). The catalytic cycle initiates with the formation of a [Mn(II)‐N_3_] adduct through complexation between the redox‐active Mn(II) catalyst and azide anion at the anode. Subsequent anodic oxidation generates the key azido transfer agent [Mn(III)‐N_3_], which releases the reactive azide radical species. The dehydroalanine peptide substrate then undergoes regioselective azide radical addition, forming the crucial alkyl radical intermediate **I**. This intermediate subsequently couples with a second azide radical, generated through continuous [Mn(III)‐N_3_] decomposition, to afford the final diazidation product **2**.

## 3 Conclusion

In conclusion, we have successfully developed an innovative strategy for the site‐selective modification of endogenous peptides derived from dehydroalanine via a Mn(II)‐catalyzed electrochemical radical relay process between NaN_3_ and Dha. This strategy leverages the unique reactivity of Mn(II) to mediate the electrochemical generation of azidyl radicals (N₃•) and their subsequent regioselective addition to Dha, enabling efficient peptide modification under mild conditions. The method demonstrates broad functional group compatibility and excellent site selectivity, offering a powerful tool for the precise modification of peptide‐based pharmaceuticals and advancing green synthetic chemistry.

## 4 Experimental Section

### General Procedure for the Synthesis of **2**

In an undivided cell (10 mL) equipped with a stirring bar, a mixture of substrates **1** (0.20 mmol), NaN_3_ (0.6 mmol, 39 mg), MnF_2_ (10 mol%, 2.0 mg), 1,10‐phen (20 mol%, 7.0 mg), LiClO_4_ (0.40 mmol, 41 mg), and MeCN:DCE:AcOH (1.5:1.25:0.25, 3.0 mL) were added. The reaction was then initiated by applying a constant cell current of 8.0 mA cm^−2^ (Pt (‐), GF (+)) at room temperature for 4 or 5 h under nitrogen. Upon completion, the solvent was removed directly under reduced pressure to afford the crude product, which was further purified by flash column chromatography to afford the desired products **2**.

## Conflict of Interest

The authors declare no conflict of interest.

## Supporting information



Supporting Information

## Data Availability

The data that support the findings of this study are available in the supplementary material of this article.
