# Supplementary material for: Manganese‐Catalyzed Electrochemical Diazidation of Dehydroalanine Peptides
Source: Adv Sci (Weinh). 2025 May 11;12(28):2502711. doi: 10.1002/advs.202502711 (PMC12302629; doi:10.1002/advs.202502711)
Supplement: Supplementary file 1 — Supporting Information [file ADVS-12-2502711-s001.pdf]

ADVANCED SCIENCE
Open Access

Supporting Information

for *Adv. Sci.*, DOI 10.1002/advs.202502711

Manganese-Catalyzed Electrochemical Diazidation of Dehydroalanine Peptides

*Xinwei Hu, Chengwei Zheng, Xiaotong Song, Balati Hasimujiang, Mu Chen and Zhixiong Ruan**

Supporting Information

Manganese-Catalyzed Electrochemical Diazidation of Dehydroalanine Peptides

Xinwei Hu,† Chengwei Zheng,† Xiaotong Song, Balati Hasimujiang, Mu Chen, and Zhixiong Ruan*

Guangzhou Municipal and Guangdong Provincial Key Laboratory of Molecular Target & Clinical Pharmacology and the State Key Laboratory of Respiratory Disease, School of Pharmaceutical Sciences, Guangzhou Medical University, Guangzhou 511436, P. R. China.

Email: zruan@gzhmu.edu.cn

†These authors contributed equally to this work.

Table of Contents

1.General remarks 3

2.Experimental procedures 4

Preparation of substrates **1a-1m, 1t, 1w,** and **1ab**. 4
Preparation of substrates **1n-1r, 1t-1u, 1x, 1al-1am,** and **1aq** 5
Preparation of substrates **1s** and**1an** 5
Preparation of substrates **1ad-1ah**, **1ao**. 6
Preparation of substrates **1ai-1aj**. 6

3.Optimization studies 7

4. General procedure for electrochemical reactions 11

5. The gram-scale synthesis of **2y** 37

6. Intermolecular [3+2]-cycloadditions 38

7. Competition experiment 38

8. Control experiments 39

9. UV-vis spectroscopy studies 40

10. Cyclic Voltammetry Studies 40

11. Crystallographicdescriptionof **2ak**. 42

12. References 46

13. ^{1}H, ^{13}C, and ^{19}F Spectra 47

1.General remarks

Electrochemical reactions were performed in constant current mode using an AXIOMET AX-3003P potentiostat under argon, equipped with an undivided cell, a graphite felt (1.0 cm × 1.0 cm × 0.2 cm) as the anode and a platinum plate (1.0 cm × 1.0 cm × 0.01 cm) as the cathode (**Figure S1 and Figure S2**).Graphite felts are commercially available from Bei Jing Jinglong Special Carbon Technology Co., Ltd. Platinum electrodes are commercially availablefrom Tian Jin Aida (China).Chemicals were obtained from commercial sources and were used without further purification. Yields refer to isolated compounds, estimated to be >95% pure as determined by ^{1}H-NMR. TLC: Macherey-Nagel, TLC plates Alugram®Sil G/UV254. Detection under UV light at 254 nm. Chromatography separations were carried out on 300-400 mesh silica gel manufactured by Qingdao Haiyang Chemical Group Co. (China). High resolution mass spectrometry (HRMS)was measured on Thermo-DFS mass spectrometer. NMR spectra were recorded onBruker spectrometer at 400 MHz (^{1}H 400 MHz; ^{13}C 100 MHz). Chemical shifts were reported relative to *tetra*-methylsilane, dimethyl sulfoxide in $CDCl_3$. If not otherwise specified, chemical shifts (δ) are given in ppm.

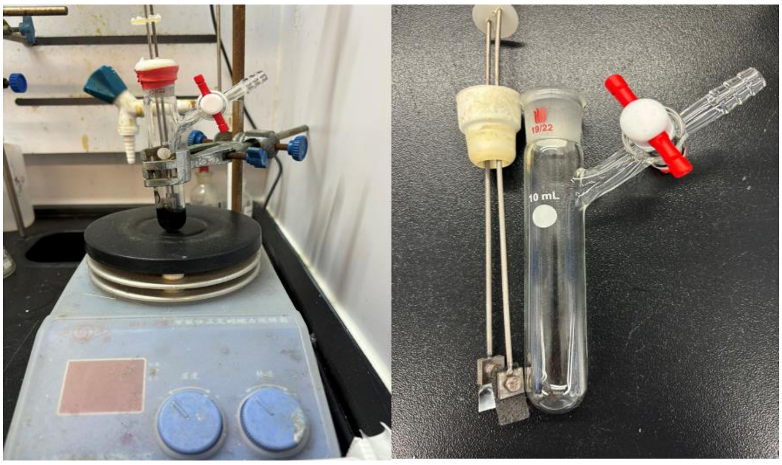

Figure S1. Equipment of standard reaction

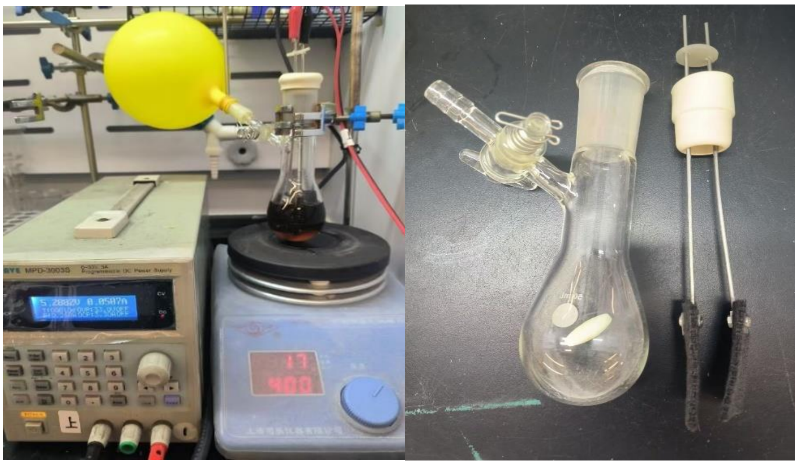

Figure S2. Equipment of gram-scale reaction

2.Experimental procedures

The substrates **1a-1ap** [1] were synthesized according to previously described methods as follows.

Preparation of substrates 1a-1m, **1t**, **1w**, and **1ab** [1].

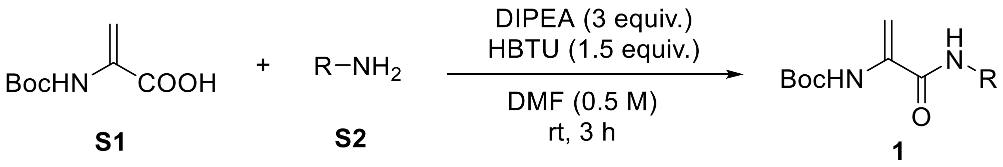

To a solution of **S1** (10 mmol, 1.87 g) and **S2** (11 mmol, 1.1 equiv.) in DMF (0.5 M) was added DIPEA (20 mmol, 2.68 g). The mixture was stirred for 10 min at room temperature, and HBTU (15 mmol, 5.69 g) was added to the solution. The rection was stirred 3 h. After regular workup, the reaction mixture washed by saturated $NaHCO_3$ solution (40 mL x 3), HCl (2M, 40 mL x 3) and H_2O (40 mL x 3). The organic layers were combined, dried over Na_2SO_4, and concentrated. The resulting crude product was purified by flash chromatography (DCM/MeOH or PE/EA) to afford **1a-1m**, **1v-1w**, **1ab**, **1ac**, and **1ak**.

Preparation of substrates 1n-1r, **1t-1u**, **1x**, **1al-1am**, and **1aq** [1,2].

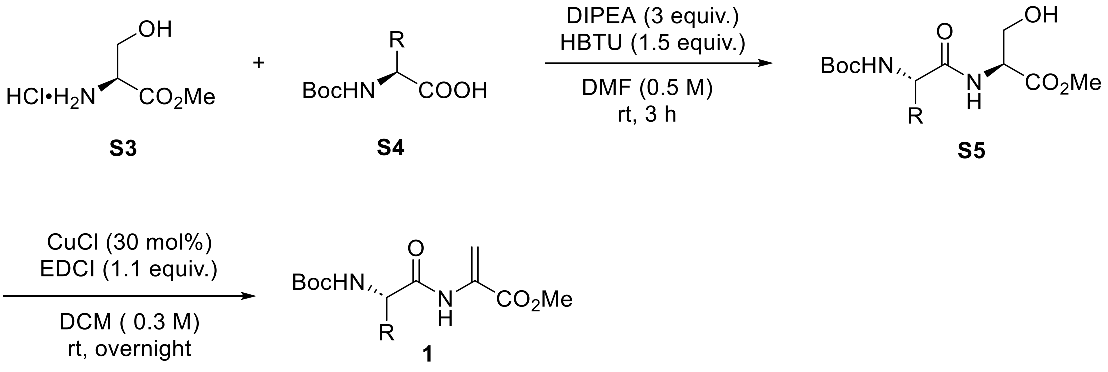

To a solution of **S3** (10 mmol, 1.55 g) and **S4 (**11 mmol, 1.1 equiv.**)** in DMF (0.5 M) was added DIPEA (20 mmol, 2.68 g). The mixture was stirred for 10 min at room temperature, and HBTU (15 mmol, 5.69 g) was added to the solution. The rection was stirred 3 h. After regular workup, the reaction mixture washed by saturated $NaHCO_3$ solution (40 mL x 3), HCl (2M, 40 mL x 3) and H_2O (40 mL x 3). The organic layers were combined, dried over Na_2SO_4, and concentrated to afford crude product **S5**. To a solution of **S5** (2.0 mmol, 1.0 equiv.) in 15 mL DCM (0.3M) was added $CuCl_2$ (30 mol%, 80 mg) and EDCI (11 mmol, 2.1 g) was stirred overnight at room temperature. After regular workup, the reaction mixture washed by saturated $NaHCO_3$ solution (40 mL x 3), HCl (2M, 40 mL x 3) and H_2O (40 mL x 3). The organic layers were combined, dried over Na_2SO_4, and concentrated. The resulting crude product was purified by flash chromatography (DCM/MeOH or PE/EA) to afford **1n-1r**, **1t-1u**, **1x**, **1al-1am**, and **1aq**.

Preparation of substrates 1s and**1an** [1, 3]

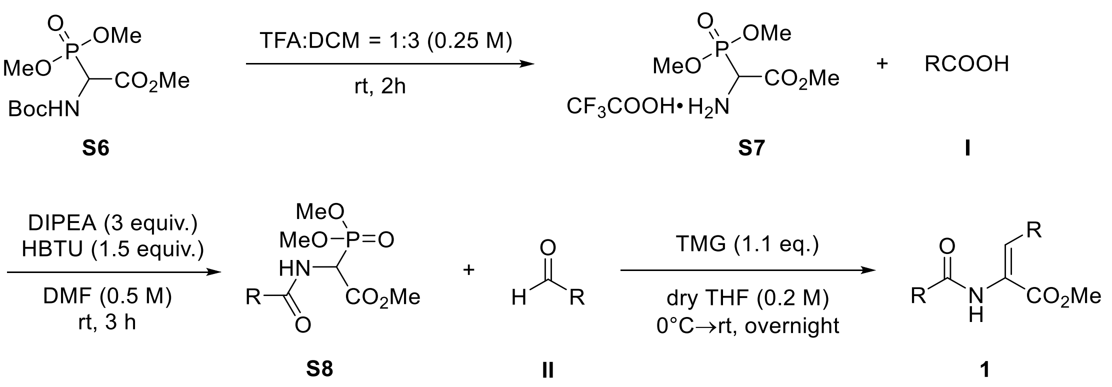

S6 (10 mmol, 2.92 g) in DCM (30 mL) was treated with TFA (10 mL) for 2 h and then concentrated in vacuo to afford **S7**. Then, a solution of **S7** (10 mmol, 1.0 equiv.,

3.11 g) and **I** in DMF (0.5 M) was added DIPEA (20 mmol, 2.68 g). The mixture was stirred for 10 min at room temperature, and HBTU (15 mmol, 5.69 g) was added to the solution. The rection was stirred 3 h. After regular workup, the reaction mixture washed by saturated $NaHCO_3$ solution (40 mL x 3), HCl (2M, 40 mL x 3) and H_2O (40 mL x 3). The organic layers were combined, dried over Na_2SO_4, and concentrated to afford crude product **S8**. Finally, a solution of **S8** (10 mmol, 1.0 equiv.) and phosphonate (11 mmol, 1.1 equiv.) in anhydrous THF (60 mL) was added *tetra*-methylguanidine (11 mmol, 1.27 g) slowly at 0 °C. The mixture was stirred at rt overnight, quenched with cold water (30 mL) at 0 °C and extracted with EA (20 x 3 mL). The combined organic extracts were washed with brine (20 mL), dried over Na_2SO_4, filtered and concentrated in vacuo. The residue was purified by flash column chromatography on silica gel (PE/EA = 50:1-10:1) to afford **1s** and **1an**.

Preparation of substrates 1ad-1ah, **1ao**[3].

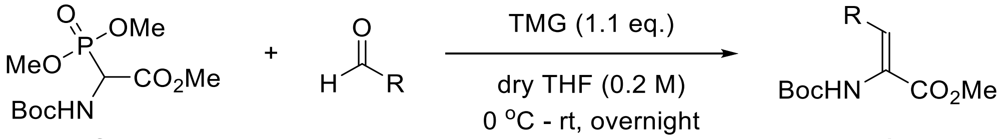

S6 **II** **1**

To a solution of aldehyde (10 mmol, 2.92 g) and phosphonate (11 mmol, 1.1 equiv.) in anhydrous THF (60 mL) was added *tetra*-methylguanidine (11 mmol, 1.27 g) slowly at 0 °C. The mixture was stirred at rt overnight, quenched with cold water (30 mL) at 0 °C and extracted with EA (20 mL). The combined organic extracts were washed with brine (20 mL), dried over Na_2SO_4, filtered and concentrated in vacuo. The residue was purified by flash column chromatography on silica gel (PE/EA = 50:1-10:1) to afford **1ad-1ah**, **1ao**.

Preparation of substrates 1ai-1aj.

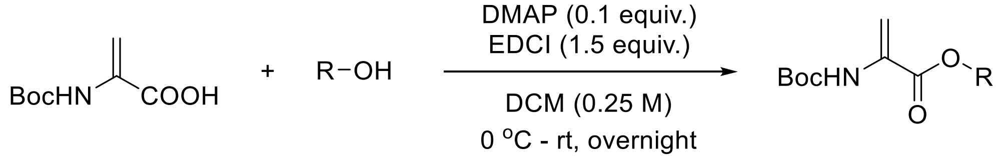

S1 **III** **1**

To a solution of **S1** (10 mmol, 1.55 g) and **III** in DCM (0.25 M) was added DMAP (2

mmol, 1.22 g). The mixture was stirred for 10 min at room temperature, and then, EDCI (15 mmol, 1.92 g) was added to the solution. The rection was stirred overnight. After regular workup, the reaction mixture washed by saturated $NaHCO_3$ solution (40 mL x 3), HCl (2M, 40 mL x 3) and H_2O (40 mL x 3). The organic layers were combined, dried over Na_2SO_4, and concentrated. The resulting crude product was purified by flash chromatography (PE/EA) to afford **1ai-1aj**.

3.Optimization studies

Table S1. Optimization of metal catalyst conditions [a,b]

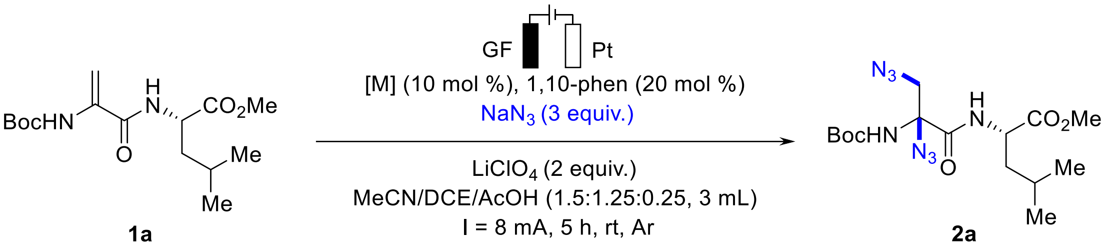

Entry	Deviation from standard conditions	Yield[b]
1	MnF_2	87%
2	No MnF_2	18%
3	$MnBr_2{\cdot}4H_2O$	75%
4	$MnCl_2{\cdot}4H_2O$	60%
5	$Mn(OAc)_2$	69%
6	$MnSO_4$	44%
7	$CuSO_4{\cdot}5H_2O$	45%
8	Cp_2Fe	NR
9	$NiCl_2{\cdot}$glyme	62%
10	$Co(OAc)_2{\cdot}4H_2O$	59%
11	$Cu(acac)_2$	39%
12	$FeCl_3$	NR

[a]Reaction conditions: undivided cell, GF anode and Pt cathode (1.5 cm × 1.0 cm × 0.2 cm), **1a** (0.2

mmol), [M] (10 mol%), 1,10-phen (20 mol%), NaN_3 (0.6 mmol), $LiClO_4$(0.4 mmol) and MeCN:DCE:AcOH (1.5:1.25:0.25, 3 mL), constant current = 8.0 mA, 4 h, rt, under Ar. [b]Isolated yield.

Table S2. Optimization of ligand conditions [a,b]

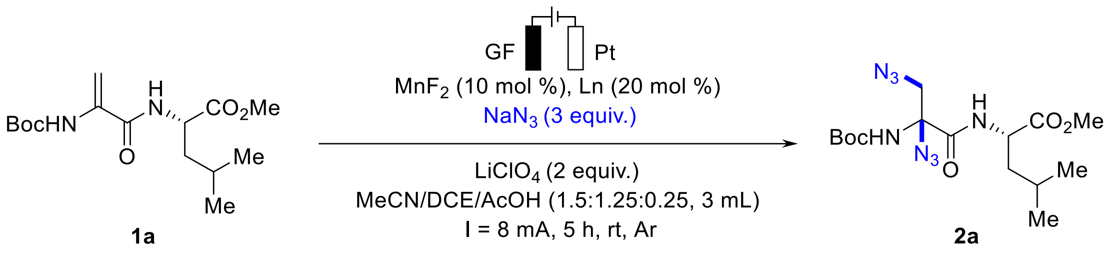

Entry	Deviation from standard conditions	yield(*dr*)[b]
1	L1	87% (1.5:1)
2	No L1	45% (1:1)
3	L2	51% (1:1)
4	L3	trace
5	L4	messy
6	L5	messy
7	L6	45% (1.2:1)
8	L7	60% (1:1)
9	L8	36% (1:1)
10	L9	25% (1.3:1)
11	L10	messy
12	L11	22% (1.2:1)

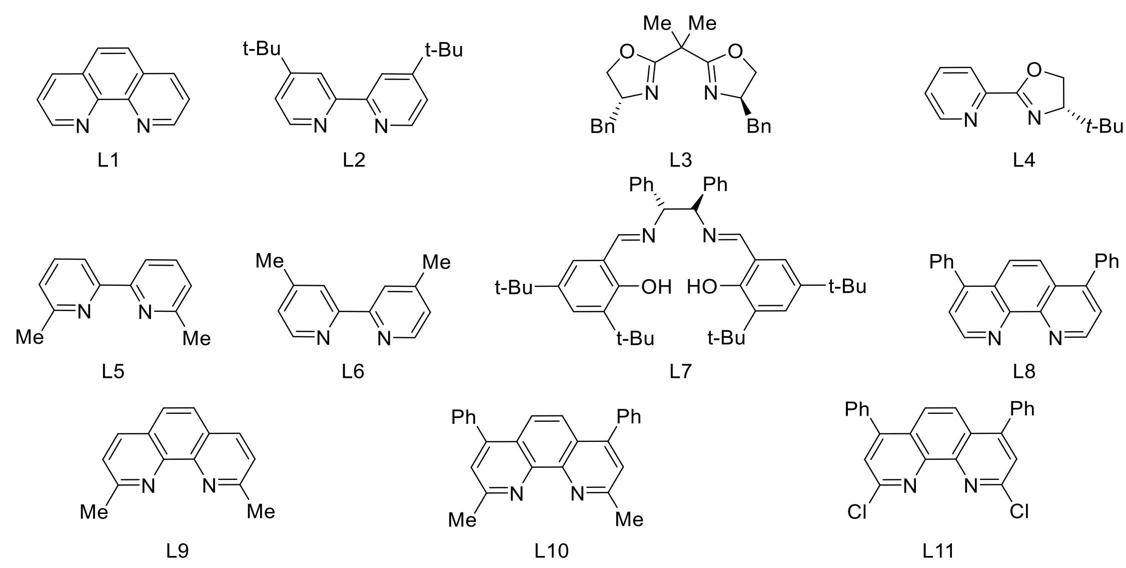

[a]Reaction conditions: undivided cell, GF anode and Pt cathode (1.5 cm × 1.0 cm × 0.2 cm), **1a** (0.2 mmol), MnF_2 (10 mol%), Ln (20 mol%), NaN_3 (0.6 mmol), $LiClO_4$ (0.4 mmol) and MeCN:DCE:AcOH (1.5:1.25:0.25, 3 mL), constant current = 8.0 mA, 4 h, rt, under Ar. [b]Isolated yield.

Table S3. Optimization of electrolyte and solvent conditions [a,b]

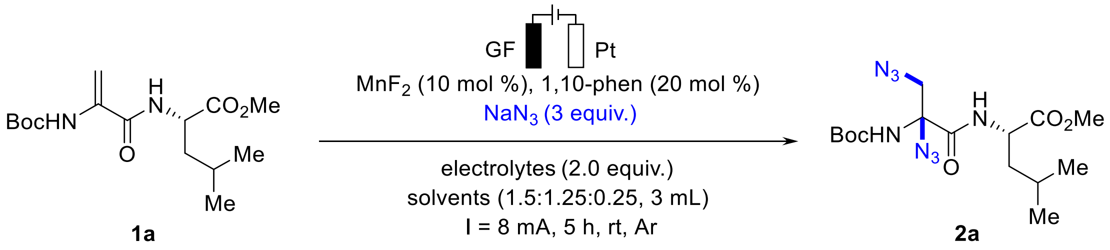

Entry	Deviation from standard conditions	Yield[b]
1	MeCN:(Tris-HCl): AcOH (1.5:1.25:0.25)	54%
2	MeCN: PBS Buffer: AcOH (1.5:1.25:0.25)	55%
3	MeCN:DCE (1.5:1.25)	NR
4	MeCN	NR
5	*n*-Bu_4NPF_6	35%
6	*n*-Bu_4NBF_4	21%

7	*n*-Bu_4NOAc	trace
8	*n*-Bu_4NClO_4	49%
9	*n*-Bu_4NHSO_4	29%
10	*n*-Bu_4NBr	NR
11	*n*-Bu_4NI	NR

[a]Reaction conditions: undivided cell, GF anode and Pt cathode (1.5 cm × 1.0 cm × 0.2 cm), **1a** (0.2 mmol), MnF_2 (10 mol%), 1,10-phen (20 mol%), NaN_3 (0.6 mmol), electrolytes (0.40 mmol) and solvents (3.0 mL), constant current = 8.0 mA, 4 h, rt, under Ar. [b]Isolated yield.

Table S4. Optimization of electrode material and electric current conditions[a,b]

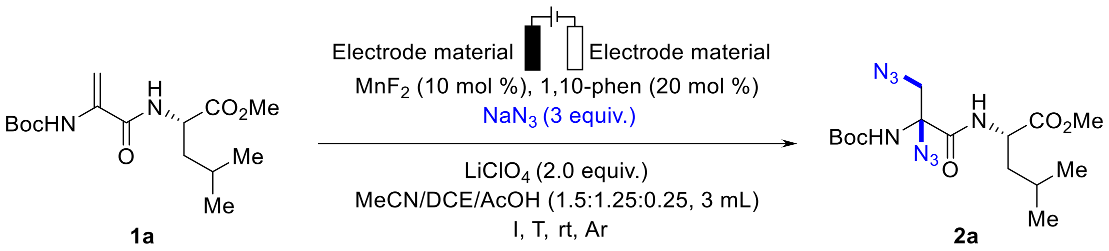

Entry	Deviation from standard conditions	yield[b]
1	C (+) / Pt (-)	57%
2	Pt (+) / Pt (-)	71%
3	GF (+) / GF (-)	73%
4	GF (+) / C (-)	NR
5	GF (+) / Ni (-)	NR
6	0	NR
7	20 mA, 1.5 h	79%
8	30 mA, 1 h	79%
9	40 mA, 0.8 h	80%
10	100mA, 0.32 h	NR

[a]Reaction conditions: undivided cell, electrode (1.5 cm × 1.0 cm × 0.2 cm), **1a** (0.2 mmol), MnF_2

(10 mol%), 1,10-phen (20 mol%), NaN_3 (0.6 mmol), $LiClO_4$ (0.4 mmol) and MeCN:DCE:AcOH (1.5:1.25:0.25, 3 mL) , rt, under Ar. [b]Isolated yield.

4. General procedure for electrochemical reactions

In an undivided cell (10 mL) equipped with a stirring bar, a mixture of substrates **1** (0.20 mmol), NaN_3 (0.6 mmol, 39 mg), MnF_2 (10 mol%, 2.0 mg), 1,10-phen (20 mol%, 7.0 mg), $LiClO_4$ (0.40 mmol, 41 mg). and MeCN:DCE:AcOH (1.5:1.25:0.25, 3.0 mL) were added. The reaction was then initiated by applying a constant cell current of 8.0 mA/cm^2 (Pt (-), GF (+)) at room temperature for 4 or 5 h under Ar. Upon completion, the solvent was removed directly under reduced pressure to afford the crude product, which was further purified by flash column chromatography to afford the desired products **2a-2ao**.

Characterization data of starting materials

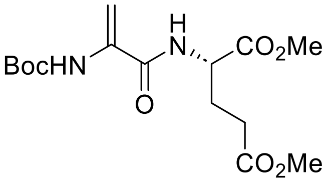

Dimethyl (2-((*tert*-butoxycarbonyl)amino)acryloyl)-*L*-glutamate (1j): a colourless liquid (24%, 826 mg). ^{1}H NMR (400 MHz, DMSO-d_6) δ 7.24 – 7.17 (m, 2H), 5.97 (s, 1H), 5.15 (t, J = 1.7 Hz, 1H), 4.58 – 4.53 (m, 1H), 3.70 (s, 3H), 3.62 (d, J = 3.4 Hz, 3H), 2.39 – 2.31 (m, 2H), 2.19 – 2.01 (m, 2H), 1.40 (s, 9H). ^{13}C NMR (100 MHz, DMSO-d_6) δ 173.8, 172.0, 164.1, 152.8, 134.2, 98.6, 80.6, 52.7, 52.5, 52.1, 30.2, 28.3, 26.7. HR-MS (ESI) m/z calcd for $C_{15}H_{24}N_2NaO_7$ $[M+Na]^+$ 367.1476, found 367.1474.

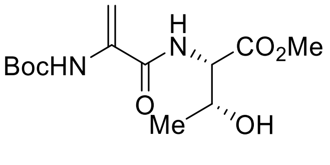

Methyl (2-((*tert*-butoxycarbonyl)amino)acryloyl)-*L*-threoninate (1m): a colourless liquid (60%, 1814 mg). ^{1}H NMR (400 MHz, $CDCl_3$) δ = 7.03 (d, J = 8.8 Hz, 1H), 6.03 (s, 1H), 5.27 (q, J = 1.5 Hz, 1H), 4.61 (dd, J = 8.8, 1.8 Hz, 1H), 4.39 (qd, J = 7.0, 2.4 Hz, 1H), 3.77 (d, J = 1.1 Hz, 3H), 1.46 (s, 9H), 1.22 (dd, J = 6.5, 1.1 Hz, 3H). ^{13}C NMR (100 MHz, $CDCl_3$) δ = 171.2, 164.8, 152.9, 134.5, 99.2, 80.8, 68.0, 57.9, 52.8, 28.3, 20.1. HR-MS (ESI) m/z calcd for $C_{13}H_{22}N_2NaO_6$ $[M+Na]^+$ 325.1370, found 325.1370.

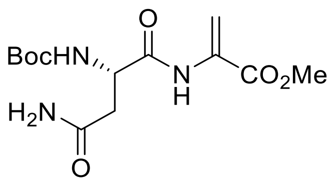

Methyl (*S*)-2-(4-amino-2-((*tert*-butoxycarbonyl)amino)-4-oxobutanamido)-acrylate (1n) [1,2]**:** a colourless liquid (55%, 1734 mg). ^{1}H NMR (400 MHz, CDCl$_3$) δ = 8.64 (s, 1H), 6.59 (s, 1H), 5.96 (d, J = 1.4 Hz, 1H), 5.58 (d, J = 8.8 Hz, 1H), 4.59 (t, J = 7.3 Hz, 1H), 3.84 (s, 3H), 3.01 – 2.89 (m, 2H), 1.47 (s, 9H). ^{13}C NMR (100 MHz, CDCl$_3$) δ = 167.5, 164.1, 155.3, 130.6, 116.9, 110.4, 81.8, 53.3, 51.5, 38.7, 28.3.

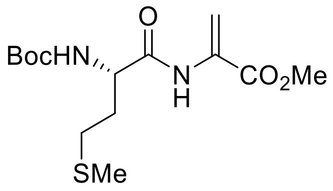

Methyl (*S*)-2-(2-((*tert*-butoxycarbonyl)amino)-4-(methylthio)butanamido)-acrylate (1o): a colourless liquid (70%, 2327 mg). ^{1}H NMR (400 MHz, CDCl$_3$) δ = 8.49 (s, 1H), 6.59 (s, 1H), 5.92 (s, 1H), 5.24 (d, J = 8.2 Hz, 1H), 4.38 (d, J = 8.2 Hz, 1H), 3.84 (s, 3H), 2.58 (td, J = 7.0, 4.8 Hz, 2H), 2.11 (s, 4H), 1.96 (dt, J = 14.3, 7.2 Hz, 1H), 1.45 (s, 9H).^{13}C NMR (100 MHz, CDCl$_3$) δ = 170.6, 164.3, 155.6, 130.9, 109.7, 80.6, 54.4, 53.1, 31.5, 30.3, 28.4, 15.4.HR-MS (ESI) *m/z* calcd for $C_{14}H_{24}N_2NaO_5S$ [M+Na]$^+$ 355.1299, found 355.1293.

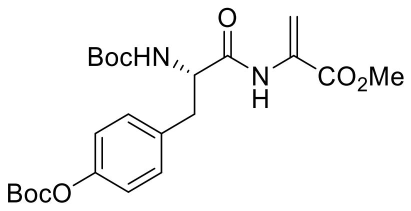

Methyl (*S*)-2-(2-((*tert*-butoxycarbonyl)amino)-3-(4-((*tert*-butoxycarbonyl)oxy)-phenyl)propanamido)acrylate (1q): a colourless liquid (53%, 2.462 mg). ^{1}H NMR (500 MHz, CDCl$_3$) δ = 7.22 (s, 1H), 7.10 – 7.05 (m, 4H), 6.59 (d, J = 7.6 Hz, 1H), 5.99 (s, 1H), 5.01 (t, J = 1.8 Hz, 1H), 5.01 – 4.84 (m, 1H), 3.72 (s, 3H), 3.20 – 3.09 (m, 2H), 1.53 (s, 9H), 1.46 (s, 9H).^{13}C NMR (100 MHz, CDCl$_3$) δ = 171.4, 163.6, 152.7, 151.8, 150.4, 134.4, 133.0, 130.3, 121.5, 98.5, 83.7, 80.7, 53.7, 52.7, 37.1, 28.3, 27.8. HR-MS (ESI) *m/z* calcd for $C_{23}H_{33}N_2O_8$ [M+H]$^+$ 465.2231, found 465.2229.

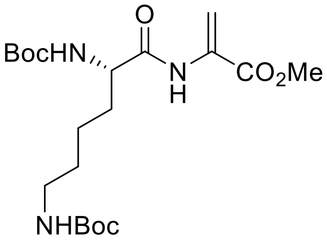

Methyl (*S*)-2-(2,6-*bis*((*tert*-butoxycarbonyl)amino)hexanamido)acrylate (1r): a light liquid (63%, 2706 mg). ^{1}H NMR (400 MHz, $CDCl_3$) δ = 8.45 (s, 1H), 6.50 (d, J = 1.4 Hz, 1H), 5.82 (s, 1H), 5.60 – 5.49 (m, 1H), 4.84 (t, J = 6.0 Hz, 1H), 4.12 (s, 1H), 3.75 (d, J = 1.4 Hz, 3H), 3.03 (q, J = 6.7 Hz, 2H), 1.81 (tt, J = 10.1, 5.2 Hz, 1H), 1.67 – 1.58 (m, 1H), 1.43 (q, J = 5.9, 5.2 Hz, 2H), 1.37 (s, 9H), 1.36 (s, 9H), 1.32 – 1.17 (m, 2H).^{13}C NMR (100 MHz, $CDCl_3$) δ = 171.5, 164.3, 156.3, 130.9, 109.4, 80.2, 79.0, 55.4, 52.9, 39.8, 31.6, 31.5, 29.8, 28.5, 28.3, 22.6.HR-MS (ESI) *m/z* calcd for $C_{20}H_{35}N_3NaO_7$ $[M+Na]^+$ 452.2367, found 452.2366.

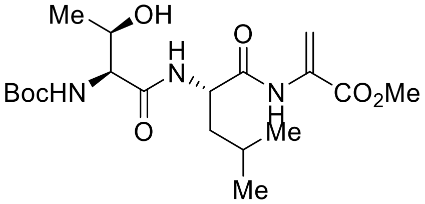

Methyl (6*S*,9*S*)-6-((*R*)-1-hydroxyethyl)-9-isobutyl-2,2-dimethyl-12-methylene-4,7, 10-trioxo-3-oxa-5,8,11-triazatridecan-13-oate (1u): a white liquid (41%, 1704 mg). ^{1}H NMR (400 MHz, $CDCl_3$) δ = 8.48 (s, 1H), 7.28 (d, J = 7.8 Hz, 1H), 6.53 (d, J = 2.5 Hz, 1H), 5.87 (s, 1H), 5.68 (d, J = 8.1 Hz, 1H), 4.53 (d, J = 8.3 Hz, 1H), 4.26 (dd, J = 6.7, 3.2 Hz, 1H), 4.17 (dd, J = 8.1, 2.7 Hz, 1H), 3.90 (s, 1H), 3.80 (d, J = 2.4 Hz, 3H), 1.69 – 1.62 (m, 2H), 1.61 – 1.56 (m, 1H), 1.40 (d, J = 2.4 Hz, 9H), 1.13 (d, J = 6.4 Hz, 3H), 0.91 – 0.86 (m, 6H).^{13}C NMR (100 MHz, $CDCl_3$) δ = 171.9, 171.0, 164.5, 156.5, 130.9, 110.1, 80.4, 67.1, 58.0, 53.1, 52.7, 40.4, 28.4, 24.7, 23.1, 21.7, 18.2. HR-MS (ESI) *m/z* calcd for $C_{19}H_{33}N_3NaO_7$ $[M+Na]^+$ 438.2211, found 438.2207.

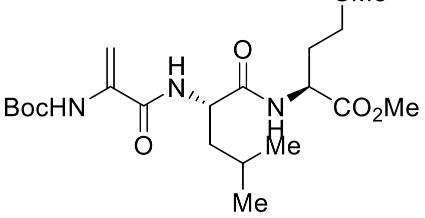

Methyl (2-((*tert*-butoxycarbonyl)amino)acryloyl)-*L*-leucyl-*L*-methioninate (1v): a

white soild (67%, 2985 mg). ^{1}H NMR (400 MHz, CDCl$_3$) δ = 7.24 (s, 1H), 7.04 (dd, J = 15.4, 8.2 Hz, 2H), 5.95 (s, 1H), 5.16 (s, 1H), 4.70 – 4.45 (m, 2H), 3.72 (s, 3H), 2.45 (t, J = 7.4 Hz, 2H), 2.15 – 2.06 (m, 1H), 2.03 (s, 3H), 1.95 (dq, J = 14.5, 7.3 Hz, 1H), 1.65 – 1.57 (m, 3H), 1.43 (s, 9H), 0.89 (t, J = 6.4 Hz, 6H).^{13}C NMR (100 MHz, CDCl$_3$) δ = 172.3, 171.9, 164.3, 152.8, 134.5, 99.1, 80.7, 52.7, 52.3, 51.7, 41.2, 31.3, 28.3, 24.8, 23.0, 22.1, 15.5. HR-MS (ESI) *m/z* calcd for $C_{25}H_{30}N_3NaO_6S$ [M+Na]$^+$ 468.2139, found 468.2136.

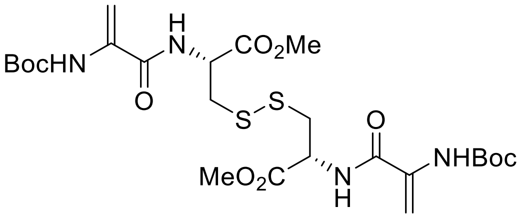

Methyl (9*R*,14*R*)-14-(2-((*tert*-butoxycarbonyl)amino)acrylamido)-9-(methoxycarbonyl)-2,2-dimethyl-6-methylene-4,7-dioxo-3-oxa-11,12-dithia-5,8-diazapentade can-15-oate (1w): a green liquid (66%, 4004 mg). ^{1}H NMR (400 MHz, CDCl$_3$) δ = 7.09 (d, J = 7.5 Hz, 2H), 6.06 (s, 2H), 5.23 (s, 2H), 4.89 (q, J = 5.9 Hz, 2H), 3.78 (s, 6H), 3.23 (d, J = 4.5 Hz, 4H), 1.46 (s, 18H). ^{13}C NMR (100 MHz, CDCl$_3$) δ = 170.5, 164.1, 152.8, 134.3, 99.3, 80.8, 53.1, 52.4, 40.5, 28.3. HR-MS (ESI) *m/z* calcd for $C_{23}H_{38}N_4NaO_{10}S_2$ [M+Na]$^+$ 629.1922, found 629.1925.

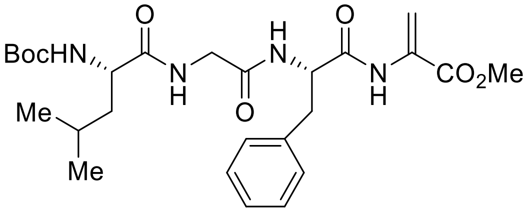

Methyl (6*S*, 12*S*)-12-benzyl-6-isobutyl-2,2-dimethyl-15-methylene-4,7,10,13-*tetra*-oxo-3-oxa-5,8,11,14-tetraazahexadecan-16-oate (1x): a green liquid (30%, 1556 mg). ^{1}H NMR (400 MHz, CDCl$_3$) δ = 8.45 (s, 1H), 7.68 (d, J = 8.0 Hz, 1H), 7.43 (t, J = 5.4 Hz, 1H), 7.22 (d, J = 7.2 Hz, 2H), 7.17 (t, J = 6.6 Hz, 3H), 6.54 (s, 1H), 5.87 (s, 1H), 5.57 (d, J = 8.2 Hz, 1H), 4.95 (q, J = 7.4 Hz, 1H), 4.24 (q, J = 7.9 Hz, 1H), 3.99 (td, J = 16.5, 5.5 Hz, 2H), 3.76 (d, J = 1.6 Hz, 3H), 3.13 (dd, J = 13.9, 6.6 Hz, 1H), 3.00 (dd, J = 13.8, 7.5 Hz, 1H), 1.68 – 1.57 (m, 2H), 1.50 (dt, J = 8.5, 4.4 Hz, 1H), 1.40 (s, 9H), 0.91 (dd, J = 6.4, 3.0 Hz, 6H).^{13}C NMR (100 MHz, CDCl$_3$) δ = 173.7, 170.2, 169.4,

164.2, 156.0, 136.4, 130.9, 129.4, 128.7, 127.0, 110.3, 79.9, 60.5, 55.4, 53.0, 43.0, 41.7, 38.4, 28.4, 24.8, 23.2, 21.9. HR-MS (ESI) *m/z* calcd for $C_{26}H_{38}N_4NaO_7$ $[M+Na]^+$ 541.2633, found 541.2632.

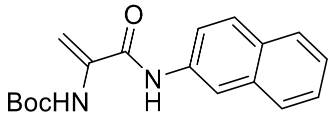

***Tert*-butyl (3-(naphthalen-2-ylamino)-3-oxoprop-1-en-2-yl)carbamate (1ac):** a colourless liquid (48%, 1499 mg). ^1H NMR (500 MHz, $CDCl_3$) δ = 8.15 (d, J = 2.1 Hz, 1H), 8.08 (s, 1H), 7.79 – 7.75 (m, 3H), 7.50 – 7.40 (m, 3H), 7.36 (s, 1H), 6.13 (s, 1H), 5.28 (t, J = 1.8 Hz, 1H), 1.49 (d, J = 0.8 Hz, 9H). ^{13}C NMR (126 MHz, $CDCl_3$) δ = 162.5, 152.9, 135.6, 134.5, 133.8, 131.0, 129.0, 127.8, 127.7, 126.8, 125.5, 120.1, 117.6, 98.7, 81.0, 28.4. HR-MS (ESI) *m/z* calcd for $C_{18}H_{20}N_2NaO_3$ $[M+Na]^+$ 335.1367, found 335.1362.

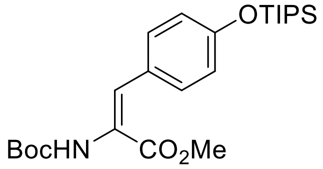

Methyl 2-((*tert*-butoxycarbonyl)amino)-3-(4-((triisopropylsilyl)oxy)phenyl)-acrylate (1ae) [3]**:** a colourless liquid (62%, 2788 mg). ^1H NMR (400 MHz, $CDCl_3$) δ = 7.45 (d, J = 8.3 Hz, 2H), 7.22 (s, 1H), 6.83 (d, J = 8.7 Hz, 2H), 6.13 (s, 1H), 3.80 (s, 3H), 1.39 (s, 9H), 1.25 – 1.23 (m, 3H), 1.07 (d, J = 7.3 Hz, 18H). ^{13}C NMR (100 MHz, $CDCl_3$) δ = 166.5, 157.3, 153.1, 131.7, 127.0, 120.1, 80.9, 52.5, 28.2, 18.0, 12.7.

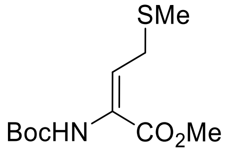

Methyl -2-((*tert*-butoxycarbonyl)amino)-4-(methylthio)but-2-enoate (1ag): a white solid (67%, 1751 mg). ^1H NMR (400 MHz, $CDCl_3$) δ = 6.51 (t, J = 7.6 Hz, 1H), 6.24 (s, 1H), 3.76 (s, 3H), 3.23 (d, J = 7.6 Hz, 2H), 2.03 (s, 3H), 1.43 (s, 9H). ^{13}C NMR (100 MHz, $CDCl_3$) δ = 165.15, 153.31, 130.83, 118.31, 81.01, 52.61, 31.27, 28.23, 15.15. HR-MS (ESI) *m/z* calcd for $C_{11}H_{19}NNaO_4S$ $[M+Na]^+$ 284.0927, found 284.0928.

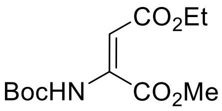

4-Ethyl 1-methyl 2-((*tert*-butoxycarbonyl)amino)maleate (1ah): a colourless liquid (69%, 1886 mg). ^{1}H NMR (400 MHz, $CDCl_3$) δ = 9.45 (s, 1H), 5.33 (s, 1H), 4.16 (q, J = 7.1 Hz, 2H), 3.82 (s, 3H), 1.43 (s, 9H), 1.25 (t, J = 7.1 Hz, 3H). ^{13}C NMR (100 MHz, $CDCl_3$) δ = 168.0, 164.5, 151.3, 144.7, 99.3, 82.5, 60.8, 53.1, 28.0, 14.2. HR-MS (ESI) *m/z* calcd for $C_{12}H_{19}NNaO_6$ $[M+Na]^+$ 296.1105, found 296.1104.

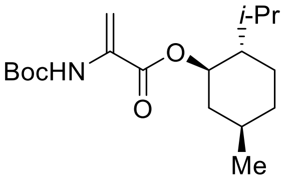

(1*R*,2*S*,5*R*)-2-Isopropyl-5-methylcyclohexyl-2-((*tert*-butoxycarbonyl)amino)-acrylate (1ai): a brown liquid (15%, 488 mg). ^{1}H NMR (400 MHz, $CDCl_3$) δ = 7.05 (s, 1H), 6.12 (s, 1H), 5.70 (s, 1H), 4.79 (td, J = 10.9, 4.4 Hz, 1H), 2.03 – 1.96 (m, 1H), 1.87 – 1.80 (m, 1H), 1.74 – 1.64 (m, 3H), 1.48 (s, 9H), 1.34 – 1.23 (m, 1H), 1.06 (t, J = 11.7 Hz, 2H), 0.90 (dd, J = 9.5, 6.8 Hz, 7H), 0.75 (d, J = 6.9 Hz, 3H). ^{13}C NMR (100 MHz, $CDCl_3$) δ = 163.7, 152.8, 131.8, 104.6, 80.7, 47.2, 40.7, 34.2, 31.5, 28.4, 26.5, 23.7, 22.1, 20.8, 16.6. HR-MS (ESI) *m/z* calcd for $C_{18}H_{31}NaO_4$ $[M+Na]^+$ 348.2146, found 348.2140.

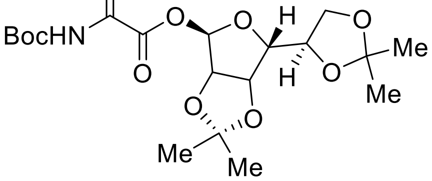

(4*R*,6*R*)-6-((*R*)-2,2-dimethyl-1,3-dioxolan-4-yl)-2,2-dimethyltetrahydrofuro[3,4-d][1,3]dioxol-4-yl 2-((*tert*-butoxycarbonyl)amino)acrylate(1aj): a white soild (16%, 687 mg) . ^{1}H NMR (500 MHz, $CDCl_3$) δ = 6.96 (s, 1H), 6.19 (s, 1H), 5.66 (d, J = 1.4 Hz, 1H), 4.88 (dd, J = 5.9, 3.6 Hz, 1H), 4.77 (d, J = 5.9 Hz, 1H), 4.39 (ddd, J = 7.9, 6.2, 4.2 Hz, 1H), 4.12 – 4.06 (m, 2H), 4.03 (dd, J = 7.9, 3.6 Hz, 1H), 4.00 (dd, J = 8.9, 4.2 Hz, 1H), 1.48 (s, 3H), 1.46 (s, 9H), 1.43 (s, 3H), 1.35 (s, 3H), 1.33 (s, 3H). ^{13}C NMR (126 MHz, $CDCl_3$) δ = 162.8, 152.6, 131.1, 113.5, 109.5, 106.2, 102.3, 85.0, 82.8, 79.3,

72.8, 66.8, 60.5, 28.3, 27.0, 26.0, 25.2, 24.7. HR-MS (ESI) *m/z* calcd for $C_{20}H_{31}NaO_9$ $[M+Na]^+$ 452.1892, found 452.1888.

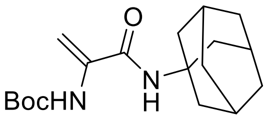

***Tert*-butyl (3-(adamantan-1-ylamino)-3-oxoprop-1-en-2-yl)carbamate (1ak):** a colourless liquid (60%, 1923 mg). ^{1}H NMR (400 MHz, $CDCl_3$) δ = 7.30 (s, 1H), 5.90 – 5.87 (m, 1H), 5.78 (s, 1H), 4.91 (t, J = 1.6 Hz, 1H), 2.09 – 2.06 (m, 3H), 1.99 (d, J = 2.9 Hz, 6H), 1.67 (t, J = 3.2 Hz, 6H), 1.44 (s, 9H). ^{13}C NMR (100 MHz, $CDCl_3$) δ = 163.0, 152.9, 135.9, 96.3, 80.4, 52.5, 41.4, 36.3, 29.4, 28.3. HR-MS (ESI) *m/z* calcd for $C_{12}H_{28}N_2NaO_3$ $[M+Na]^+$ 343.1993, found 343.1991.

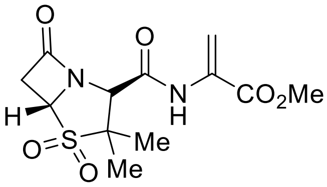

Methyl 2-((2*R*,5*R*)-3,3-dimethyl-4,4-dioxido-7-oxo-4-thia-1-azabicyclo[3.2.0]-heptane-2-carboxamido)acrylate (1al): a colourless liquid (60%, 1898 mg). ^{1}H NMR (400 MHz, $CDCl_3$) δ = 8.78 (s, 1H), 6.64 (s, 1H), 5.99 (d, J = 1.5 Hz, 1H), 4.69 (dd, J = 4.7, 2.3 Hz, 1H), 4.27 (s, 1H), 3.86 (d, J = 1.0 Hz, 3H), 3.64 – 3.51 (m, 2H), 1.68 (s, 3H), 1.43 (s, 3H). ^{13}C NMR (100 MHz, $CDCl_3$) δ = 172.3, 165.2, 164.3, 130.1, 110.7, 64.6, 63.7, 60.7, 53.3, 38.5, 19.6, 18.4. HR-MS (ESI) *m/z* calcd for $C_{12}H_{16}N_2NaO_6$ $[M+Na]^+$ 339.0622, found 339.0617.

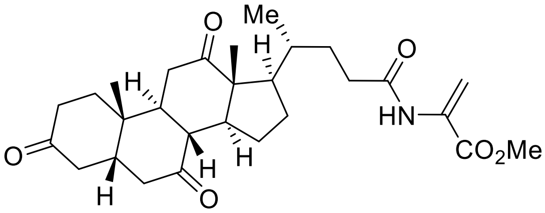

Methyl 2-((4*S*)-4-((5*R*,8*R*,9*S*,10*R*,13*S*,17*S*)-10,13-dimethyl-3,7,12-trioxohexadeca-hydro-1H-cyclopenta[a]phenanthren-17-yl)pentanamido)acrylate (1am): a colourless liquid (63%, 3059 mg). ^{1}H NMR (400 MHz, $CDCl_3$) δ = 7.72 (s, 1H), 6.61 (d, J = 1.1 Hz, 1H), 5.88 (q, J = 1.2 Hz, 1H), 3.86 (d, J = 1.2 Hz, 3H), 2.90 (ddd, J = 18.2, 12.9, 6.6 Hz, 3H), 2.35 – 2.07 (m, 10H), 2.06 – 2.00 (m, 3H), 1.88 (qd, J = 11.2,

10.7, 5.1 Hz, 2H), 1.62 (s, 2H), 1.48 – 1.44 (m, 1H), 1.41 (d, *J* = 1.2 Hz, 3H), 1.36 – 1.24 (m, 3H), 1.08 (d, *J* = 1.2 Hz, 3H), 0.88 (dd, *J* = 6.6, 1.2 Hz, 3H). ^{13}C NMR (100 MHz, $CDCl_3$) δ = 212.1, 209.2, 208.8, 172.2, 164.8, 131.0, 108.7, 57.0, 53.1, 51.9, 49.1, 47.0, 45.8, 45.7, 45.1, 42.9, 38.7, 36.6, 36.1, 35.6, 35.4, 34.8, 30.8, 27.7, 25.3, 22.0, 18.9, 12.0. HR-MS (ESI) *m/z* calcd for $C_{28}H_{40}NO_6$ $[M+H]^+$ 486.2851, found 486.2853.

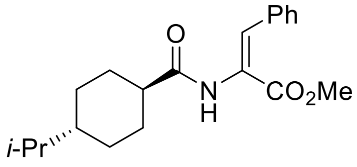

Methyl (*E*)-2-((1*r*,4*r*)-4-isopropylcyclohexane-1-carboxamido)-3-phenylacrylate (1an): a colourless liquid (51%, 1680 mg). ^{1}H NMR (500 MHz, $CDCl_3$) δ = 7.41 (d, *J* = 7.3 Hz, 2H), 7.33 – 7.28 (m, 4H), 7.10 (s, 1H), 3.80 (s, 3H), 2.20 – 2.14 (m, 1H), 2.01 – 1.95 (m, 2H), 1.81 – 1.75 (m, 2H), 1.50 – 1.45 (m, 2H), 1.43 – 1.38 (m, 1H), 1.05 – 0.95 (m, 3H), 0.85 (d, *J* = 6.8 Hz, 6H). ^{13}C NMR (126 MHz, $CDCl_3$) δ = 174.5, 166.0, 134.1, 131.7, 129.7, 129.4, 128.5, 124.4, 52.7, 45.8, 43.3, 32.8, 29.5, 29.0, 19.8. HR-MS (ESI) *m/z* calcd for $C_{20}H_{27}NO_3$ $[M+H]^+$ 352.1889, found 352.1880.

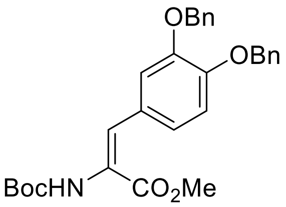

Methyl (*E*)-2-((1*r*,4*r*)-4-isopropylcyclohexane-1-carboxamido)-3-phenylacrylate (1ao): a colourless liquid (72%, 3525 mg). ^{1}H NMR (500 MHz, $CDCl_3$) δ = 7.48 – 7.40 (m, 5H), 7.40 – 7.33 (m, 5H), 7.32 – 7.29 (m, 2H), 7.27 (d, *J* = 2.1 Hz, 1H), 7.11 (dd, *J* = 8.4, 1.8 Hz, 1H), 6.89 (d, *J* = 8.4 Hz, 1H), 5.18 (s, 2H), 5.14 (s, 2H), 3.81 (s, 3H), 1.43 (s, 9H).^{13}C NMR (126 MHz, $CDCl_3$) δ = 166.4, 150.1, 148.6, 137.1, 136.9, 131.4, 128.7, 128.0, 128.0, 127.5, 127.4, 127.3, 124.7, 116.1, 114.1, 81.0, 71.3, 70.9, 52.6, 28.3. HR-MS (ESI) *m/z* calcd for $C_{29}H_{31}NNaO_6$ $[M+H]^+$ 512.2044, found 512.2041.

Characterization Data of Products

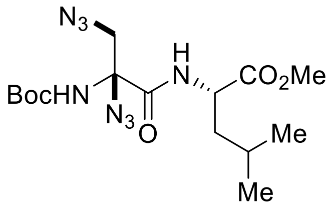

***Tert*-butyl (2,3-diazido-2-((*tert*-butoxycarbonyl)amino)propanoyl)glycinate (2a):** The general procedure was followed using substrate **1a** (0.20 mmol, 70 mg). Isolation by column chromatography (PE/EA: 10/1→5/1) yielded **2a** (60 mg, 87%, *dr* = 1.4:1) as a colourless liquid. ^{1}H NMR (400 MHz, $CDCl_3$) δ 7.02 (d, J = 8.1 Hz, 1H), 6.03 (s, 0.41H), 5.95 (s, 0.57H), 4.60 - 4.55 (m, 1H), 3.73 (d, J = 6.4 Hz, 5H), 1.70 – 1.59 (m, 3H), 1.43 (s, 9H), 0.94 – 0.91 (m, 6H). ^{13}C NMR (100 MHz, $CDCl_3$) δ 172.7, 172.6, 166.5, 166.4, 153.6, 81.8, 76.2, 55.8, 52.6, 52.6, 51.6, 41.4, 28.1, 28.1, 24.9, 24.7, 22.9, 22.9, 21.9, 21.8. HR-MS (ESI) *m/z* calcd for $C_{15}H_{26}N_8KO_5^+$ $[M+K]^+$ 437.1658, found 437.1658.

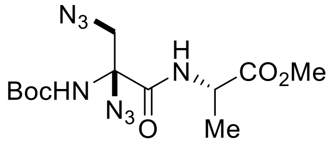

Methyl (2,3-diazido-2-((*tert*-butoxycarbonyl)amino)propanoyl)-*L*-alaninate (2b): The general procedure was followed using substrate **1b** (0.20 mmol, 54 mg). Isolation by column chromatography (PE/EA: 10/1→5/1) yielded **2b** (53 mg, 75%, *dr* = 1.2:1) as colourless liquid. ^{1}H NMR (400 MHz, $CDCl_3$) δ 7.25 (d, J = 6.9 Hz, 0.61H), 7.17 (d, J = 6.9 Hz, 0.50H), 6.05 (s, 0.45H), 5.99 (s, 0.53H), 4.56 – 4.49 (m, 1H), 3.74 (d, J = 7.6 Hz, 5H), 1.49 – 1.34 (m, 12H). ^{13}C NMR (100 MHz, $CDCl_3$) δ 172.7, 172.6, 166.2, 166.1, 153.6, 81.9, 76.2, 56.0, 52.8, 52.7, 49.0, 48.9, 28.1, 18.1, 17.9. HR-MS (ESI) *m/z* calcd for $C_{12}H_{20}N_8NaO_5^+$ $[M+Na]^+$ 379.1449, found 379.1447.

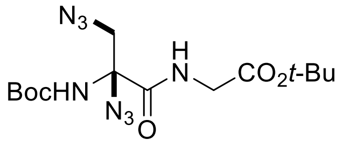

***Tert*-butyl (2,3-diazido-2-((*tert*-butoxycarbonyl)amino)propanoyl)glycinate (2c):** The general procedure was followed using substrate **1c** (0.20 mmol, 61 mg). Isolation by column chromatography (PE/EA: 10/1→5/1) yielded **2c** (60 mg, 78%) as colourless

liquid. ^{1}H NMR (400 MHz, $CDCl_3$) δ 7.18 (s, 1H), 6.02 (s, 1H), 4.04 – 3.84 (m, 2H), 3.73 (d, J = 8.4 Hz, 2H), 1.46 (s, 9H), 1.43 (s, 9H). ^{13}C NMR (100 MHz, $CDCl_3$) δ 168.2, 166.6, 153.6, 82.9, 81.9, 76.2, 55.8, 42.6, 28.1. HR-MS (ESI) *m/z* calcd for $C_{14}H_{24}N_8NaO_5^+$ $[M+Na]^+$ 407.1762, found 407.1762.

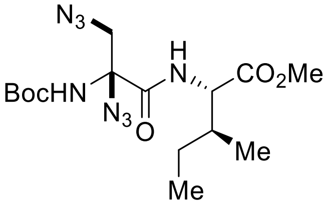

Methyl (2,3-diazido-2-((*tert*-butoxycarbonyl)amino)propanoyl)-*L*-isoleucinate (2d): The general procedure was followed using substrate **1d** (0.20 mmol, 63 mg). Isolation by column chromatography (PE/EA: 10/1→5/1) yielded **2d** (61 mg, 76%, *dr* = 1:1) as colourless liquid. ^{1}H NMR (400 MHz, DMSO-d_6) δ 8.31 – 7.83 (m, 2H), 4.25 (t, J = 7.6, 0.5H), 4.16 (t, J = 7.6, 0.5H), 3.67 – 3.55 (m, 5H), 1.87 – 1.85 (m, 1H), 1.37 (s, 4.5H), 1.36 (s, 4.5H), 1.30 – 1.04 (m, 2H), 0.82 – 0.78 (m, 6H). ^{13}C NMR (100 MHz, DMSO-d_6) δ 172.1, 172.0, 166.7, 166.5, 154.6, 154.4, 80.4, 77.6, 77.4, 57.8, 57.4, 55.6, 52.3, 36.7, 36.4, 28.3, 25.3, 25.1, 15.8, 11.4, 11.2. HR-MS (ESI) *m/z* calcd for $C_{15}H_{26}N_8NaO_5^+$ $[M+Na]^+$ 547.1919, found 547.1916.

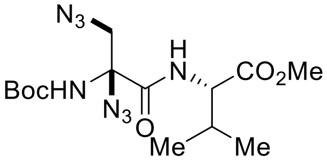

Methyl (2,3-diazido-2-((*tert*-butoxycarbonyl)amino)propanoyl)-*L*-valinate (2e): The general procedure was followed using substrate **1e** (0.20 mmol, 61 mg). Isolation by column chromatography (PE/EA: 10/1→5/1) yielded **2e** (71 mg, 92%, *dr* = 1.3:1) as colourless liquid. ^{1}H NMR (400 MHz, $CDCl_3$) δ 7.12 (d, J = 7.1 Hz, 0.96H), 6.06 (s, 0.40H), 6.00 (s, 0.52H), 4.52 - 4.46 (m, 1H), 3.74 (dd, J = 7.0, 1.2 Hz, 5H), 2.29 – 2.12 (m, 1H), 1.43 (s, 10H), 0.99 – 0.74 (m, 6H). ^{13}C NMR (100 MHz, $CDCl_3$) δ 171.7, 171.6, 166.6, 166.5, 153.6, 153.6, 81.8, 76.3, 58.1, 58.0, 55.7, 52.5, 52.5, 31.4, 31.4, 28.1, 19.0, 17.8. HR-MS (ESI) *m/z* calcd for $C_{14}H_{24}N_8NaO_5^+$ $[M+Na]^+$ 407.1762, found 407.1759.

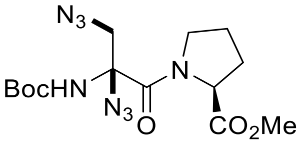

Methyl (2,3-diazido-2-((*tert*-butoxycarbonyl)amino)propanoyl)-*L*-prolinate (2f): The general procedure was followed using substrate **1f** (0.20 mmol, 60 mg). Isolation by column chromatography (PE/EA: 10/1→5/1) yielded **2f** (64 mg, 84%, *dr* = 1.6:1) as colourless liquid. ^{1}H NMR (400 MHz, $CDCl_3$) δ 6.74 (s, 0.34H), 6.56 (s, 0.56H), 4.61 – 4.47 (m, 1H), 3.77 (s, 1H), 3.73 (s, 4H), 3.59 - 3.56 (m, 1.57H), 3.42 (d, J = 13.2 Hz, 0.54H), 2.26 - 2.13 (m, 2H), 2.04 – 1.95 (m, 2H), 1.46 (d, J = 3.9 Hz, 9H). ^{13}C NMR (100 MHz, $CDCl_3$) δ 172.0, 172.0, 164.8, 164.8, 153.4, 81.4, 75.8, 61.4, 52.6, 52.5, 48.5, 48.4, 28.2, 28.1, 26.0, 25.6. HR-MS (ESI) *m/z* calcd for $C_{14}H_{22}N_8NaO_5^+$ [M+Na]$^+$ 405.1606, found 405.1600.

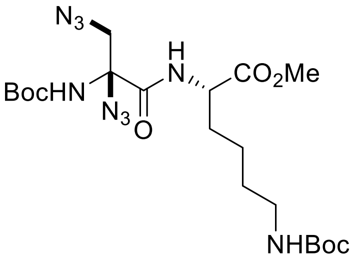

Methyl (2,3-diazido-2-((*tert*-butoxycarbonyl)amino)propanoyl)-*L*-methioninate (2g): The general procedure was followed using substrate **1g** (0.20 mmol, 86 mg). Isolation by column chromatography (PE/EA: 10/1→5/1) yielded **2g** (97 mg, 82%, *dr* = 1:1) as colourless liquid. ^{1}H NMR (400 MHz, $CDCl_3$) δ 7.21 (s, 0.4H), 7.19 (s, 0.4H), 6.07 (s, 0.5H), 5.98 (s, 0.4H), 4.81 (s, 0.3H), 4.65 (s, 0.4H), 4.58 – 4.51 (m 1H), 3.89 – 3.74 (m, 4H), 3.69 (s, 1H), 3.14 – 3.01 (m, 2H), 1.95 – 1.85 (m, 1H), 1.77 – 1.63 (m, 1H), 1.51 – 1.43 (m, 2H), 1.43 (s, 9H), 1.41 (s, 9H), 1.32 – 1.22 (m, 2H). ^{13}C NMR (100 MHz, $CDCl_3$) δ 172.1, 172.0, 166.5, 166.1, 156.1, 153.6, 81.8, 79.2, 79.1, 76.3, 76.2, 56.2, 52.8, 52.7, 40.2, 40.1, 31.8, 31.6, 29.4, 29.2, 28.5, 28.1, 22.3, 22.1. HR-MS (ESI) *m/z* calcd for $C_{20}H_{35}N_9NaO_7^+$ [M+Na]$^+$ 536.2552, found 536.2548.

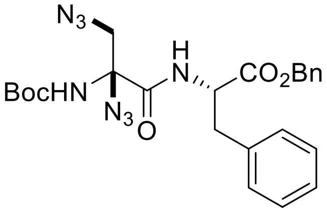

Methyl (2,3-diazido-2-((*tert*-butoxycarbonyl)amino)propanoyl)-*L*-methioninate (2h): The general procedure was followed using substrate **1h** (0.20 mmol, 85 mg). Isolation by column chromatography (PE/EA: 10/1→5/1) yielded **2h** (94 mg, 82%, *dr* = 1:1) as colourless liquid. ^{1}H NMR (400 MHz, $CDCl_3$) δ 7.38 – 7.28 (m, 5H), 7.25–7.22 (m, 3H), 7.17 – 7.07 (m, 2H), 7.03 – 7.01 (m, 1H), 6.04 (s, 0.5H), 6.00 (s, 0.5H), 5.23 – 5.06 (m, 2H), 4.91 – 4.85 (m, 1H), 3.61 – 3.52 (m, 2H), 3.23 – 3.05 (m, 2H), 1.45 (s, 4.5H), 1.43 (s, 4.5H). ^{13}C NMR (100 MHz, $CDCl_3$) δ 170.6, 170.6, 166.3, 166.2, 153.6, 135.4, 135.3, 135.0, 129.5, 129.4, 128.9, 128.8, 128.7, 127.4, 127.4, 81.9, 76.3, 76.1, 67.7, 67.6, 53.9, 53.9, 37.7, 28.1. HR-MS (ESI) *m/z* calcd for $C_{24}H_{28}N_8KO_5^+$ $[M+K]^+$ 547.1815, found 547.1816.

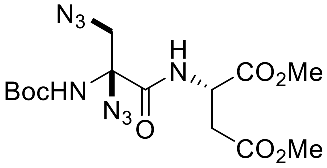

Dimethyl (2,3-diazido-2-((*tert*-butoxycarbonyl)amino)propanoyl)-*L*-aspartate (2i): The general procedure was followed using substrate **1i** (0.20 mmol, 66 mg). Isolation by column chromatography (PE/EA: 10/1→5/1) yielded **2i** (60 mg, 72%, *dr* = 1.2:1) as colourless liquid. ^{1}H NMR (400 MHz, $CDCl_3$) δ 7.66 (d, *J* = 8.5 Hz, 0.5H), 7.57 (d, *J* = 8.5 Hz, 0.4H), 6.01 (s, 0.4H), 5.94 (s, 1=0.5H), 4.81 – 4.75 (m, 1H), 3.77 – 3.76 (m, 4H), 3.70 – 3.68 (m, 4H), 3.09 – 2.86 (m, 2H), 1.43 (s, 3.8H), 1.42 (s, 5.2H). ^{13}C NMR (100 MHz, $CDCl_3$) δ 171.5, 171.3, 170.5, 170.3, 166.5, 166.4, 153.6, 81.8, 76.3, 56.1, 53.2, 53.1, 52.3, 49.4, 49.3, 35.5, 35.4, 28.1. HR-MS (ESI) *m/z* calcd for $C_{14}H_{22}N_8NaO_7^+$ $[M+Na]^+$ 437.1504, found 437.1509.

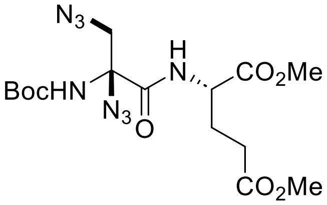

Dimethyl ((*S*)-2,3-diazido-2-((*tert*-butoxycarbonyl)amino)propanoyl)-*L*-glutamate (2j): The general procedure was followed using substrate **1j** (0.20 mmol, 69 mg). Isolation by column chromatography (PE/EA: 10/1→3/1) yielded **2j** (48 mg, 56%, *dr* = 1.6:1) as white oil. ^{1}H NMR (400 MHz, $CDCl_3$) δ 7.39 (dd, J = 24.2, 7.7 Hz, 1H), 5.94 (d, J = 42.3 Hz, 1H), 4.58 (q, J = 7.3, 5.8 Hz, 1H), 3.75 (dd, J = 5.9, 1.1 Hz, 3H), 3.68 (s, 2H), 3.66 (dd, J = 3.8, 1.1 Hz, 3H), 2.52 – 2.36 (m, 2H), 2.32 – 2.20 (m, 1H), 2.06 – 1.96 (m, 1H), 1.43 (s, 9H). ^{13}C NMR (100 MHz, $CDCl_3$) δ 173.5, 171.5, 166.6, 153.6, 81.9, 76.3, 56.2, 52.9, 52.5, 52.0, 29.8, 28.1 270. HR-MS (ESI) *m/z* calcd for $C_{15}H_{24}N_8NaO_7^+$ [M+Na]$^+$ 451.1660, found 451.1660.

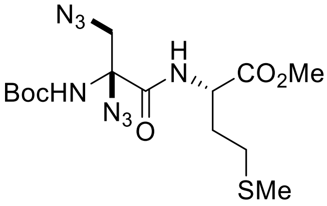

Methyl (2,3-diazido-2-((*tert*-butoxycarbonyl)amino)propanoyl)-*L*-methioninate (2k): The general procedure was followed using substrate **1k** (0.20 mmol, 67 mg). Isolation by column chromatography (PE/EA: 10/1→5/1) yielded **2k** (73 mg, 88%, *dr* = 1.6:1) as colourless liquid. ^{1}H NMR (400 MHz, $CDCl_3$) δ 7.40 – 7.30 (m, 1H), 6.04 (s, 0.4H), 5.94 (s, 0.6H), 4.73 – 4.64 (m, 1H), 3.79 – 3.75 (m, 4H), 3.69 (s, 1H), 2.60 – 2.46 (m, 2H), 2.22 – 2.18 (m, 1H), 2.09 (d, J = 6.0 Hz, 3H), 2.06 – 2.00 (m, 1H), 1.44 (s, 3.4H), 1.43 (s, 5.6H). ^{13}C NMR (100 MHz, $CDCl_3$) δ 171.7, 171.6, 166.5, 166.3, 153.6, 81.9, 76.3, 76.2, 56.2, 52.9, 52.8, 52.4, 52.3, 31.6, 31.2, 29.8, 28.1, 15.5, 15.4. HR-MS (ESI) *m/z* calcd for $C_{14}H_{24}N_8KO_5S^+$ [M+K]$^+$ 455.1222, found 455.1226.

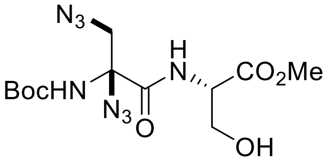

Methyl (2,3-diazido-2-((*tert*-butoxycarbonyl)amino)propanoyl)-*L*-serinate (2l):

The general procedure was followed using substrate **1l** (0.20 mmol, 58 mg). Isolation by column chromatography (PE/EA: 10/1→5/1) yielded **2l** (60 mg, 81%, *dr* = 1.5:1) as colourless liquid. ^{1}H NMR (400 MHz, $CDCl_3$) δ 6.39 (s, 1H), 3.85 (s, 3H), 1.44 (s, 9H). ^{13}C NMR (100 MHz, $CDCl_3$) δ 170.3, 170.2, 166.8, 166.2, 154.2, 154.1, 82.6, 82.2, 76.4, 76.2, 61.9, 61.5, 56.6, 55.8, 55.6, 53.0, 53.0, 28.1, 28.0. HR-MS (ESI) *m/z* calcd for $C_{12}H_{20}N_8NaO_6^+$ $[M+Na]^+$ 395.1399, found 395.1390.

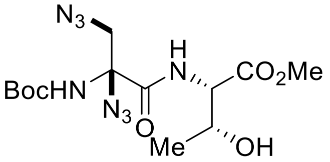

Methyl (2,3-diazido-2-((*tert*-butoxycarbonyl)amino)propanoyl)-*L*-threoninate (2m): The general procedure was followed using substrate **1m** (0.20 mmol, 60 mg). Isolation by column chromatography (PE/EA: 10/1→5/1) yielded **2m** (60 mg, 78%, *dr* = 1:1) as colourless liquid. ^{1}H NMR (400 MHz, $CDCl_3$) δ 7.47 (d, *J* = 9.2 Hz, 0.5H), 7.35 (d, *J* = 9.2 Hz, 0.4H), 6.21 (s, 0.4H), 6.16 (s, 0.5H), 4.53 – 4.48 (m, 1H), 4.37 – 4.34 (m, 0.5H), 4.29 – 4.20 (m, 0.5H), 3.98 – 3.69 (m, 5H),3.31 (d, *J* = 4.8 Hz, 0.5H), 3.08 (d, *J* = 4.8 Hz, 0.4H), 1.43 (s, 4.5H), 1.41 (s, 4.5H), 1.25 (d, *J* = 6.5 Hz, 1.5H), 1.20 (d, *J* = 6.5 Hz, 1.5H). ^{13}C NMR (100 MHz, $CDCl_3$) δ 170.7, 167.4, 166.7, 153.9, 153.8, 82.3, 81.9, 76.5, 76.4, 68.8, 67.8, 58.6, 58.3, 56.2, 53.0, 52.9, 28.1, 28.1, 20.0. HR-MS (ESI) *m/z* calcd for $C_{13}H_{22}N_8NaO_6^+$ $[M+Na]^+$ 409.1555, found 409.1549.

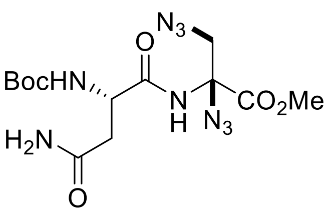

Methyl 2-(4-amino-2-((*tert*-butoxycarbonyl)amino)-4-oxobutanamido)-2,3-diazidopropanoate (2n): The general procedure was followed using substrate **1m** (0.20 mmol, 63 mg). Isolation by column chromatography (DCM/MeOH: 100/1→40/1) yielded **2n** (60 mg, 76%, *dr* = 1.2:1) as brown oil liquid. ^{1}H NMR (400 MHz, DMSO-*d$_6$*) δ 9.53 (s, 0.6H), 9.48 (s, 0.4H), 7.45 (d, *J* = 8.2 Hz, 0.6H), 7.40 (d, *J* = 8.2 Hz, 0.6H), 4.37 – 4.32 (m, 1H), 3.69 (s, 5H), 3.53 (t, *J* = 12.8 Hz, 1H), 2.89 – 2.67 (m, 3H), 1.38 (s, 9H). ^{13}C NMR (100 MHz, DMSO-*d$_6$*) δ 171.1, 170.9, 167.2, 167.1, 155.6, 118.2,

79.5, 79.5, 76.2, 75.9, 55.1, 55.0, 53.9, 53.9, 50.7, 28.6. HR-MS (ESI) *m/z* calcd for $C_{13}H_{21}N_9NaO_6^+$ [M+Na]$^+$ 422.1507, found 422.1496.

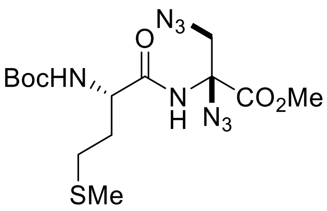

Methyl 2,3-diazido-2-((*S*)-2-((*tert*-butoxycarbonyl)amino)-4-(methylthio)-butan amido)propanoate (2o): The general procedure was followed using substrate **1o** (0.20 mmol, 67 mg). Isolation by column chromatography (PE/EA: 10/1→5/1) yielded **2o** (61 mg, 73%, *dr* = 1:1) as colourless liquid. ^{1}H NMR (400 MHz, DMSO-*d*$_6$) δ 9.42 (s, 0.43H), 9.32 (s, 0.46H), 7.10 (d, J = 8.0 Hz, 0.42H), 7.02 (d, J = 8.0 Hz, 0.37H), 4.02 – 3.97 (m, 1H), 3.68 – 3.60 (m, 5H), 3.51 – 3.41 (m, 1H), 2.43 – 2.37 (m, 1H), 1.99 (s, 1.5H), 1.99 (s, 1.5H), 1.81 – 1.69 (m, 2H), 1.32 (s, 4.5H), 1.32 (s, 4.5H). ^{13}C NMR (100 MHz, DMSO-*d*$_6$) δ 174.0, 173.8, 167.5, 156.0, 155.9, 78.8, 76.1, 76.0, 55.2, 55.0, 53.8, 53.7, 53.7, 32.1, 31.5, 30.1, 30.0, 28.7, 15.2. HR-MS (ESI) *m/z* calcd for $C_{14}H_{24}N_8NaO_5S^+$ [M+Na]$^+$ 439.1483, found 439.1477.

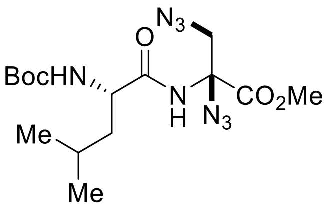

Methyl 2,3-diazido-2-((*S*)-2-((*tert*-butoxycarbonyl)amino)-4-methyl-pentanamido) propanoate (2p): The general procedure was followed using substrate **1p** (0.20 mmol, 63 mg). Isolation by column chromatography (PE/EA: 10/1→5/1) yielded **2p** (79 mg, 99%, *dr* = 1.8:1) as colourless liquid. ^{1}H NMR (400 MHz, DMSO-*d*$_6$) δ 9.29 (s, 0.29H), 9.29 (s, 0.53H), 7.01 (d, J = 8.0 Hz, 0.29H), 6.95 (d, J = 8.0 Hz, 0.46H), 4.08 – 3.96 (m, 1H), 3.72 – 3.64 (m, 4H), 3.54 – 3.43 (m, 1H), 1.68 – 1.53 (m, 1H), 1.37 (s, 5.8H), 1.36 (s, 3.3H), 1.31 – 1.20 (m, 2H), 0.89 – 0.83 (m, 6H). ^{13}C NMR (100 MHz, DMSO-*d*$_6$) δ 174.7, 174.6, 167.4, 156.0, 155.9, 78.7, 76.1, 76.0, 55.2, 55.1, 53.7, 53.7, 52.8, 41.2, 28.7, 28.7, 24.8, 24.6, 23.5, 23.4, 22.1, 22.0. HR-MS (ESI) *m/z* calcd for $C_{15}H_{26}N_8NaO_5^+$ [M+Na]$^+$421.1924, found 421.1920.

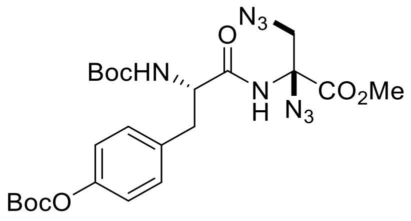

Methyl 2,3-diazido-2-((*S*)-2-((*tert*-butoxycarbonyl)amino)-3-(4-((*tert*-Butoxycarbonyl)oxy)phenyl)propanamido)propanoate (2q): The general procedure was followed using substrate **1q** (0.20 mmol, 73 mg). Isolation by column chromatography (PE/EA: 10/1→5/1) yielded **2q** (103 mg, 94%, *dr* = 1:1) as brown liquid. ^{1}H NMR (400 MHz, DMSO-d_6) δ 9.47 (s, 0.5H), 9.42 (s, 0.4H), 7.31 (d, J = 8.1 Hz, 2H), 7.15 – 7.01 (m, 3H), 4.24 – 4.19 (m, 1H), 3.74 – 3.63 (m, 4H), 3.58 – 3.45 (m, 1H), 2.96 – 2.63 (m, 2H), 1.47 (s, 9H), 1.30 (s, 9H). ^{13}C NMR (100 MHz, DMSO-d_6) δ 173.8, 173.6, 167.4, 156.0, 155.9, 151.9, 151.9, 149.8, 135.9, 130.8, 121.5, 83.6, 78.8, 78.8, 76.2, 76.0, 56.0, 55.9, 55.2, 55.1, 53.8, 53.7, 37.2, 36.5, 28.6, 28.6, 27.8. HR-MS (ESI) *m/z* calcd for $C_{23}H_{32}N_8NaO_8^+$ [M+Na]$^+$ 571.2236, found 571.2240.

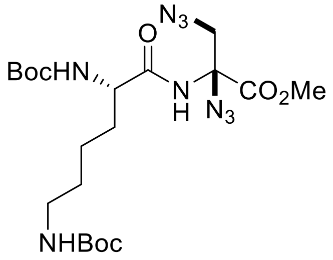

Methyl 2,3-diazido-2-((*S*)-2,6-bis((*tert*-butoxycarbonyl)amino)hexanamido)-propanoate (2r): The general procedure was followed using substrate **1r** (0.20 mmol, 86 mg). Isolation by column chromatography (PE/EA: 10/1→3/1) yielded **2r** (74 mg, 72%, *dr* = 1:1) as colourless oil liquid. ^{1}H NMR (400 MHz, DMSO-d_6) δ 9.36 (s, 0.48H), 9.25 (s, 0.48H), 6.94 (d, J = 8.0 Hz, 0.5H), 6.94 (d, J = 8.0 Hz, 0.4H), 6.74 (t, J = 5.6 Hz, 1H), 3.98 – 3.86 (m, 1H), 3.70 – 3.66 (m, 4H), 3.54 – 3.44 (m, 1H), 2.88– 2.85 (m, 2H), 1.55 – 1.43 (m, 3H), 1.36 (s, 18H), 1.27 – 1.22 (m, 3H). ^{13}C NMR (100 MHz, DMSO-d_6) δ 174.3, 174.2, 172.6, 167.4, 167.4, 156.1, 156.0, 155.9, 78.7, 77.9, 76.1, 76.0, 60.3, 55.2, 55.1, 54.4, 53.7, 53.7, 32.1, 31.5, 31.4, 29.7, 28.8, 28.7, 28.2, 23.3, 23.2, 22.6, 21.6, 21.3, 14.6, 14.5. HR-MS (ESI) *m/z* calcd for $C_{20}H_{35}N_9NaO_7^+$ [M+Na]$^+$ 536.2552, found 536.2553.

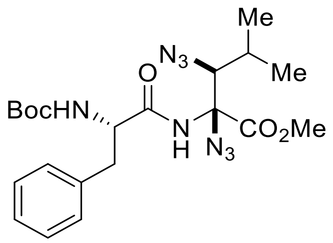

Methyl 2,3-diazido-2-((*S*)-2-((*tert*-butoxycarbonyl)amino)-3-phenylpropanamido)-4-methylpentanoate (2s): The general procedure was followed using substrate **1s** (0.20 mmol, 86 mg). Isolation by column chromatography (PE/EA: 10/1→5/1) yielded **2s** (63 mg, 66%, *dr* = 1:1) as colourless oil liquid. ^{1}H NMR (400 MHz, DMSO-d_6) δ 9.29 (d, J = 17.4 Hz, 0.6H), 9.03 (d, J = 17.4 Hz, 0.4H), 7.32 – 7.27 (m, 4H), 7.22 – 7.09 (m, 2H), 4.53 – 4.23 (m, 1H), 3.77 – 3.59 (m, 5H), 2.93 – 2.70 (m, 2H), 1.31 – 1.29 (m, 9H), 1.02 – 0.93 (m, 6H). ^{13}C NMR (100 MHz, DMSO-d_6) δ 174.2, 174.0, 173.9, 167.8, 167.4, 167.2, 167.1, 156.1, 156.1, 156.0, 138.3, 138.2, 138.1, 129.9, 129.8, 128.6, 128.6, 126.9, 126.9, 78.8, 78.8, 78.6, 78.5, 78.2, 70.4, 70.3, 70.2, 69.7, 60.3, 55.8, 55.7, 55.7, 55.6, 53.7, 53.6, 38.1, 37.8, 37.0, 30.6, 29.2, 29.1, 28.6, 28.3, 22.5, 22.2, 21.9, 18.2, 17.7, 17.5, 17.3, 14.6. HR-MS (ESI) *m/z* calcd for $C_{21}H_{30}N_8NaO_5^+$ [M+Na]$^+$ 497.2232, found 497.2232.

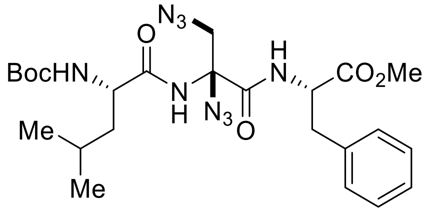

Methyl (2,3-diazido-2-((*S*)-2-((*tert*-butoxycarbonyl)amino)-4-methyl-pentanamido)propanoyl)-*L*-phenylalaninate (2t): The general procedure was followed using substrate **1t** (0.20 mmol, 92 mg). Isolation by column chromatography (PE/EA: 10/1→3/1) yielded **2t** (87 mg, 80%, *dr* = 1:1) as colourless oil liquid. ^{1}H NMR (400 MHz, DMSO-d_6) δ 8.88 (s, 0.4H), 8.70 (s, 0.4H), 8.33 (d, J = 8.0 Hz, 0.4H), 8.19 (d, J = 8.0 Hz, 0.4H), 7.24 – 7.14 (m, 6H), 7.06 (t, J = 8.0 Hz, 1H), 4.51 – 4.39 (m, 1H), 4.06 – 3.97 (m, 1H), 3.61 (d, J = 7.8 Hz, 1H), 3.56 – 3.53 (d, 4H), 3.03 – 2.95 (m, 2H), 1.61 – 1.52 (m, 1H), 1.32 (s, 9H), 1.21 – 1.17 (m, 1H), 0.82 – 0.79 (m, 6H). ^{13}C NMR (100 MHz, DMSO-d_6) δ 74.3, 174.0, 171.7, 171.5, 166.4, 166.3, 156.1, 155.9, 137.6, 137.5, 129.7, 129.7, 128.8, 127.2, 127.1, 78.8, 78.7, 76.4, 76.0, 54.9, 54.9, 54.6, 53.2, 53.0,

52.6, 52.5, 41.2, 37.0, 36.9, 28.7, 24.8, 24.7, 23.7, 23.6, 21.9, 21.8. HR-MS (ESI) *m/z* calcd for $C_{24}H_{35}N_9NaO_6^+$ $[M+Na]^+$ 568.2603, found 568.2597.

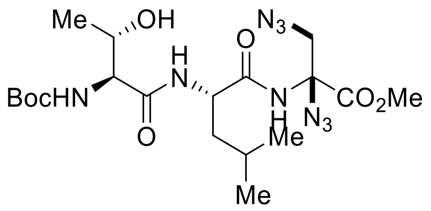

Methyl (6*S*,9*S*)-12-azido-12-(azidomethyl)-6-((*S*)-1-hydroxyethyl)-9-isobutyl-2,2-dimethyl-4,7,10-trioxo-3-oxa-5,8,11-triazatridecan-13-oate (2u): The general procedure was followed using substrate **1u** (0.20 mmol, 83 mg). Isolation by column chromatography (PE/EA: 10/1→1/1) yielded **2u** (65 mg, 65%) as colourless oil liquid. ^{1}H NMR (400 MHz, DMSO-*d*$_6$) δ 9.40 (s, 0.5H), 9.35 (s, 0.3H), 7.98 – 7.94 (m, 1H), 6.44 (d, J = 8.0 Hz, 1H), 4.81 (d, J = 5.2 Hz, 0.4H), 4.77 (d, J = 5.2 Hz, 0.5H), 4.44 – 4.38 (m, 1H), 3.88 – 3.78 (m, 2H), 3.67 (s, 1.7H), 3.65 (s, 1.4H), 3.50 – 3.40 (m, 1H), 1.66 – 1.57 (m, 1H), 1.36 (s, 9H), 1.27 – 1.21 (m, 3H), 1.00 – 0.99 (m, 3H), 0.87 – 0.80 (m, 6H). ^{13}C NMR (100 MHz, DMSO-*d*$_6$) δ 173.9, 173.9, 170.9, 167.3, 155.9, 78.8, 76.1, 75.9, 67.4, 60.7, 60.6, 55.2, 55.1, 53.8, 53.8, 50.9, 50.8, 31.5, 28.7, 26.9, 24.5, 24.4, 23.6, 23.5, 22.7, 22.1, 22.0, 20.2, 14.5. HR-MS (ESI) *m/z* calcd for $C_{19}H_{33}N_9NaO_7^+$ $[M+Na]^+$ 522.2396, found 522.2385.

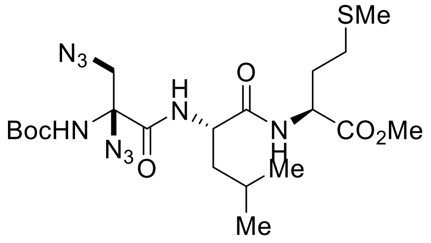

Methyl (2,3-diazido-2-((*tert*-butoxycarbonyl)amino)propanoyl)-*L*-leucyl-*L*-methioninate (2v): The general procedure was followed using substrate **1v** (0.20 mmol, 89 mg). Isolation by column chromatography (PE/EA: 10/1→3/1) yielded **2v** (81 mg, 76%, *dr* = 1.2:1) as oil liquid. ^{1}H NMR (400 MHz, $CDCl_3$) δ 7.58 – 6.95 (m, 2H), 6.42 (d, J = 8.8 Hz, 0.5H), 6.24 (s, 0.3H), 4.66 – 4.22 (m, 2H), 3.79 – 3.61 (m, 5H), 2.76 – 2.37 (m, 2H), 2.20 – 1.87 (m, 5H), 1.78 – 1.45 (m, 3H), 1.43 – 1.40 (m, 9H), 0.94 – 0.87 (m, 6H). ^{13}C NMR (100 MHz, $CDCl_3$) δ 172.2, 172.1, 172.0, 171.9, 171.4, 171.3, 166.8, 166.3, 154.2, 153.8, 82.3, 81.9, 76.6, 76.4, 64.9, 56.4, 52.9, 52.7, 52.5, 52.3, 52.0,

51.6, 40.7, 40.0, 31.4, 30.8, 30.6, 30.0, 28.1, 28.1, 25.0, 24.7, 23.3, 23.1, 21.8, 21.3, 15.5, 15.5. HR-MS (ESI) *m/z* calcd for $C_{20}H_{35}N_{9}NaO_{6}^{+}$ $[M+Na]^{+}$552.2324, found 552.2324.

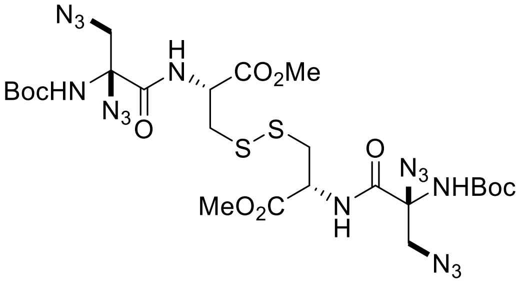

Methyl (9*R*,14*R*)-6-azido-6-(azidomethyl)-14-(2,3-diazido-2-((*tert*-butoxycarbonyl) amino)propanamido)-9-(methoxycarbonyl)-2,2-dimethyl-4,7-dioxo-3-oxa-11,12-dithia-5,8-diazapentadecan-15-oate (2w): The general procedure was followed using substrate **1w** (0.20 mmol, 121 mg). Isolation by column chromatography (PE/EA: 10/1→2/1) yielded **2w** (126 mg, 80%, *dr* = 1.4:1) as brown solid. ^{1}H NMR (400 MHz, $CDCl_3$) δ 7.54 – 7.48 (m, 2H), 6.07 – 6.05 (m, 2H), 4.86 – 4.78 (m, 2H), 3.78 – 3.76 (d, *J* = 6.9 Hz, 8H), 3.73 – 3.68 (m, 2H), 3.27 – 3.16 (m, 4H), 1.44 (s, 10.6H), 1.43 (s, 7.4H). ^{13}C NMR (100 MHz, $CDCl_3$) δ 170.1, 170.0, 166.7, 166.6, 153.7, 153.6, 81.9, 76.3, 76.2, 55.7, 53.1, 53.1, 53.1, 53.0, 52.5, 52.4, 40.1, 39.9, 39.8, 39.7, 28.1. HR-MS (ESI) *m/z* calcd for $C_{24}H_{38}N_{16}NaO_{10}S_{2}^{+}$ $[M+Na]^{+}$ 797.2290, found 797.2280.

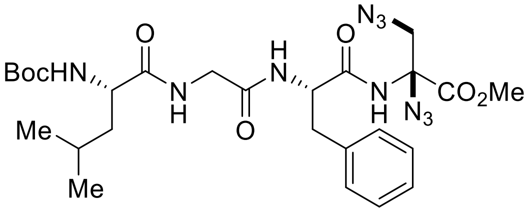

Methyl (6*S*,12*S*)-15-azido-15-(azidomethyl)-12-benzyl-6-isobutyl-2,2-dimethyl-4,7,10,13-tetraoxo-3-oxa-5,8,11,14-tetraazahexadecan-16-oate (2x): The general procedure was followed using substrate **1x** (0.20 mmol, 104 mg). Isolation by column chromatography (DCM/MeOH: 100/1→50/1) yielded **2x** (112 mg, 93%, *dr* = 1:1) as brown solid. ^{1}H NMR (400 MHz, DMSO-d_6) δ 9.43 (d, *J* = 17.4 Hz, 1H), 8.14 (t, *J* = 7.8 Hz, 1H), 7.99 (d, *J* = 5.8 Hz, 1H), 7.28 – 7.16 (m, 5H), 6.90 (d, *J* = 7.5 Hz, 1H), 4.61 – 4.51 (m, 1H), 3.94 (q, *J* = 7.0 Hz, 1H), 3.76 – 3.66 (m, 4H), 3.66 – 3.54 (m, 2H), 3.50 (d, *J* = 12.8 Hz, 0.53H),3.46 (d, *J* = 12.8 Hz, 0.48H), 3.06 – 2.86 (m, 1H), 2.86 –

2.64 (m, 1H), 1.61 – 1.33 (m, 12H), 0.86 – 0.81 (m, 6H).^{13}C NMR (100 MHz, DMSO-D_6) δ 173.6, 172.9, 172.7, 169.2, 167.4, 167.3, 156.0, 137.8, 137.6, 129.8, 128.8, 128.7, 127.1, 127.0, 78.6, 76.1, 76.0, 55.3, 55.2, 53.9, 53.3, 42.5, 41.4, 38.4, 37.8, 31.6, 28.8, 24.8, 23.6, 22.0, 14.6. HR-MS (ESI) *m/z* calcd for $C_{26}H_{38}N_{10}NaO_7^+$ $[M+Na]^+$ 625.2818, found 625.2816.

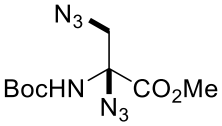

Methyl 2,3-diazido-2-((*tert*-butoxycarbonyl)amino)propanoate (2y): The general procedure was followed using substrate **1y** (0.20 mmol, 40 mg). Isolation by column chromatography (PE/EA: 20/1→10/1) yielded **2y** (49 mg, 86%) as colourless oil liquid. ^{1}H NMR (400 MHz, $CDCl_3$) δ 6.01 (s, 1H), 4.11 (s, 1H), 3.87 (s, 3H), 3.45 (d, J = 12.6 Hz, 1H), 1.45 (s, 9H). ^{13}C NMR (100 MHz, $CDCl_3$) δ 167.3, 153.6, 82.0, 76.0, 54.1, 53.4, 28.1. HR-MS (ESI) *m/z* calcd for $C_9H_{15}N_7NaO_4^+$ $[M+Na]^+$ 308.1078, found 308.1075.

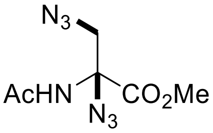

Methyl 2-acetamido-2,3-diazidopropanoate (2z): The general procedure was followed using substrate **1z** (0.20 mmol, 29 mg). Isolation by column chromatography (PE/EA: 10/1→2/1) yielded **2z** (39 mg, 86%) as colourless oil liquid. ^{1}H NMR (400 MHz, $CDCl_3$) δ 6.84 (s, 1H), 4.05 (d, J = 12.6 Hz, 1H), 3.88 (s, 3H), 3.50 (d, J = 12.6 Hz, 1H), 2.11 (s, 3H). ^{13}C NMR (100 MHz, $CDCl_3$) δ 170.6, 167.3, 75.4, 54.2, 53.6, 23.2. HR-MS (ESI) *m/z* calcd for $C_6H_9N_7NaO_3^+$ $[M+Na]^+$ 250.0660, found 250.0659.

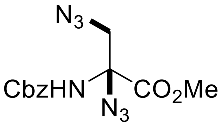

Methyl 2,3-diazido-2-(((benzyloxy)carbonyl)amino)propanoate (2z'): The general procedure was followed using substrate **1z'** (0.20 mmol, 47 mg). Isolation by column chromatography (PE/EA: 20/1→7/1) yielded **2z'** (39 mg, 84%) as colourless oil liquid. ^{1}H NMR (400 MHz, $CDCl_3$) δ 7.40 – 7.31 (m, 5H), 6.25 (s, 1H), 5.20 – 5.10 (m, 2H),

4.13 (s, 1H), 3.87 (s, 3H), 3.53 (d, *J* = 12.5 Hz, 1H). ^{13}C NMR (100 MHz, $CDCl_3$) δ 167.1, 154.4, 135.4, 128.8, 128.7, 128.5, 76.0, 67.9, 54.3, 53.4. HR-MS (ESI) *m/z* calcd for $C_{12}H_{13}N_7NaO_4^+$ $[M+Na]^+$ 342.0922, found 342.0919.

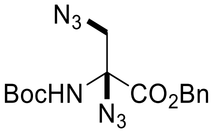

Benzyl 2,3-diazido-2-((*tert*-butoxycarbonyl)amino)propanoate (2aa): The general procedure was followed using substrate **1aa** (0.20 mmol, 56 mg). Isolation by column chromatography (PE/EA: 20/1→7/1) yielded **2aa** (62 mg, 86%) as colourless oil liquid. ^1H NMR (400 MHz, $CDCl_3$) δ 7.37 (s, 5H), 5.97 (s, 1H), 5.28 (q, *J* = 12.1 Hz, 2H), 4.11 (s, 1H), 3.46 (d, *J* = 12.6 Hz, 1H), 1.45 (s, 9H). ^{13}C NMR (100 MHz, $CDCl_3$) δ 166.7, 153.6, 134.4, 129.0, 128.8, 128.6, 82.0, 76.1, 69.2, 53.5, 28.1. HR-MS (ESI) *m/z* calcd for $C_{15}H_{19}N_7NaO_4^+$ $[M+Na]^+$ 384.1391, found 384.1388.

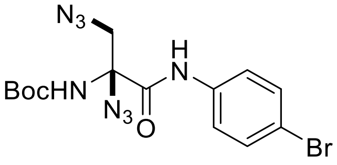

***Tert*-butyl (2,3-diazido-1-((4-bromophenyl)amino)-1-oxopropan-2-yl)carbamate (2ab):** The general procedure was followed using substrate **1ab** (0.20 mmol, 68 mg). Isolation by column chromatography (PE/EA: 20/1→7/1) yielded **2ab** (55 mg, 65%) as faint yellow oil liquid. ^1H NMR (400 MHz, $CDCl_3$) δ 8.56 (s, 1H), 7.44 (s, 4H), 6.10 (s, 1H), 3.87 – 3.67 (m, 2H), 1.42 (s, 9H). ^{13}C NMR (100 MHz, $CDCl_3$) δ 164.6, 153.7, 135.9, 132.2, 121.8, 118.1, 76.7, 60.6, 56.4, 28.1. HR-MS (ESI) *m/z* calcd for $C_{14}H_{17}BrN_8NaO_3^+$ $[M+Na]^+$ 447.0500, found 447.0498.

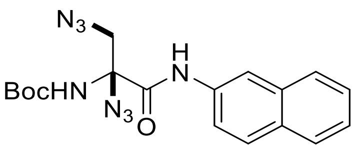

***Tert*-butyl (2,3-diazido-1-(naphthalen-2-ylamino)-1-oxopropan-2-yl)carbamate (2ac):** The general procedure was followed using substrate **1ac** (0.20 mmol, 62 mg). Isolation by column chromatography (PE/EA: 20/1→7/1) yielded **2ac** (73 mg, 92%) as faint yellow oil liquid. ^1H NMR (400 MHz, $CDCl_3$) δ 8.70 (s, 1H), 8.23 (s, 1H), 7.82 – 7.77 (m, 3H), 7.51 (dd, *J* = 8.8, 2.1 Hz, 1H), 7.49 – 7.41 (m, 2H), 6.15 (s, 1H), 3.85 (dd,

J = 34.7, 12.9 Hz, 2H), 1.45 (s, 9H). ^{13}C NMR (100 MHz, $CDCl_3$) δ 164.6, 153.7, 134.2, 133.8, 131.2, 129.1, 127.9, 127.7, 126.8, 125.6, 119.9, 117.4, 82.3, 56.4, 28.1. HR-MS (ESI) *m/z* calcd for $C_{18}H_{20}N_8NaO_3^+$ $[M+Na]^+$ 419.1551, found 419.1548.

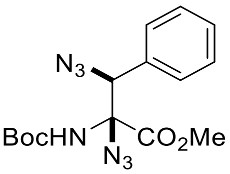

Methyl 2,3-diazido-2-((*tert*-butoxycarbonyl)amino)-3-phenylpropanoate (2ad): The general procedure was followed using substrate **1ad** (0.20 mmol, 55 mg). Isolation by column chromatography (PE/EA: 20/1→10/1) yielded **2ad** (71 mg, 98%) as colourless oil liquid. ^1H NMR (400 MHz, $CDCl_3$) δ 7.47 (s, 1.8H), 7.38 (s, 3.2H), 5.99 (s, 0.6H), 4.87 (s, 0.3H), 3.88 (s, 1.0H), 3.65 (s, 2.0H), 1.45 (s, 6.0H), 1.40 (s, 3.0H). ^{13}C NMR (100 MHz, $CDCl_3$) δ 167.5, 166.5, 154.1, 153.8, 131.8, 130.4, 130.0, 129.4, 129.3, 129.0, 128.9, 82.2, 81.9, 78.7, 77.9, 69.4, 69.2, 53.9, 53.5, 28.0, 28.0. HR-MS (ESI) *m/z* calcd for $C_{15}H_{19}N_7NaO_4^+$ $[M+Na]^+$ 384.1391, found 384.1384.

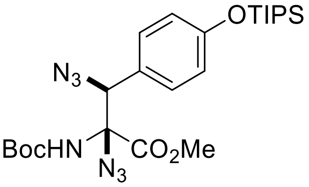

Methyl 2,3-diazido-2-((*tert*-butoxycarbonyl)amino)-3-(4-((triisopropylsilyl)oxy)-phenyl)propanoate (2ae): The general procedure was followed using substrate **1ae** (0.20 mmol, 55 mg). Isolation by column chromatography (PE/EA: 30/1→15/1) yielded **2ae** (60 mg, 57%) as colourless oil liquid. ^1H NMR (400 MHz, $CDCl_3$) δ 7.36 (d, J = 8.3 Hz, 0.6H), 7.26 (d, J = 8.3 Hz, 1.4H), 6.96 (d, J = 8.1 Hz, 0.6H), 6.88 (d, J = 8.2 Hz, 1.3H), 5.96 (s, 0.5H), 4.77 (s, 0.3H), 1.12-1.08 (s, 1.0H), 3.61 (s, 2.0H), 1.46 (s, 6.0H), 1.41 (s, 3.1H), 1.28 – 1.24 (m, 3.1H), 1.09 (m, 18.1H). ^{13}C NMR (100 MHz, $CDCl_3$) δ 167.7, 166.6, 158.0, 157.6, 130.7, 130.4, 124.0, 120.8, 120.3, 78.8, 69.1, 68.9, 53.8, 53.4, 28.1, 28.0, 18.0, 17.9, 12.7, 12.7. HR-MS (ESI) *m/z* calcd for $C_{24}H_{39}N_7NaO_5Si^+$ $[M+Na]^+$ 556.2675, found 556.2670.

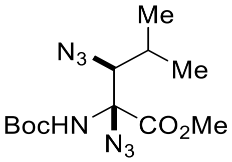

Methyl 2,3-diazido-2-((*tert*-butoxycarbonyl)amino)-4-methylpentanoate (2af): The general procedure was followed using substrate **1af** (0.20 mmol, 49 mg). Isolation by column chromatography (PE/EA: 50/1→20/1) yielded **2af** (53 mg, 81%) as colourless oil liquid. ^{1}H NMR (400 MHz, $CDCl_3$) δ 5.96 (s, 0.5H), 5.56 (s, 0.3H), 3.87 (s, 1.2H), 3.86 (s, 1.8H), 3.54 (d, J = 5.4 Hz, 0.3H), 3.38 (d, J = 5.4 Hz, 0.5H), 2.21-2.03 (m, 0.6H), 1.99-1.90 (m, 0.4H), 1.46 (s, 9.1H), 1.09 (d, J = 2.1 Hz, 1.7H), 1.07 (d, J = 2.1 Hz, 1.2H), 1.01 (d, J = 1.9 Hz, 1.7H), 0.99 (s, 1.2H). ^{13}C NMR (100 MHz, $CDCl_3$) δ 167.9, 167.2, 154.0, 153.8, 81.9, 81.9, 79.7, 78.4, 72.3, 71.2, 53.7, 53.6, 29.9, 29.8, 28.1, 28.1, 22.3, 21.1, 18.7, 16.9. HR-MS (ESI) *m/z* calcd for $C_{12}H_{21}N_7NaO_4^+$ $[M+Na]^+$ 350.1548, found 350.1542.

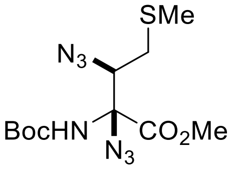

Methyl 2,3-diazido-2-((*tert*-butoxycarbonyl)amino)-4-(methylthio)butanoate (2ag): The general procedure was followed using substrate **1ag** (0.20 mmol, 52 mg). Isolation by column chromatography (PE/EA: 50/1→20/1) yielded **2ag** (54 mg, 94%) as colourless oil liquid. ^{1}H NMR (400 MHz, $CDCl_3$) δ 6.29 (s, 0.4H), 5.98 (s, 0.4H), 3.90 – 3.87 (m, 4.0H), 2.98 (dd, J = 14.4, 4.6 Hz, 0.6H), 2.78 (dd, J = 14.4, 4.6 Hz, 0.5H), 2.64 (dd, J = 6.1, 2.6 Hz, 0.5H), 2.61 (dd, J = 6.1, 2.6 Hz, 0.4H), 2.20 (s, 1.6H), 2.12 (s, 1.4H), 1.45 (s, 4.5H), 1.45 (s, 4.7H). ^{13}C NMR (100 MHz, $CDCl_3$) δ 167.1, 167.0, 154.1, 153.9, 82.4, 82.2, 79.1, 78.7, 64.7, 64.6, 53.9, 53.9, 34.0, 33.7, 28.1, 28.0, 16.3, 16.0. HR-MS (ESI) *m/z* calcd for $C_{11}H_{19}N_7NaO_4S^+$ $[M+Na]^+$ 368.1112, found 368.1110.

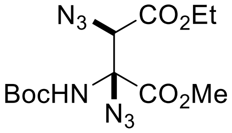

4-Ethyl 1-methyl 2,3-diazido-2-((*tert*-butoxycarbonyl)amino)succinate (2ah): The

general procedure was followed using substrate **1ah** (0.20 mmol, 55 mg). Isolation by column chromatography (PE/EA: 30/1→15/1) yielded **2ah** (60 mg, 84%, *dr* = 1:1) as colourless oil liquid. ^{1}H NMR (400 MHz, $CDCl_3$) δ 6.73 (s, 0.4H), 6.06 (s, 0.4H), 4.41 – 4.33 (m, 1.1H), 4.32 – 4.23 (m, 1.0H), 4.14 (s, 0.4H), 4.00 (s, 0.5H),3.90 (s, 1.5H), 3.89 (s, 1.4H), 1.45 (s, 4.5H), 1.44 (s, 4.6H), 1.38 (d, J = 7.1 Hz, 1.5H), 1.31 (t, J = 7.2 Hz, 1.6H). ^{13}C NMR (100 MHz, $CDCl_3$) δ 166.4, 166.2, 165.3, 154.0, 153.8, 82.1, 78.1, 65.3, 64.5, 63.7, 63.2, 54.0, 53.9, 28.0, 14.1, 14.0. HR-MS (ESI) *m/z* calcd for $C_{12}H_{19}N_7NaO_6^+$ $[M+Na]^+$ 380.1290, found 380.1290.

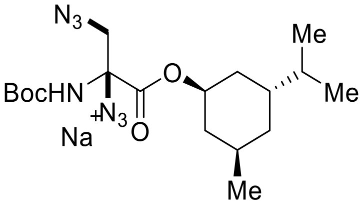

(1*R*,3*R*,5*R*)-3-*Iso*propyl-5-methylcyclohexyl 2,3-diazido-2-((*tert*-butoxycarbonyl) -amino)propanoate (2ai): The general procedure was followed using substrate **1ai**(0.20 mmol, 65 mg). Isolation by column chromatography (PE/EA: 50/1→20/1) yielded **2ai** (63 mg, 76%, *dr* = 1:1) as colourless liquid. ^{1}H NMR (400 MHz, $CDCl_3$) δ 6.02 (s, 1H), 4.87 – 4.77 (m, 1H), 4.26 (t, J = 14.1 Hz, 1H), 3.47 (d, J = 3.8 Hz, 0.5H), 3.44 (d, J = 3.8 Hz, 0.5H), 2.07 – 2.01 (m, 1H), 1.92 – 1.83 (m, 1H), 1.73 – 1.68 (m, 2H), 1.47 (s, 9H), 1.26 (d, J = 12.1 Hz, 2H), 1.09 – 0.99 (m, 2H), 0.93 – 0.88 (m, 7H), 0.78 – 0.75 (m, 3H). ^{13}C NMR (100 MHz, $CDCl_3$) δ 166.4, 166.4, 153.6, 153.5, 81.8, 78.5, 78.4, 76.0, 75.9, 52.9, 47.0, 46.9, 40.4, 34.1, 31.5, 29.8, 28.2, 26.2, 25.9, 23.2, 23.0, 22.0, 22.0, 20.9, 20.9, 16.0, 15.8. HR-MS (ESI) *m/z* calcd for $C_{18}H_{31}N_7NaO_4^+$ $[M+Na]^+$ 432.2335, found 432.2331.

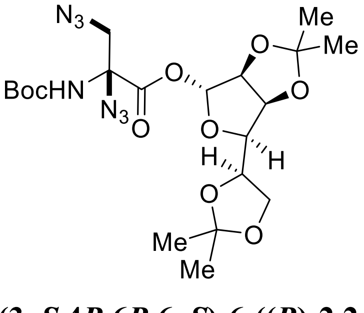

(3a*S*,4*R*,6*R*,6a*S*)-6-((*R*)-2,2-Dimethyl-1,3-dioxolan-4-yl)-2,2-dimethyltetrahydro-furo[3,4-d][1,3]dioxol-4-yl 2,3-diazido-2-((*tert*-butoxycarbonyl)amino)-propano

ate (2aj): The general procedure was followed using substrate **1aj** (0.20 mmol, 86 mg). Isolation by column chromatography (PE/EA: 50/1→10/1) yielded **2aj** (72 mg, 70%, *dr* = 1.5:1) as white solid. ^{1}H NMR (400 MHz, DMSO-d_6) δ 8.68 (s, 0.3H), 8.62 (s, 0.4H), 5.97 – 5.94 (m, 1H), 4.85 – 4.69 (m, 2H), 4.28 – 4.23 (m, 1H), 4.03 – 3.95 (m, Hz, 2H), 3.84 – 3.75 (m, 1H), 3.65 (s, 0.4H), 3.62 (s, 0.6H), 3.54 – 3.46 (m, 1H), 1.39 (s, 3.6H), 1.38 (s, 5.4H), 1.37 – 11.35 (m, 2H), 1.33 – 1.21 (m, 12H). ^{13}C NMR (100 MHz, DMSO-d_6) δ 165.9, 165.8, 155.2, 155.0, 112.9, 112.8, 108.9, 103.3, 103.1, 84.8, 84.6, 83.1, 83.0, 81.0, 79.2, 79.1, 77.3, 77.2, 72.5, 66.5, 55.3, 55.1, 28.8, 28.4, 28.2, 27.0, 26.3, 25.6, 25.5, 24.8. HR-MS (ESI) *m/z* calcd for $C_{21}H_{33}N_7NaO_9^+$ [M+Na]$^+$ 536.2076, found 536.2077.

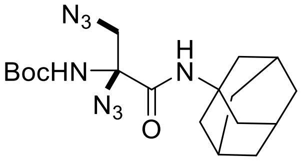

***Tert*-butyl (1-(adamantan-1-ylamino)-2,3-diazido-1-oxopropan-2-yl)carbamate (2ak):** The general procedure was followed using substrate **1ak** (0.20 mmol, 64 mg). Isolation by column chromatography (PE/EA: 50/1→10/1) yielded **2ak** (58 mg, 72%) as yellow solid. ^{1}H NMR (400 MHz, CDCl$_3$) δ 6.34 (s, 1H), 6.02 (s, 1H), 3.74 (d, J = 5.6 Hz, 2H), 2.10 – 2.06 (m, 3H), 2.00 (s, 6H), 1.67 (t, J = 3.1 Hz, 6H), 1.45 (s, 9H). ^{13}C NMR (100 MHz, CDCl$_3$) δ 165.0, 153.8, 81.5, 76.2, 55.3, 52.8, 41.1, 36.3, 29.4, 28.2. HR-MS (ESI) *m/z* calcd for $C_{18}H_{28}N_8NaO_3^+$ [M+Na]$^+$ 427.2177, found 427.2175.

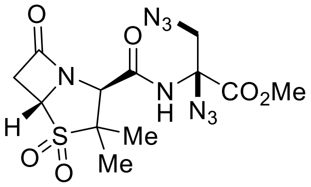

Methyl 2,3-diazido-2-((2*S*,5*R*)-3,3-dimethyl-4,4-dioxido-7-oxo-4-thia-1-azabicyclo [3.2.0]heptane-2-carboxamido)propanoate (2al): The general procedure was followed using substrate **1al** (0.20 mmol, 63 mg). Isolation by column chromatography (DCM/MeOH: 100/1→50/1) yielded **2al** (74 mg,90%, *dr* = 1.5:1) as white solid. ^{1}H NMR (400 MHz, CDCl$_3$) δ 7.72 (s, 0.4H), 7.70 (s, 0.6H), 4.70 – 4.67 (m, 1H), 4.27 (s, 0.4H), 4.25 (s, 0.6H), 3.96 (t, J = 12.1 Hz, 1H), 3.89 (s, 3H), 3.67 – 3.61 (m, 1H), 3.58 – 3.51 (m, 2H), 1.64 (s, 3H), 1.49 (s, 2H), 1.45 (s, 1H). ^{13}C NMR (100 MHz, CDCl$_3$) δ

172.5, 172.4, 167.4, 167.2, 166.7, 166.6, 75.3, 75.1, 63.8, 63.7, 63.6, 63.5, 60.7, 54.4, 54.4, 53.8, 53.7, 38.5, 19.6, 19.5, 18.3. HR-MS (ESI) *m/z* calcd for $C_{12}H_{16}N_8NaO_6^+$ $[M+Na]^+$ 423.0806, found 423.0798.

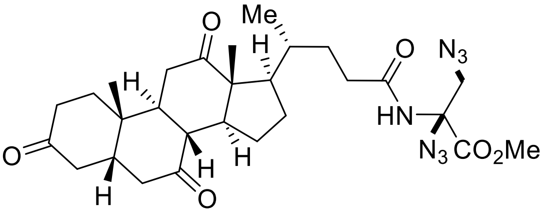

Methyl 2,3-diazido-2-((*R*)-4-((5*S*,8*R*,9*S*,10*S*,13*R*,14*S*,17*R*)-10,13-dimethyl-3,7,12-trioxohexadecahydro-1H-cyclopenta[a]phenanthren-17-yl)pentanamido) propanoate (2am): The general procedure was followed using substrate **1am** (0.20 mmol, 97 mg). Isolation by column chromatography (DCM/MeOH: 200/1→50/1) yielded **2am** (92 mg, 81%, *dr* = 1.25:1) as white solid. ^{1}H NMR (400 MHz, DMSO-d_6) δ 9.29 (s, 0.5H), 9.28 (s, 0.4H), 3.69 – 3.67 (m, 1.7H), 3.67 (s, 1.3H), 3.66 – 13.62 (m, 1H), 3.47 – 3.43 (m, 1H), 3.06 – 2.93 (m, 2H), 2.81 (t, *J* = 12.7 Hz, 1H), 2.26 – 2.11 (m, 5H), 1.98 – 1.87 (m, 4H), 1.86 – 1.77 (m, 4H), 1.73 – 1.62 (m, 2H), 1.30 (s, 3H), 1.25 – 1.19 (m, 5H), 0.98 (s, 1.3H), 0.97 (s, 1.7H), 0.76 (s, 1.3H), 0.74 (s, 1.7H). ^{13}C NMR (100 MHz, DMSO-d_6) δ 212.6, 210.2, 210.2, 174.4, 174.3, 167.6, 167.6, 76.1, 76.0, 56.8, 55.2, 55.1, 53.8, 51.8, 48.5, 46.6, 45.9, 45.1, 44.5, 43.1, 38.9, 36.7, 36.2, 35.4, 35.3, 35.1, 32.2, 31.4, 31.2, 29.6, 27.8, 25.2, 21.7, 19.3, 12.0. HR-MS (ESI) *m/z* calcd for $C_{28}H_{39}N_7NaO_6^+$ $[M+Na]^+$ 592.2854, found 592.2857.

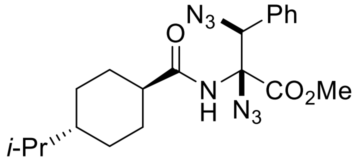

Methyl 2,3-diazido-2-((1*r*,4*r*)-4-isopropylcyclohexane-1-carboxamido)-3-phenyl propanoate (2an): The general procedure was followed using substrate **1an** (0.20 mmol, 66 mg). Isolation by column chromatography (PE/EA: 10/1→5/1) yielded **1an** (36 mg, 43%) as colourless oil liquid. ^{1}H NMR (400 MHz, $CDCl_3$) δ 7.43 (d, *J* = 8.0 Hz, 2H), 7.35 – 7.31 (m, 3H), 7.12 (s, 1H), 3.82 (s, 3H), 2.24 – 2.15 (m, 1H), 2.03 – 1.96 (m, 2H), 1.83 – 1.78 (m, 2H), 1.52 – 1.41 (m, 3H), 1.06 – 0.96 (m, 3H), 0.87 – 0.85 (d, *J* = 6.8 Hz, 7H). ^{13}C NMR (100 MHz, $CDCl_3$) δ 174.6, 166.0, 134.1, 131.8,

129.7, 129.4, 128.6, 124.4, 52.8, 45.8, 43.3, 32.9, 29.5, 29.0, 19.8. HR-MS (ESI) *m/z* calcd for $C_{20}H_{27}N_7NaO_3^+$ $[M+Na]^+$ 436.2068, found 436.2067.

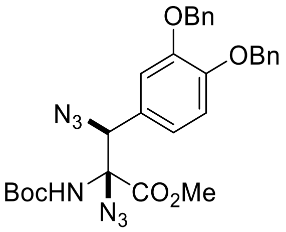

Methyl 2,3-diazido-3-(3,4-bis(benzyloxy)phenyl)-2-((*tert*-butoxycarbonyl)amino) propanoate (2ao): The general procedure was followed using substrate **1ao** (0.20 mmol, 64 mg). Isolation by column chromatography (PE/EA: 20/1→5/1) yielded **2ao** (100 mg, 88%, *dr* = 3:1) as yellow oil liquid. ^{1}H NMR (400 MHz, $CDCl_3$) δ 7.50 – 7.27 (m, 11H), 7.05 – 6.88 (m, 2H), 5.26 – 5.18 (m, 4H), 4.95 (s, 1H), 4.74 (s, 1H), 3.91 (s, 2.3H), 3.58 (s, 0.7H), 1.48 (s, 2.3H), 1.43 (s, 6.7H).^{13}C NMR (100 MHz, $CDCl_3$) δ 167.7, 166.5, 153.8, 150.7, 150.3, 149.3, 148.8, 137.0, 136.9, 136.8, 136.7, 128.8, 128.7, 128.2, 128.1, 127.5, 127.5, 127.4, 127.3, 124.6, 124.3, 123.0, 122.6, 115.6, 115.4, 114.2, 114.1, 82.2, 78.8, 77.9, 71.5, 71.4, 71.1, 69.0, 68.9, 53.9, 53.5, 28.0. HR-MS (ESI) *m/z* calcd for $C_{29}H_{31}N_7NaO_6^+$ $[M+Na]^+$ 596.2228, found 596.2228.

5. The gram-scale synthesis of 2y

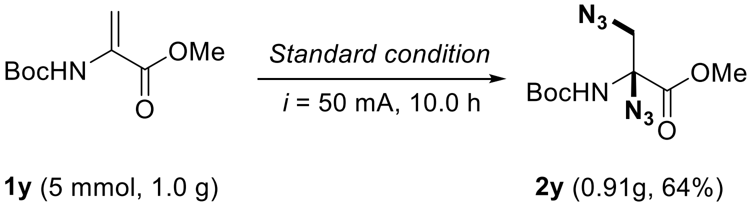

In an undivided cell (10 mL) equipped with a stirring bar, a mixture of substrates **1y** (5 mmol, 1.0 g), NaN_3 (15 mmol, 0.98g), MnF_2 (10 mol%, 0.046 g), 1,10-phen (20 mol%, 0.18 g), $LiClO_4$ (10 mmol, 1.1 g). and MeCN:DCE:AcOH (1.5:1.25:0.25, 75 mL) were added. The reaction was then initiated by applying a constant cell current of 50 mA (Pt (-), GF (+)) at room temperature for 10 h under Ar. When the reaction was finished, the solvent was concentrated under reduced pressure. The residue was purified by column chromatography on silica gel (PE/EtOAc: 100/1→10/1) yielded **2y** (0.91 g, 69%) as colorless oily liquid.

6. Intermolecular [3+2]-cycloadditions

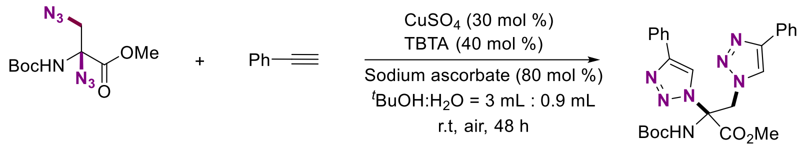

2y **3**

Phenylacetylene (816 mg, 8 mmol) was added to a solution of **3y** (910 mg, 3.64 mmol), $CuSO_4.H_2O$ (195 mg, 1.1 mmol), sodium *L*-ascorbate (218 mg, 1.1 mmol) and TBTA (586 mg, 1.1 mmol) in ^{t}BuOH (2.0 mL) and H_2O (0.60 mL). The mixture was stirred at room temperature for 48 h. The reaction was diluted with water and extracted 3 times with EtOAc. The organic layers were gathered, washed with brine, dried over MgSO4, filtered off and solvents were removed under reduced pressure. Purification by column chromatography (SiO_2, PE:EA = 10:1 to 3:1) afforded methyl 2-((tert-butoxycarbonyl)amino)-2,3-bis(4-phenyl-1H-1,2,3-triazol-1-yl)propanoate **3** (748 mg, 42%) as a white solid.

Methyl 2-((*tert*-butoxycarbonyl)amino)-2,3-bis(4-phenyl-1H-1,2,3-triazol-1-yl)pro panoate: **R_f** (PE: EA = 3:1) = 0.32. ^{1}H NMR (400 MHz, DMSO-d_6) δ 8.90 (s, 1H), 8.56 (s, 1H), 8.37 (s, 1H), 7.83 – 7.80 (m, 2H), 7.77 – 7.74 (m, 2H), 7.43 – 7.38 (m, 4H), 7.31 (dd, J = 7.8, 6.0 Hz, 2H), 5.61 (d, J = 14.6 Hz, 1H), 5.43 (d, J = 14.6 Hz, 1H), 3.72 (s, 3H), 1.33 (s, 9H). ^{13}C NMR (150 MHz, DMSO) δ 166.2, 146.6, 146.5, 130.7, 130.6, 129.6, 129.5, 129.4, 128.7, 128.6, 126.0, 125.8, 125.7, 123.7, 122.3, 121.8, 81.3, 80.7, 76.7, 54.3, 53.2, 28.3. HR-MS (ESI) *m/z* calcd for $C_{25}H_{28}N_7O_4^+$ $[M+H]^+$490.2197, found 490.2196.

7. Competition experiment

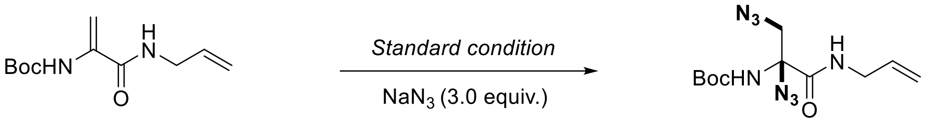

1ap **2ap**, 57%

In an undivided cell (10 mL) equipped with a stirring bar, a mixture of substrates **1ap** (0.20 mmol, 45 mg), NaN_3 (0.6 mmol, 39 mg), MnF_2 (10 mol%, 2.0 mg), 1,10-phen (20 mol%, 7.0 mg), $LiClO_4$ (0.4 mmol, 41 mg). and MeCN:DCE:AcOH (1.5:1.25:0.25,

3.0 mL) were added. The reaction was then initiated by applying a constant cell current of 8.0 mA/cm^2 (Pt (-), GF (+)) at room temperature for 5 h under Ar. When the reaction was finished, the solvent was concentrated under reduced pressure. The residue was purified by column chromatography on silica gel (PE/EtOAc: 100/1→10/1) yielded **2ap** (33 mg, 53%) as yellow oil.

***Tert*-butyl-(1-(allylamino)-2,3-diazido-1-oxopropan-2-yl)carbamate (2ap):** ^{1}H NMR (400 MHz, $CDCl_3$) δ 6.85 (t, J = 6.2 Hz, 1H), 6.07 (s, 1H), 5.91 – 5.80 (m, 1H), 5.24 (dd, J = 29.1, 13.7 Hz, 2H), 4.01 – 3.87(m, 2H), 3.78 (q, J = 12.7 Hz, 2H), 1.47 (s, 9H). ^{13}C NMR (100 MHz, $CDCl_3$) δ 166.4, 153.7, 133.1, 117.3, 81.9, 76.4, 55.8, 42.7, 28.3, 28.2. HR-MS (ESI) *m/z* calcd for $C_{11}H_{18}N_8NaO_3^+$ [M+Na]$^+$ 333.1395, found 333.1397.

8. Control experiments

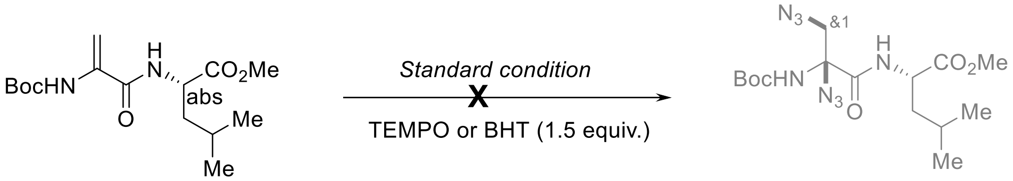

1a

not detected by
^{1}H NMR or HR-MS

In an undivided cell (10 mL) equipped with a stirring bar, a mixture of substrates **1a** (0.20 mmol), NaN_3 (0.6 mmol, 39 mg), MnF_2 (10 mol%, 1.9 mg), 1,10-phen (20 mol%, 7.2 mg), $LiClO_4$ (0.4 mmol, 41 mg), MeCN:DCE:AcOH (1.5:1.25:0.25, 3.0 mL) and H_2O (0.5mL) were added. The reaction was then initiated by applying a constant cell current of 8.0 mA/cm^2 (Pt (-), GF (+)) at room temperature for 5 h under Ar. Product **2a** was not detected by ^{1}H NMR or HS-MS.

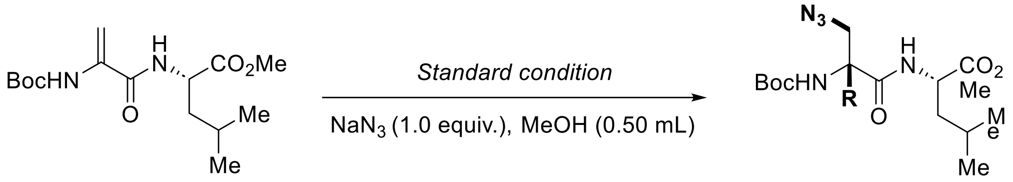

1a

2a, R = N_3, 62%
2aq, R = MeO, 0%

In an undivided cell (10 mL) equipped with a stirring bar, a mixture of substrates **1a** (0.20 mmol), NaN_3 (0.2 mmol, 39 mg), MnF_2 (10 mol%, 1.9 mg), 1,10-phen (20 mol%,

7.2 mg), $LiClO_4$ (0.4 mmol, 41 mg), MeCN:DCE:AcOH (1.5:1.25:0.25, 3.0 mL) and MeOH (0.5mL) were added. The reaction was then initiated by applying a constant cell current of 8.0 mA/cm^2 (Pt (-), GF (+)) at room temperature for 5 h under Ar. Isolation by column chromatography (PE/EA: 10/1→5/1) yielded **2a** (31 mg, 39%) as white oil.

9. UV-vis spectroscopy studies

In an undivided cell (10 mL), a mixture of **1a** (0.20 mmol), NaN_3 (0.60 mmol), MnF_2 (0.020 mmol), 1,10-Phen (0.040 mmol), $LiClO_4$ (0.40 mmol), and MeCN:DCE:AcOH(1.5:1.25:0.25, 3mL) were added. The reaction was then initiated by applying a constant cell current of 8.3 mA (Pt(-),GF (+)) at room temperature for 5 min under Ar. 0.5 mL aliquot was taken from the reaction mixture and diluted with 9.5 mL of MeCN and UV measurement was performed which showed a characteristic peak of Mn(III)-N_3 at 425 nm. This experiment suggests that the oxidation of Mn(II)-N_3 complex to Mn(III)-N_3 through electrolysis of the azide and Mn(II) mixture.

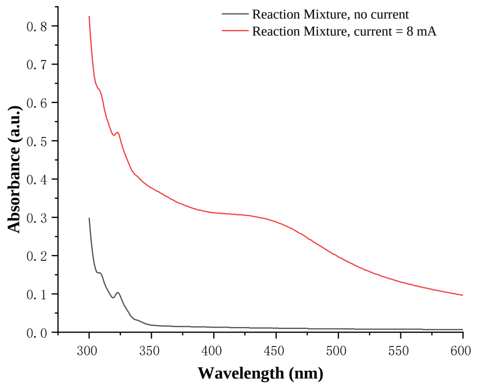

Figure S3. UV-vis spectra of diazidation reaction system before and after the application of potential.

10. Cyclic Voltammetry Studies

General information: Cyclic voltammetry (CV) studies were carried out in a 25 mL glass vial equipped with a glassy carbon working electrode, a platinum counter electrode, and a Ag/AgCl (3 M KCl) reference electrode. The electrolyte consisted of MeCN/DCE/AcOH (6:5:1 v/v, total 10 mL) with $LiClO_4$ (0.1 M) as the supporting electrolyte. The oxidation peaks of Dha (1 mM) and Dha-Leu (**1a**, 1 mM) were observed at 1.924 V and 2.443 V (vs Ag/AgCl), respectively (Figure S4). Both potentials were higher than the oxidation peak of NaN_3 (1.618 V vs Ag/AgCl, Figure

S5).

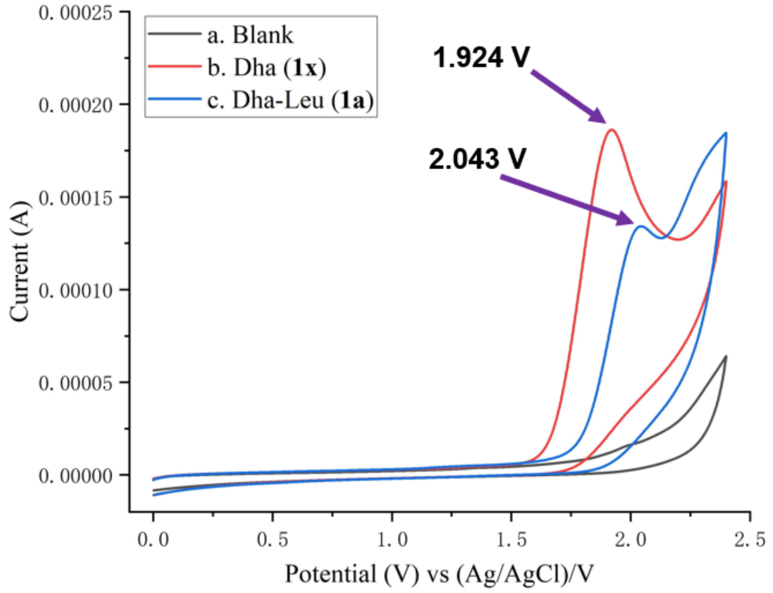

Figure S4. Cyclic voltammetry studies. Conditions: a 0.1 M $LiClO_4$ solution in MeCN/DCE/AcOH = 6:5:1 (10 mL) at room temperature; a glassy carbon working electrode, Ag/AgCl (3 M KCl) reference electrode, and a graphite counter electrode, respectively. Scan rate: 100m V/s. (a) Black line: background; (b) Red line: Dha (**1y**), oxidation peak observed at 1.924 V; (c) Blue line: Dha-Leu (**1a**), oxidation peak observed at 2.043 V.

General information: Cyclic voltammetry (CV) experiments were performed in a 25 mL glass vial equipped with a glassy carbon working electrode, a platinum counter electrode, and an Ag/AgCl (3 M KCl) reference electrode. The electrolyte solution consisted of MeCN/DCE/AcOH (6:5:1 v/v, total 10 mL) containing $LiClO_4$ (0.1 M) as the supporting electrolyte. As shown in Figure S5, the oxidation peak of NaN_3 was observed at 1.618 V (vs Ag/AgCl, line b), while MnF_2 exhibited no obvious oxidation peak under the standard solvent conditions (line c). However, in the presence of 1,10-phenanthroline, an oxidation peak of MnF_2 appeared at 1.609 V (line d). When the amount of NaN_3 was increased from 10 to 50 equiv., the oxidation peak intensity was significantly enhanced, with corresponding peaks observed at 1.487 V, 1.638 V, and 1.668 V (lines e-g). Notably, under the standard reaction conditions for manganese-catalyzed electrochemical diazidation (30 equiv. of NaN_3 added to the MnF_2 and 1,10-phenanthroline system), a new oxidation peak emerged at 0.771 V (vs Ag/AgCl, line f).

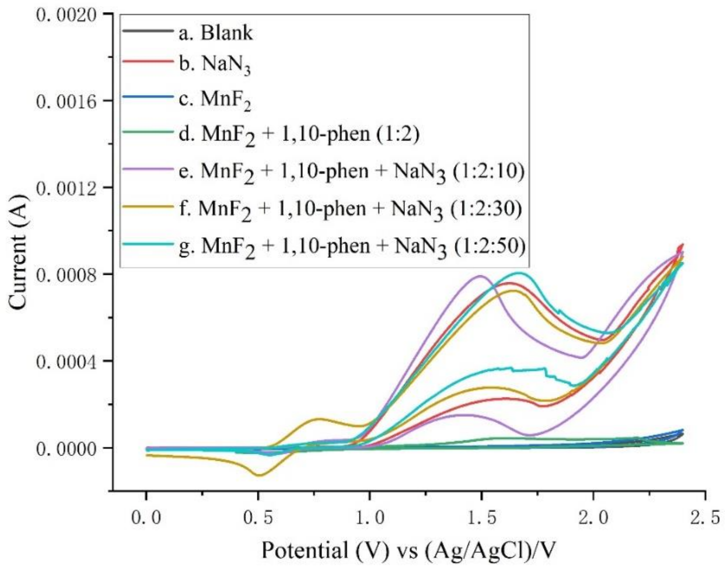

Figure S5. Cyclic voltammetry studies. Conditions: a 0.1 M $LiClO_4$ solution in MeCN/DCE/AcOH = 6:5:1 (10 mL) at room temperature; a glassy carbon working electrode, Ag/AgCl (3 M KCl) reference electrode, and a graphite counter electrode, respectively. Scan rate: 100m V/s. (a) background; (b) NaN_3, oxidation peak observed at 1.618 V; (c) MnF_2, no obivies oxidation peak observed; (d) MnF_2 + 1,4-phen (1:2), oxidation peak observed at 1.609 V; (e) MnF_2 + 1,4-phen + NaN_3 (1:2:10), oxidation peak observed at 1.487 V; (f) MnF_2 + 1,4-phen + NaN_3 (1:2:30), redox peaks observed at 0.771 vs 0.504, and oxidation peak at 1.638 V. (g) MnF_2 + 1,4-phen + NaN_3 (1:2:50), oxidation peak observed at 1.668 V.

11. Crystallographicdescriptionof 2ak.

The compound **2ak** was crystallized from MeOH. Suitable single crystal was picked from the mother liquor and covered with perfluorinated polyether oil on a microscope slide. Compound **2ak** was collected at 180.00 K on a Rigaku Oxford Diffraction Supernova Dual Source, Cu at Zero equipped with an AtlasS2 CCD using Cu Kα radiation. Data reduction was carried out with the diffractometer's software [10]. The structure was solved by direct methods using Olex2 software, and the non-hydrogen atoms were located from the trial structure and then refined anisotropically with SHELXL-2018using a full-matrix least squares procedure based on F^2. The weighted R factor, $wR$and goodness-of-fit S values were obtained based on F^2. The hydrogen atom positions were fixed geometrically at the calculated distances and allowed to ride on their parent atoms. Crystallographic data has been deposited with the Cambridge Crystallographic Centre and allocated with the deposition numbers: CCDC 2344054.

Copies of the data can be obtained free of charge from The Cambridge Crystallographic Data Centre *via* www.ccdc.cam.ac.uk/data_request/cif.

Crystal structure determination of 2ak.

Crystal Data for $C_{18}H_{28}N_8O_3$ (M =404.48 g/mol): triclinic, space group P-1 (no. 2), a = 8.82930(10) Å, b = 11.32510(10) Å, c = 11.5341(2) Å, α = 77.0450(10)°, β = 72.2040(10)°, γ = 87.5370(10)°, V = 1069.78(3) Å3, Z = 2, T = 149.99(10) K, μ(Cu Kα) = 0.735 mm-1, Dcalc = 1.256 g/cm3, 9782 reflections measured (8.014° ≤ 2Θ ≤ 143.06°), 4020 unique (Rint = 0.0156, Rsigma = 0.0171) which were used in all calculations. The final R1 was 0.0444 (I > 2σ (I)) and wR2 was 0.1133 (all data).

Table S5 Crystal data and structure refinement for 2ak.

Identification code	**2ak**
Empirical formula	$C_{18}H_{28}N_8O_3$
Formula weight	404.48
Temperature/K	149.99(10)
Crystal system	triclinic
Space group	P-1
a/Å	8.82930(10)
b/Å	11.32510(10)
c/Å	11.5341(2)
α/°	77.0450(10)
β/°	72.2040(10)
γ/°	87.5370(10)
Volume/Å^3	1069.78(3)
Z	2
ρ_{calc}g/cm^3	1.256
μ/mm^{-1}	0.735
F(000)	432.0
Crystal size/mm^3	0.14 × 0.12 × 0.1
Radiation	Cu Kα (λ = 1.54184)
2Θ range for data collection/°	8.014 to 143.06
Index ranges	-10 ≤ h ≤ 10, -12 ≤ k ≤ 13, -14 ≤ l ≤ 14
Reflections collected	9782
Independent reflections	4020 [R_{int} = 0.0156, R_{sigma} = 0.0171]
Data/restraints/parameters	4020/7/269
Goodness-of-fit on F^2	1.031
Final R indexes [I>=2σ (I)]	R_1 = 0.0444, wR_2 = 0.1102
Final R indexes [all data]	R_1 = 0.0490, wR_2 = 0.1133

Largest diff. peak/hole / e Å^{-3}	0.28/-0.26

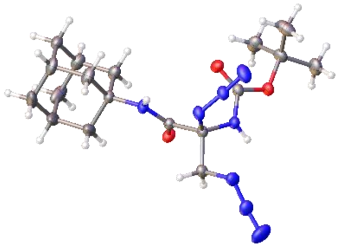

Figure S6. X-ray structure of **2ak**.Ellipsoids show 30% probability levels. H atoms are omitted for clarity.

Table S6. Bond Lengths for **2ak**.

Atom	Atom	Length/Å	Atom	Atom	Length/Å
O1	C11	1.2301(17)	C2	C3	1.517(2)
O2	C14	1.2112(18)	C2	C10	1.502(3)
O3	C14	1.3405(17)	C3	C4	1.516(3)
O3	C16	1.4786(17)	C4	C5	1.534(2)
N1	C6	1.4723(19)	C4	C9	1.535(3)
N1	C11	1.3334(18)	C5	C6	1.530(2)
N2	N3	1.2294(17)	C6	C7	1.522(2)
N2	C12	1.4967(17)	C7	C8	1.544(3)
N3	N4	1.1307(19)	C8	C9	1.535(3)
N5	N6	1.2266(19)	C8	C10	1.515(4)
N5	C13	1.4753(19)	C11	C12	1.554(2)
N6	N7	1.128(2)	C12	C13	1.5287(19)
N8	C12	1.4364(17)	C15	C16	1.516(2)
N8	C14	1.3626(19)	C16	C17	1.519(2)
C1	C2	1.534(2)	C16	C18	1.514(3)
C1	C6	1.527(2)			

Table S7. Bond Angles for **2ak**.

Atom	Atom	Atom	Angle/°	Atom	Atom	Atom	Angle/°
C14	O3	C16	120.51(11)	C10	C8	C7	109.85(17)
C11	N1	C6	126.35(12)	C10	C8	C9	109.82(16)
N3	N2	C12	115.31(11)	C8	C9	C4	109.14(14)
N4	N3	N2	174.73(15)	C2	C10	C8	109.82(16)
N6	N5	C13	114.95(13)	O1	C11	N1	125.86(14)
N7	N6	N5	172.73(18)	O1	C11	C12	117.97(12)
C14	N8	C12	119.85(12)	N1	C11	C12	116.07(12)

C6	C1	C2	109.33(13)	N2	C12	C11	109.78(11)
C3	C2	C1	110.31(14)	N2	C12	C13	106.49(11)
C10	C2	C1	109.38(14)	N8	C12	N2	113.18(11)
C10	C2	C3	109.87(16)	N8	C12	C11	111.00(11)
C4	C3	C2	109.34(13)	N8	C12	C13	109.23(11)
C3	C4	C5	109.35(14)	C13	C12	C11	106.87(11)
C3	C4	C9	109.29(16)	N5	C13	C12	108.13(11)
C5	C4	C9	109.20(16)	O2	C14	O3	126.96(13)
C6	C5	C4	110.11(14)	O2	C14	N8	123.81(13)
N1	C6	C1	110.89(12)	O3	C14	N8	109.22(12)
N1	C6	C5	107.47(12)	O3	C16	C15	101.74(12)
N1	C6	C7	110.57(12)	O3	C16	C17	109.87(13)
C1	C6	C5	107.96(13)	O3	C16	C18	109.39(13)
C7	C6	C1	109.73(14)	C15	C16	C17	111.06(15)
C7	C6	C5	110.16(14)	C18	C16	C15	111.08(14)
C6	C7	C8	108.98(13)	C18	C16	C17	113.09(16)
C9	C8	C7	108.8(2)				

12. References

[1] Aycock, R. A.; Pratt, C. J.; Jui, N. T. Aminoalkyl Radicals as Powerful Intermediates for the Synthesis of Unnatural Amino Acids and Peptides. *ACS Catal.*, **2018**, *8*, 9115-9119.

[2] Wan, Y.; Zhu, J.; Yuan, Q.; Wang, W.; Zhang, Y. Synthesis of *β*-Silyl α-Amino Acids via Visible-Light-Mediated Hydrosilylation. *Org. Lett.*, **2021**, *23*, 1406-1410.

[3] Qi, X.; Jambu, S.; Ji, Y.; Belyk, K. M.; Panigrahi, N. R.; Arora, P. S.; Strotman, N. A.; Diao, T. Late-Stage Modification of Oligopeptides by Nickel-Catalyzed Stereoselective Radical Addition to Dehydroalanine. *Angew. Chem. Int. Ed.*, **2022**, *61*, e202213315.

13. ^{1}H, ^{13}C, and ^{19}F Spectra

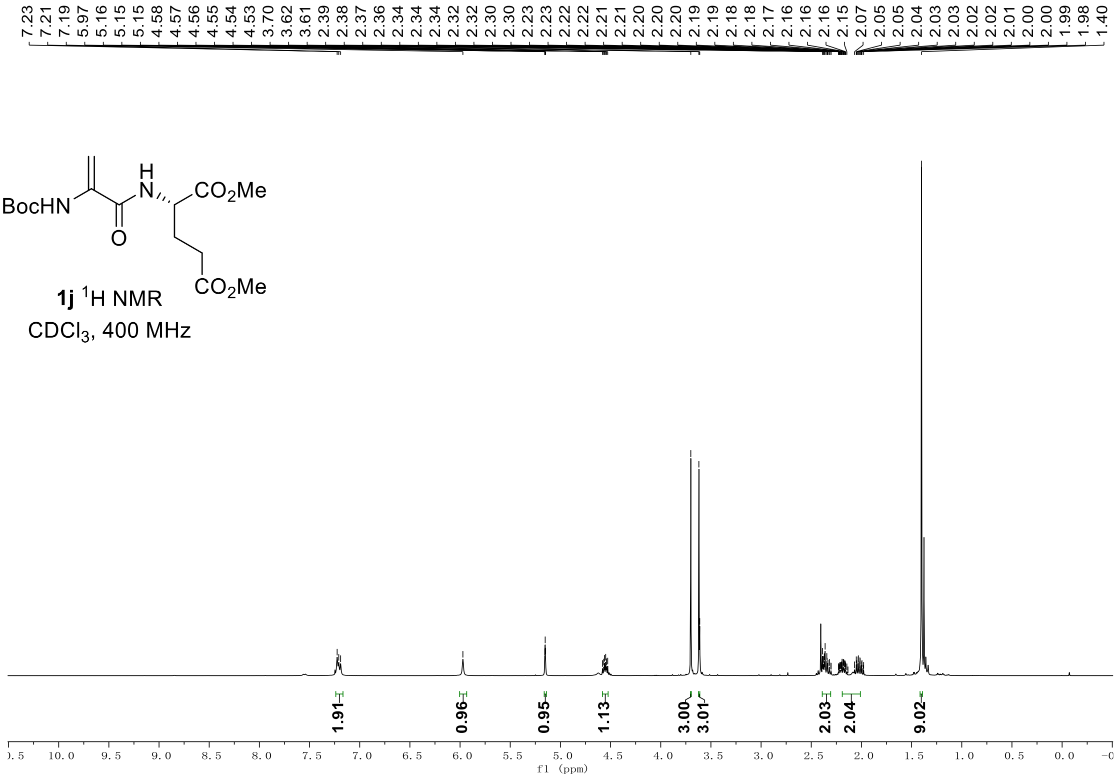

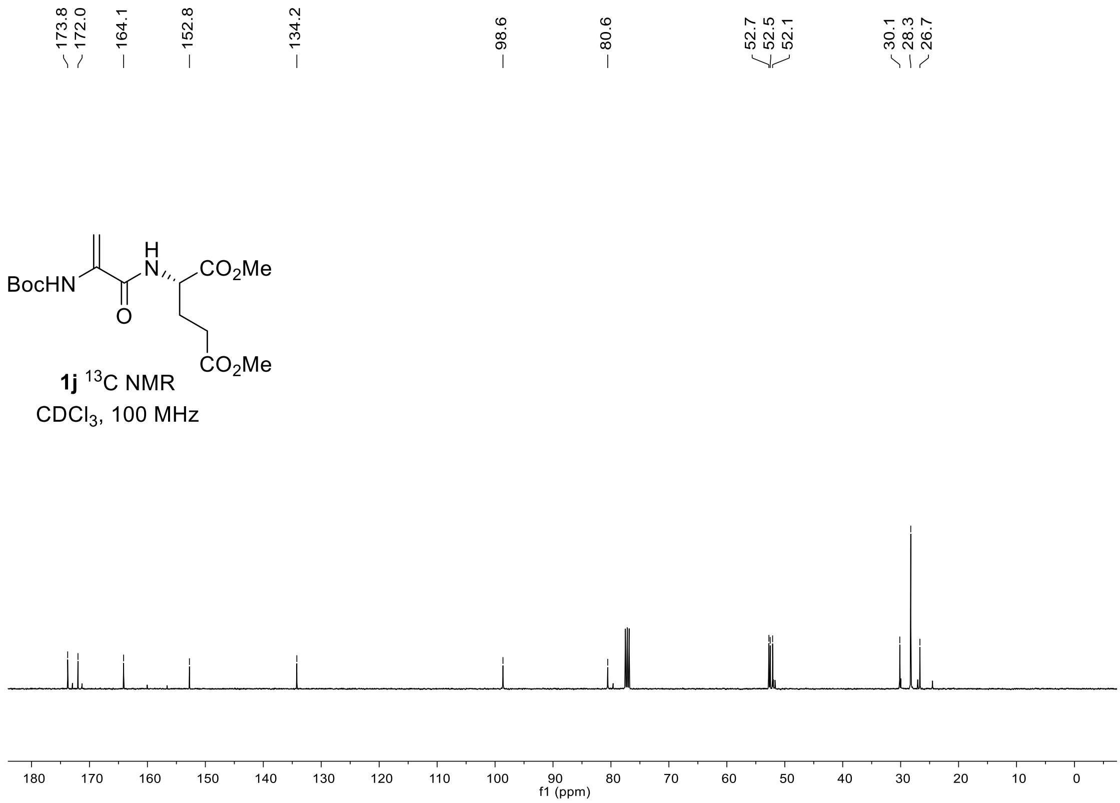

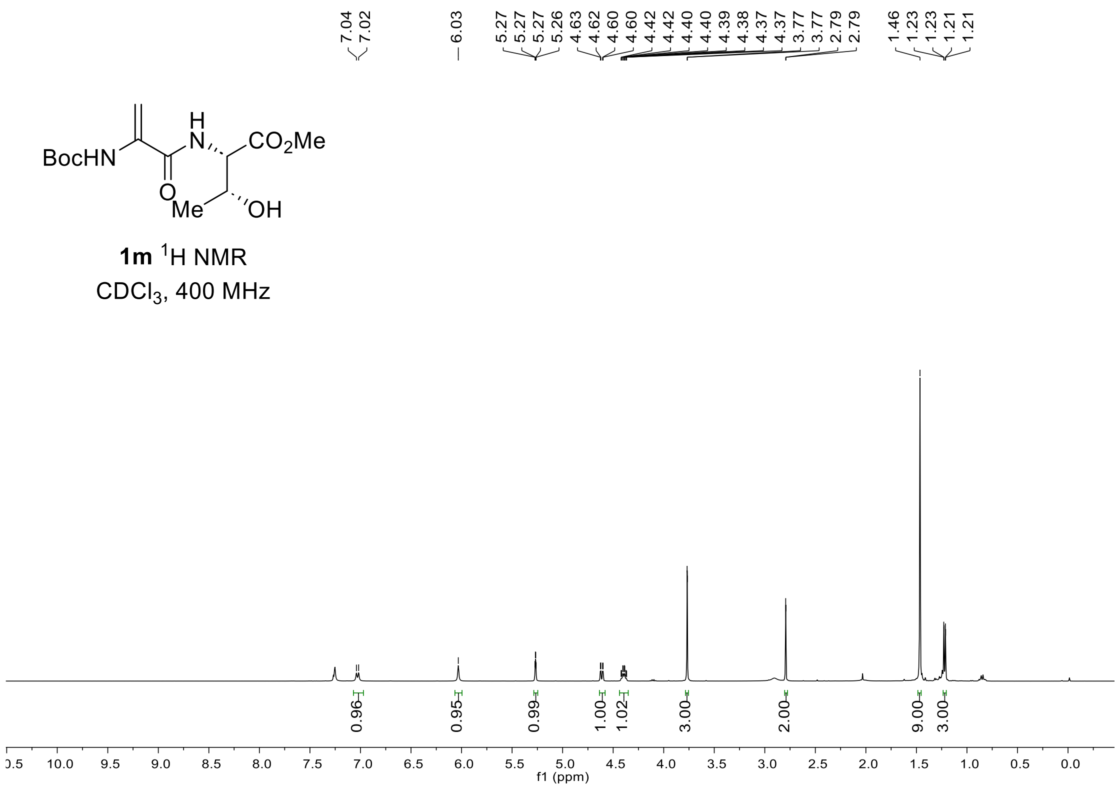
7.04
7.02
6.03
5.27
5.27
5.27
5.26
4.63
4.62
4.60
4.60
4.42
4.42
4.40
4.40
4.39
4.38
4.37
4.37
3.77
3.77
2.79
2.79
1.46
1.23
1.23
1.21
1.21
BocHN
H
N
CO2Me
O
Me
OH
1m 1H NMR
CDCl3, 400 MHz
0.96
0.95
0.99
1.00
1.02
3.00
2.00
9.00
3.00
f1 (ppm)

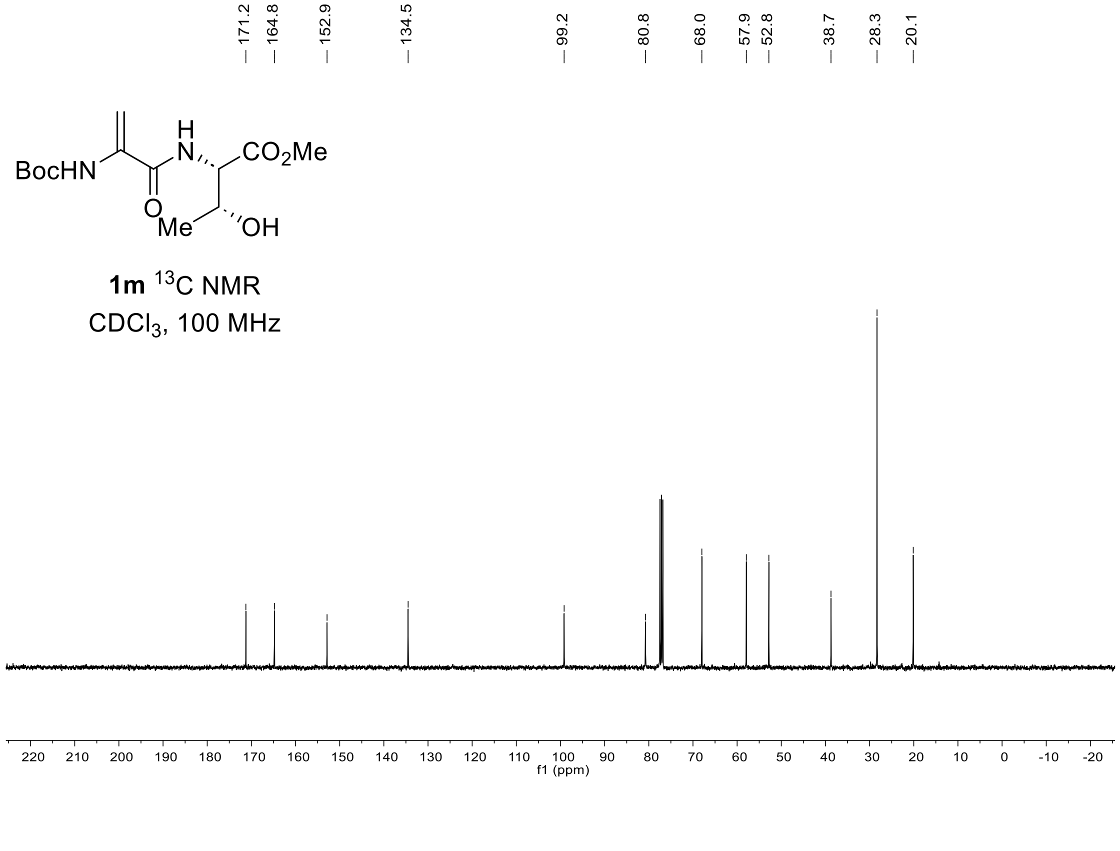
171.2
164.8
152.9
134.5
99.2
80.8
68.0
57.9
52.8
38.7
28.3
20.1
BocHN
H
N
CO2Me
O
Me
OH
1m 13C NMR
CDCl3, 100 MHz
f1 (ppm)

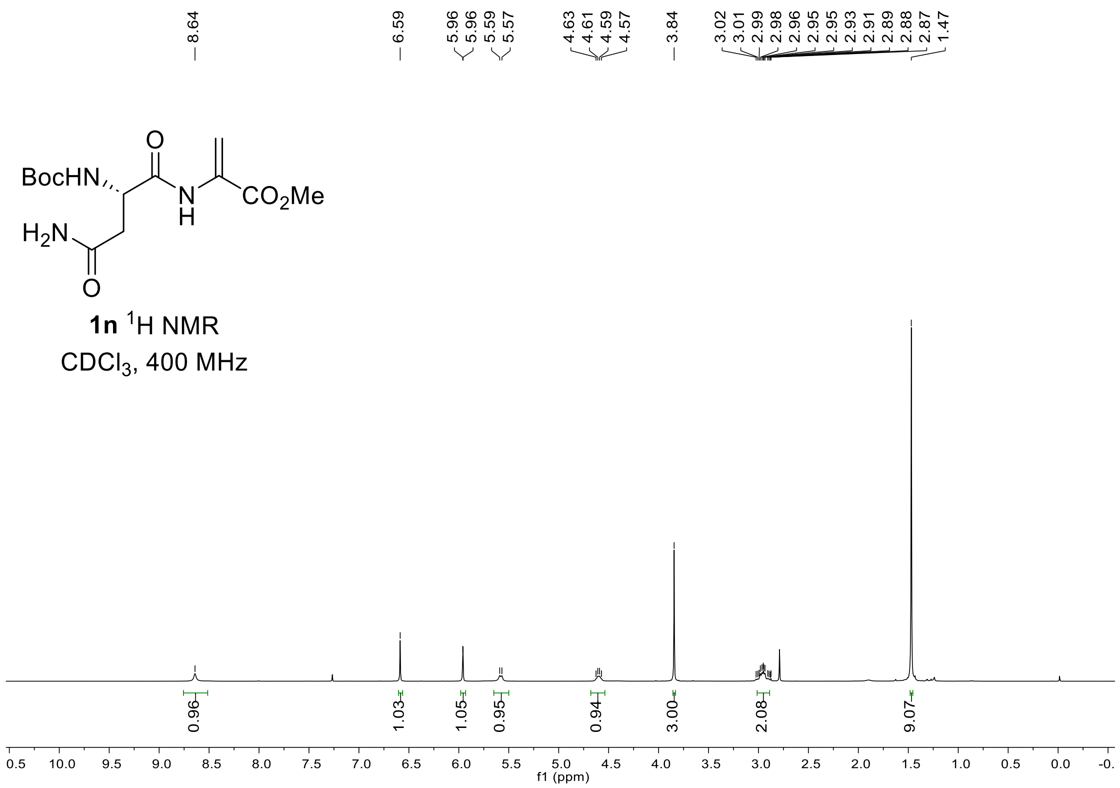

8.64
6.59
5.96
5.96
5.59
5.57
4.63
4.61
4.59
4.57
3.84
3.02
3.01
2.99
2.98
2.96
2.95
2.95
2.93
2.91
2.89
2.88
2.87
1.47
BocHN
O
N
H
CO2Me
H2N
O
1n 1H NMR
CDCl3, 400 MHz
0.96
1.03
1.05
0.95
0.94
3.00
2.08
9.07
f1 (ppm)

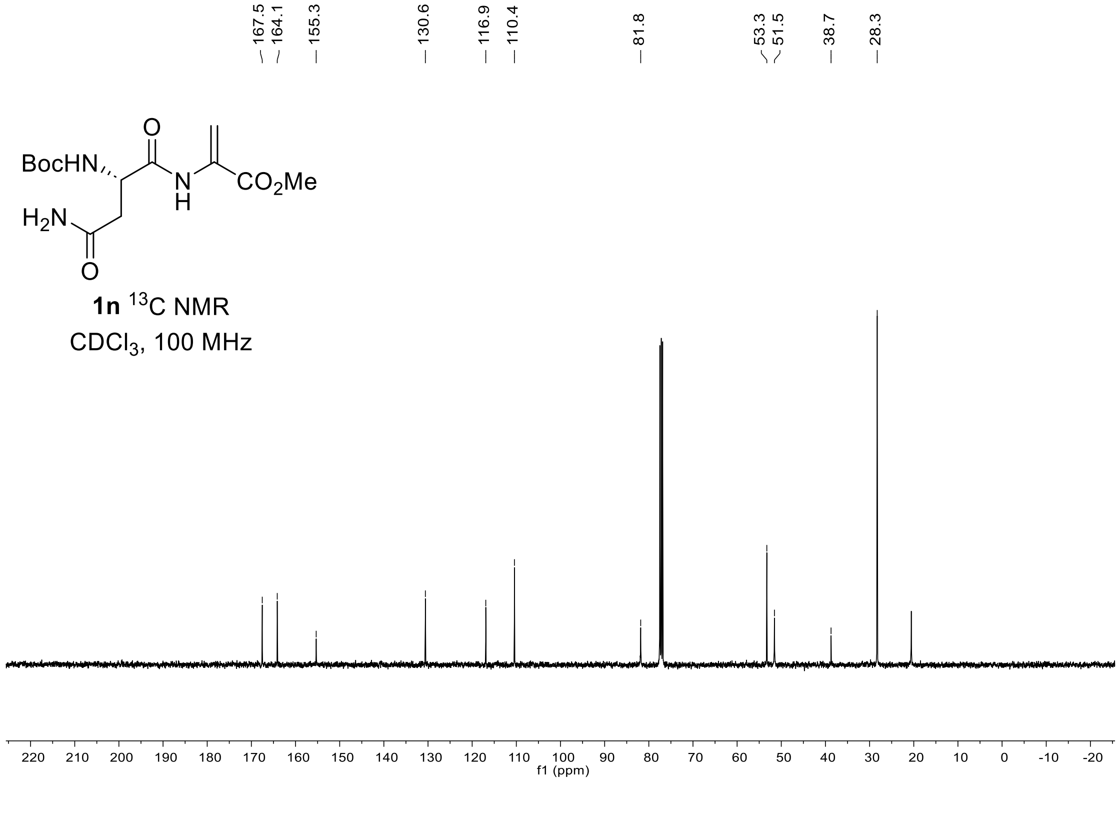

167.5
164.1
155.3
130.6
116.9
110.4
81.8
53.3
51.5
38.7
28.3
BocHN
O
N
H
CO2Me
H2N
O
1n 13C NMR
CDCl3, 100 MHz
f1 (ppm)

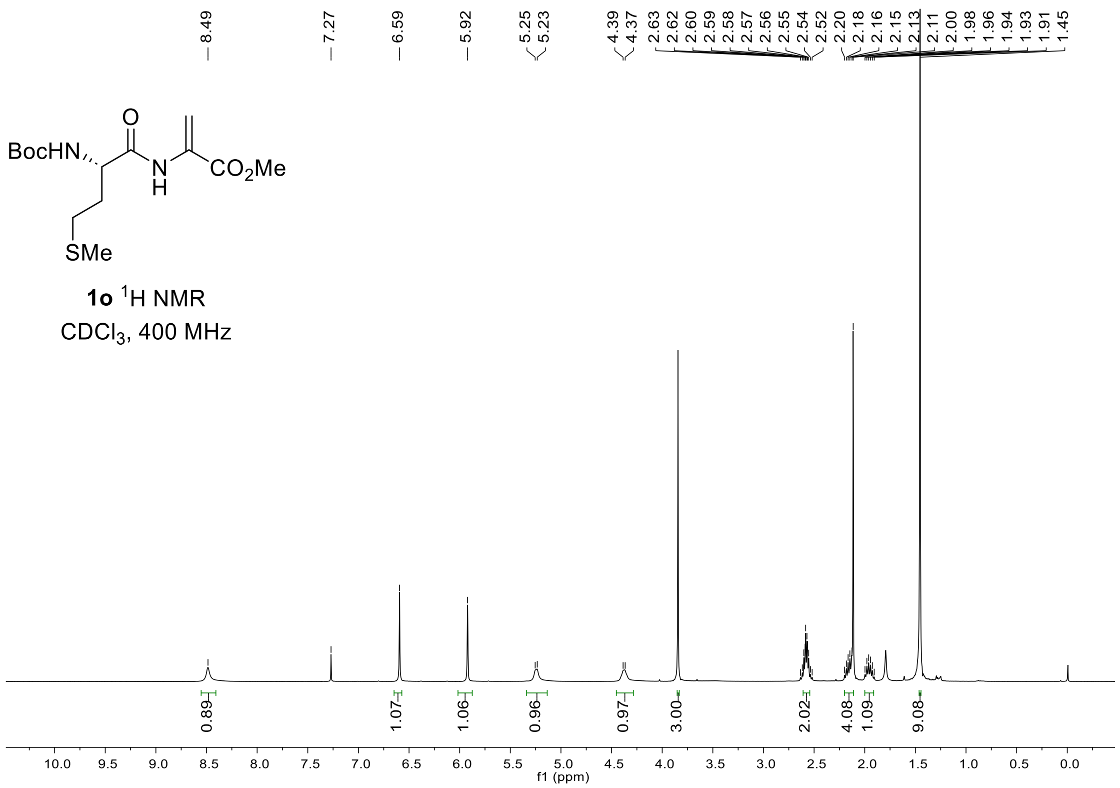
8.49
7.27
6.59
5.92
5.25
5.23
4.39
4.37
2.63 2.62 2.60 2.59 2.58 2.57 2.56 2.55 2.54 2.52 2.20 2.18 2.16 2.15 2.13 2.11 2.00 1.98 1.96 1.94 1.93 1.91 1.45
BocHN
O
N
H
CO2Me
SMe
1o 1H NMR
CDCl3, 400 MHz
0.89
1.07
1.06
0.96
0.97
3.00
2.02
4.08
1.09
9.08
10.0 9.5 9.0 8.5 8.0 7.5 7.0 6.5 6.0 5.5 5.0 4.5 4.0 3.5 3.0 2.5 2.0 1.5 1.0 0.5 0.0
f1 (ppm)

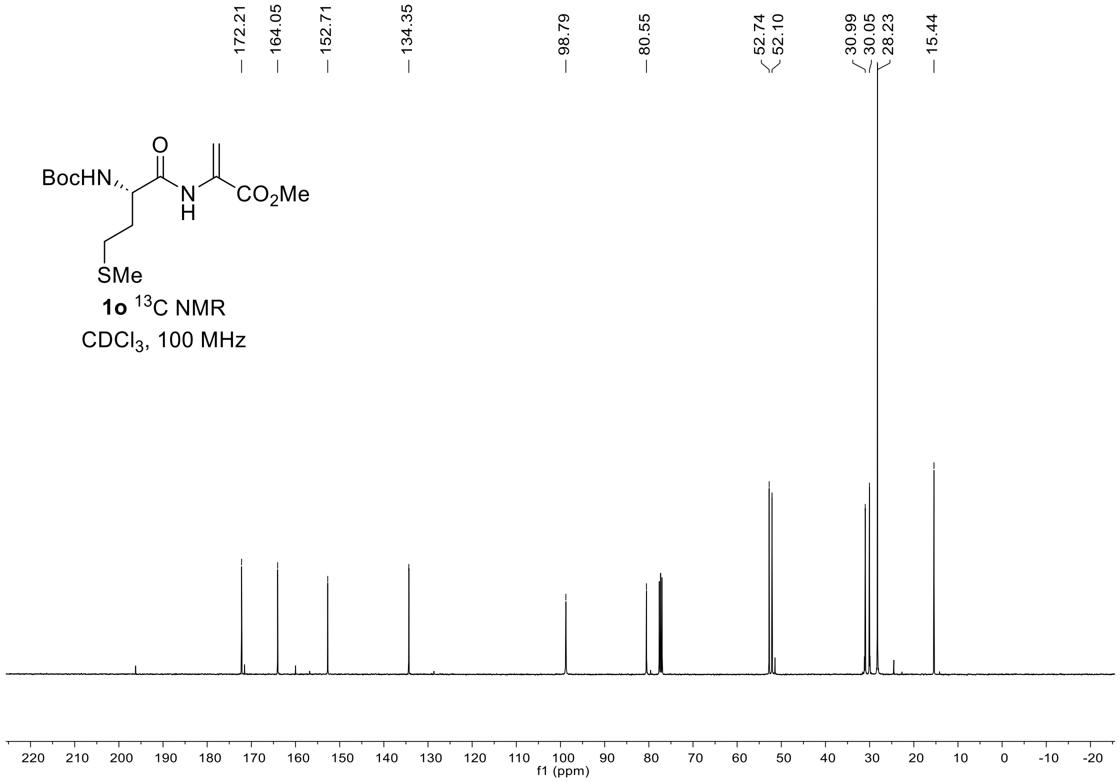
172.21
164.05
152.71
134.35
98.79
80.55
52.74
52.10
30.99
30.05
28.23
15.44
BocHN
O
N
H
CO2Me
SMe
1o 13C NMR
CDCl3, 100 MHz
220 210 200 190 180 170 160 150 140 130 120 110 100 90 80 70 60 50 40 30 20 10 0 -10 -20
f1 (ppm)

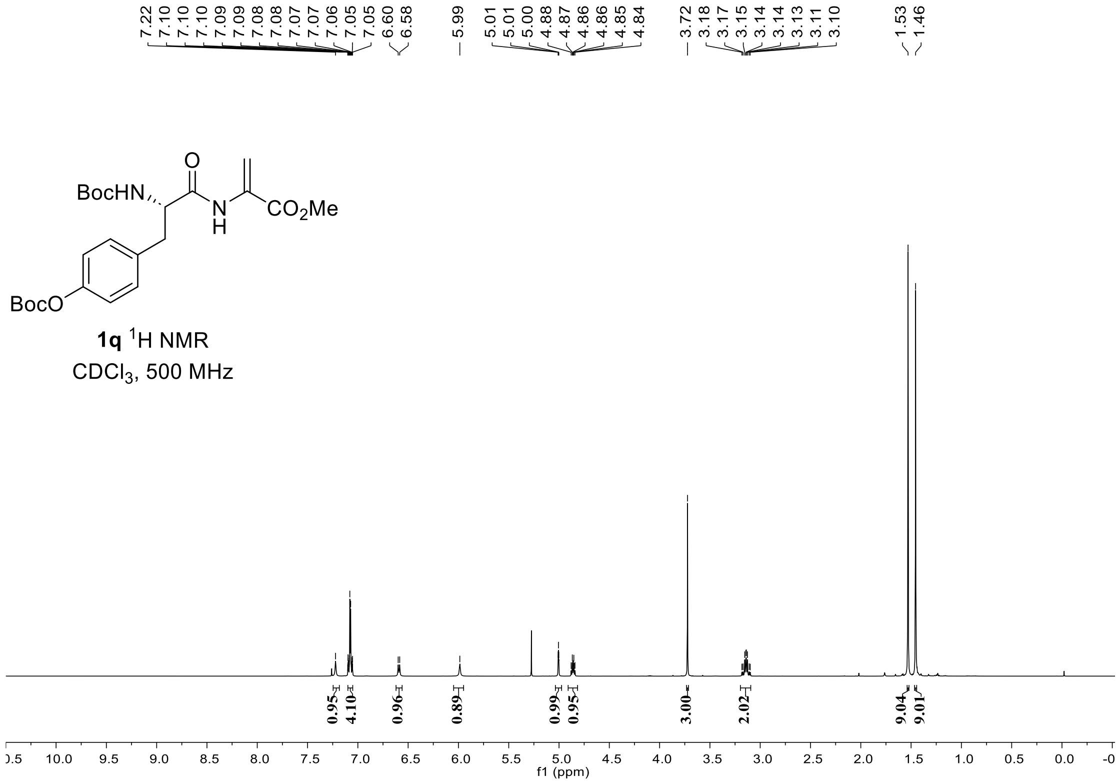

7.22
7.10
7.10
7.09
7.09
7.08
7.07
7.07
7.06
7.05
7.05
6.60
6.58
5.99
5.01
5.01
5.00
4.88
4.87
4.86
4.86
4.85
4.84
3.72
3.18
3.17
3.15
3.14
3.14
3.13
3.11
3.10
1.53
1.46
BocHN
O
N
H
CO2Me
BocO
1q 1H NMR
CDCl3, 500 MHz
0.95
4.10
0.96
0.89
0.99
0.95
3.00
2.02
9.04
9.01
f1 (ppm)

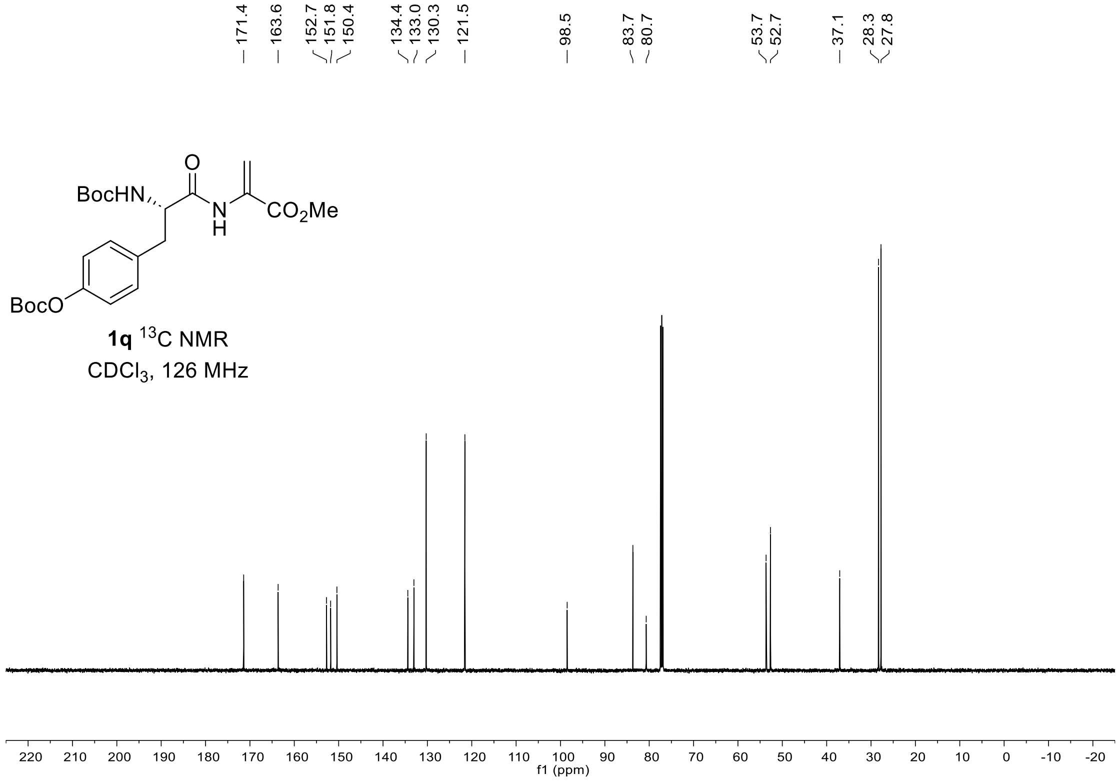

171.4
163.6
152.7
151.8
150.4
134.4
133.0
130.3
121.5
98.5
83.7
80.7
53.7
52.7
37.1
28.3
27.8
BocHN
O
N
H
CO2Me
BocO
1q 13C NMR
CDCl3, 126 MHz
f1 (ppm)

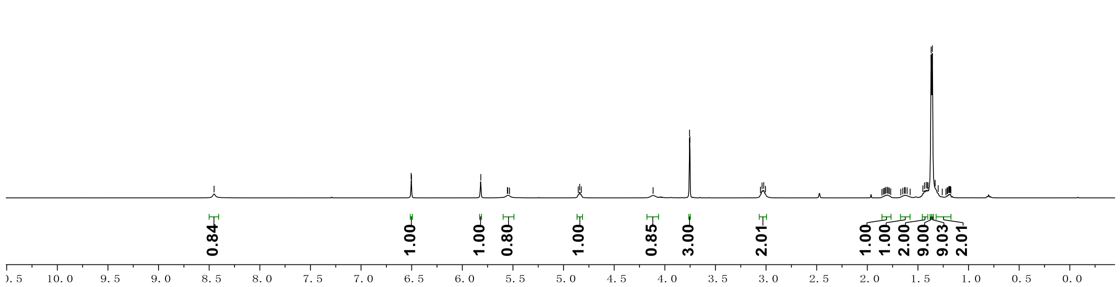

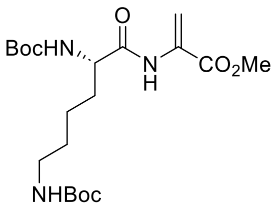

1r ^{1}H NMR

$CDCl_3$, 500 MHz

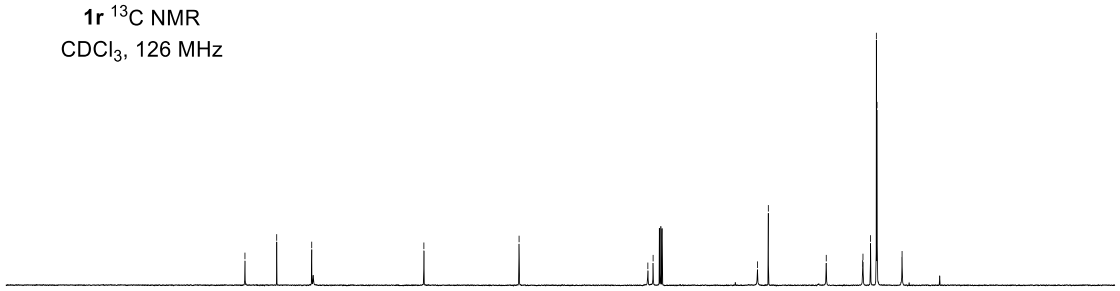

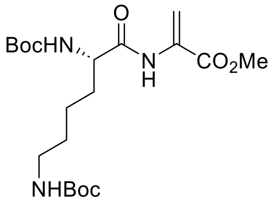

1r ^{13}C NMR

$CDCl_3$, 126 MHz

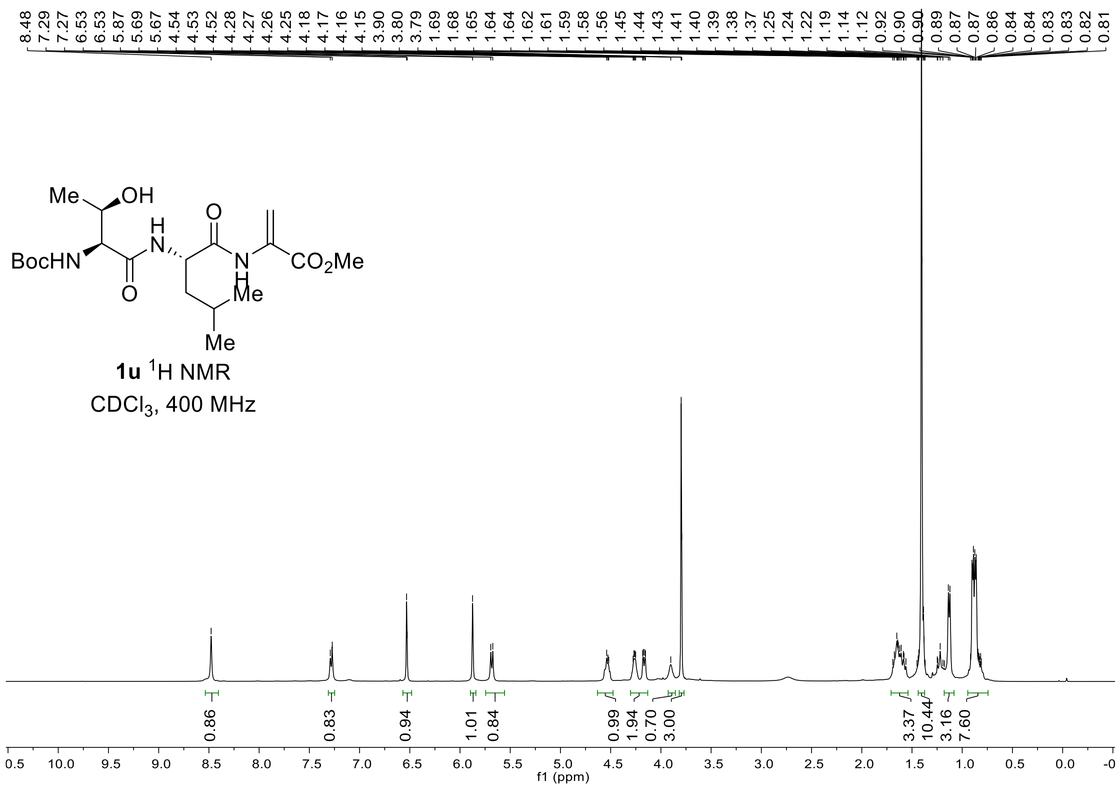
8.48 7.29 7.27 6.53 6.53 5.87 5.69 5.67 4.54 4.53 4.52 4.28 4.27 4.26 4.25 4.18 4.17 4.16 4.15 3.90 3.80 3.79 1.69 1.68 1.65 1.64 1.64 1.62 1.61 1.59 1.58 1.56 1.45 1.44 1.43 1.41 1.40 1.39 1.38 1.37 1.25 1.24 1.22 1.19 1.14 1.12 0.92 0.90 0.90 0.89 0.87 0.87 0.86 0.84 0.84 0.83 0.83 0.82 0.81
Me
OH
BocHN
H
N
O
O
N
H
CO2Me
Me
Me
1u 1H NMR
CDCl3, 400 MHz
0.86
0.83
0.94
1.01
0.84
0.99
1.94
0.70
3.00
3.37
10.44
3.16
7.60
f1 (ppm)

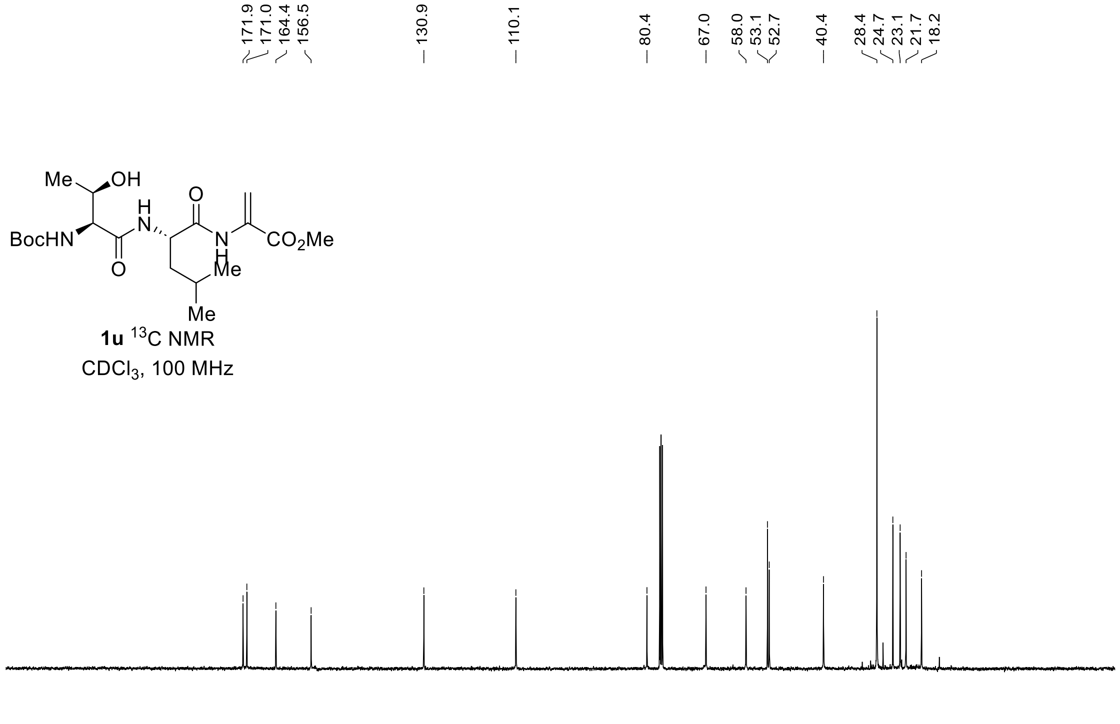
171.9
171.0
164.4
156.5
130.9
110.1
80.4
67.0
58.0
53.1
52.7
40.4
28.4
24.7
23.1
21.7
18.2
Me
OH
BocHN
H
N
O
O
N
H
CO2Me
Me
Me
1u 13C NMR
CDCl3, 100 MHz
f1 (ppm)

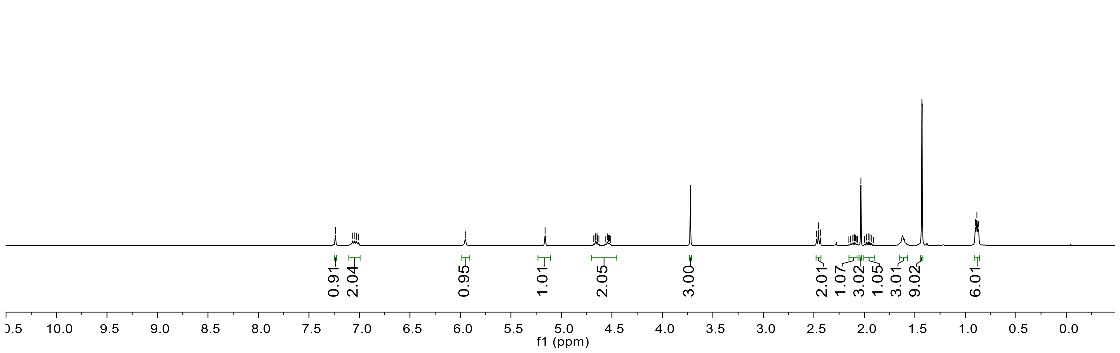

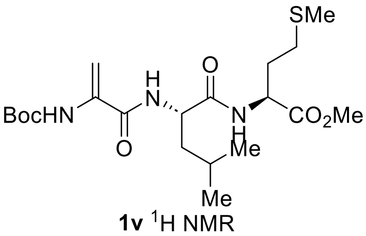

1v ^{1}H NMR

$CDCl_3$, 400 MHz

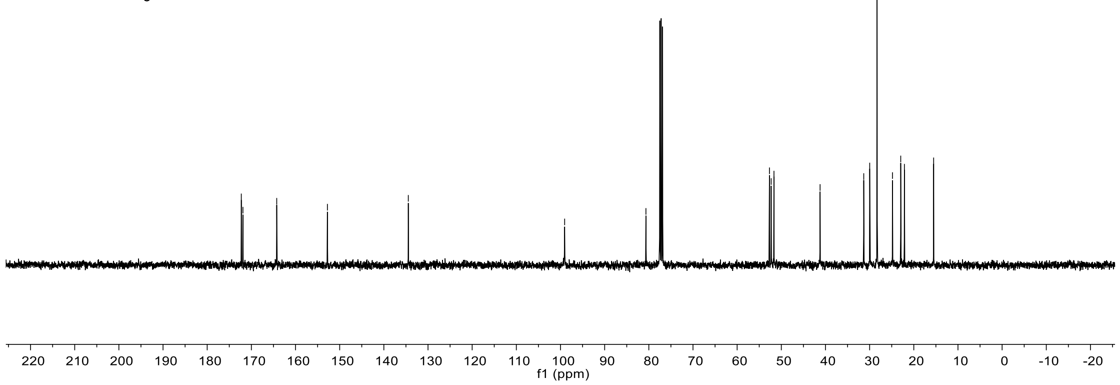

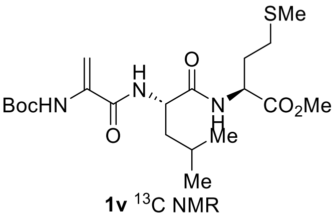

1v ^{13}C NMR

$CDCl_3$, 100 MHz

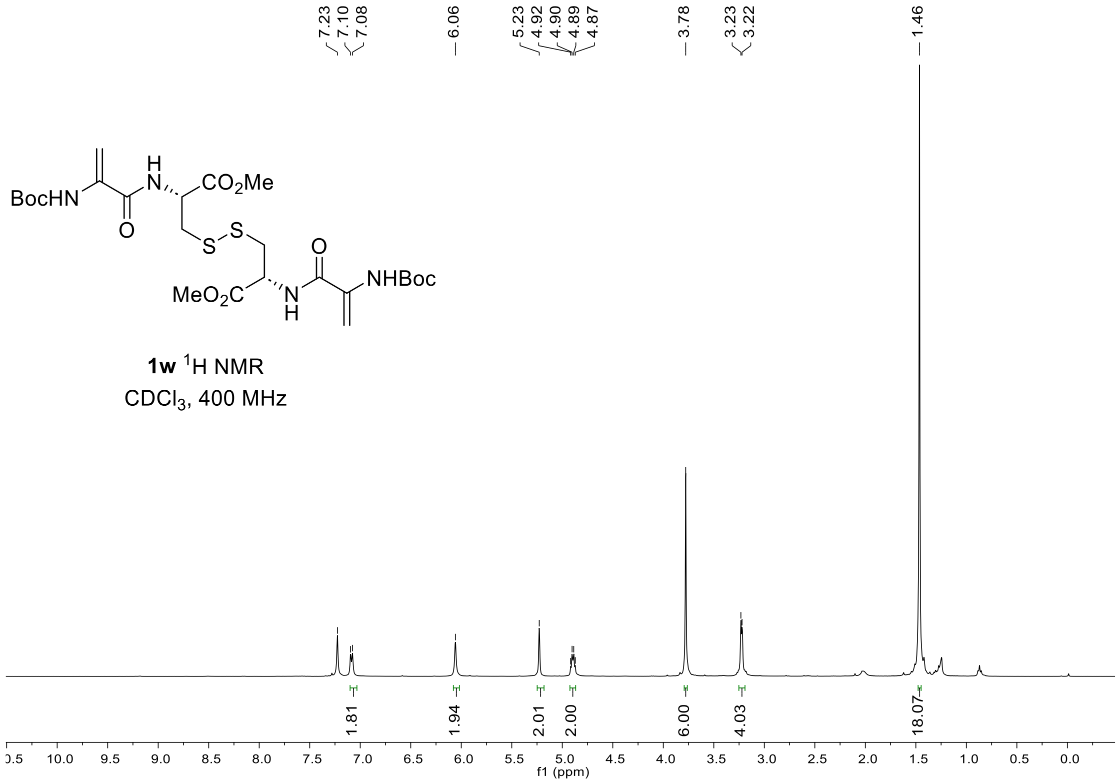

7.23
7.10
7.08
6.06
5.23
4.92
4.90
4.89
4.87
3.78
3.23
3.22
1.46
BocHN
CO2Me
MeO2C
NHBoc
1w 1H NMR
CDCl3, 400 MHz
1.81
1.94
2.01
2.00
6.00
4.03
18.07
f1 (ppm)

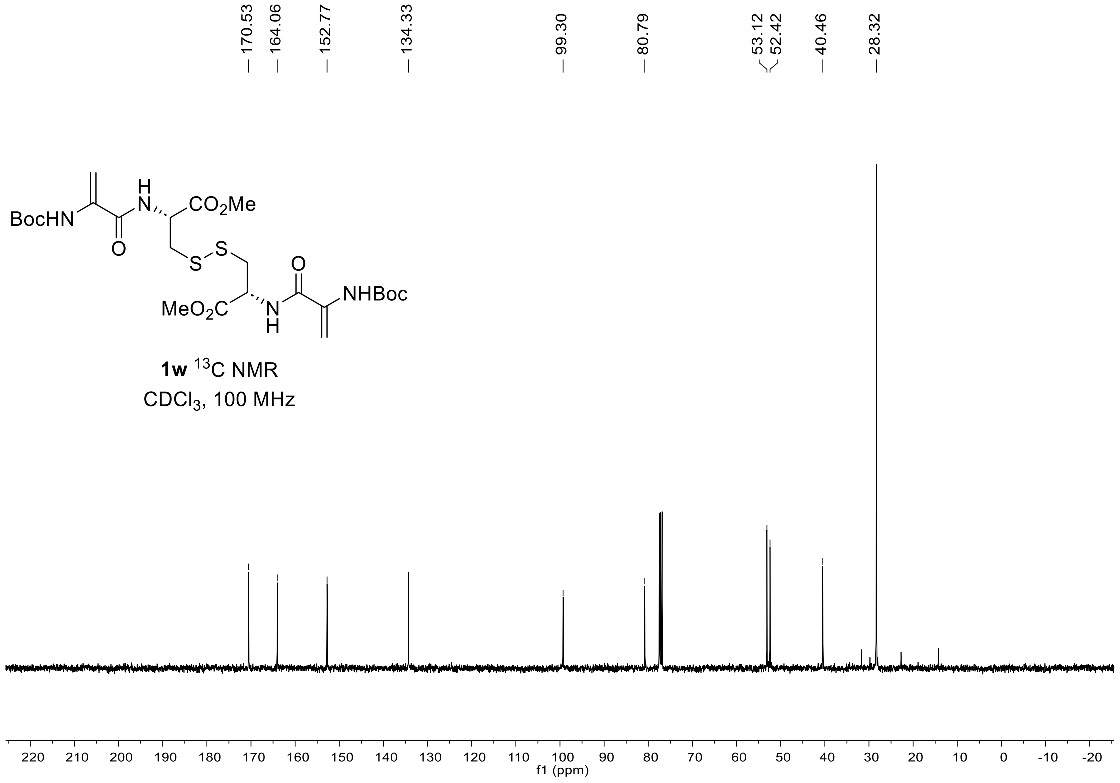

170.53
164.06
152.77
134.33
99.30
80.79
53.12
52.42
40.46
28.32
BocHN
CO2Me
MeO2C
NHBoc
1w 13C NMR
CDCl3, 100 MHz
f1 (ppm)

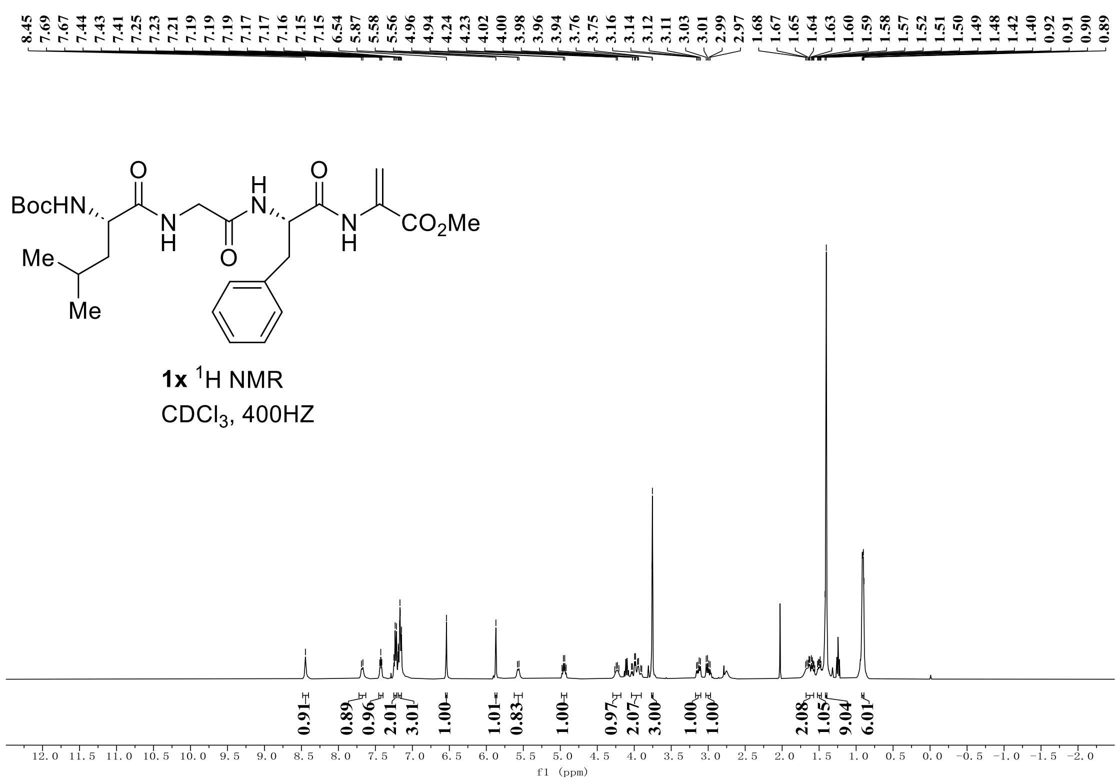

1x ^{1}H NMR

$CDCl_3$, 400HZ

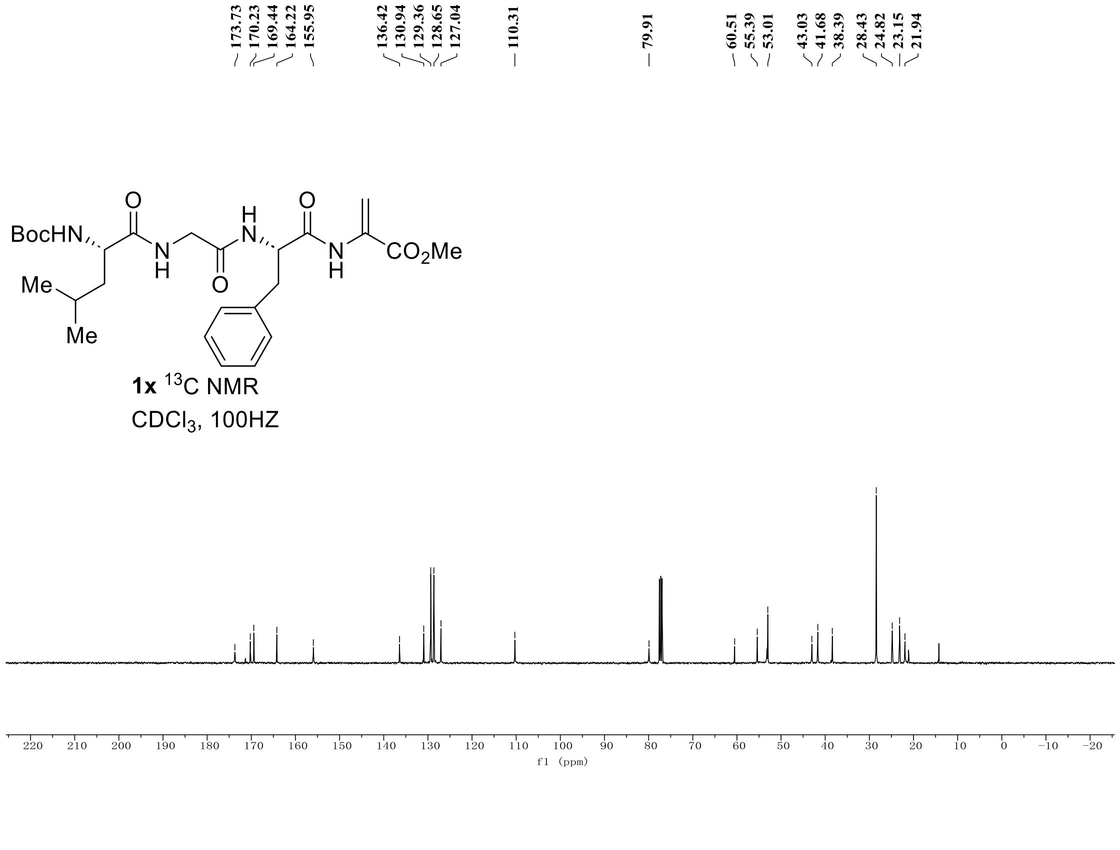

1x ^{13}C NMR

$CDCl_3$, 100HZ

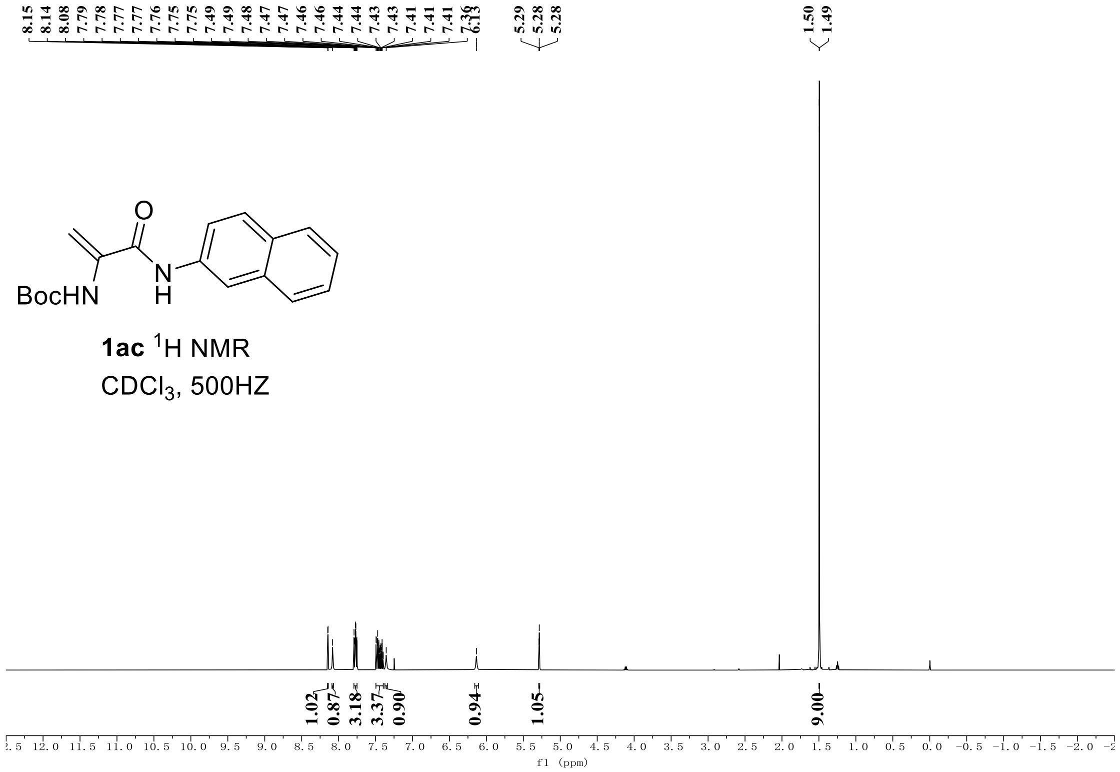
8.15 8.14 8.08 7.79 7.78 7.77 7.77 7.76 7.75 7.75 7.49 7.49 7.48 7.47 7.47 7.46 7.46 7.44 7.44 7.43 7.43 7.41 7.41 7.41 7.36 6.13
5.29 5.28 5.28
1.50 1.49
O
BocHN
N
H
1ac 1H NMR
CDCl3, 500HZ
1.02 0.87 3.18 3.37 0.90 0.94 1.05 9.00
f1 (ppm)

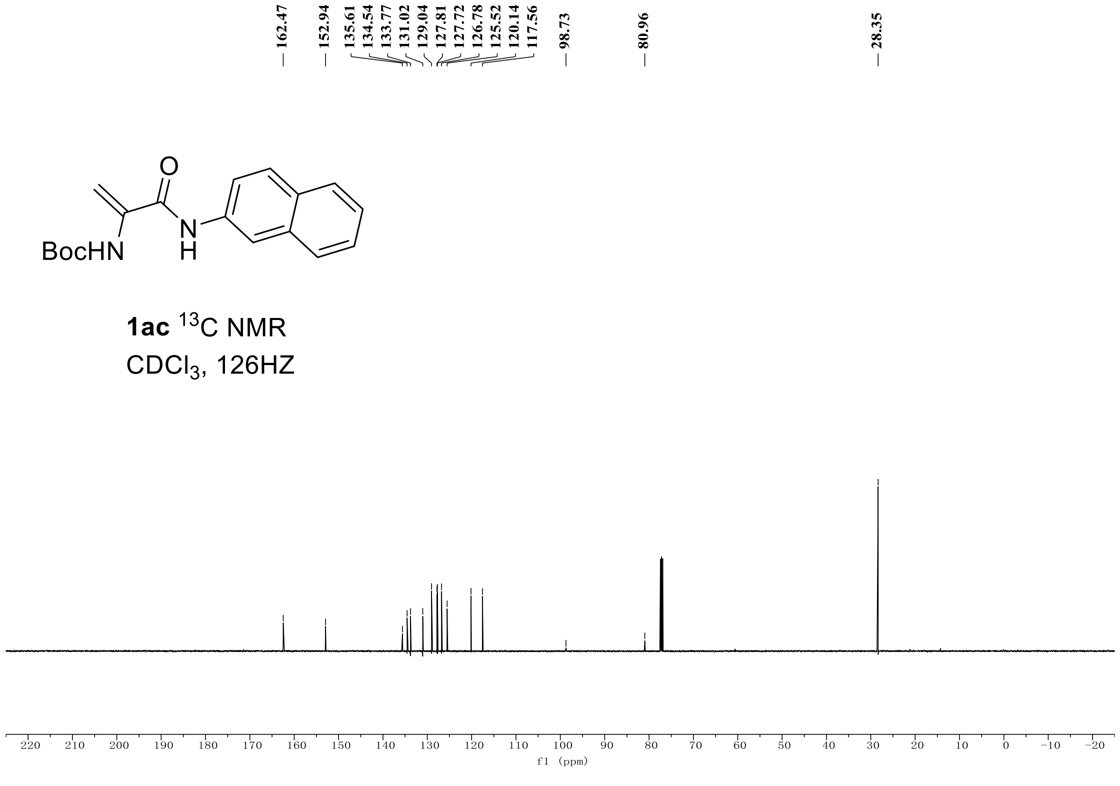
162.47 152.94 135.61 134.54 133.77 131.02 129.04 127.81 127.72 126.78 125.52 120.14 117.56 98.73 80.96 28.35
O
BocHN
N
H
1ac 13C NMR
CDCl3, 126HZ
f1 (ppm)

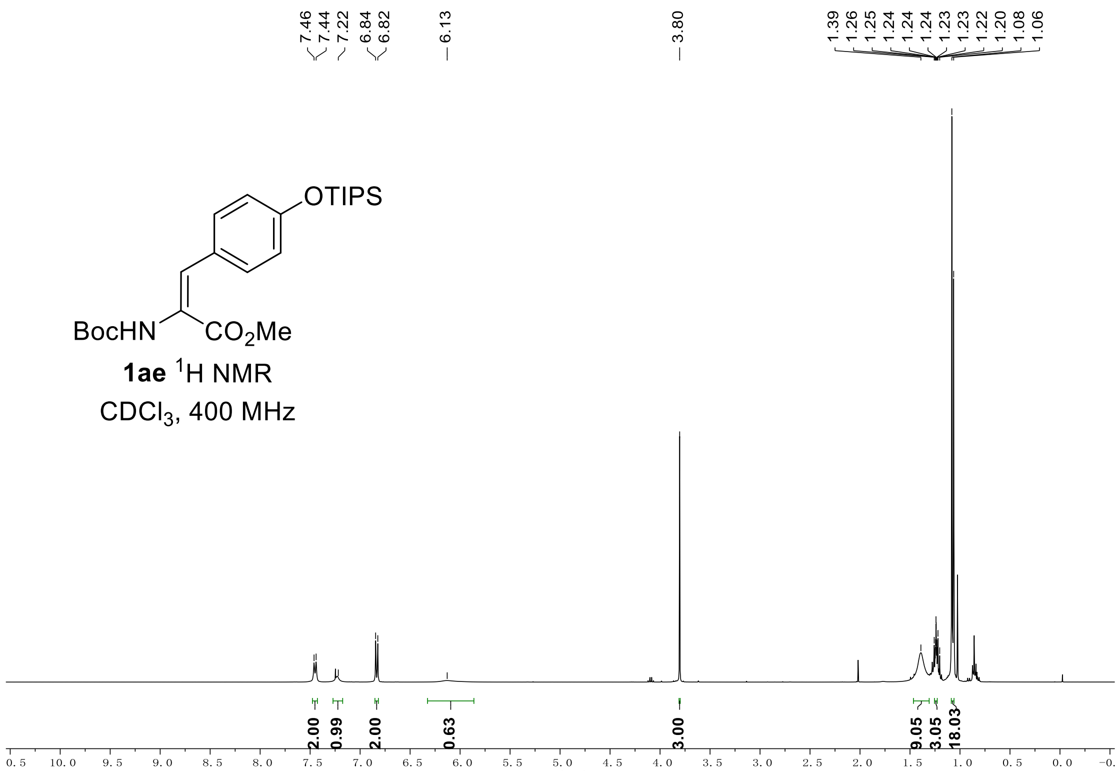
7.46
7.44
7.22
6.84
6.82
6.13
3.80
1.39
1.26
1.25
1.24
1.24
1.24
1.23
1.23
1.22
1.20
1.08
1.06
OTIPS
BocHN
CO2Me
1ae 1H NMR
CDCl3, 400 MHz
2.00
0.99
2.00
0.63
3.00
9.05
3.05
18.03
f1 (ppm)

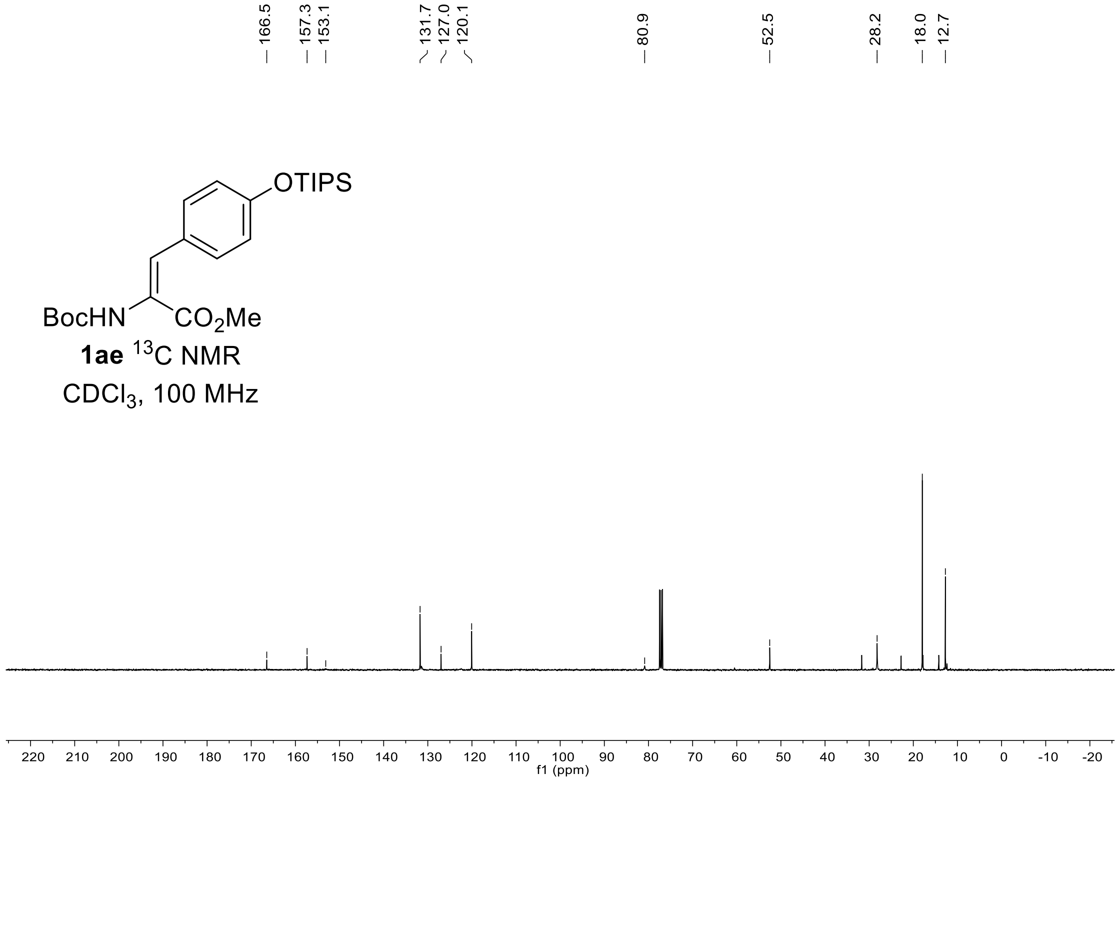
166.5
157.3
153.1
131.7
127.0
120.1
80.9
52.5
28.2
18.0
12.7
OTIPS
BocHN
CO2Me
1ae 13C NMR
CDCl3, 100 MHz
f1 (ppm)

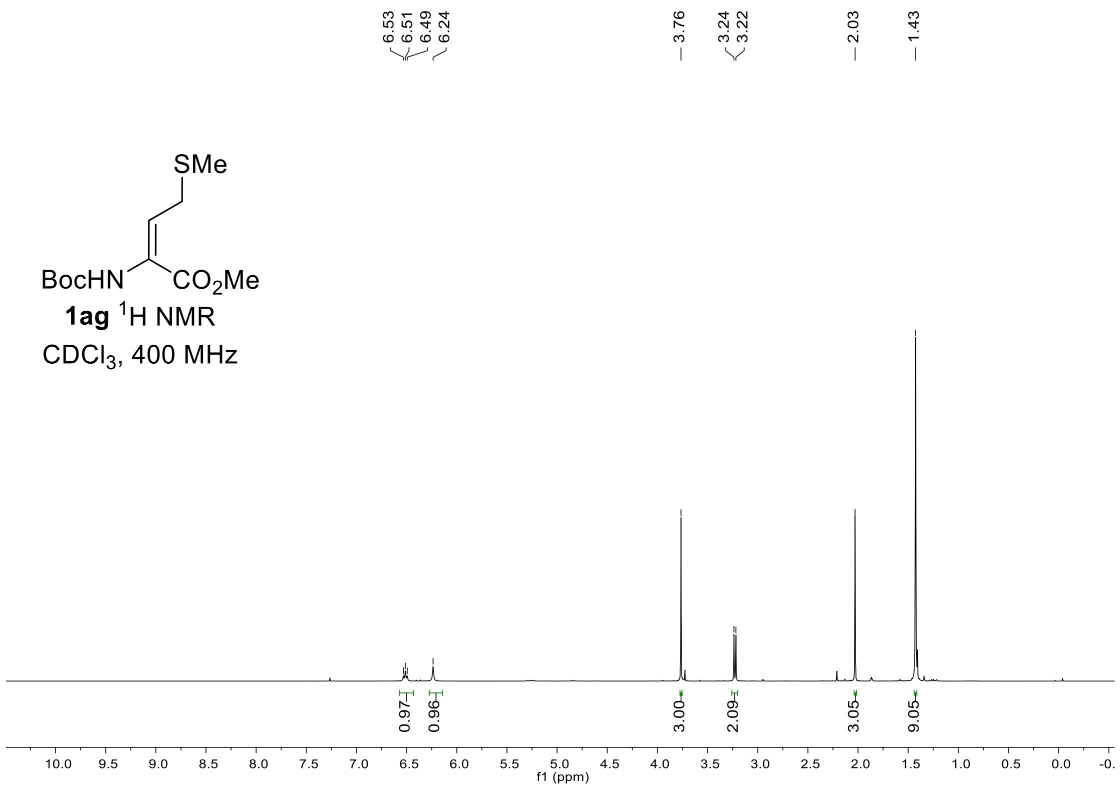

6.53
6.51
6.49
6.24
3.76
3.24
3.22
2.03
1.43
SMe
BocHN
CO2Me
1ag 1H NMR
CDCl3, 400 MHz
0.97
0.96
3.00
2.09
3.05
9.05
10.0 9.5 9.0 8.5 8.0 7.5 7.0 6.5 6.0 5.5 5.0 4.5 4.0 3.5 3.0 2.5 2.0 1.5 1.0 0.5 0.0 -0.
f1 (ppm)

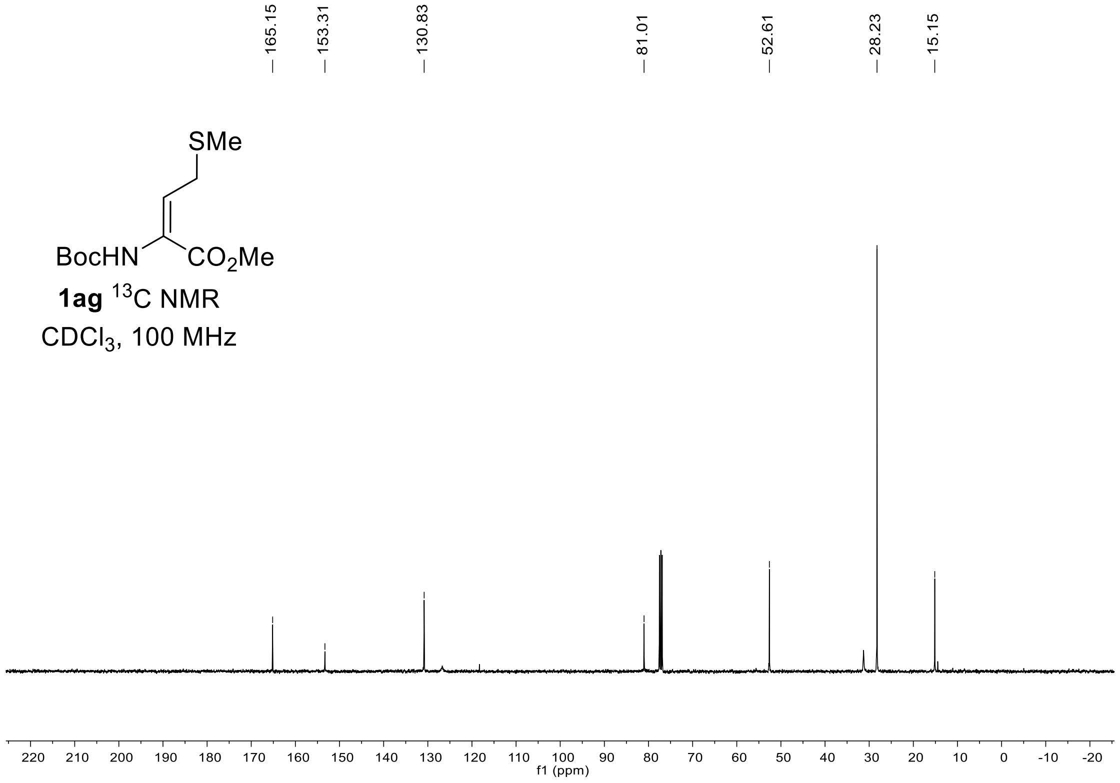

165.15
153.31
130.83
81.01
52.61
28.23
15.15
SMe
BocHN
CO2Me
1ag 13C NMR
CDCl3, 100 MHz
220 210 200 190 180 170 160 150 140 130 120 110 100 90 80 70 60 50 40 30 20 10 0 -10 -20
f1 (ppm)

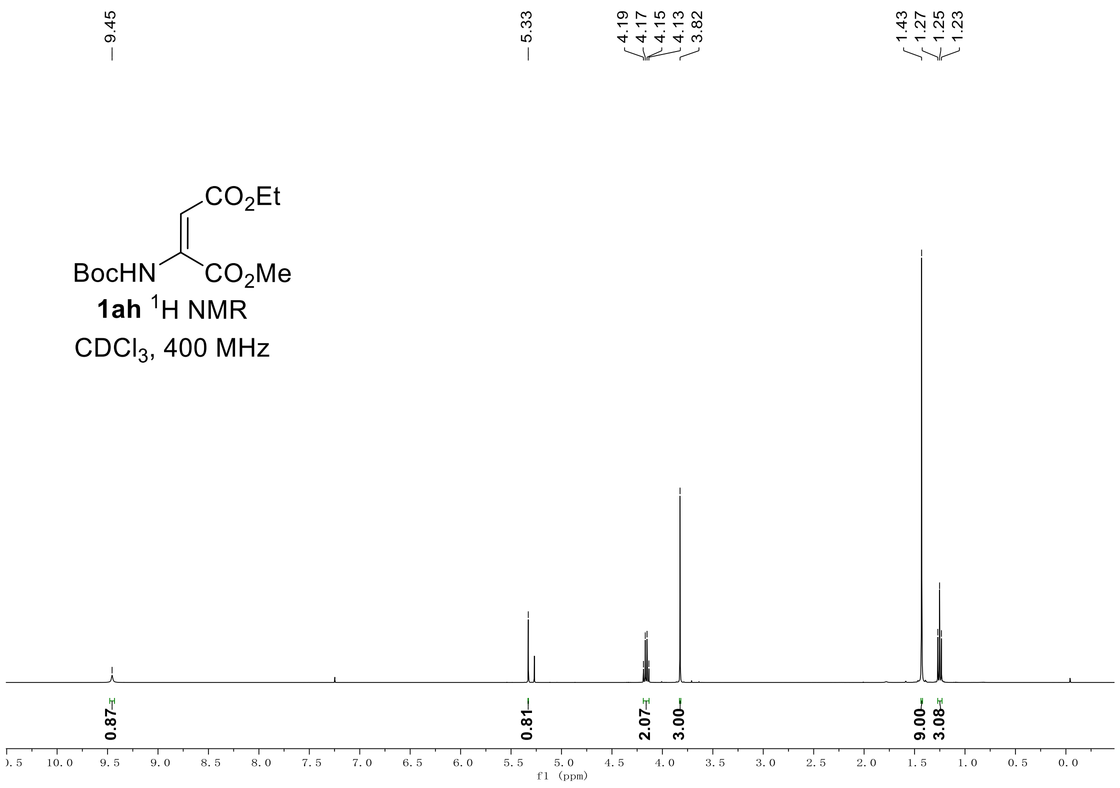

9.45
5.33
4.19
4.17
4.15
4.13
3.82
1.43
1.27
1.25
1.23
CO2Et
BocHN
CO2Me
1ah 1H NMR
CDCl3, 400 MHz
0.87
0.81
2.07
3.00
9.00
3.08
f1 (ppm)

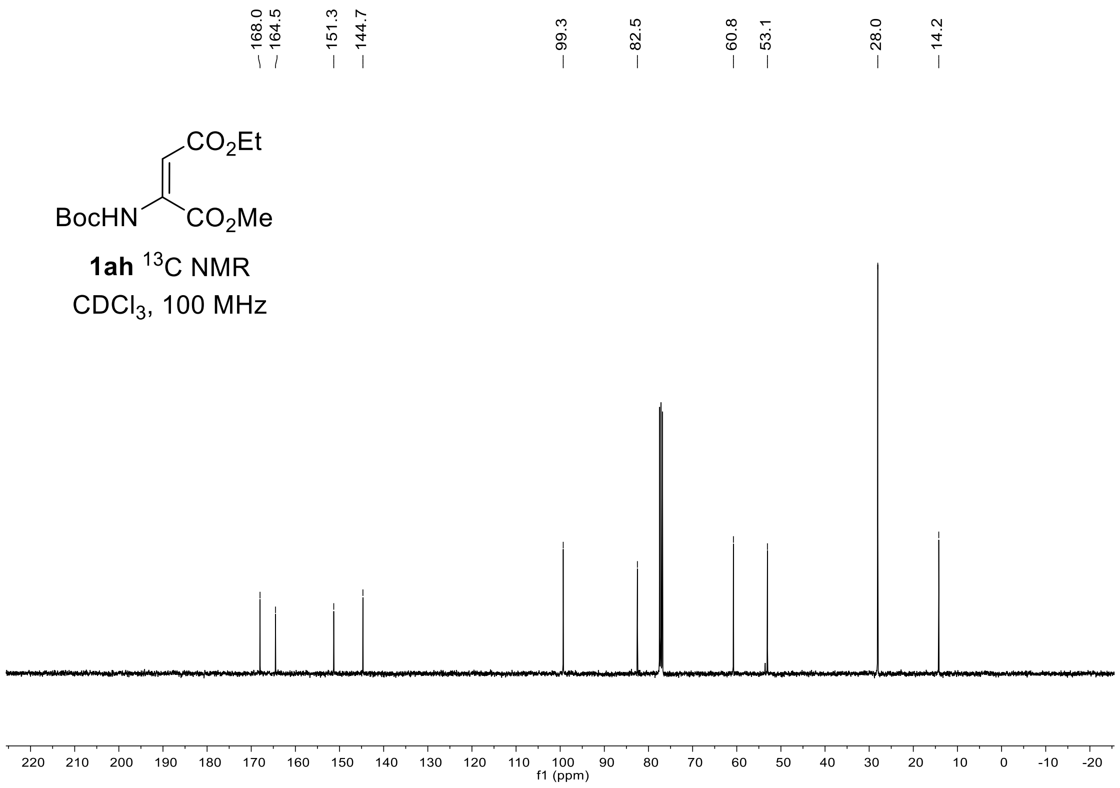

168.0
164.5
151.3
144.7
99.3
82.5
60.8
53.1
28.0
14.2
CO2Et
BocHN
CO2Me
1ah 13C NMR
CDCl3, 100 MHz
f1 (ppm)

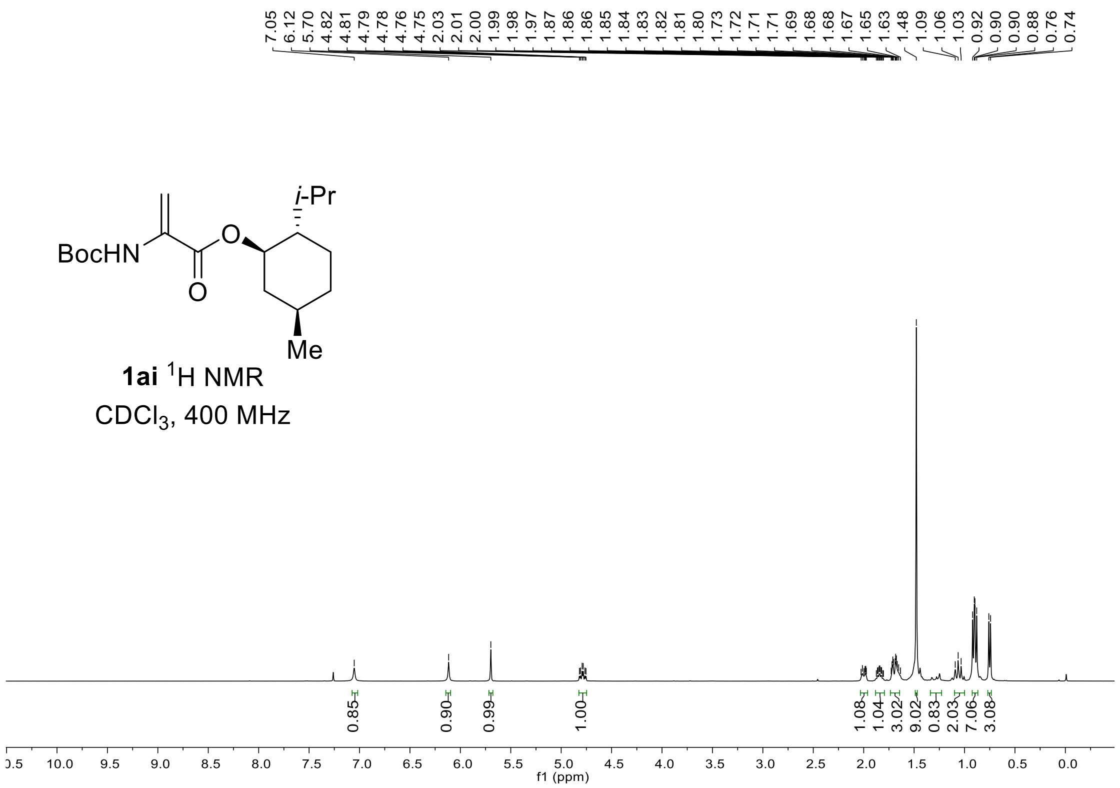
7.05 6.12 5.70 4.82 4.81 4.79 4.78 4.76 4.75 2.03 2.01 2.00 1.99 1.98 1.97 1.87 1.86 1.86 1.85 1.84 1.83 1.82 1.81 1.80 1.73 1.72 1.71 1.71 1.69 1.68 1.68 1.67 1.65 1.63 1.48 1.09 1.06 1.03 0.92 0.90 0.90 0.88 0.76 0.74
i-Pr
BocHN
O
O
Me
1ai 1H NMR
CDCl3, 400 MHz
0.85 0.90 0.99 1.00 1.08 1.04 3.02 9.02 0.83 2.03 7.06 3.08
f1 (ppm)

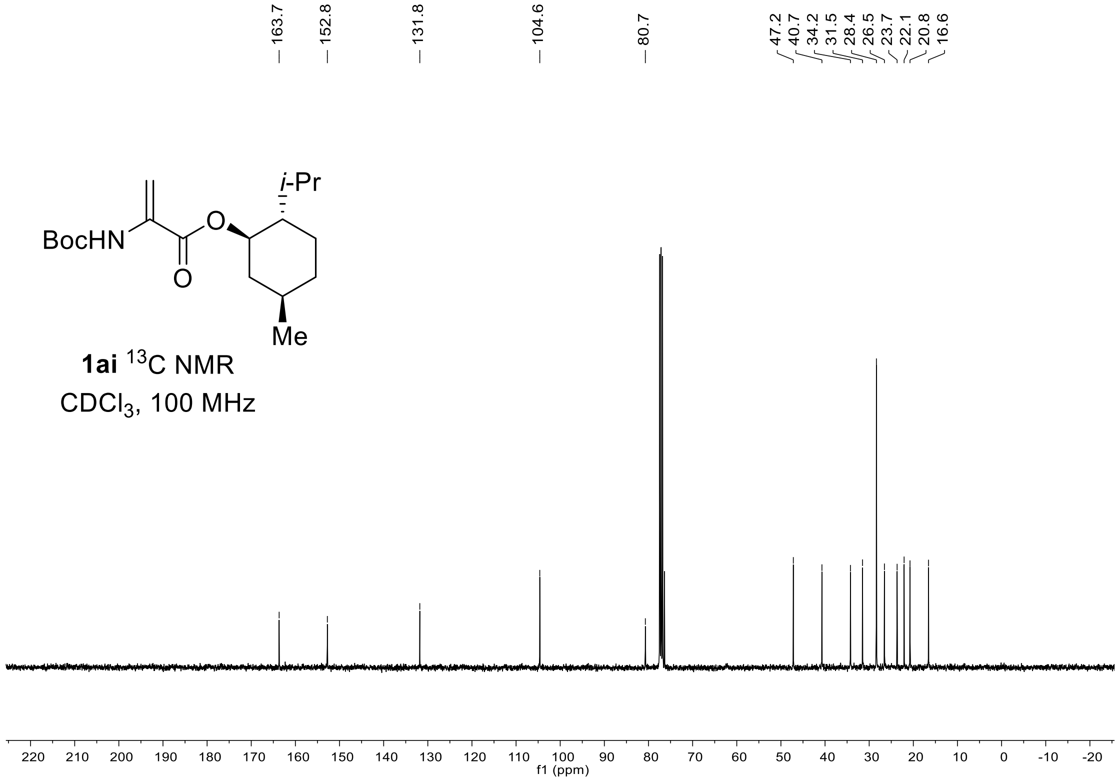
163.7 152.8 131.8 104.6 80.7 47.2 40.7 34.2 31.5 28.4 26.5 23.7 22.1 20.8 16.6
i-Pr
BocHN
O
O
Me
1ai 13C NMR
CDCl3, 100 MHz
f1 (ppm)

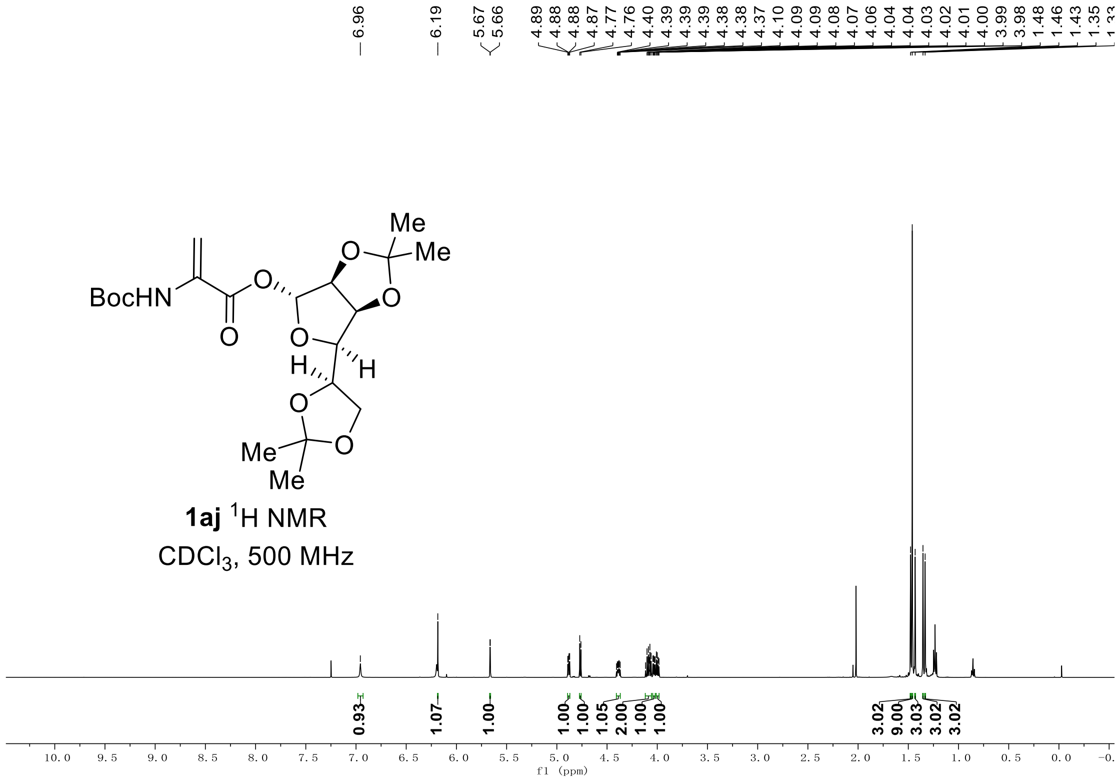

6.96
6.19
5.67
5.66
4.89
4.88
4.88
4.87
4.77
4.76
4.40
4.39
4.39
4.39
4.38
4.38
4.37
4.10
4.09
4.09
4.08
4.07
4.06
4.04
4.04
4.03
4.02
4.01
4.00
3.99
3.98
1.48
1.46
1.43
1.35
Me
O
Me
BocHN
O
O
O
O
O
H
H
O
Me
O
Me
1aj ^{1}H NMR
$CDCl_3$, 500 MHz
0.93
1.07
1.00
1.00
1.00
1.05
2.00
1.00
1.00
3.02
9.00
3.03
3.02
3.02
10.0 9.5 9.0 8.5 8.0 7.5 7.0 6.5 6.0 5.5 5.0 4.5 4.0 3.5 3.0 2.5 2.0 1.5 1.0 0.5 0.0
f1 (ppm)

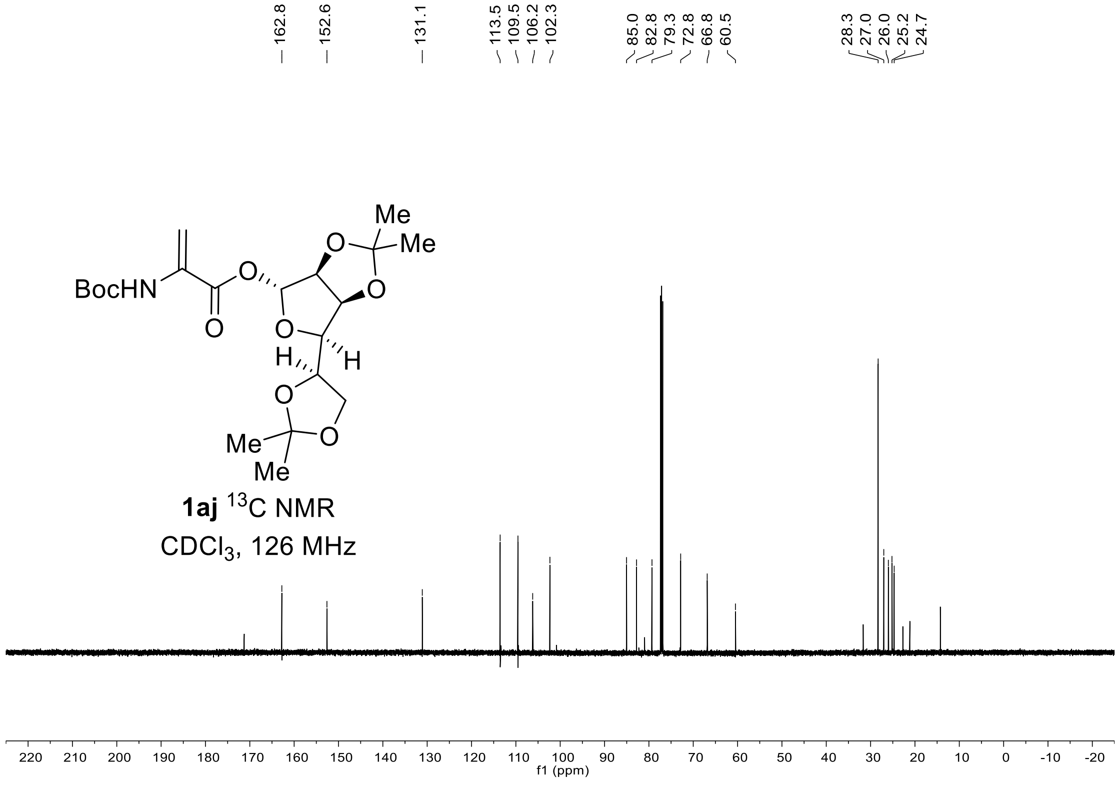

162.8
152.6
131.1
113.5
109.5
106.2
102.3
85.0
82.8
79.3
72.8
66.8
60.5
28.3
27.0
26.0
25.2
24.7
Me
O
Me
BocHN
O
O
O
O
O
H
H
O
Me
O
Me
1aj ^{13}C NMR
$CDCl_3$, 126 MHz
220 210 200 190 180 170 160 150 140 130 120 110 100 90 80 70 60 50 40 30 20 10 0 -10 -20
f1 (ppm)

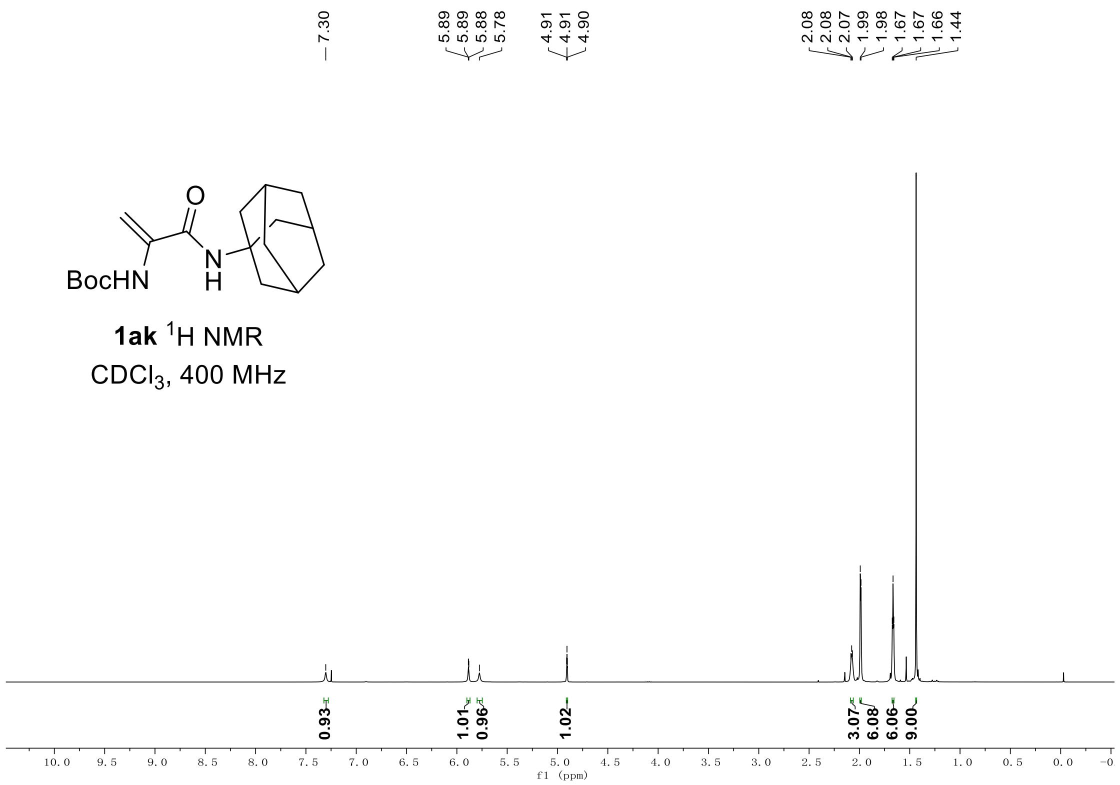

1ak ^{1}H NMR
$CDCl_3$, 400 MHz

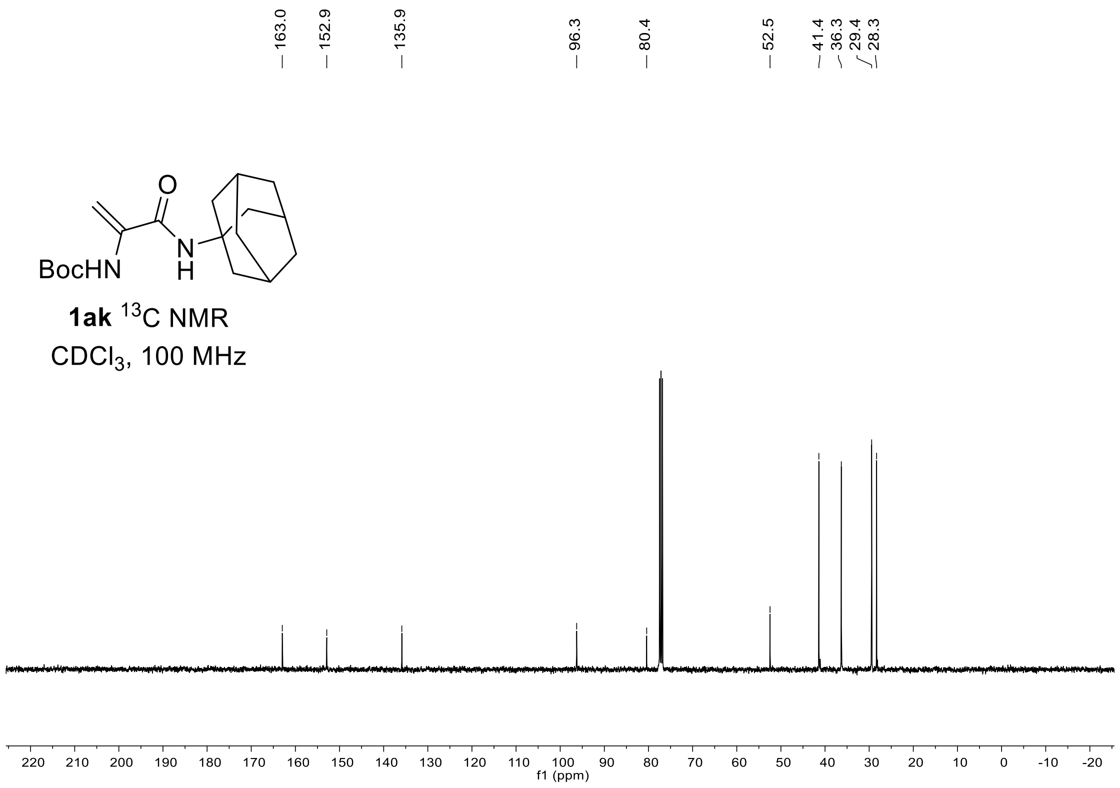

1ak ^{13}C NMR
$CDCl_3$, 100 MHz

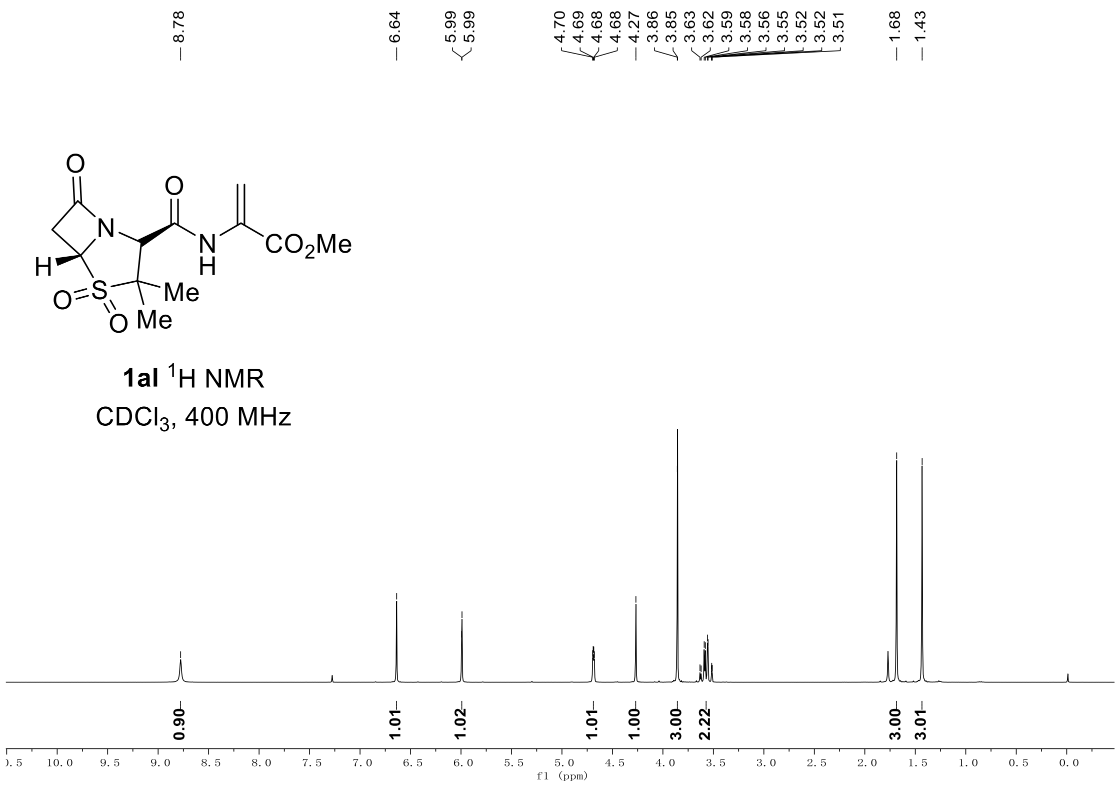

1al ^{1}H NMR
$CDCl_3$, 400 MHz

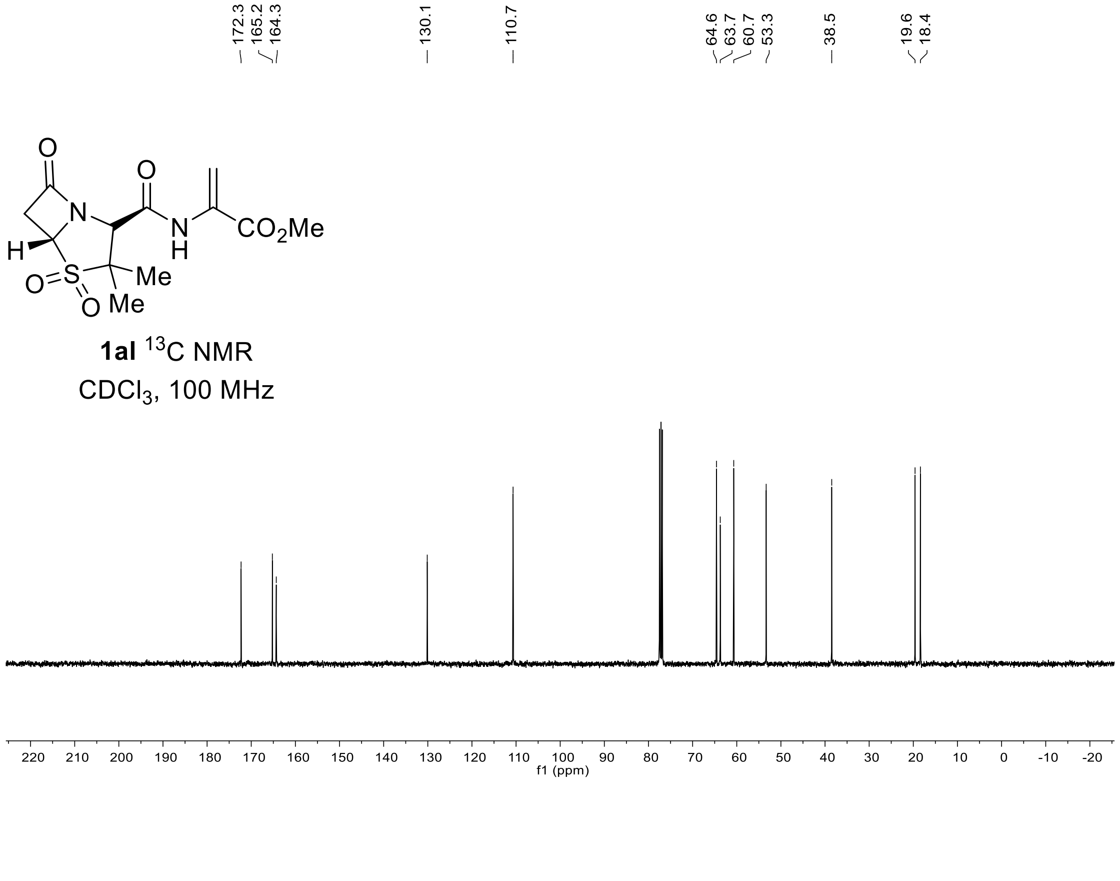

1al ^{13}C NMR
$CDCl_3$, 100 MHz

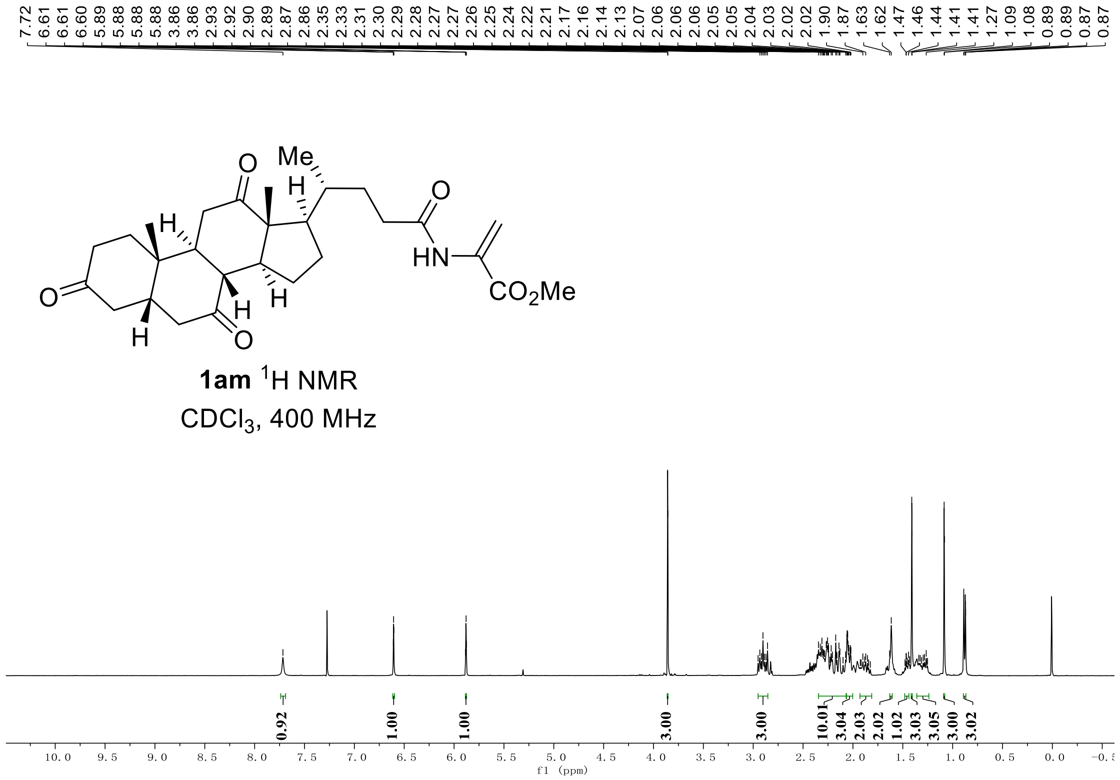
7.72 6.61 6.61 6.60 5.89 5.88 5.88 5.88 3.86 2.93 2.92 2.90 2.89 2.87 2.86 2.35 2.33 2.31 2.30 2.29 2.28 2.27 2.27 2.26 2.25 2.24 2.22 2.21 2.17 2.16 2.14 2.13 2.07 2.06 2.06 2.06 2.05 2.05 2.04 2.03 2.02 2.02 1.90 1.87 1.63 1.62 1.47 1.46 1.44 1.41 1.41 1.27 1.09 1.08 0.89 0.89 0.87 0.87
Me
H
O
HN
CO2Me
1am 1H NMR
CDCl3, 400 MHz
0.92 1.00 1.00 3.00 3.00 10.01 3.04 2.03 2.02 1.02 3.03 3.05 3.00 3.02
f1 (ppm)
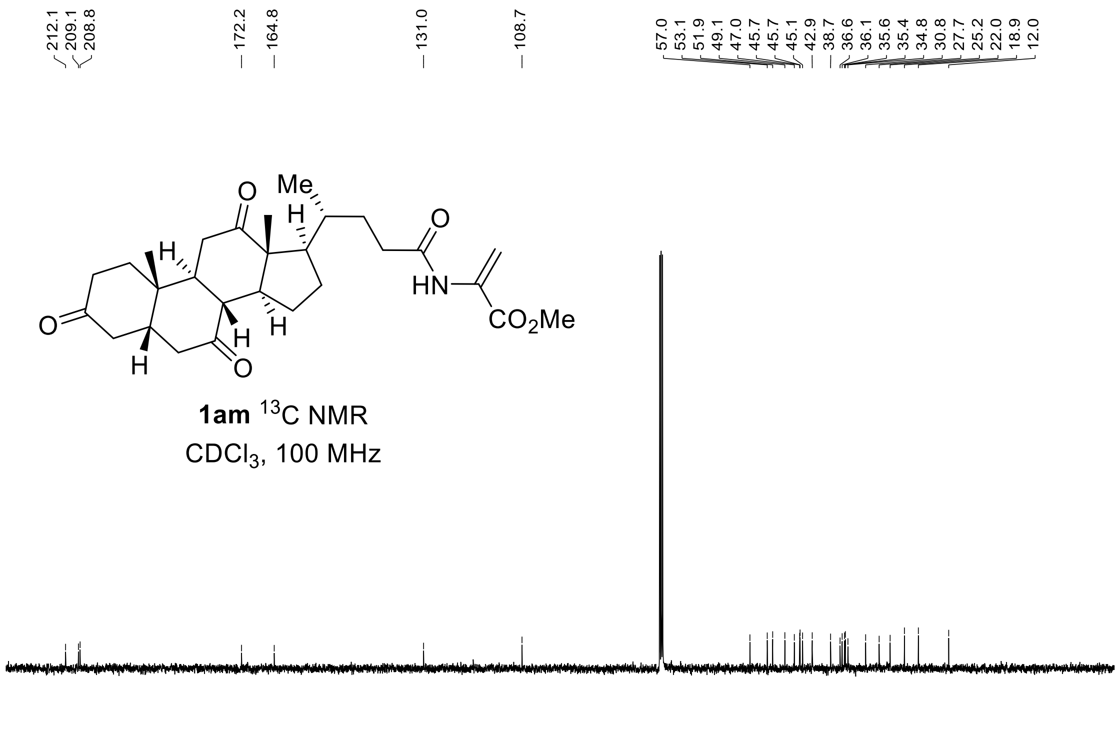
212.1 209.1 208.8 172.2 164.8 131.0 108.7 57.0 53.1 51.9 49.1 47.0 45.7 45.7 45.1 42.9 38.7 36.6 36.1 35.6 35.4 34.8 30.8 27.7 25.2 22.0 18.9 12.0
Me
H
O
HN
CO2Me
1am 13C NMR
CDCl3, 100 MHz
f1 (ppm)

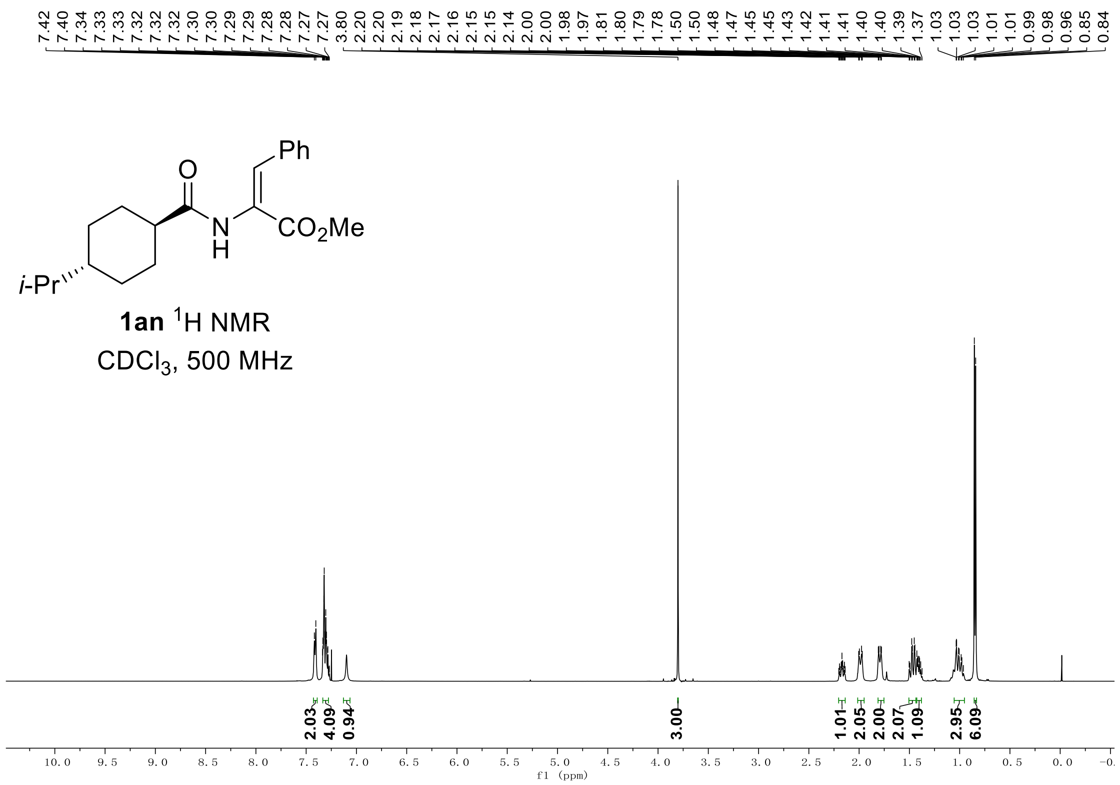
7.42 7.40 7.34 7.33 7.32 7.32 7.32 7.30 7.30 7.29 7.29 7.28 7.28 7.27 7.27 3.80 2.20 2.20 2.19 2.18 2.17 2.16 2.15 2.15 2.14 2.00 2.00 1.98 1.97 1.81 1.80 1.79 1.78 1.50 1.50 1.48 1.47 1.45 1.45 1.43 1.42 1.41 1.41 1.40 1.40 1.39 1.37 1.03 1.03 1.03 1.01 1.01 0.99 0.98 0.96 0.85 0.84
Ph
O
N
H
CO2Me
i-Pr
1an 1H NMR
CDCl3, 500 MHz
2.03 4.09 0.94 3.00 1.01 2.05 2.00 2.07 1.09 2.95 6.09
f1 (ppm)

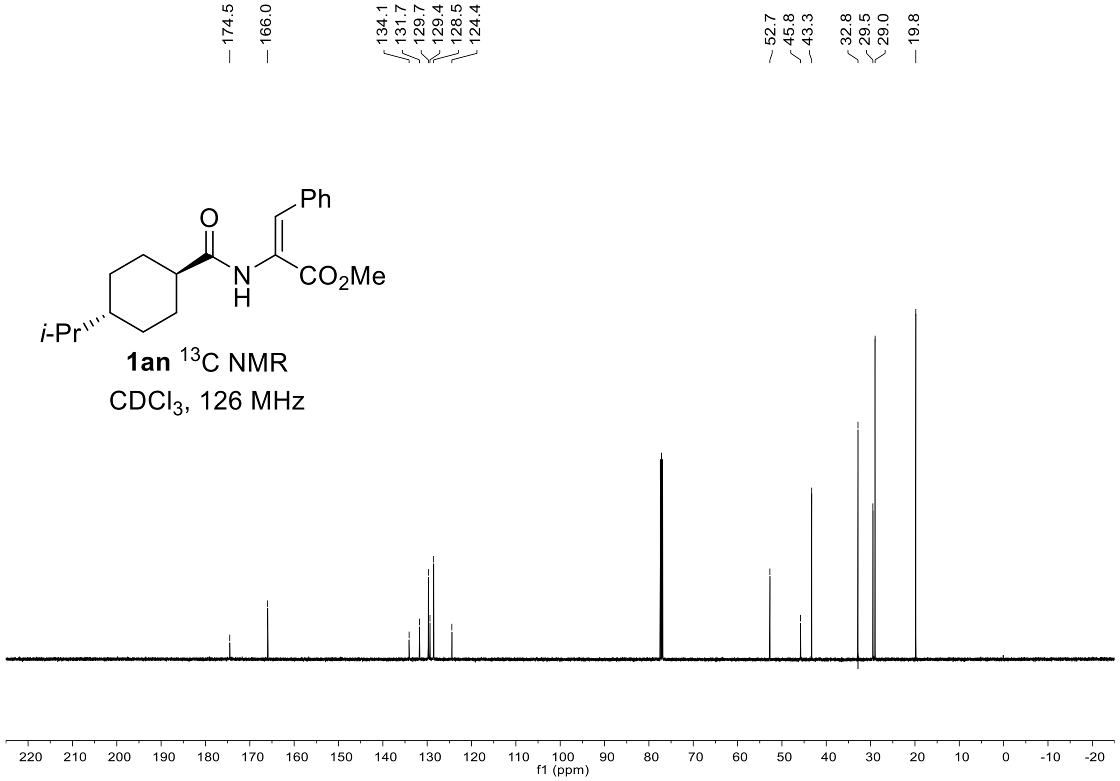
174.5 166.0 134.1 131.7 129.7 129.4 128.5 124.4 52.7 45.8 43.3 32.8 29.5 29.0 19.8
Ph
O
N
H
CO2Me
i-Pr
1an 13C NMR
CDCl3, 126 MHz
f1 (ppm)

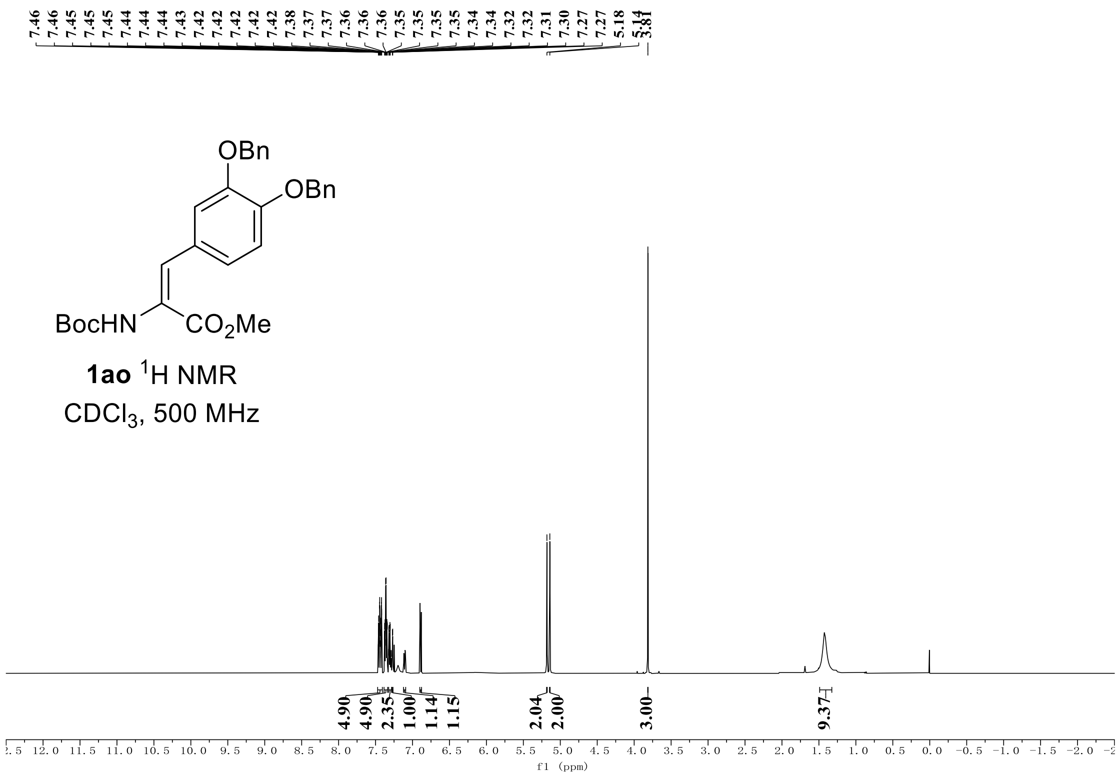
7.46 7.46 7.45 7.45 7.44 7.44 7.44 7.43 7.42 7.42 7.42 7.42 7.42 7.38 7.37 7.37 7.36 7.36 7.36 7.35 7.35 7.35 7.35 7.35 7.34 7.34 7.32 7.32 7.31 7.30 7.27 7.27 5.18 5.14 3.81
OBn
OBn
BocHN
CO2Me
1ao 1H NMR
CDCl3, 500 MHz
4.90 4.90 2.35 1.00 1.14 1.15 2.04 2.00 3.00 9.37
f1 (ppm)

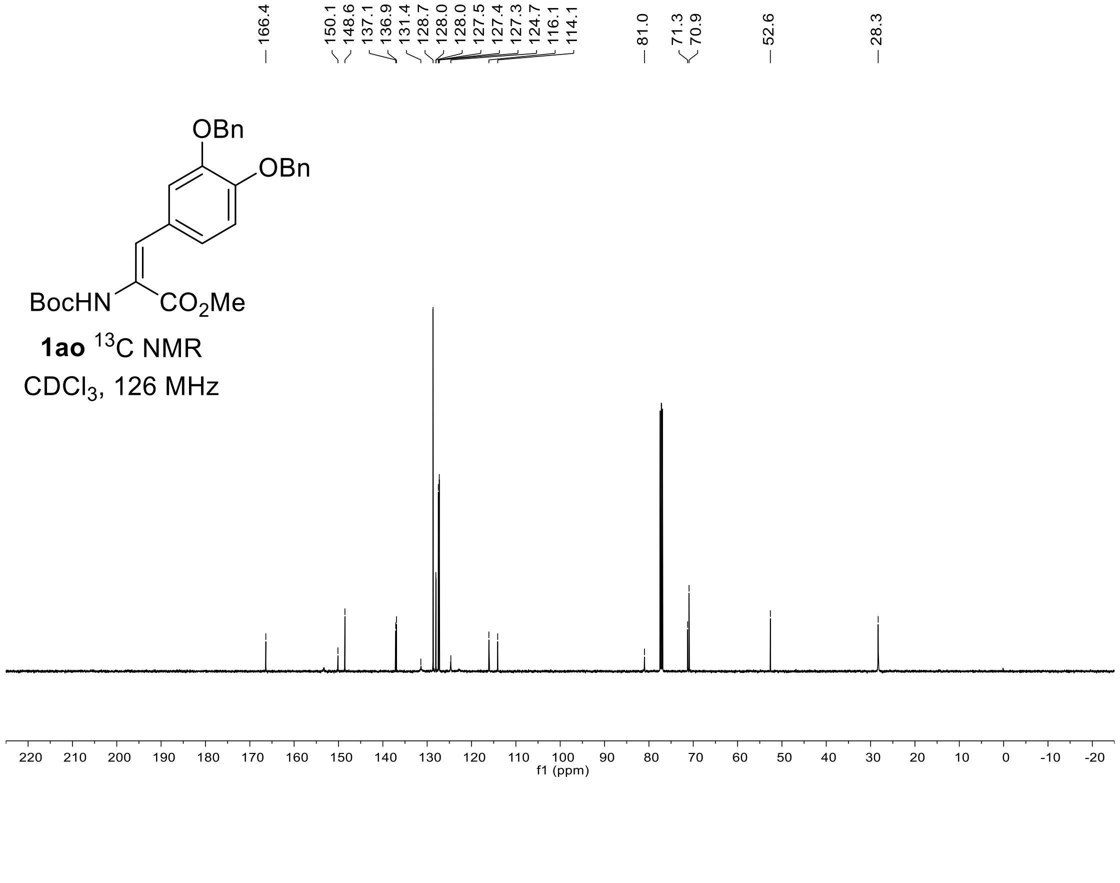
166.4 150.1 148.6 137.1 136.9 131.4 128.7 128.0 128.0 127.5 127.4 127.3 124.7 116.1 114.1 81.0 71.3 70.9 52.6 28.3
OBn
OBn
BocHN
CO2Me
1ao 13C NMR
CDCl3, 126 MHz
220 210 200 190 180 170 160 150 140 130 120 110 100 90 80 70 60 50 40 30 20 10 0 -10 -20
f1 (ppm)

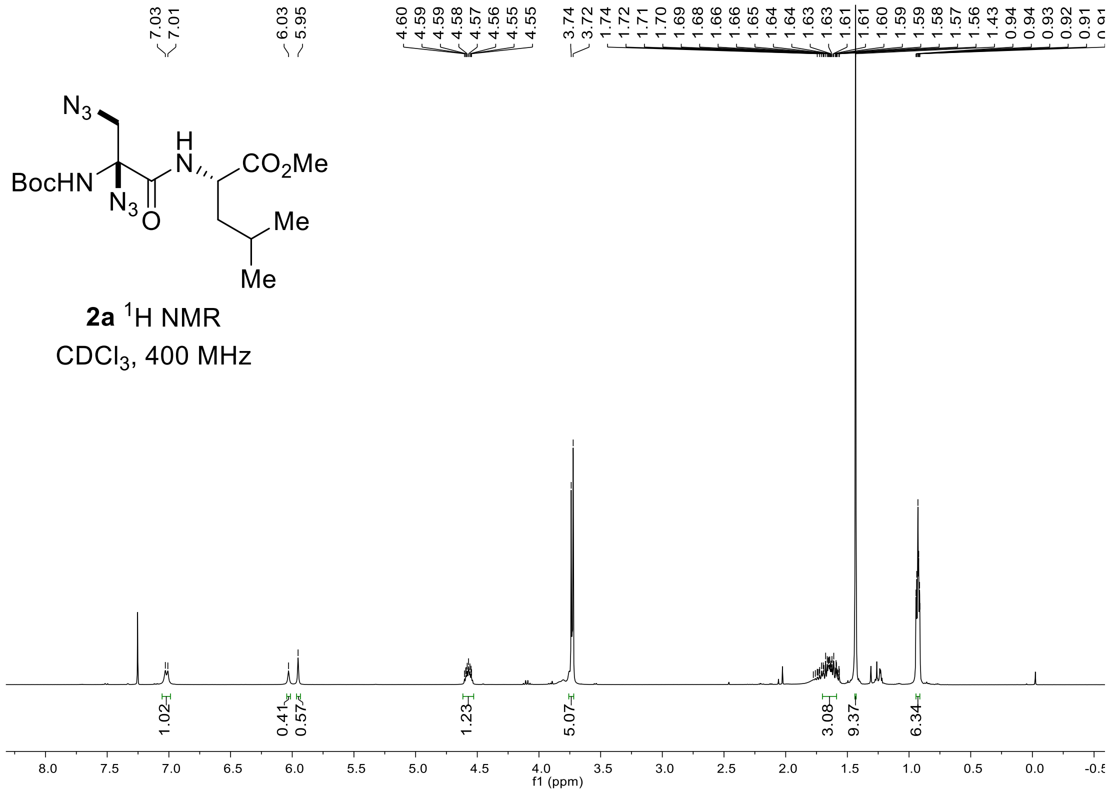

2a ^{1}H NMR
$CDCl_3$, 400 MHz

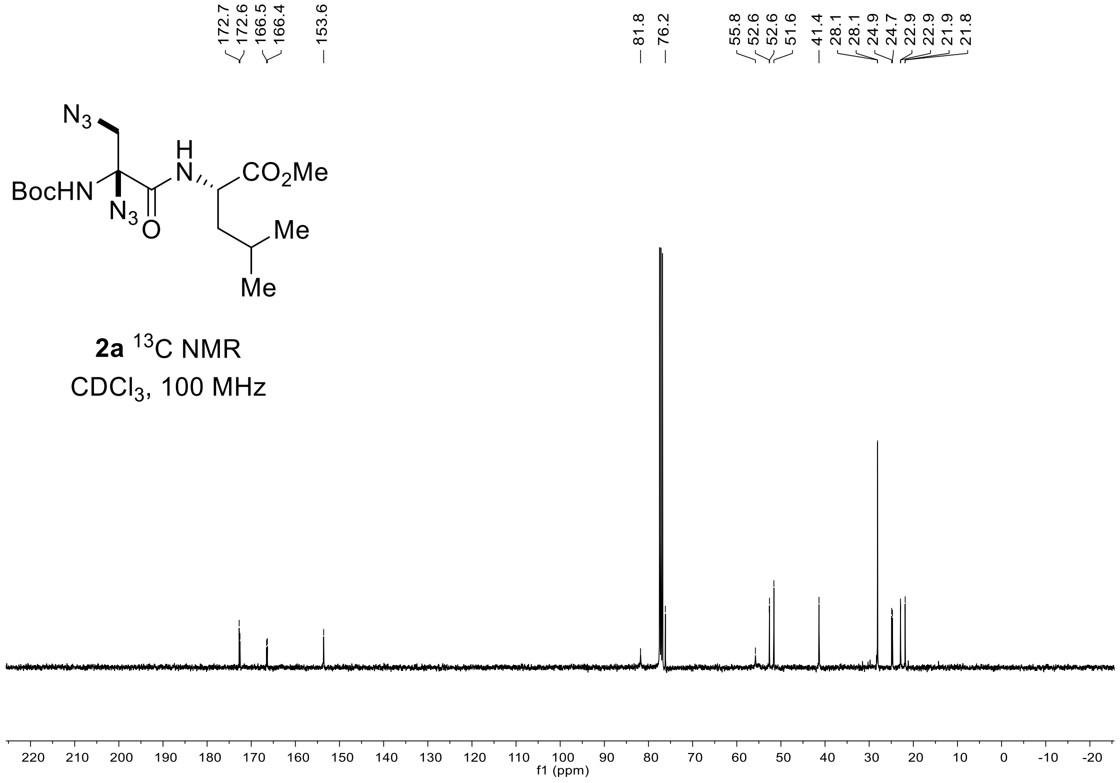

2a ^{13}C NMR
$CDCl_3$, 100 MHz

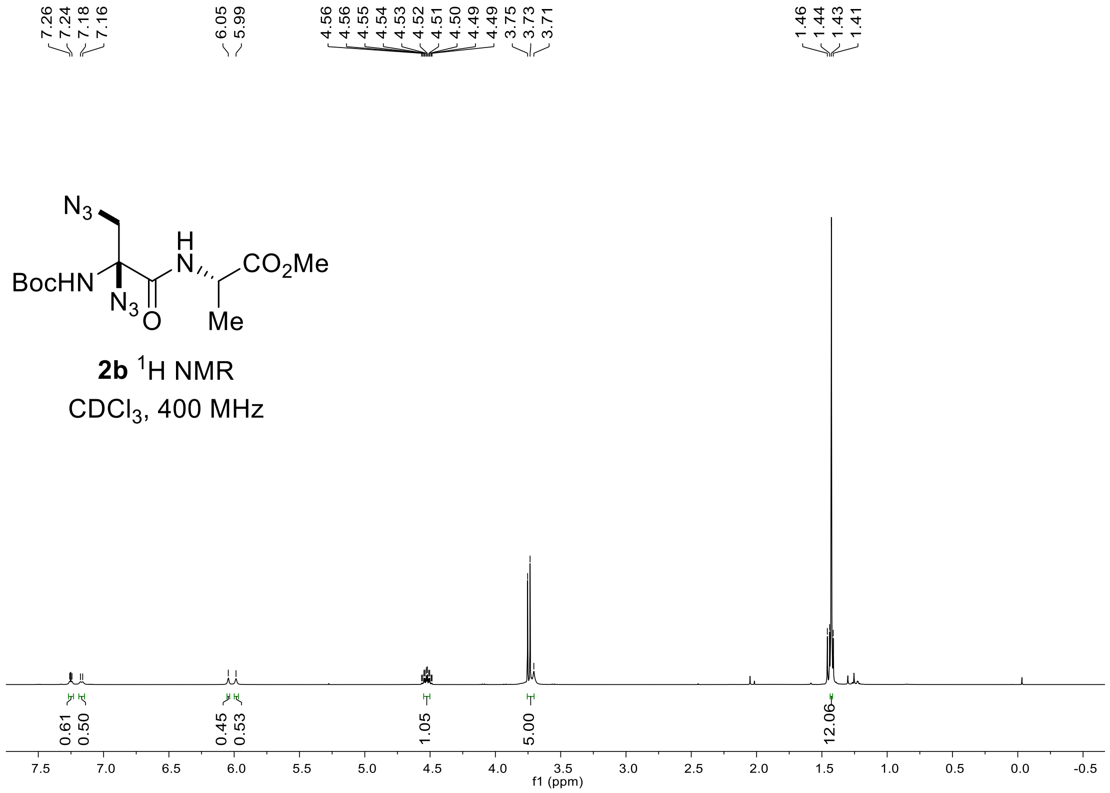
7.26
7.24
7.18
7.16
6.05
5.99
4.56
4.56
4.55
4.54
4.53
4.52
4.51
4.50
4.49
4.49
3.75
3.73
3.71
1.46
1.44
1.43
1.41
N3
H
N
CO2Me
BocHN
N3
O
Me
2b 1H NMR
CDCl3, 400 MHz
0.61
0.50
0.45
0.53
1.05
5.00
12.06
7.5 7.0 6.5 6.0 5.5 5.0 4.5 4.0 3.5 3.0 2.5 2.0 1.5 1.0 0.5 0.0 -0.5
f1 (ppm)

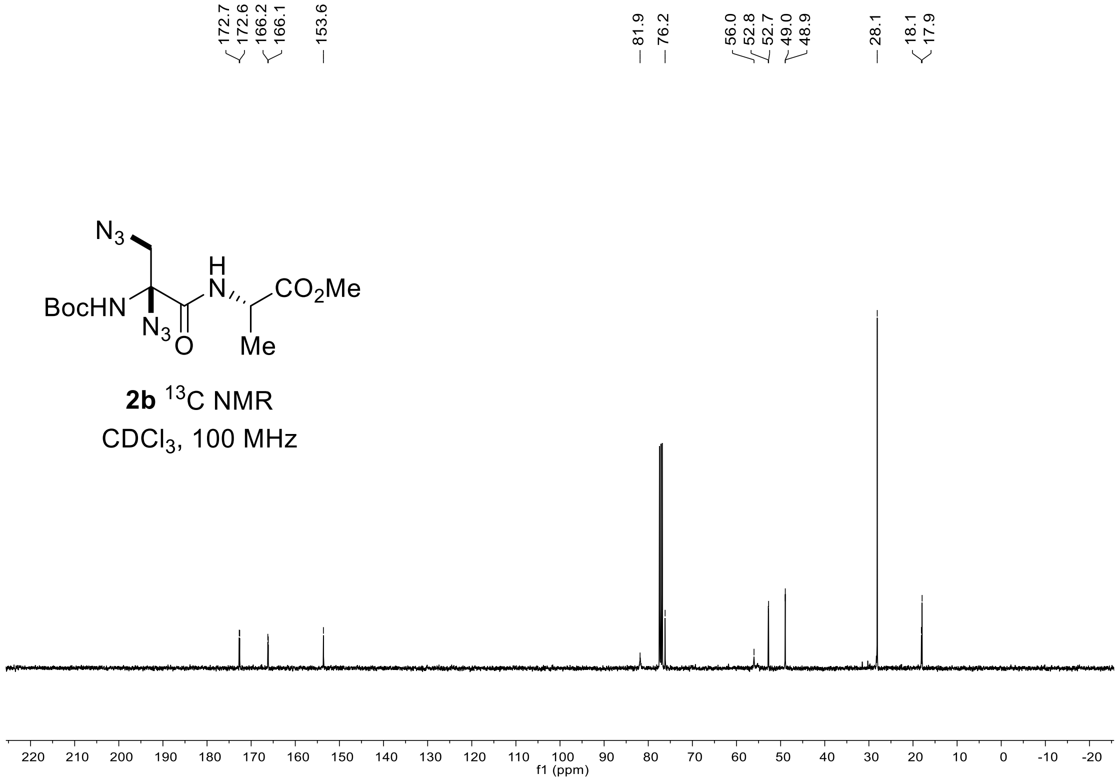
172.7
172.6
166.2
166.1
153.6
81.9
76.2
56.0
52.8
52.7
49.0
48.9
28.1
18.1
17.9
N3
H
N
CO2Me
BocHN
N3
O
Me
2b 13C NMR
CDCl3, 100 MHz
220 210 200 190 180 170 160 150 140 130 120 110 100 90 80 70 60 50 40 30 20 10 0 -10 -20
f1 (ppm)

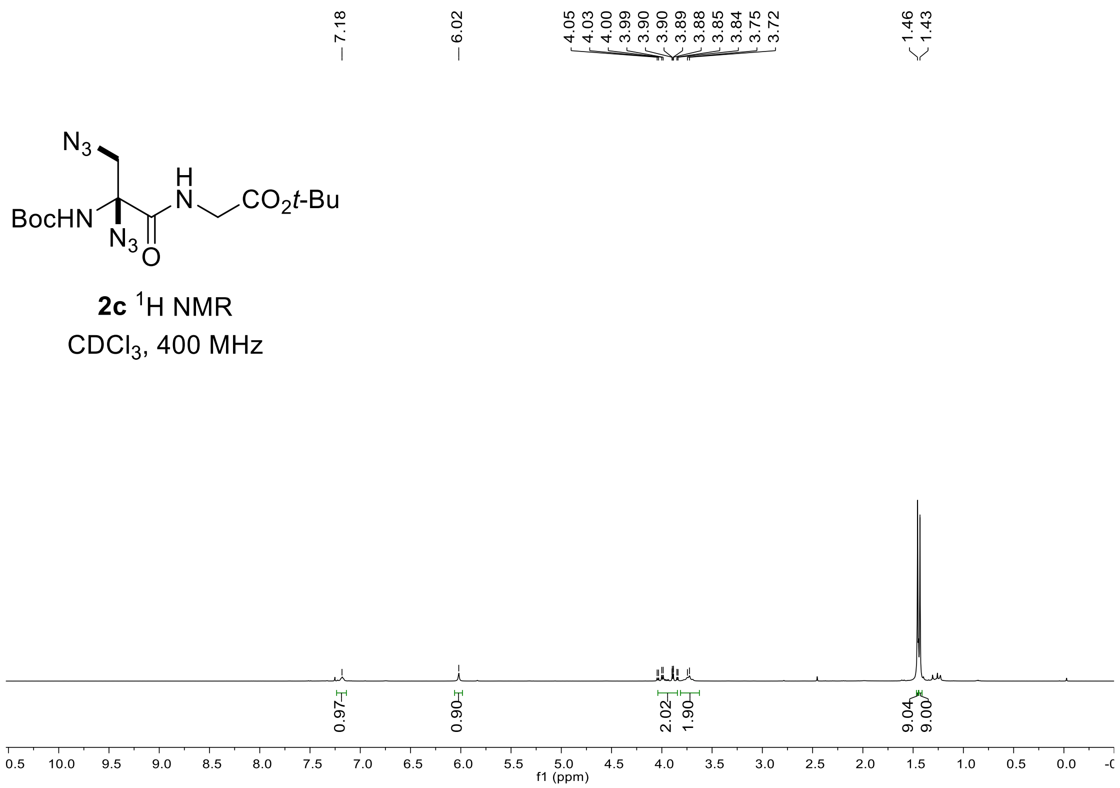
BocHN
N3
H
N
CO2t-Bu
O
2c 1H NMR
CDCl3, 400 MHz
7.18
6.02
4.05 4.03 4.00 3.99 3.90 3.90 3.89 3.88 3.85 3.84 3.75 3.72
1.46 1.43
0.97
0.90
2.02
1.90
9.04
9.00
f1 (ppm)

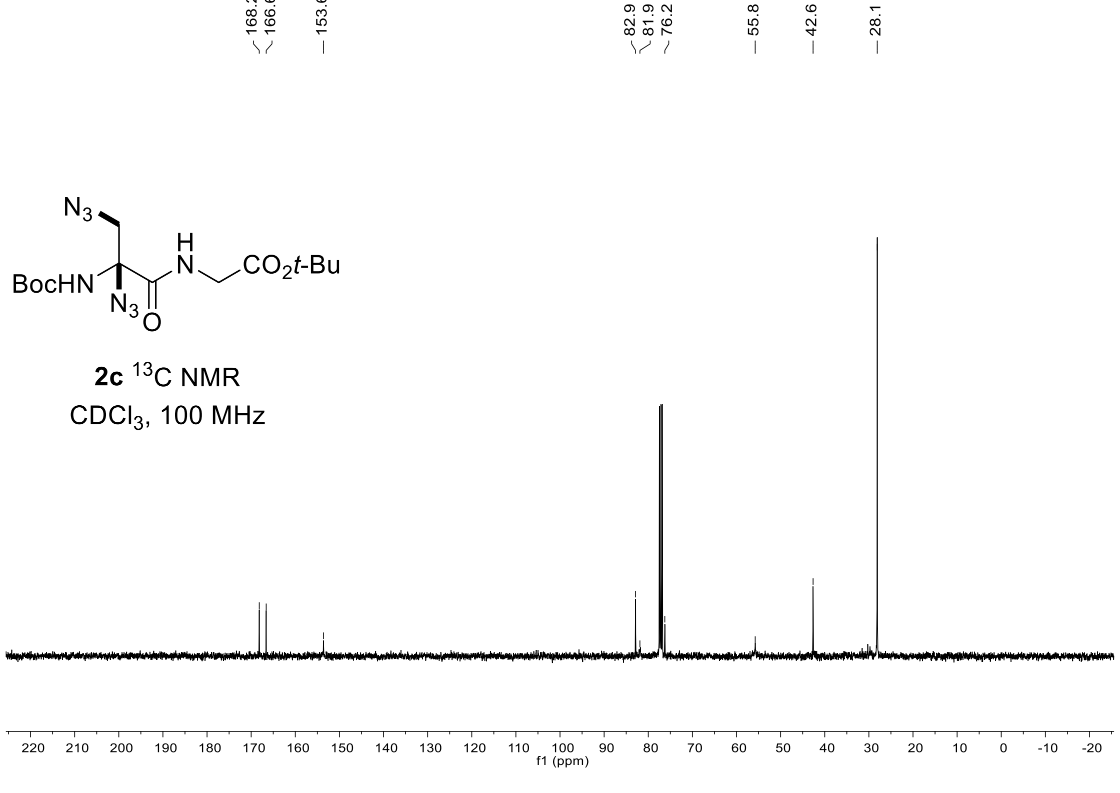
BocHN
N3
H
N
CO2t-Bu
O
2c 13C NMR
CDCl3, 100 MHz
168.2 166.6
153.6
82.9 81.9 76.2
55.8
42.6
28.1
f1 (ppm)

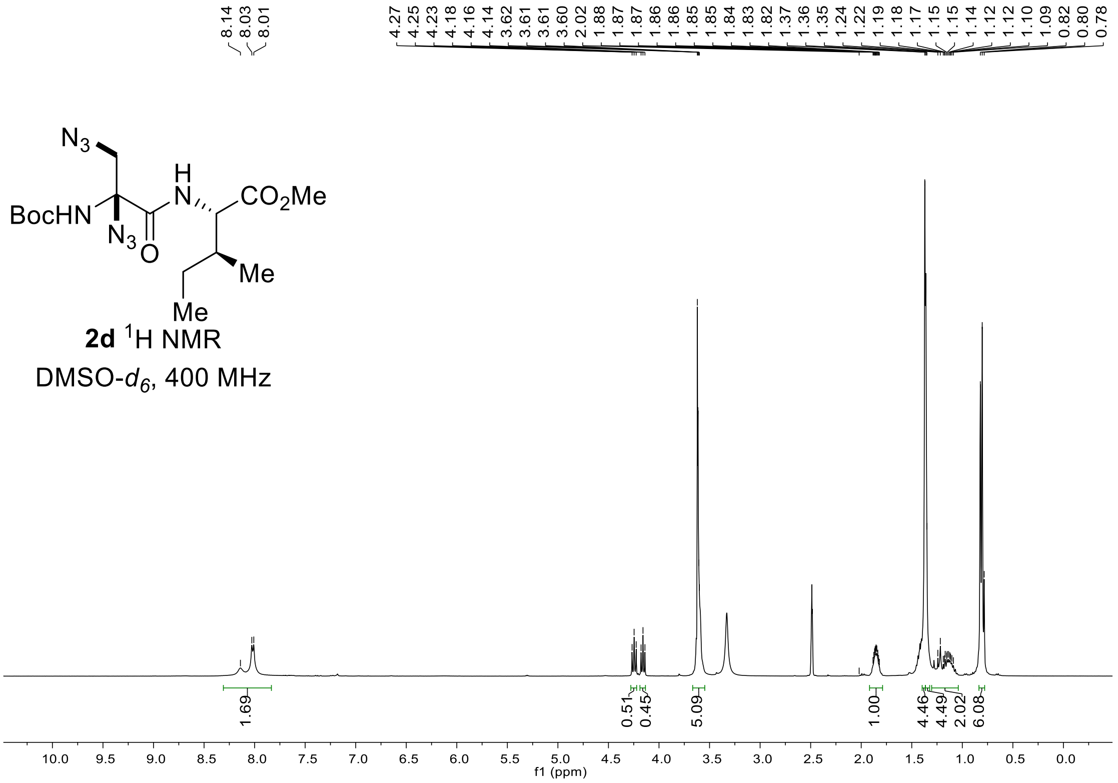
8.14
8.03
8.01
4.27 4.25 4.23 4.18 4.16 4.14 3.62 3.61 3.60 2.02 1.88 1.87 1.87 1.86 1.86 1.85 1.85 1.84 1.83 1.82 1.37 1.36 1.35 1.24 1.22 1.19 1.18 1.17 1.15 1.15 1.14 1.12 1.12 1.10 1.09 0.82 0.80 0.78
N_3
H
N
BocHN
N_3
O
CO_2Me
Me
Me
2d ^{1}H NMR
DMSO-d_6, 400 MHz
1.69
0.51
0.45
5.09
1.00
4.46
4.49
2.02
6.08
f1 (ppm)

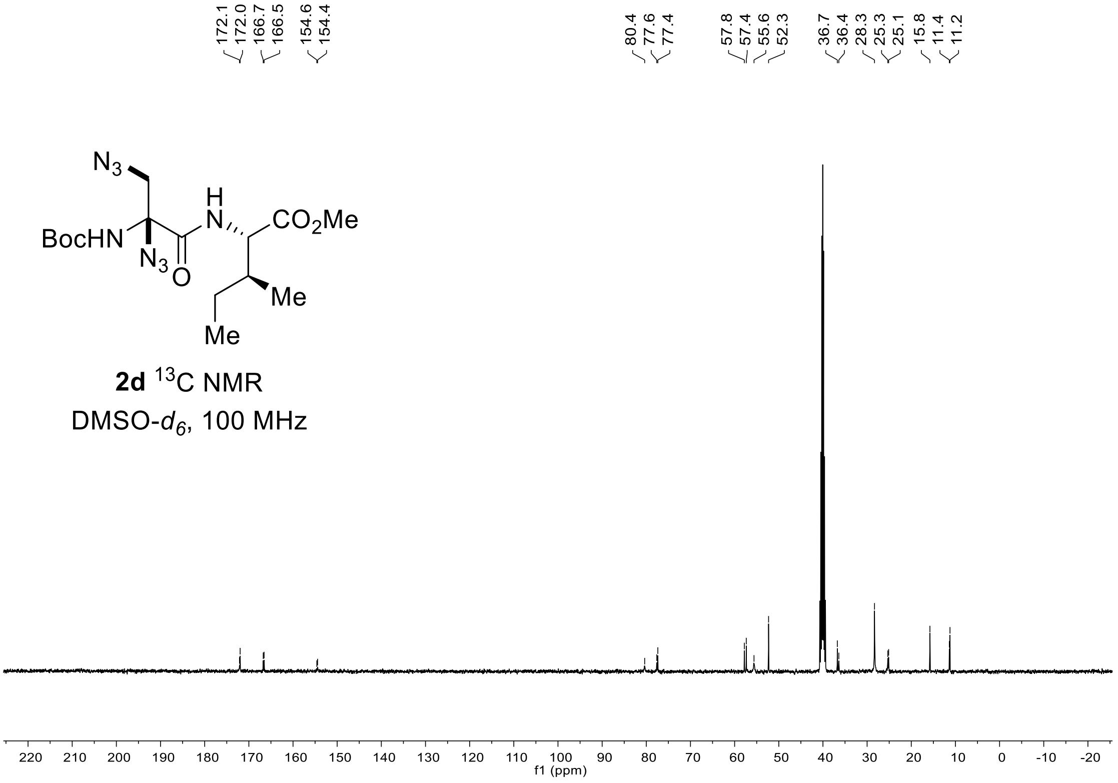
172.1 172.0 166.7 166.5 154.6 154.4
80.4 77.6 77.4
57.8 57.4 55.6 52.3
36.7 36.4 28.3 25.3 25.1 15.8 11.4 11.2
N_3
H
N
BocHN
N_3
O
CO_2Me
Me
Me
2d ^{13}C NMR
DMSO-d_6, 100 MHz
f1 (ppm)

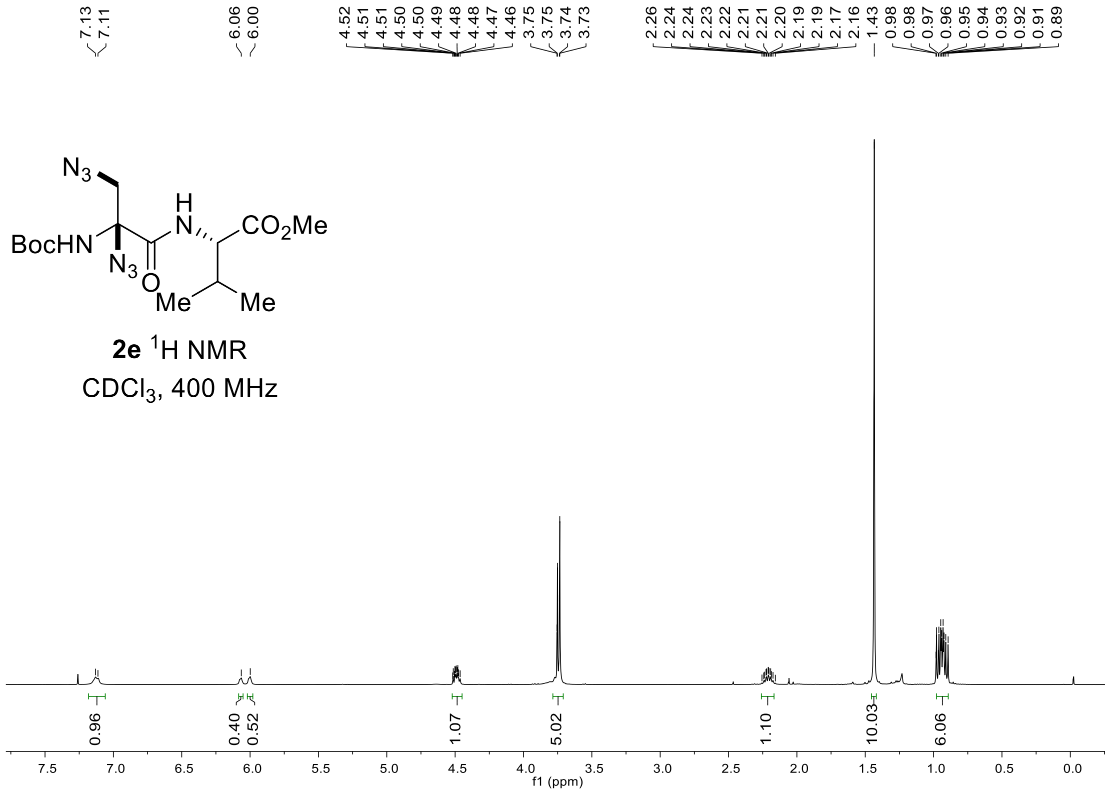
2e ^{1}H NMR
$CDCl_3$, 400 MHz
f1 (ppm)

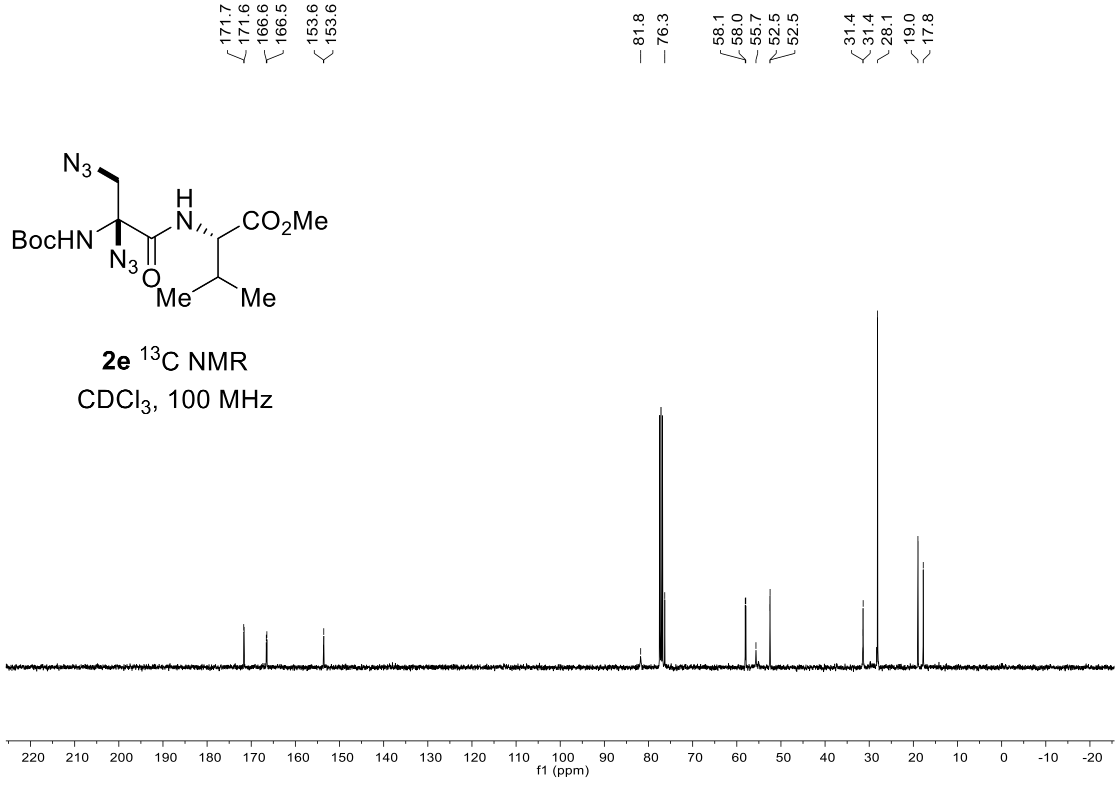
2e ^{13}C NMR
$CDCl_3$, 100 MHz
f1 (ppm)

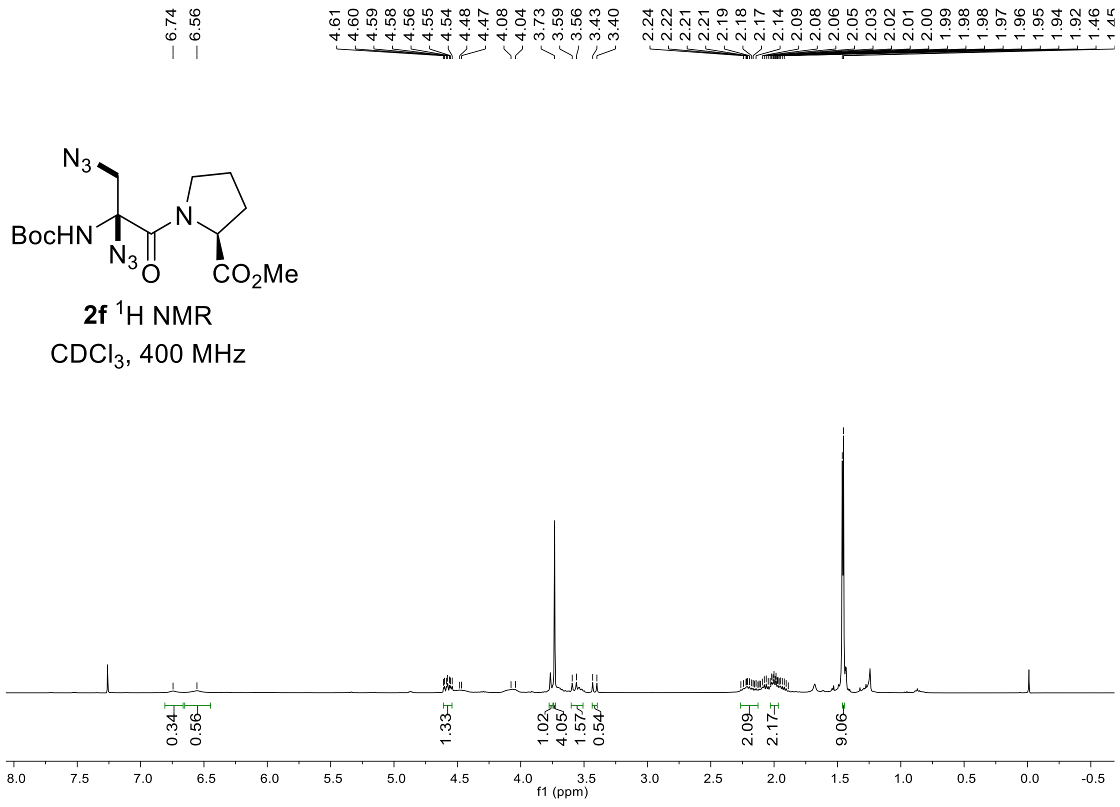
N3
BocHN
N3
O
N
CO2Me
2f 1H NMR
CDCl3, 400 MHz
f1 (ppm)

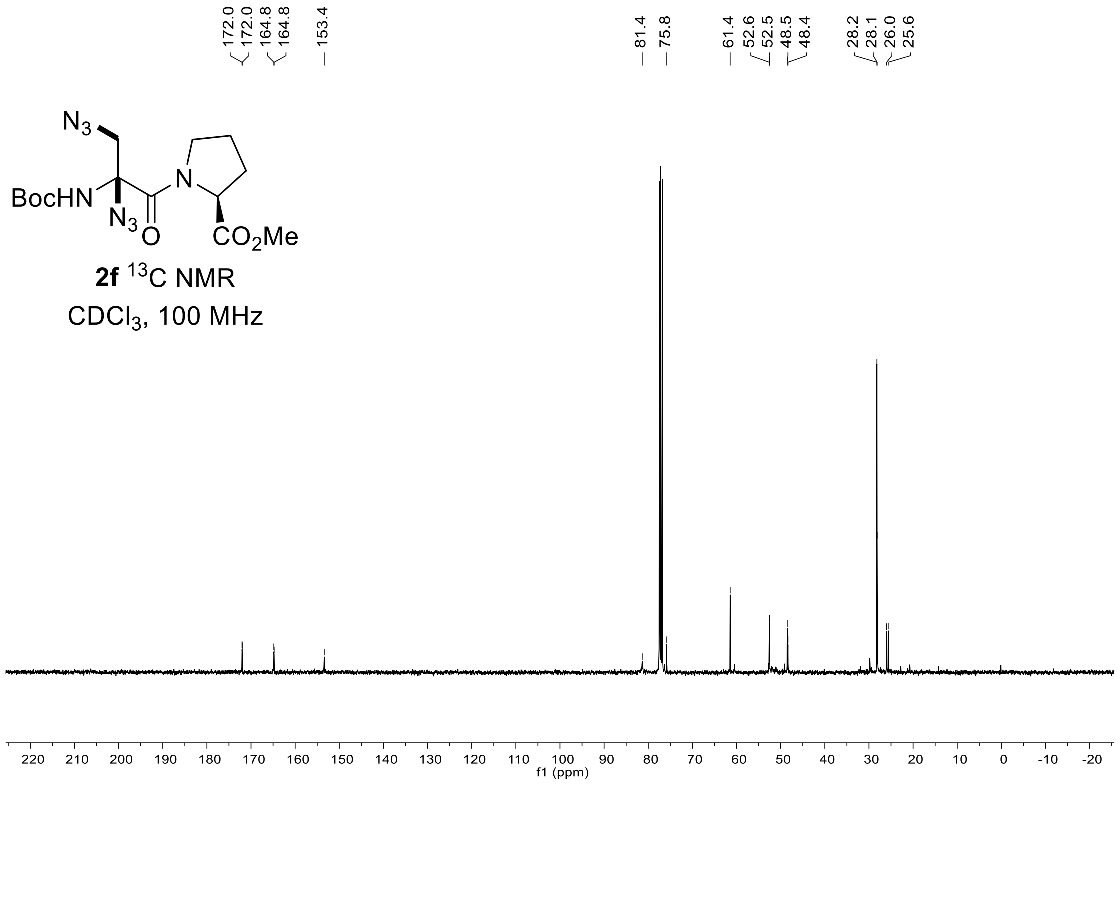
N3
BocHN
N3
O
N
CO2Me
2f 13C NMR
CDCl3, 100 MHz
f1 (ppm)

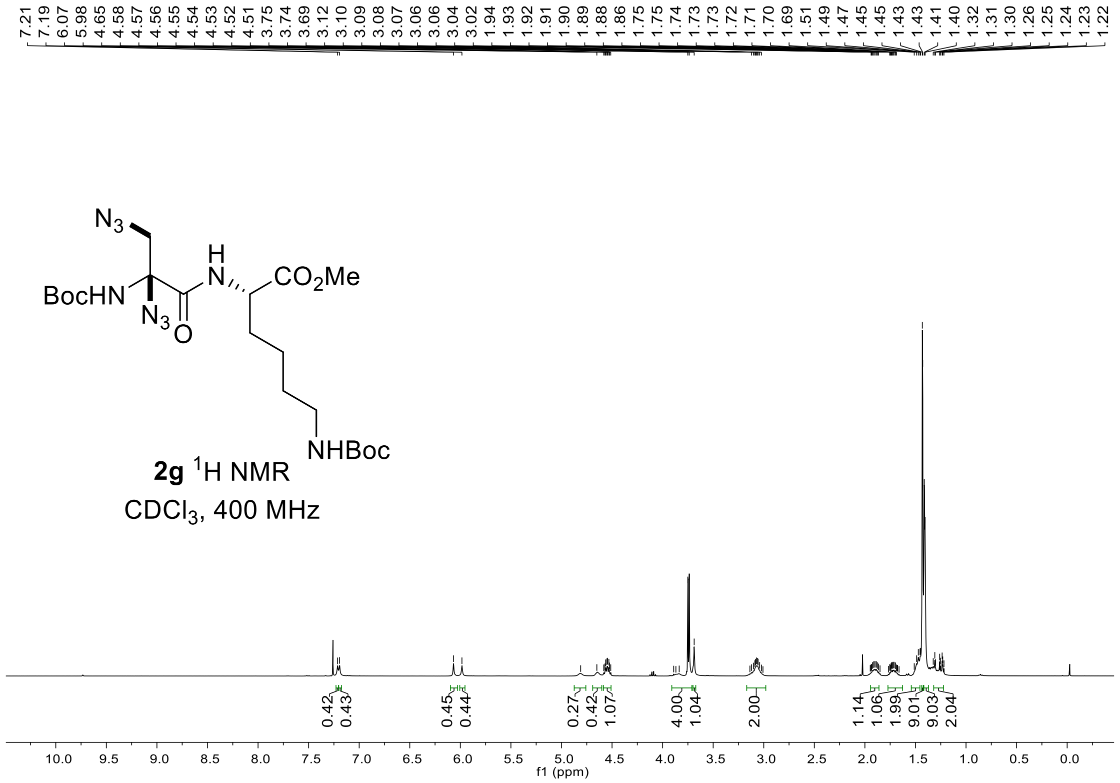
7.21 7.19 6.07 5.98 4.65 4.58 4.57 4.56 4.55 4.54 4.53 4.52 4.51 3.75 3.74 3.69 3.12 3.10 3.09 3.08 3.07 3.06 3.06 3.04 3.02 1.94 1.93 1.92 1.91 1.90 1.89 1.88 1.86 1.75 1.75 1.74 1.73 1.73 1.72 1.71 1.70 1.69 1.51 1.49 1.47 1.45 1.45 1.43 1.43 1.41 1.40 1.32 1.31 1.30 1.26 1.25 1.24 1.23 1.22
N3
H
N
CO2Me
BocHN
N3
O
NHBoc
2g 1H NMR
CDCl3, 400 MHz
0.42 0.43 0.45 0.44 0.27 0.42 1.07 4.00 1.04 2.00 1.14 1.06 1.99 9.01 9.03 2.04
10.0 9.5 9.0 8.5 8.0 7.5 7.0 6.5 6.0 5.5 5.0 4.5 4.0 3.5 3.0 2.5 2.0 1.5 1.0 0.5 0.0
f1 (ppm)

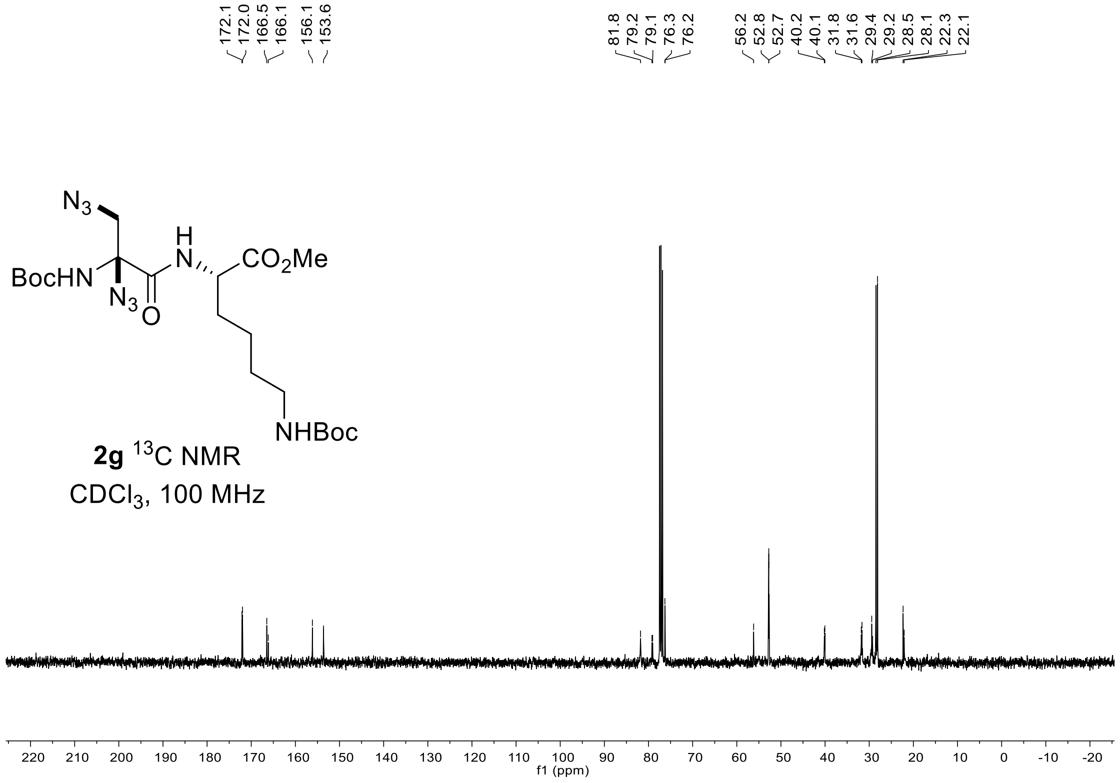
172.1 172.0 166.5 166.1 156.1 153.6 81.8 79.2 79.1 76.3 76.2 56.2 52.8 52.7 40.2 40.1 31.8 31.6 29.4 29.2 28.5 28.1 22.3 22.1
N3
H
N
CO2Me
BocHN
N3
O
NHBoc
2g 13C NMR
CDCl3, 100 MHz
220 210 200 190 180 170 160 150 140 130 120 110 100 90 80 70 60 50 40 30 20 10 0 -10 -20
f1 (ppm)

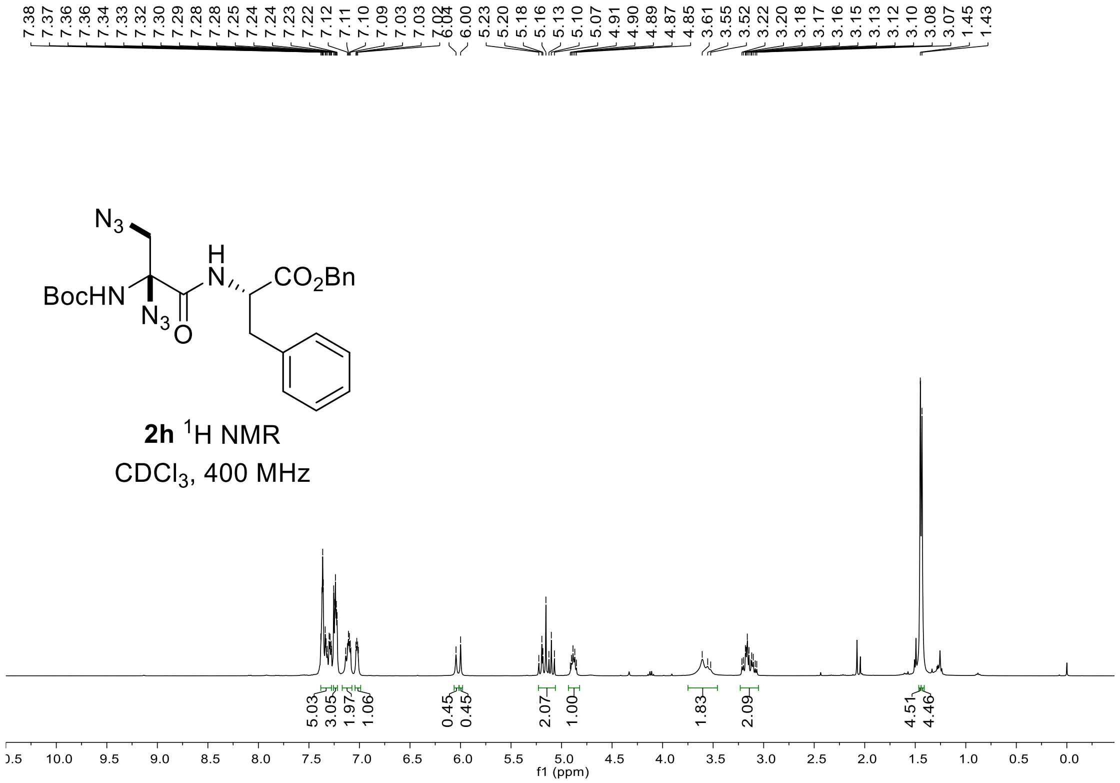
7.38 7.37 7.36 7.34 7.33 7.32 7.30 7.29 7.28 7.28 7.25 7.24 7.24 7.23 7.22 7.12 7.11 7.10 7.09 7.03 7.02 6.04 6.00 5.23 5.20 5.18 5.16 5.13 5.10 5.07 4.91 4.90 4.89 4.87 4.85 3.61 3.55 3.52 3.22 3.20 3.18 3.17 3.16 3.15 3.13 3.12 3.10 3.08 3.07 1.45 1.43
N3
H
N
CO2Bn
BocHN
N3
O
2h 1H NMR
CDCl3, 400 MHz
5.03 3.05 1.97 1.06 0.45 0.45 2.07 1.00 1.83 2.09 4.51 4.46
f1 (ppm)

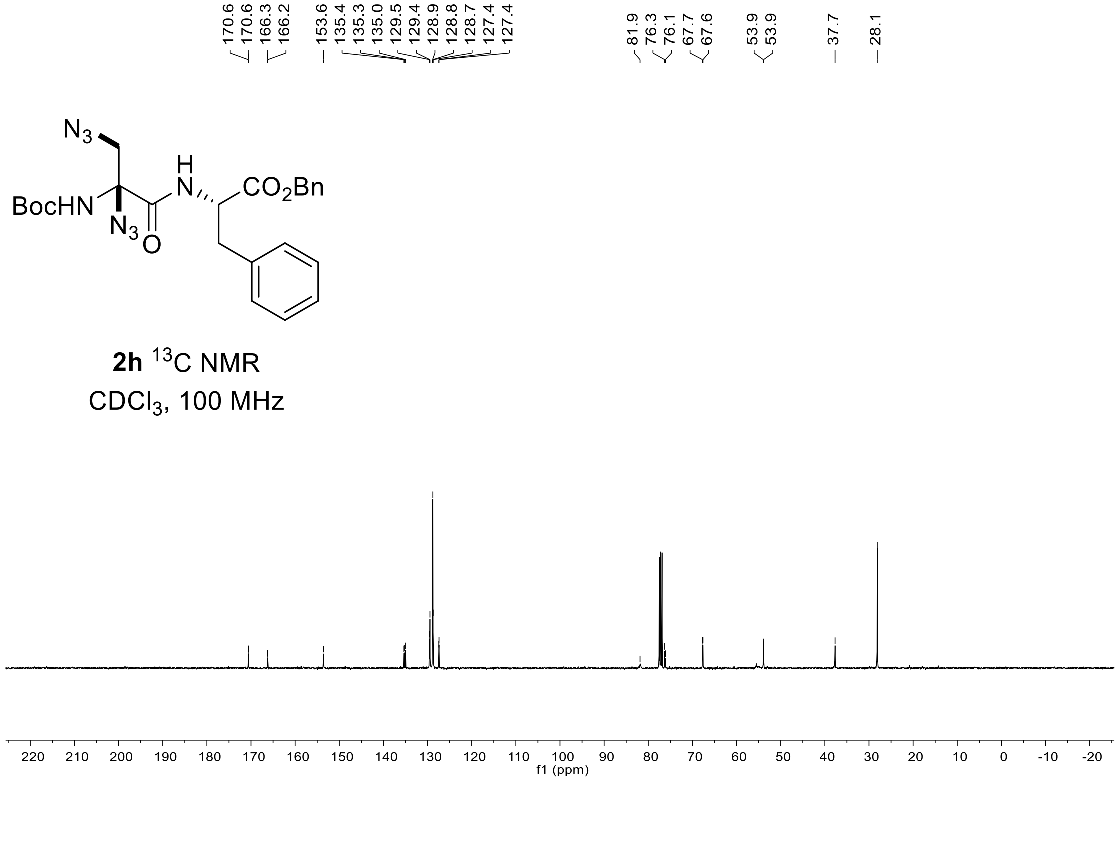
170.6 170.6 166.3 166.2 153.6 135.4 135.3 135.0 129.5 129.4 128.9 128.8 128.7 127.4 127.4 81.9 76.3 76.1 67.7 67.6 53.9 53.9 37.7 28.1
N3
H
N
CO2Bn
BocHN
N3
O
2h 13C NMR
CDCl3, 100 MHz
f1 (ppm)

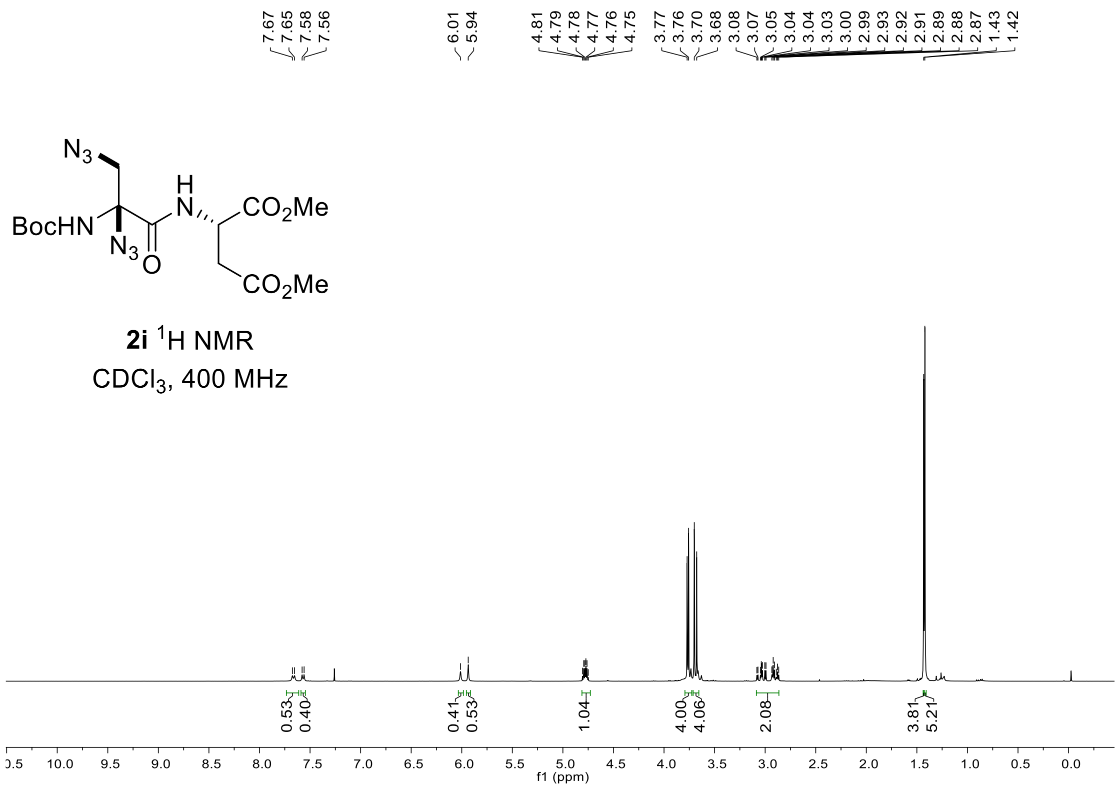

2i ^{1}H NMR
$CDCl_3$, 400 MHz
f1 (ppm)

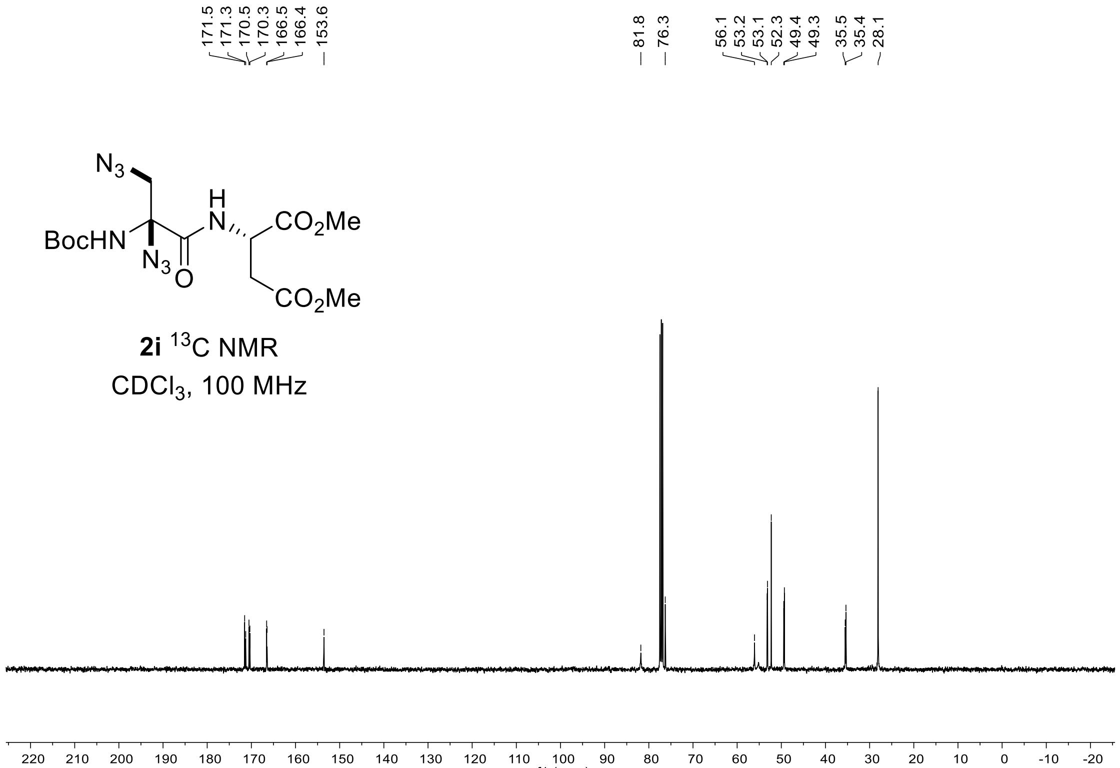

2i ^{13}C NMR
$CDCl_3$, 100 MHz
f1 (ppm)

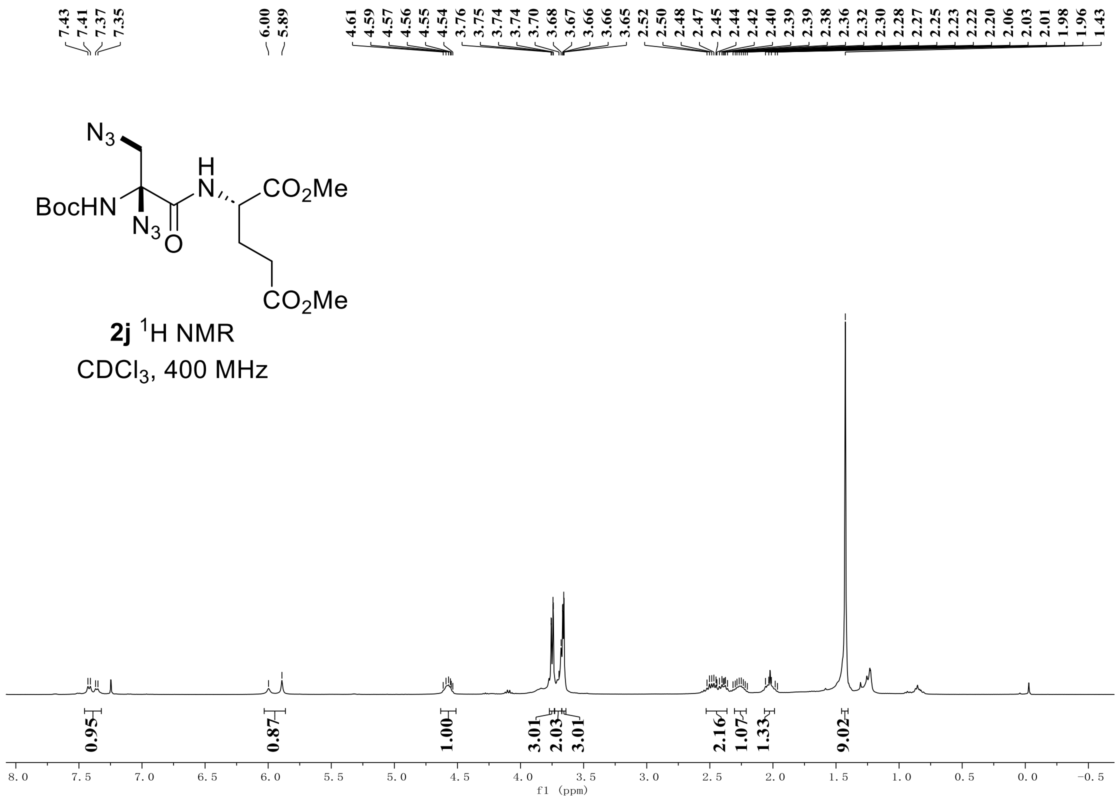
2j ¹H NMR
CDCl3, 400 MHz
f1 (ppm)

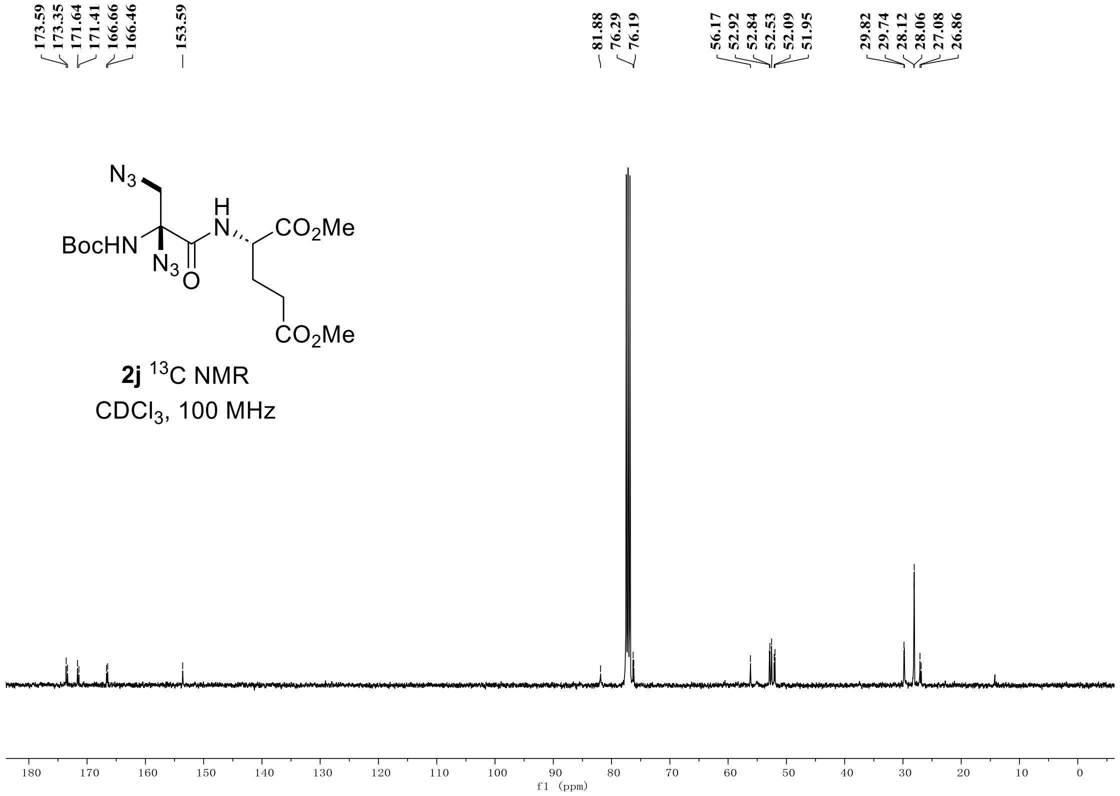
2j ¹³C NMR
CDCl3, 100 MHz
f1 (ppm)

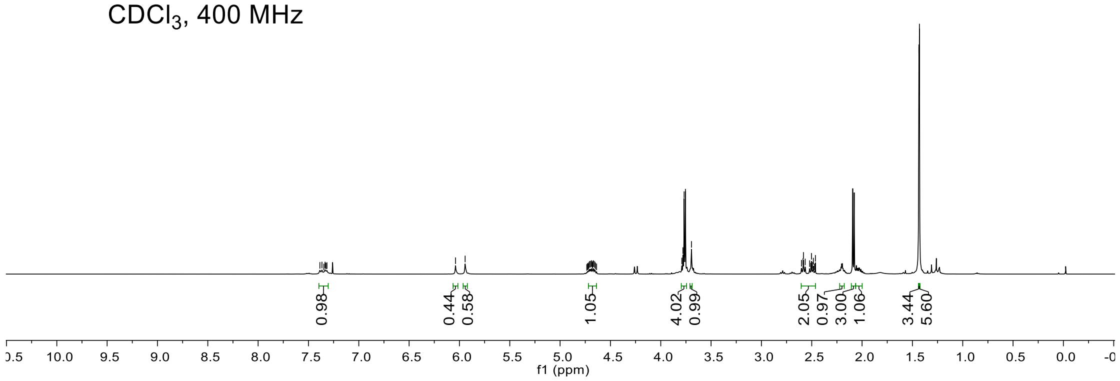

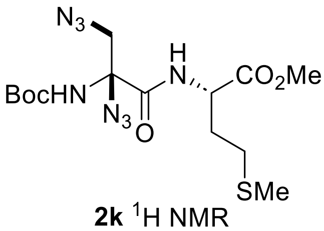

2k ^{1}H NMR

$CDCl_3$, 400 MHz

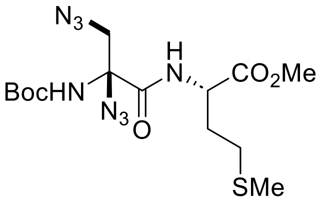

2k ^{13}C NMR

$CDCl_3$, 100 MHz

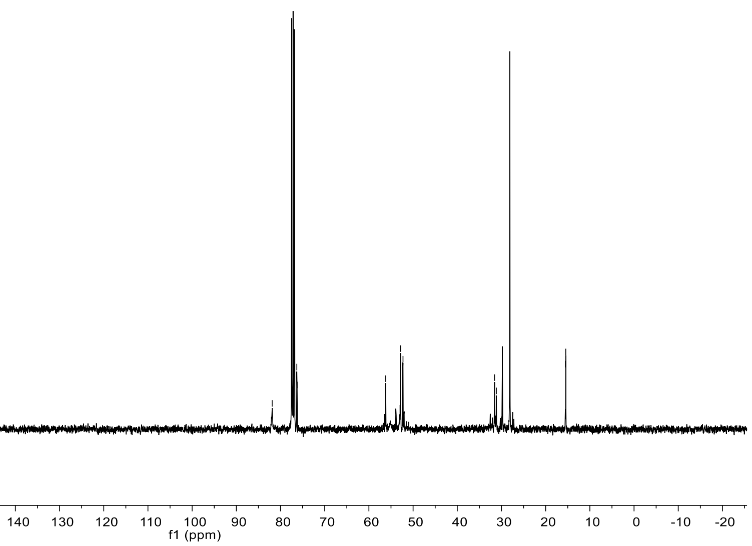

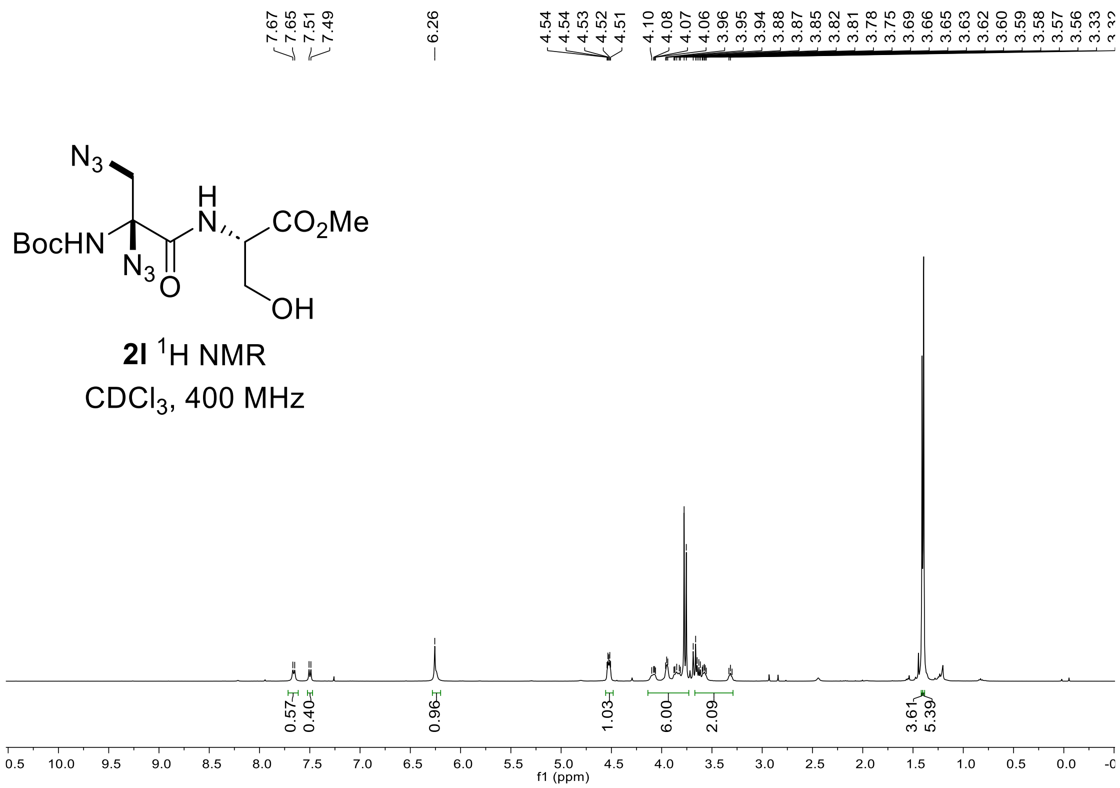
7.67
7.65
7.51
7.49
6.26
4.54
4.54
4.53
4.52
4.51
4.10
4.08
4.07
4.06
3.96
3.95
3.94
3.88
3.87
3.85
3.82
3.81
3.78
3.75
3.69
3.66
3.65
3.63
3.62
3.60
3.59
3.58
3.57
3.56
3.33
N_3
BocHN
N_3
O
H
N
CO_2Me
OH
2l ^{1}H NMR
$CDCl_3$, 400 MHz
0.57
0.40
0.96
1.03
6.00
2.09
3.61
5.39
f1 (ppm)

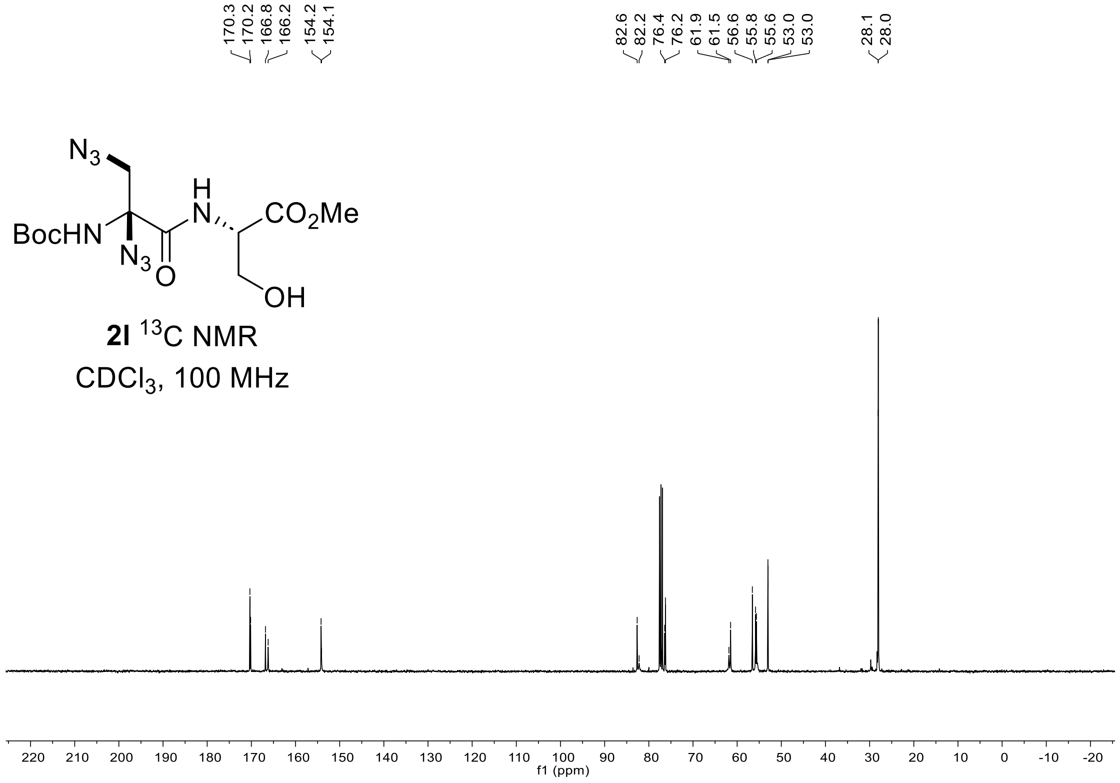
170.3
170.2
166.8
166.2
154.2
154.1
82.6
82.2
76.4
76.2
61.9
61.5
56.6
55.8
55.6
53.0
53.0
28.1
28.0
N_3
BocHN
N_3
O
H
N
CO_2Me
OH
2l ^{13}C NMR
$CDCl_3$, 100 MHz
f1 (ppm)

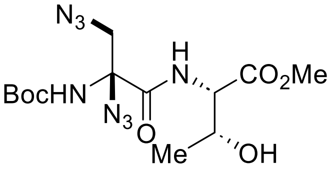

2m ^{1}H NMR

$CDCl_3$, 400 MHz

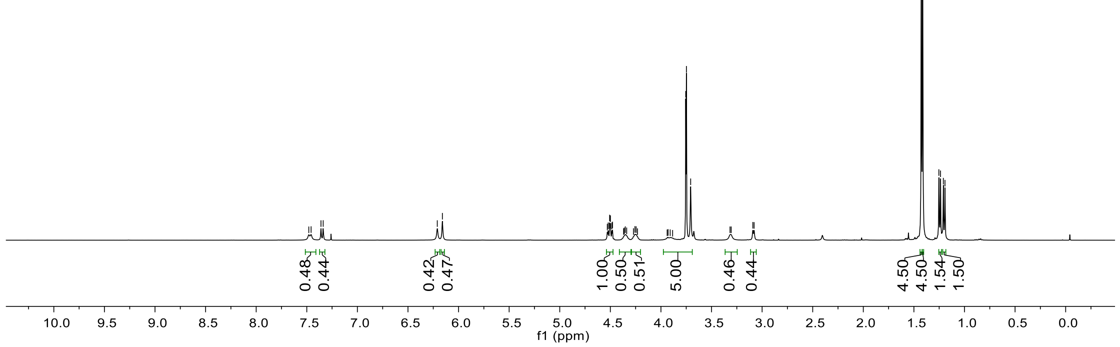

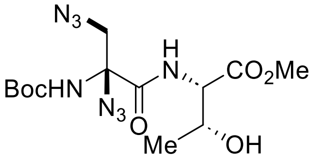

2m ^{13}C NMR

$CDCl_3$, 100 MHz

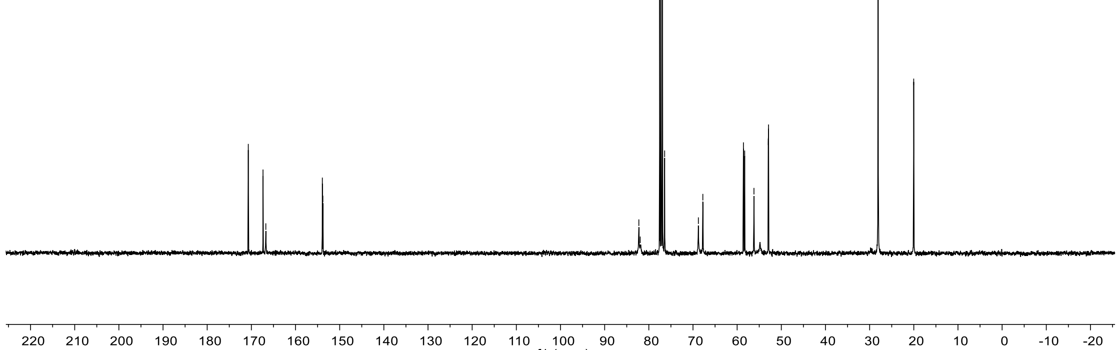

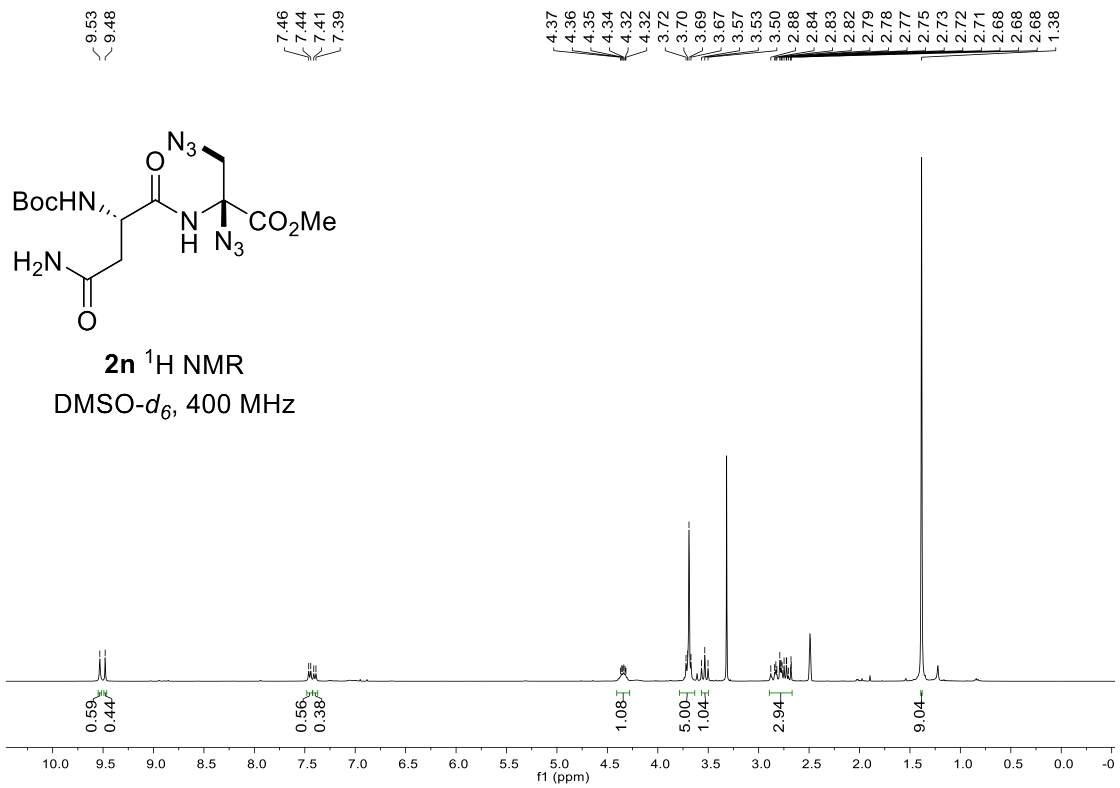
9.53
9.48
7.46
7.44
7.41
7.39
4.37
4.36
4.35
4.34
4.32
4.32
3.72
3.70
3.69
3.67
3.57
3.53
3.50
2.88
2.84
2.83
2.82
2.79
2.78
2.77
2.75
2.73
2.72
2.71
2.68
2.68
2.68
1.38
N3
O
BocHN
N
H
N3
CO2Me
H2N
O
2n 1H NMR
DMSO-d6, 400 MHz
0.59
0.44
0.56
0.38
1.08
5.00
1.04
2.94
9.04
f1 (ppm)

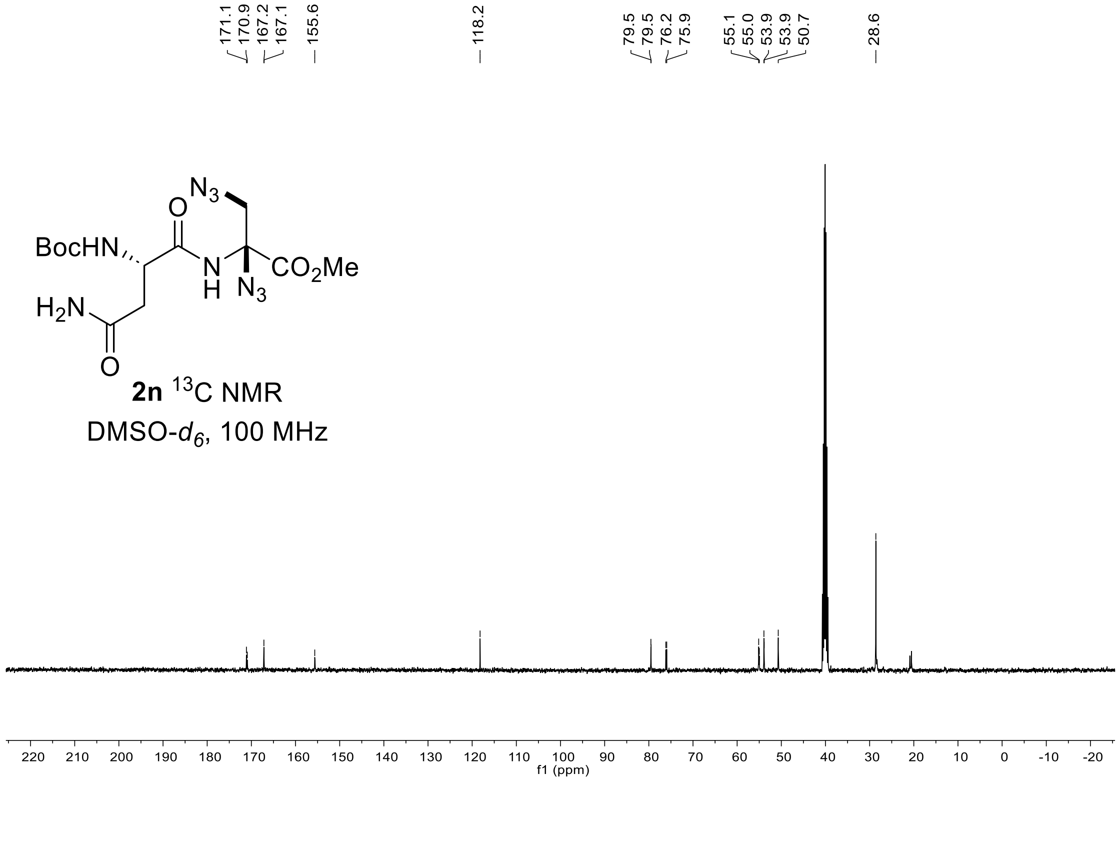
171.1
170.9
167.2
167.1
155.6
118.2
79.5
79.5
76.2
75.9
55.1
55.0
53.9
53.9
50.7
28.6
N3
O
BocHN
N
H
N3
CO2Me
H2N
O
2n 13C NMR
DMSO-d6, 100 MHz
f1 (ppm)

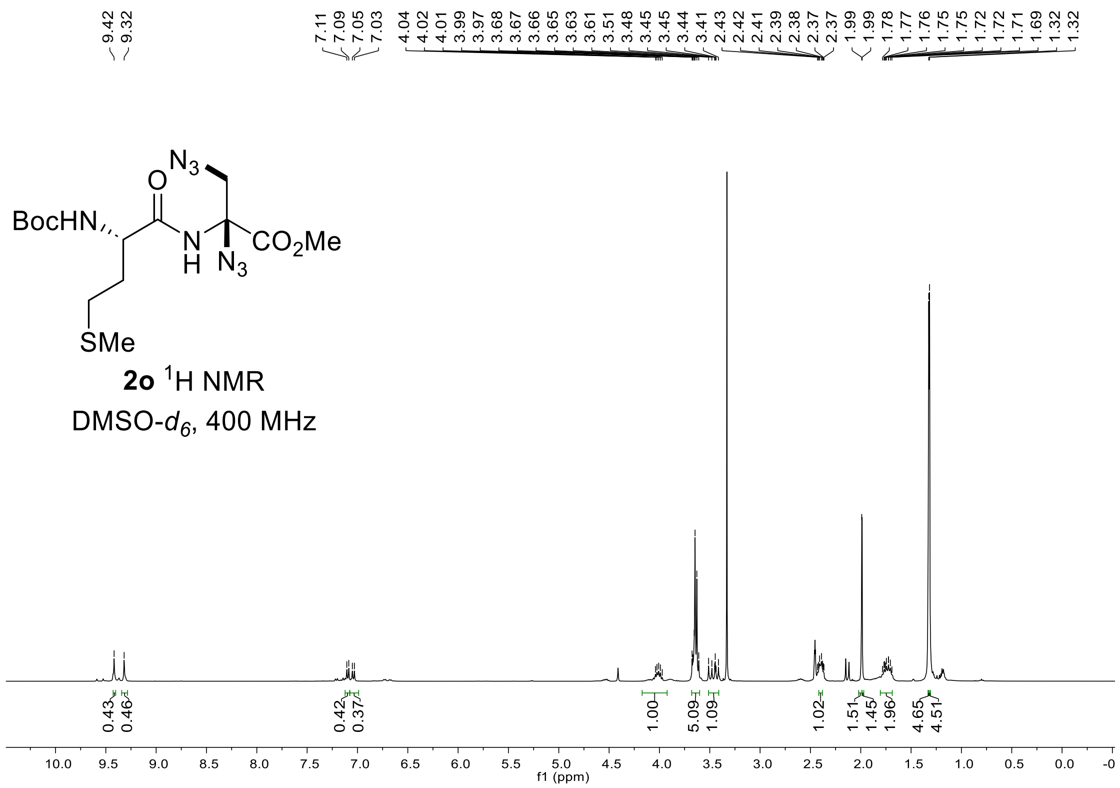
9.42
9.32
7.11
7.09
7.05
7.03
4.04
4.02
4.01
3.99
3.97
3.68
3.67
3.66
3.65
3.63
3.61
3.51
3.48
3.45
3.45
3.44
3.41
2.43
2.42
2.41
2.39
2.38
2.37
2.37
1.99
1.99
1.78
1.77
1.76
1.75
1.75
1.72
1.72
1.71
1.69
1.32
1.32
BocHN
N
H
O
N_3
N_3
CO_2Me
SMe
2o ^{1}H NMR
DMSO-d_6, 400 MHz
0.43
0.46
0.42
0.37
1.00
5.09
1.09
1.02
1.51
1.45
1.96
4.65
4.51
f1 (ppm)

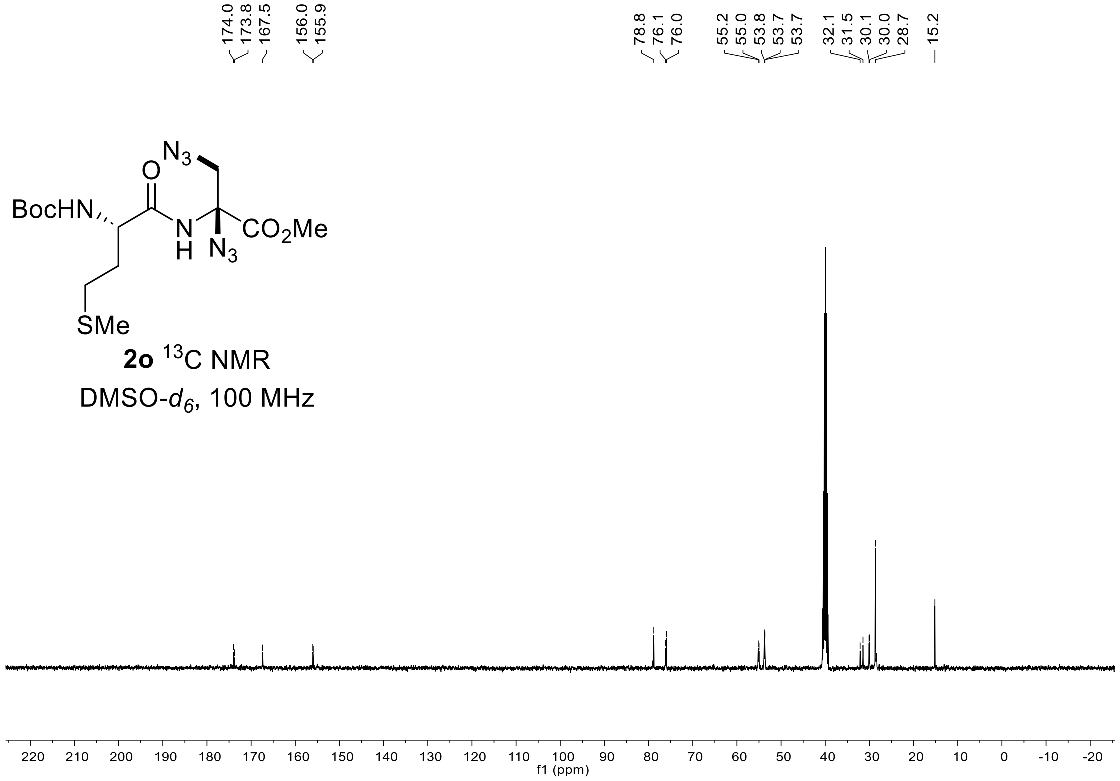
174.0
173.8
167.5
156.0
155.9
78.8
76.1
76.0
55.2
55.0
53.8
53.7
53.7
32.1
31.5
30.1
30.0
28.7
15.2
BocHN
N
H
O
N_3
N_3
CO_2Me
SMe
2o ^{13}C NMR
DMSO-d_6, 100 MHz
f1 (ppm)

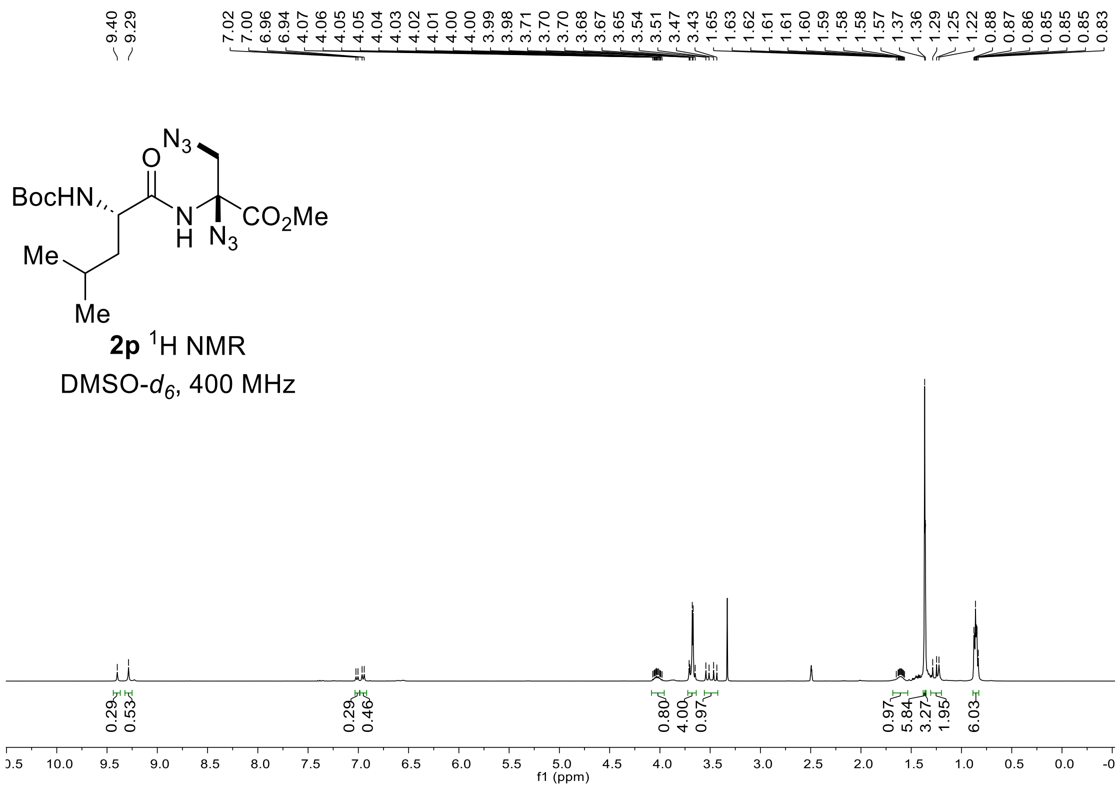
9.40
9.29
7.02
7.00
6.96
6.94
4.07
4.06
4.05
4.05
4.04
4.03
4.02
4.01
4.00
4.00
3.99
3.98
3.71
3.70
3.70
3.68
3.67
3.65
3.54
3.51
3.47
3.43
1.65
1.63
1.62
1.61
1.61
1.60
1.59
1.58
1.58
1.57
1.37
1.36
1.29
1.25
1.22
0.88
0.87
0.86
0.85
0.85
0.85
0.83
N_3
O
BocHN
N
H
N_3
CO_2Me
Me
Me
2p ^{1}H NMR
DMSO-d_6, 400 MHz
0.29
0.53
0.29
0.46
0.80
4.00
0.97
0.97
5.84
3.27
1.95
6.03
f1 (ppm)

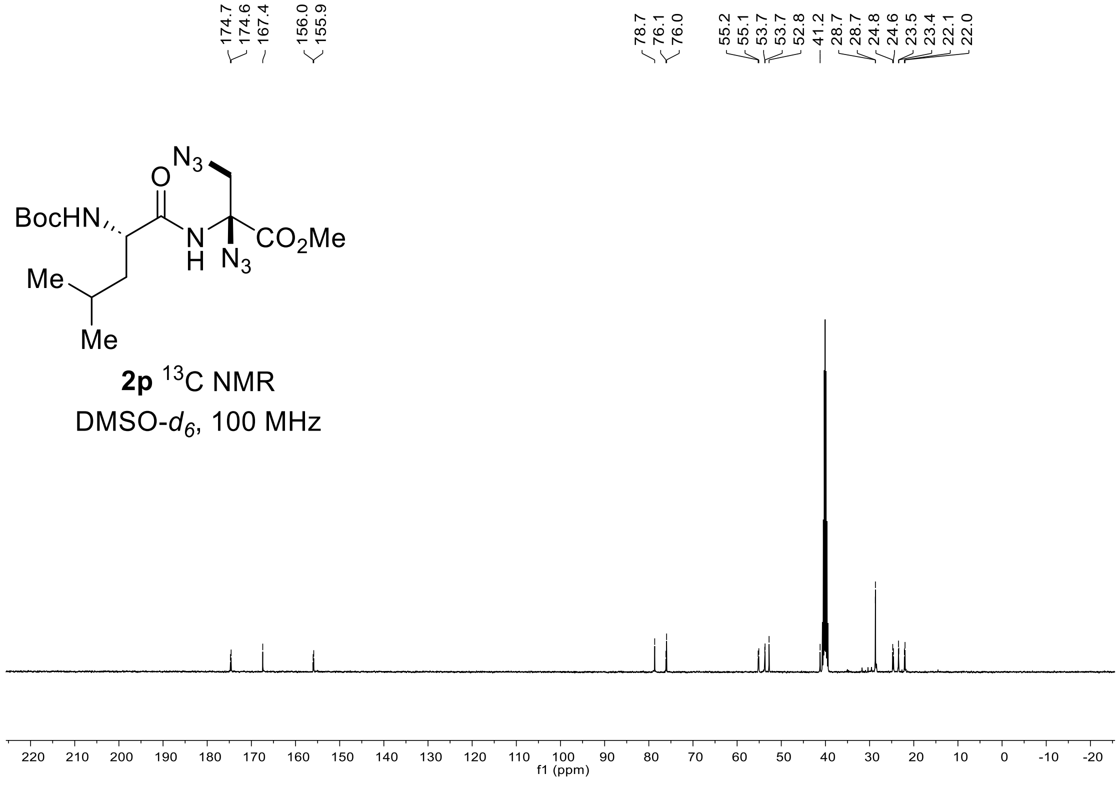
174.7
174.6
167.4
156.0
155.9
78.7
76.1
76.0
55.2
55.1
53.7
53.7
52.8
41.2
28.7
28.7
24.8
24.6
23.5
23.4
22.1
22.0
N_3
O
BocHN
N
H
N_3
CO_2Me
Me
Me
2p ^{13}C NMR
DMSO-d_6, 100 MHz
f1 (ppm)

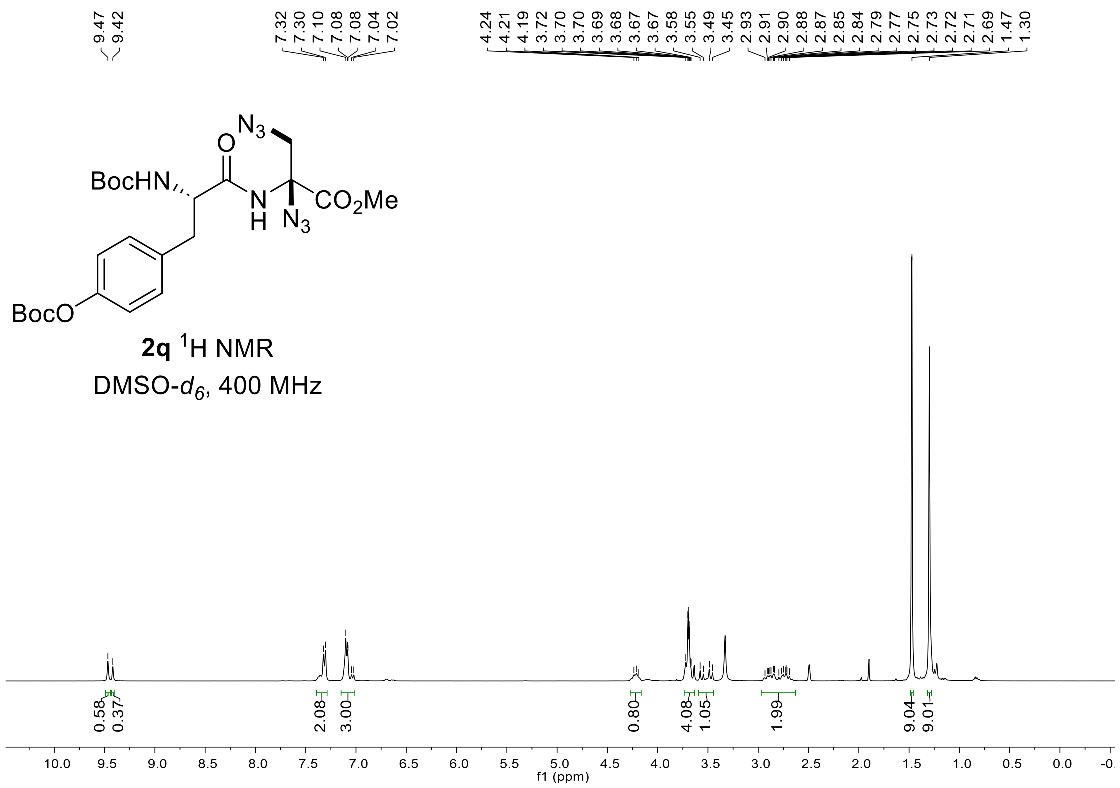
9.47
9.42
7.32
7.30
7.10
7.08
7.08
7.04
7.02
4.24
4.21
4.19
3.72
3.70
3.70
3.69
3.68
3.67
3.67
3.58
3.55
3.49
3.45
2.93
2.91
2.90
2.88
2.87
2.85
2.84
2.79
2.77
2.75
2.73
2.72
2.71
2.69
1.47
1.30
N3
O
BocHN
N
H
N3
CO2Me
BocO
2q 1H NMR
DMSO-d6, 400 MHz
0.58
0.37
2.08
3.00
0.80
4.08
1.05
1.99
9.04
9.01
f1 (ppm)

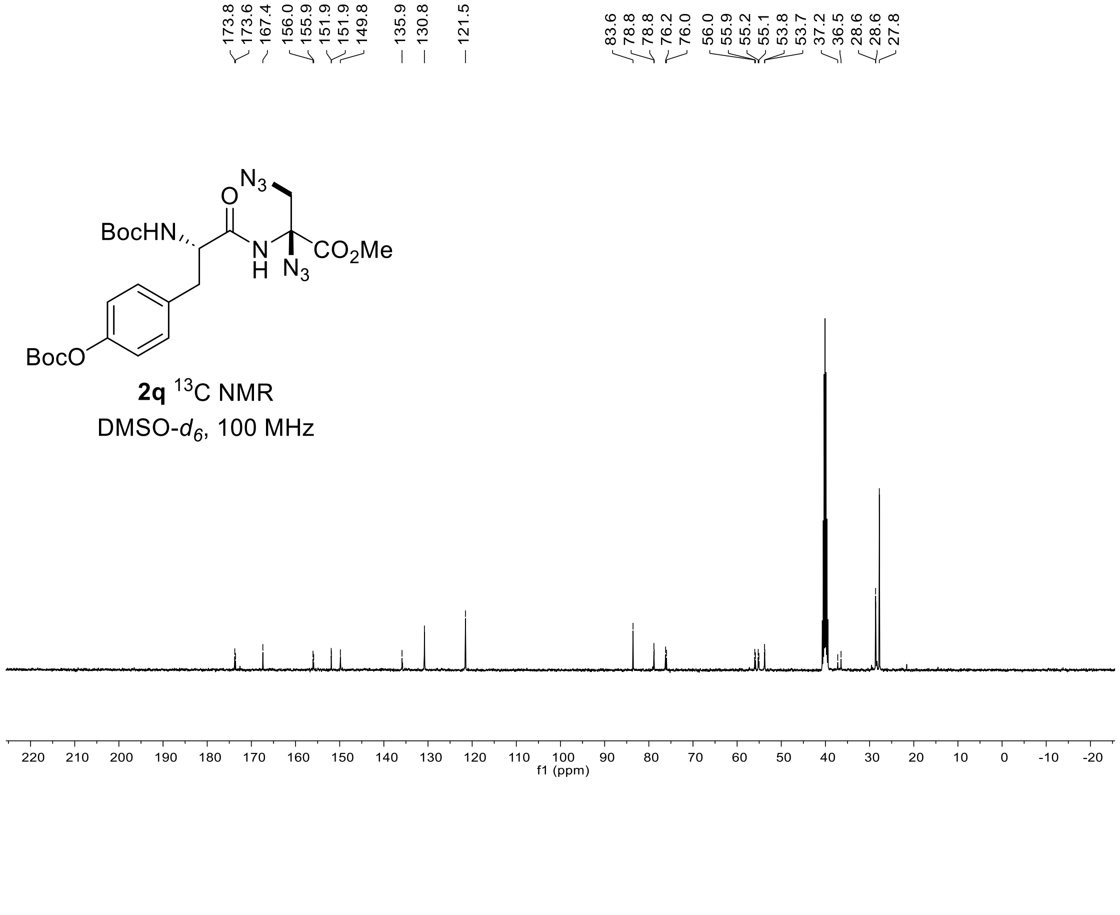
173.8
173.6
167.4
156.0
155.9
151.9
151.9
149.8
135.9
130.8
121.5
83.6
78.8
78.8
76.2
76.0
56.0
55.9
55.2
55.1
53.8
53.7
37.2
36.5
28.6
27.8
N3
O
BocHN
N
H
N3
CO2Me
BocO
2q 13C NMR
DMSO-d6, 100 MHz
f1 (ppm)

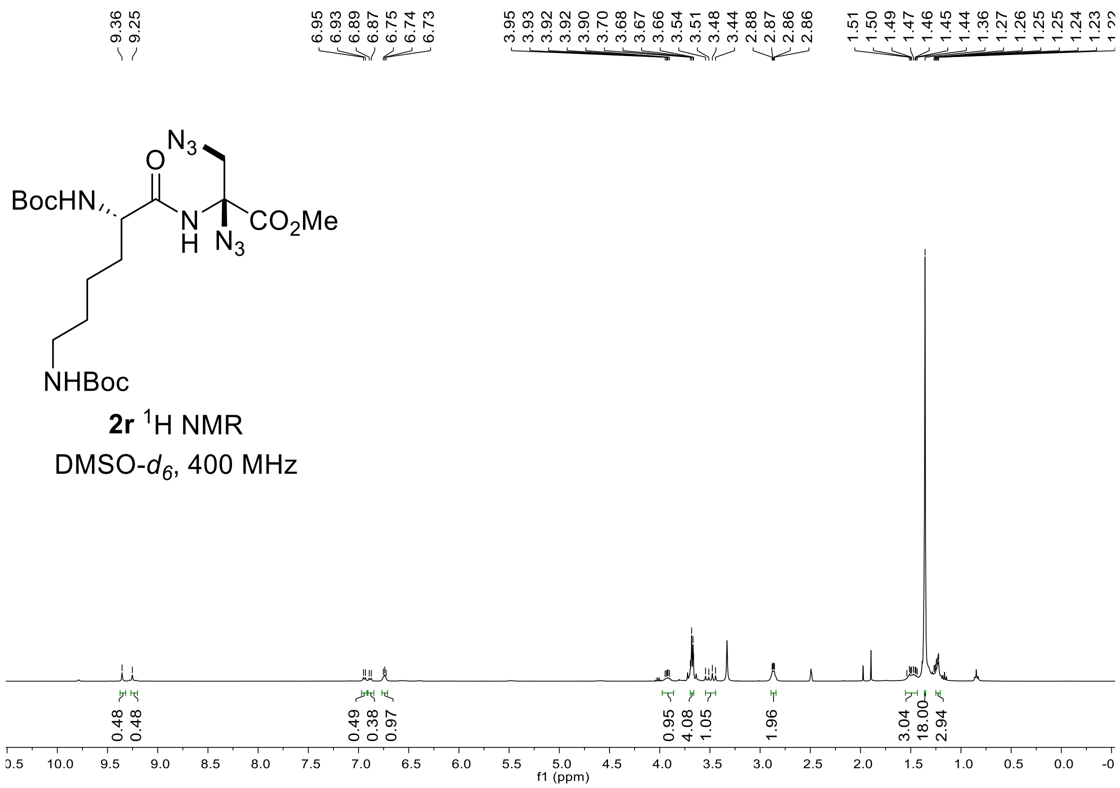

2r ^{1}H NMR
DMSO-d_6, 400 MHz

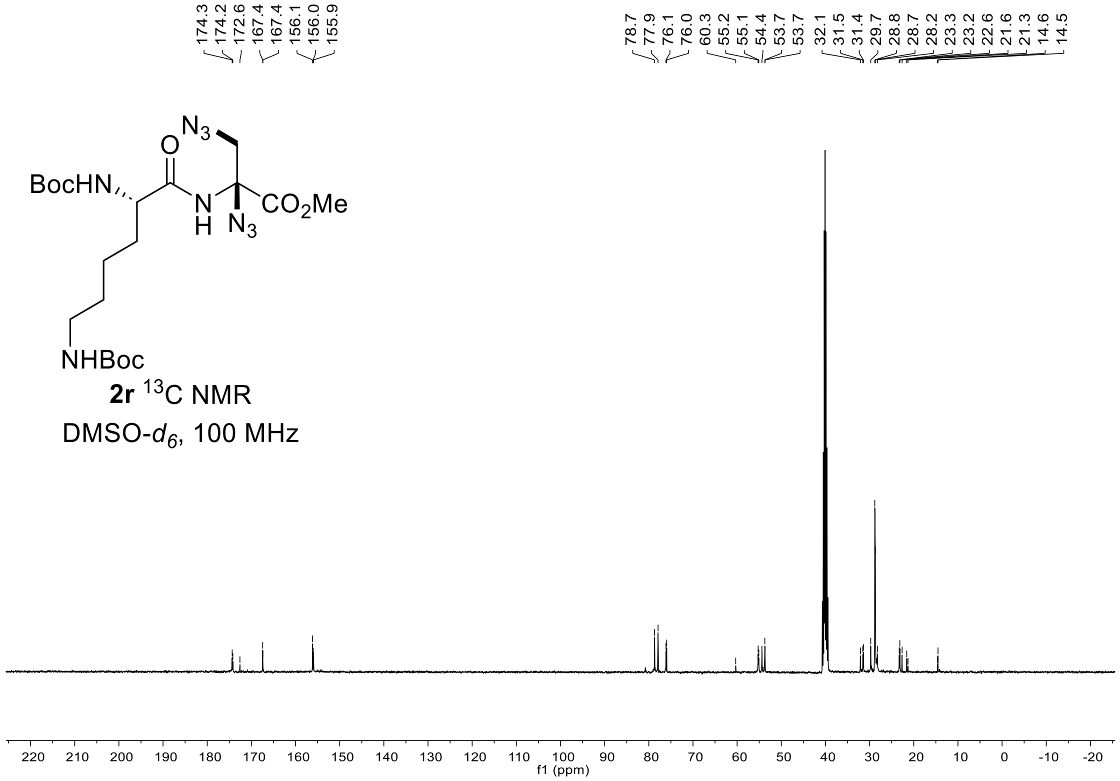

2r ^{13}C NMR
DMSO-d_6, 100 MHz

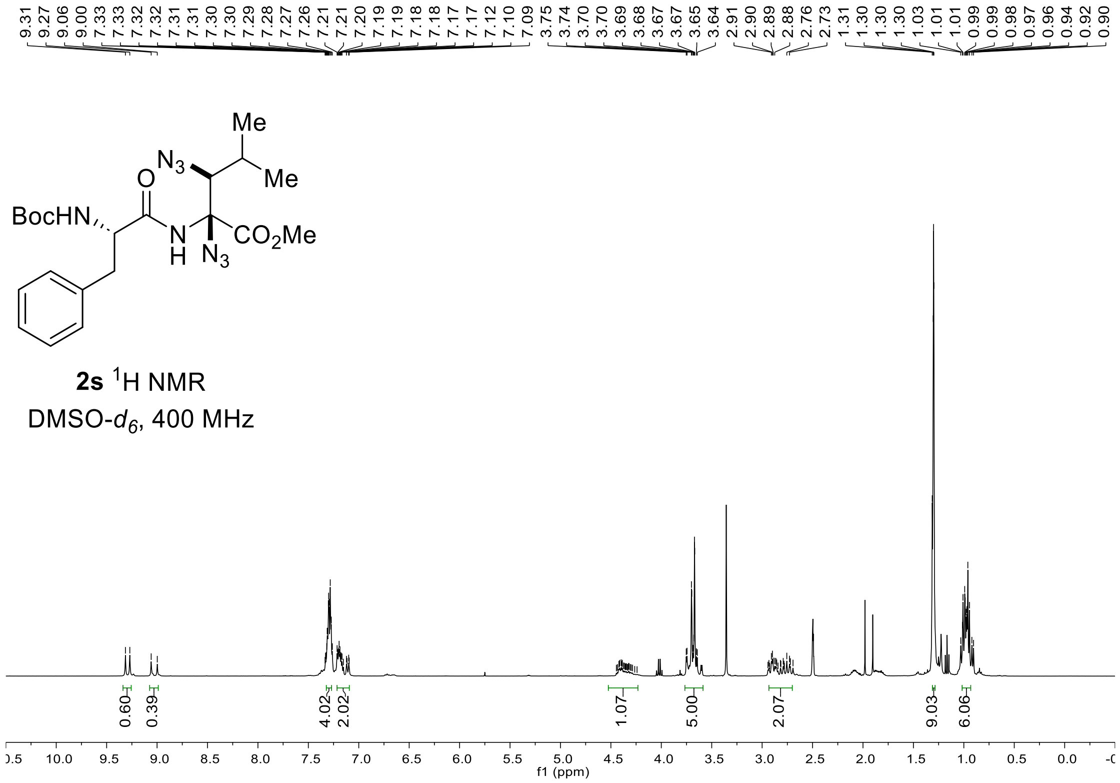
9.31 9.27 9.06 9.00 7.33 7.32 7.32 7.31 7.31 7.30 7.30 7.29 7.28 7.27 7.26 7.21 7.21 7.20 7.19 7.19 7.18 7.18 7.17 7.17 7.12 7.10 7.09 3.75 3.74 3.70 3.70 3.69 3.68 3.67 3.67 3.65 3.64 2.91 2.90 2.89 2.88 2.76 2.73 1.31 1.30 1.30 1.30 1.03 1.01 1.01 0.99 0.99 0.98 0.97 0.96 0.94 0.92 0.90
Me
N3
Me
O
BocHN
N
H
N3
CO2Me
2s 1H NMR
DMSO-d6, 400 MHz
0.60
0.39
4.02
2.02
1.07
5.00
2.07
9.03
6.06
10.0 9.5 9.0 8.5 8.0 7.5 7.0 6.5 6.0 5.5 5.0 4.5 4.0 3.5 3.0 2.5 2.0 1.5 1.0 0.5 0.0
f1 (ppm)

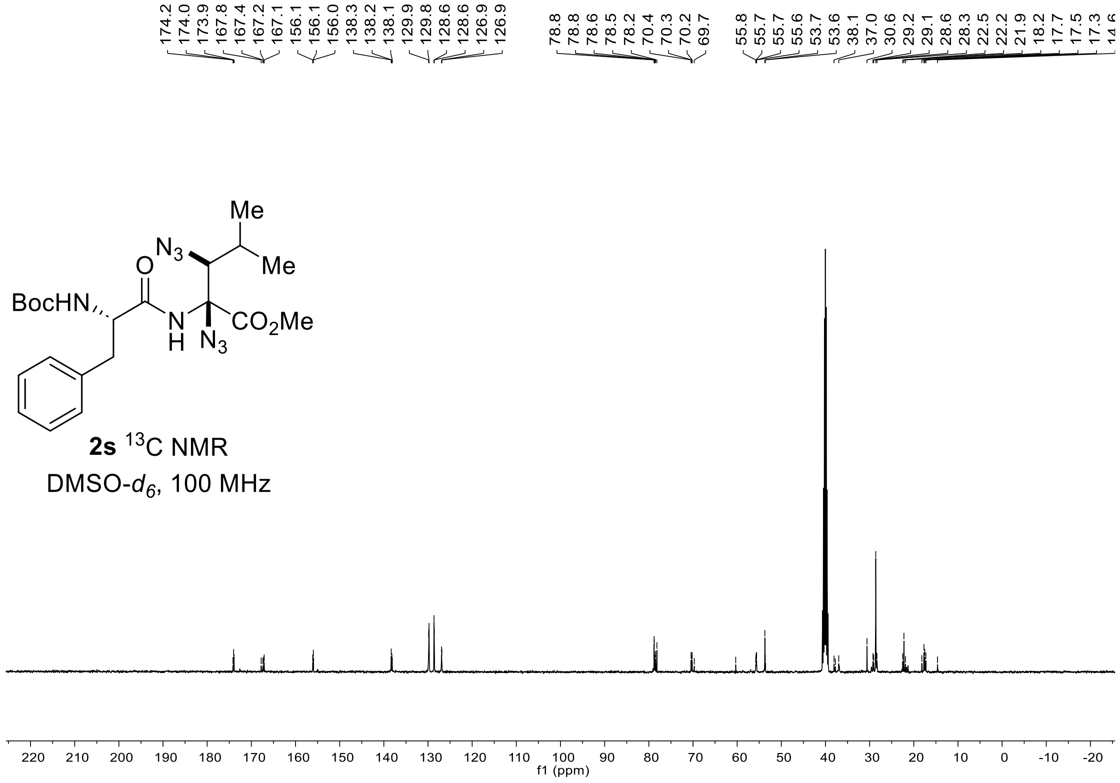
174.2 174.0 173.9 167.8 167.4 167.2 167.1 156.1 156.1 156.0 138.3 138.2 138.1 129.9 129.8 128.6 128.6 126.9 126.9 78.8 78.8 78.6 78.5 78.2 70.4 70.3 70.2 69.7 55.8 55.7 55.7 55.6 53.7 53.6 38.1 37.0 30.6 29.2 29.1 28.6 28.3 22.5 22.2 21.9 18.2 17.7 17.5 17.3
Me
N3
Me
O
BocHN
N
H
N3
CO2Me
2s 13C NMR
DMSO-d6, 100 MHz
220 210 200 190 180 170 160 150 140 130 120 110 100 90 80 70 60 50 40 30 20 10 0 -10 -20
f1 (ppm)

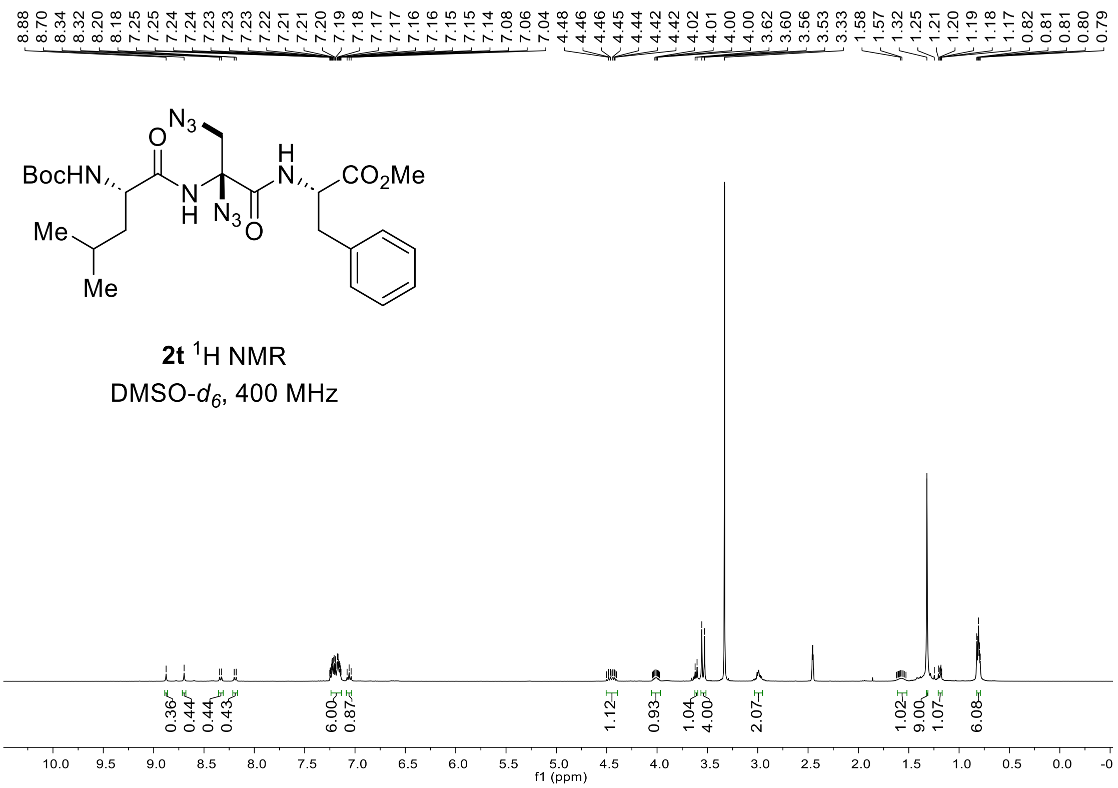

2t ^{1}H NMR
DMSO-d_6, 400 MHz

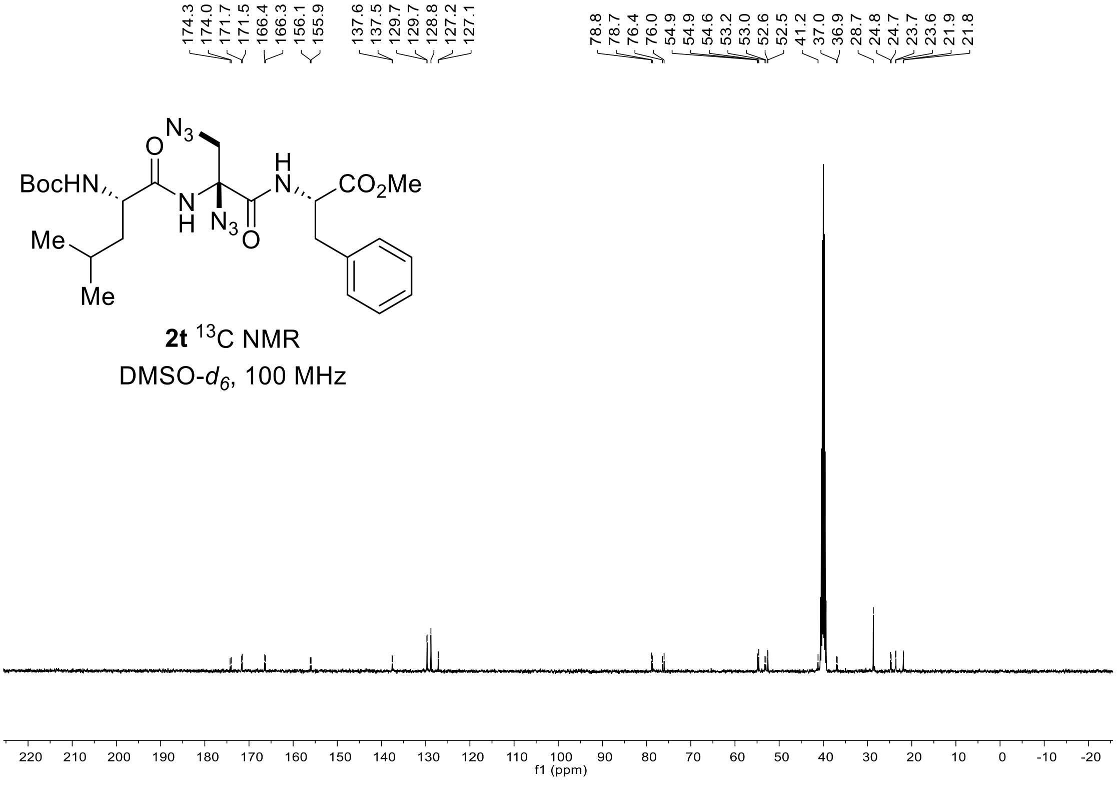

2t ^{13}C NMR
DMSO-d_6, 100 MHz

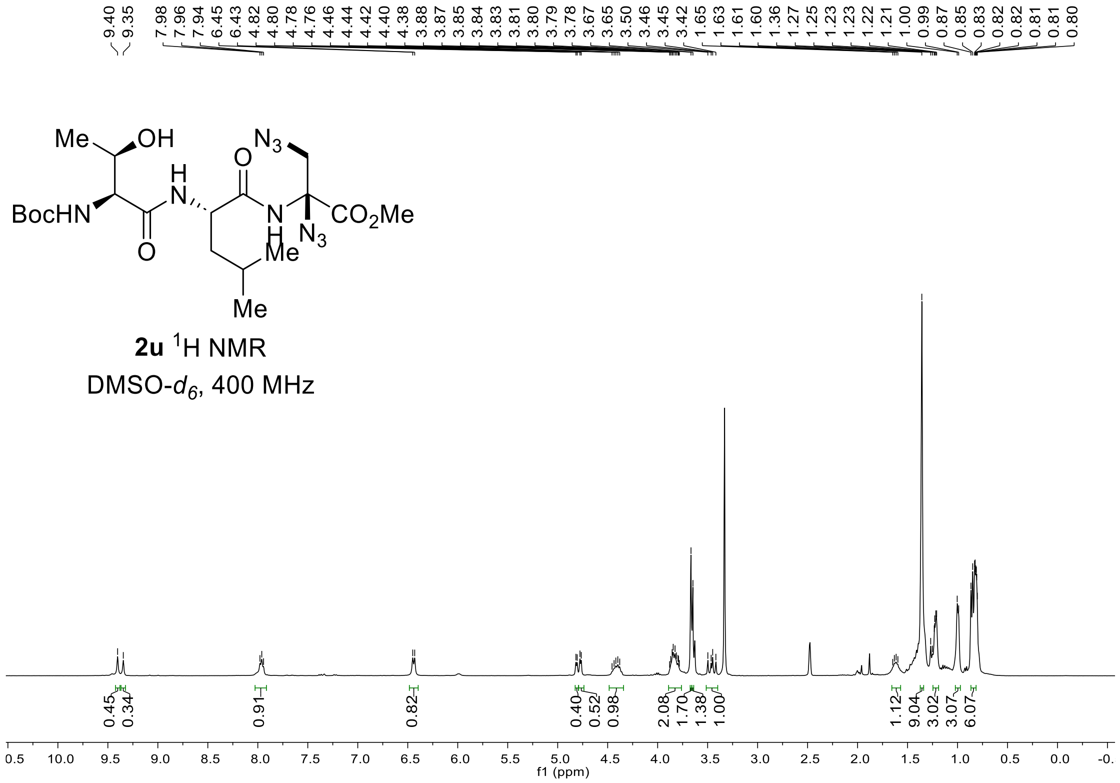

2u ^{1}H NMR
DMSO-d_6, 400 MHz

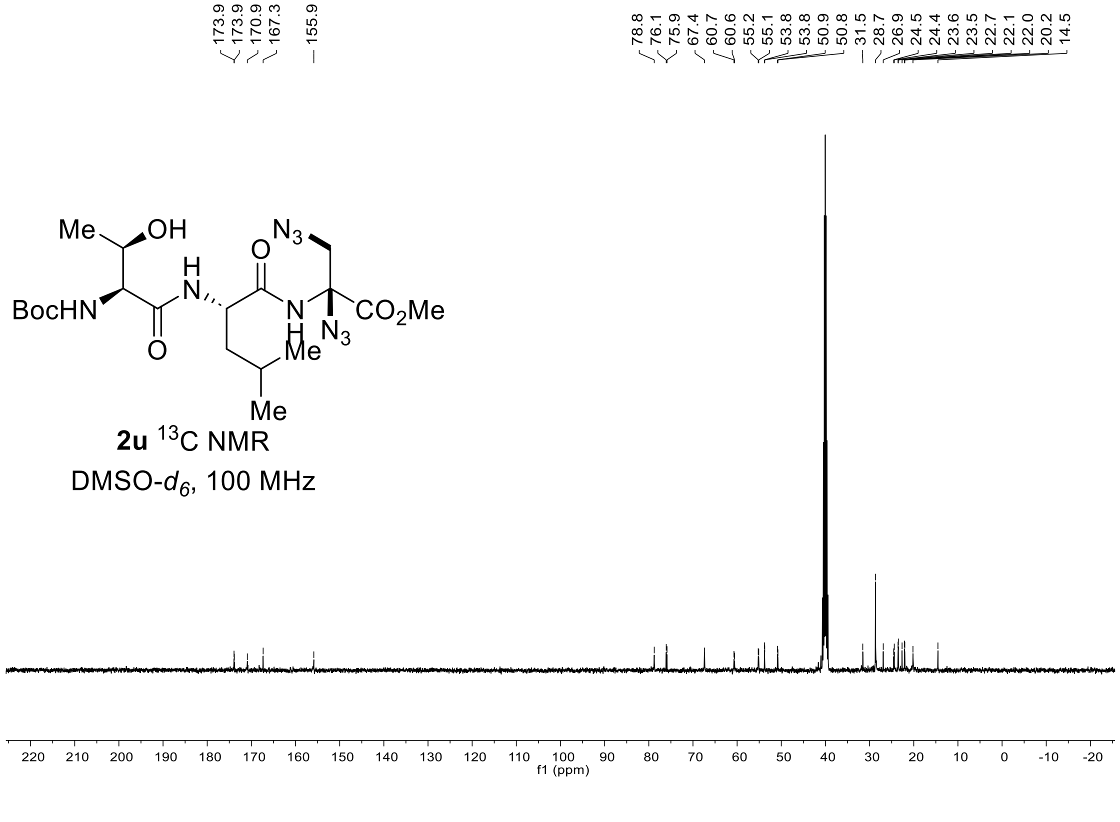

2u ^{13}C NMR
DMSO-d_6, 100 MHz

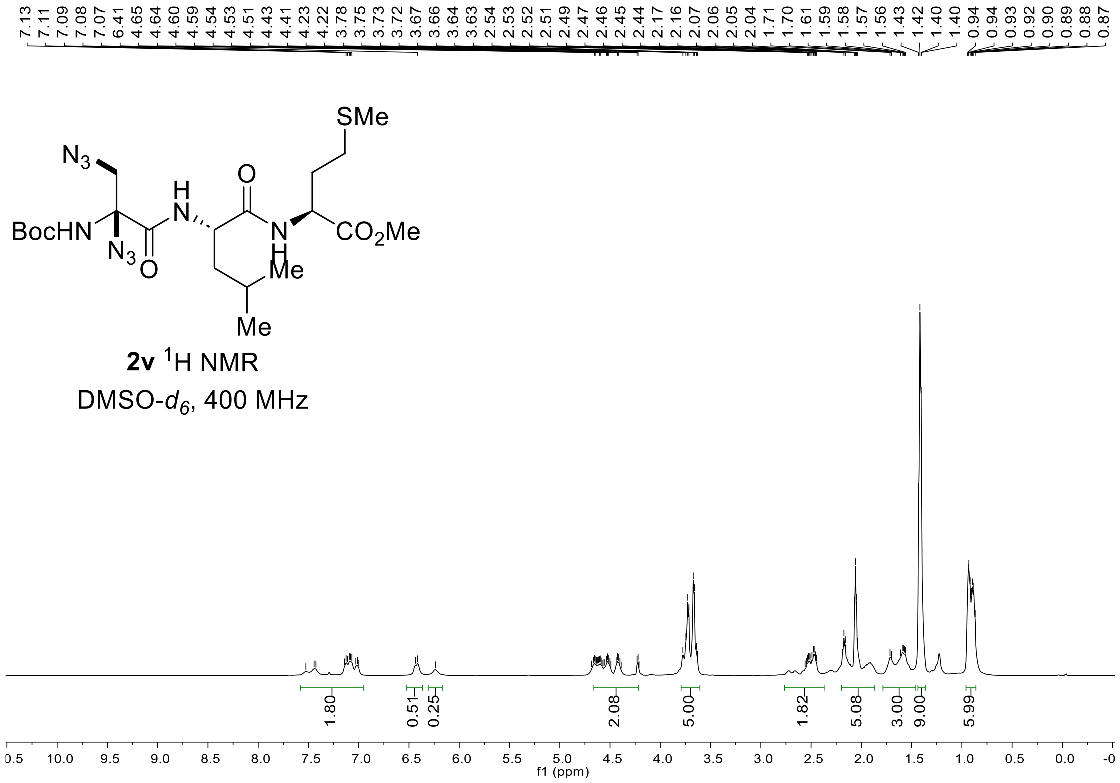
2v 1H NMR
DMSO-d6, 400 MHz
f1 (ppm)

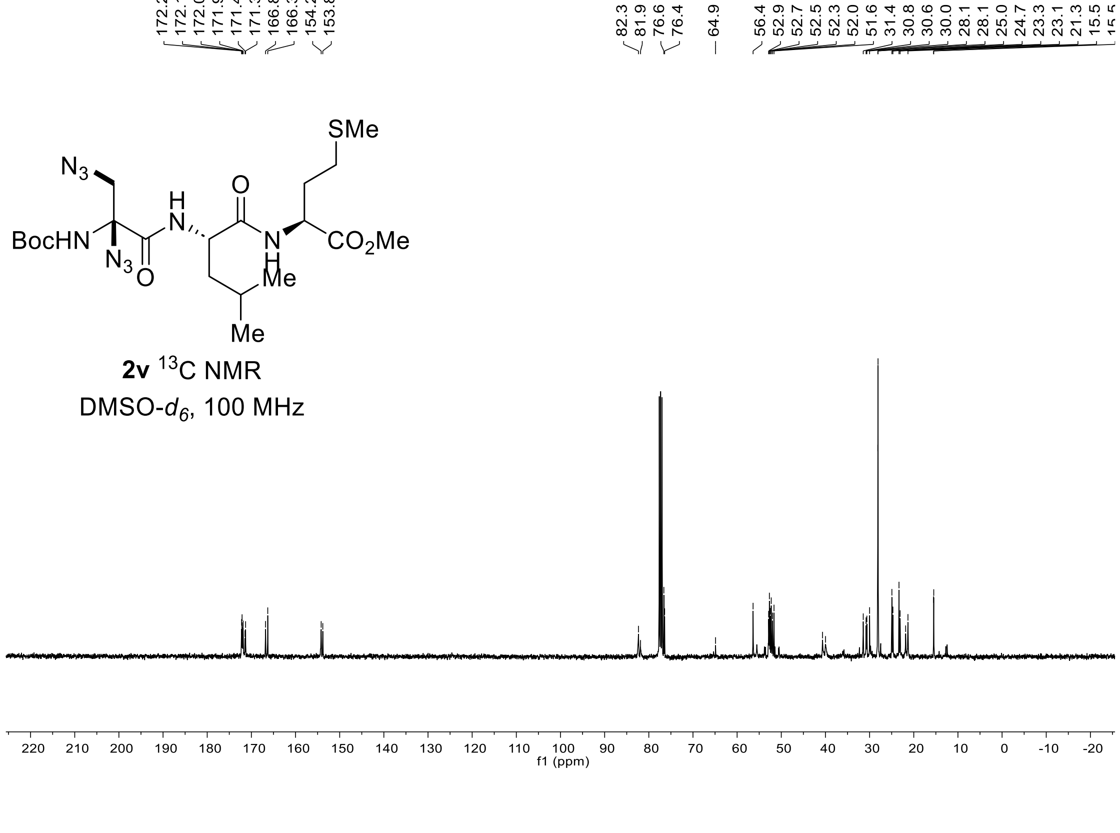
2v 13C NMR
DMSO-d6, 100 MHz
f1 (ppm)

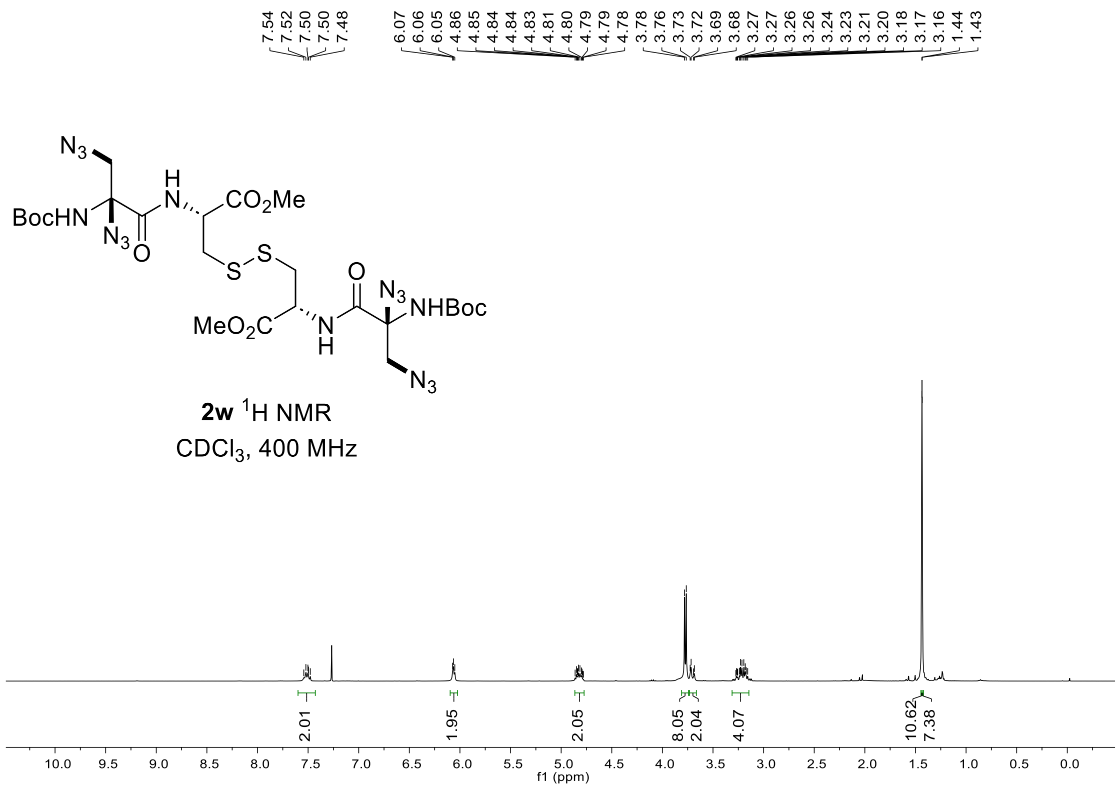

2w ^{1}H NMR
$CDCl_3$, 400 MHz

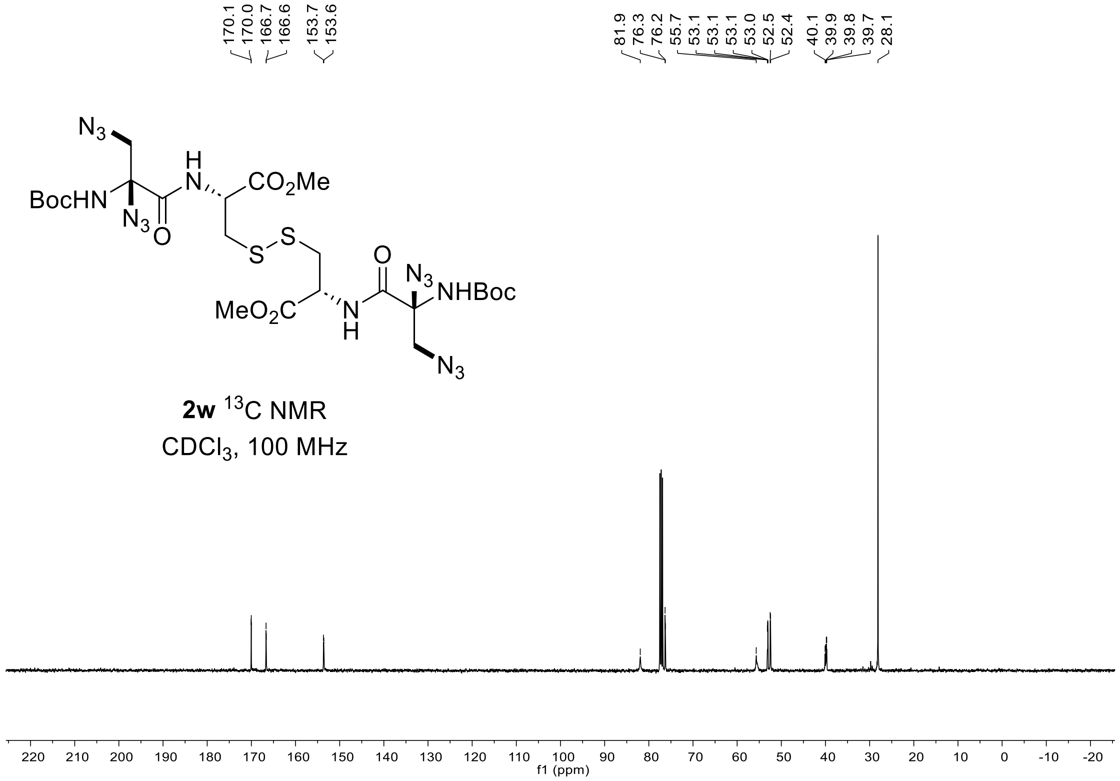

2w ^{13}C NMR
$CDCl_3$, 100 MHz

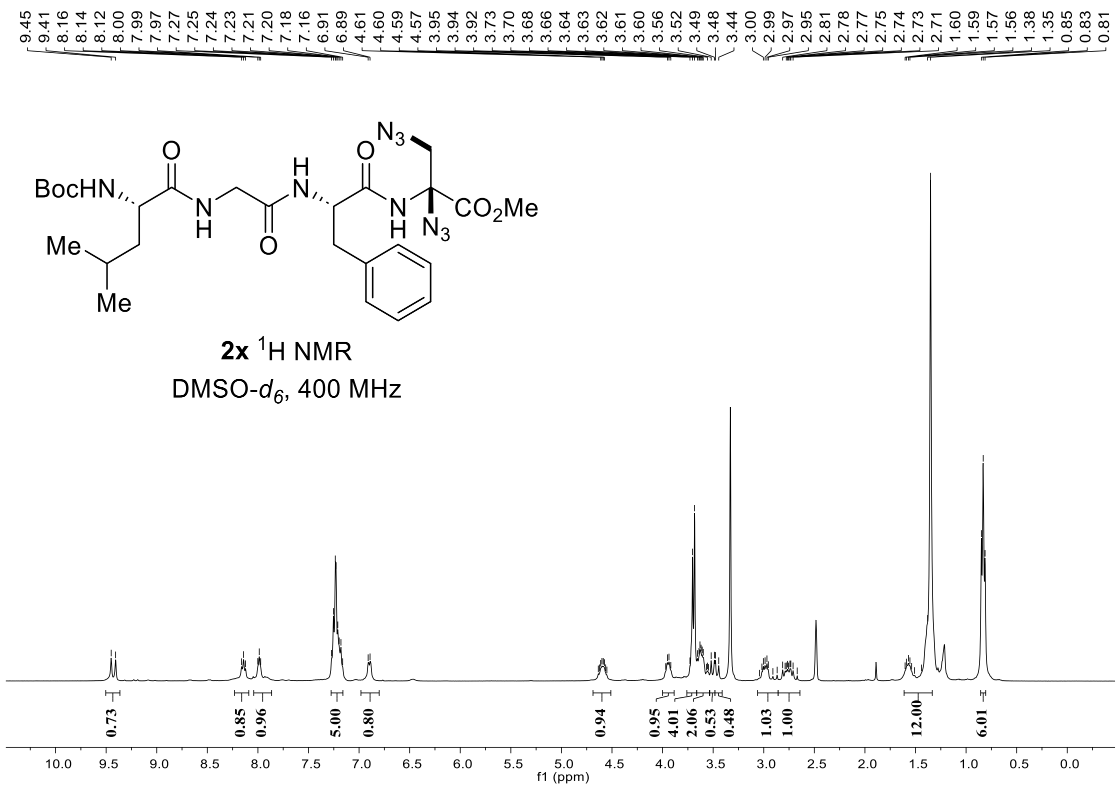

9.45 9.41 8.16 8.14 8.12 8.00 7.99 7.97 7.27 7.25 7.24 7.23 7.21 7.20 7.18 7.16 6.91 6.89 4.61 4.60 4.59 4.57 3.95 3.94 3.92 3.73 3.70 3.68 3.66 3.64 3.63 3.62 3.61 3.60 3.56 3.52 3.49 3.48 3.44 3.00 2.99 2.97 2.95 2.81 2.78 2.77 2.75 2.74 2.73 2.71 1.60 1.59 1.57 1.56 1.38 1.35 0.85 0.83 0.81
BocHN
Me
Me
N3
CO2Me
N3
2x 1H NMR
DMSO-d6, 400 MHz
0.73 0.85 0.96 5.00 0.80 0.94 0.95 4.01 2.06 0.53 0.48 1.03 1.00 12.00 6.01
10.0 9.5 9.0 8.5 8.0 7.5 7.0 6.5 6.0 5.5 5.0 4.5 4.0 3.5 3.0 2.5 2.0 1.5 1.0 0.5 0.0
f1 (ppm)

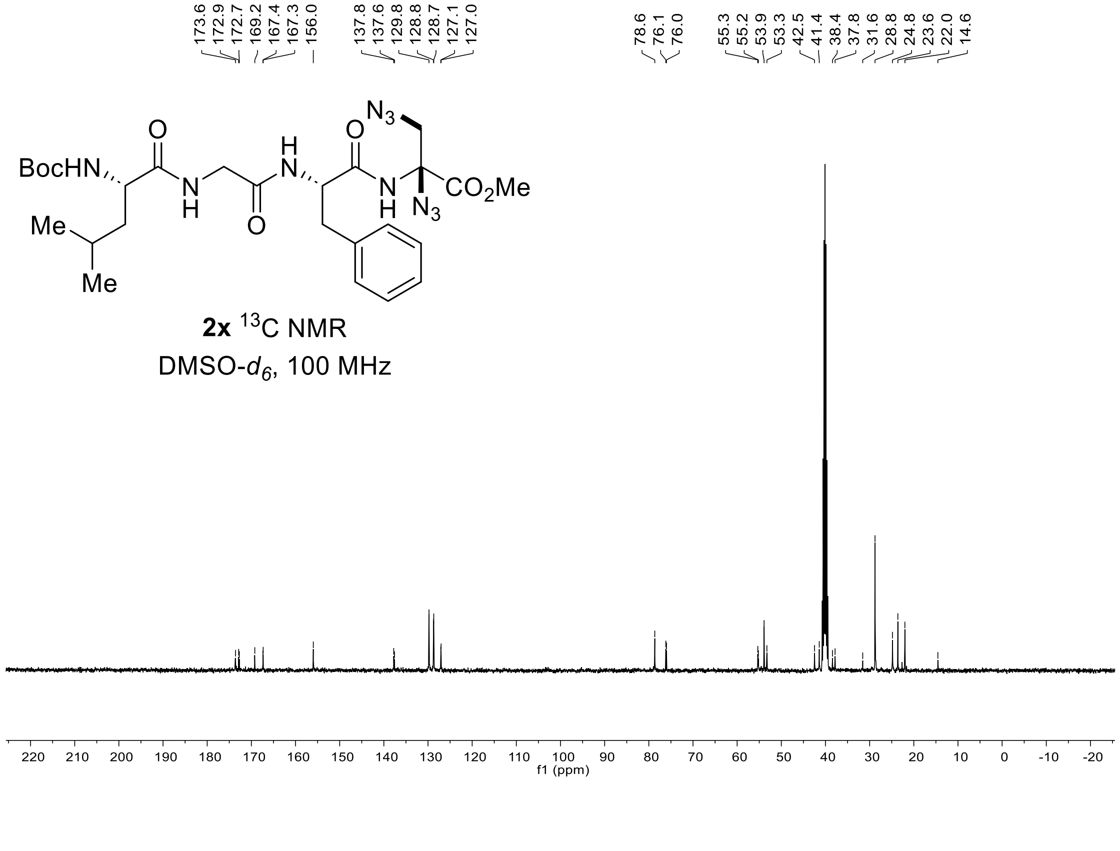

173.6 172.9 172.7 169.2 167.4 167.3 156.0 137.8 137.6 129.8 128.8 128.7 127.1 127.0 78.6 76.1 76.0 55.3 55.2 53.9 53.3 42.5 41.4 38.4 37.8 31.6 28.8 24.8 23.6 22.0 14.6
BocHN
Me
Me
N3
CO2Me
N3
2x 13C NMR
DMSO-d6, 100 MHz
220 210 200 190 180 170 160 150 140 130 120 110 100 90 80 70 60 50 40 30 20 10 0 -10 -20
f1 (ppm)

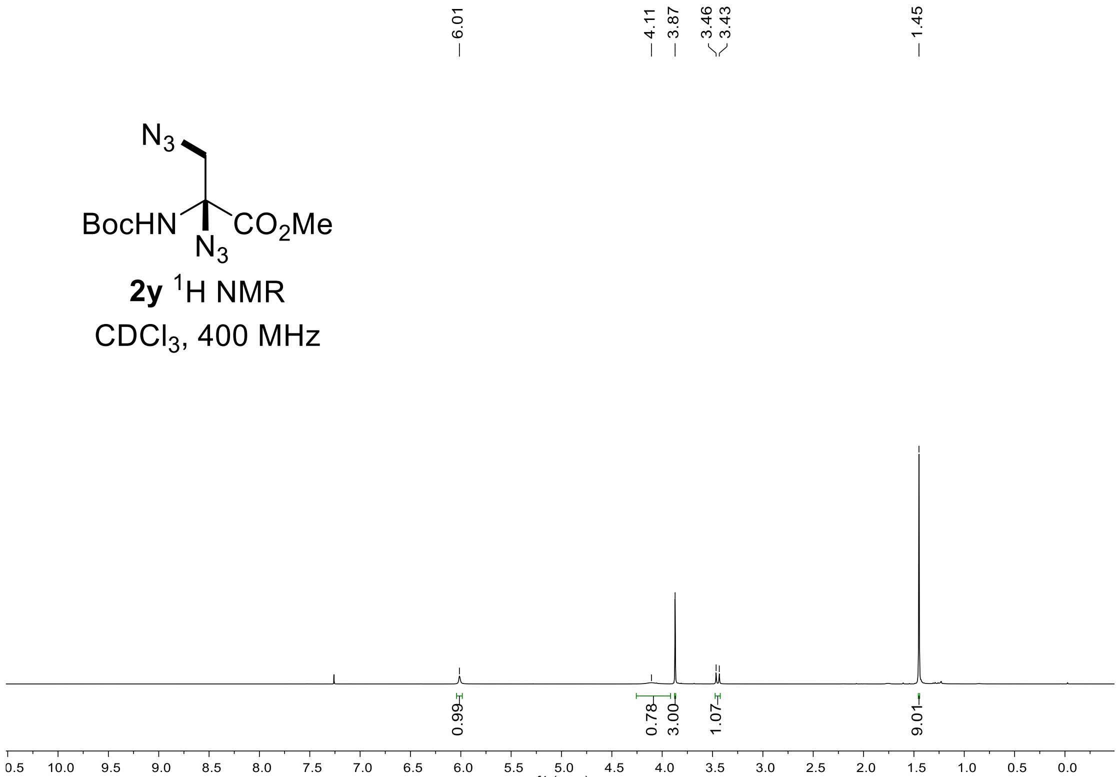
6.01
4.11
3.87
3.46
3.43
1.45
N_3
BocHN
CO_2Me
N_3
2y ^1H NMR
$CDCl_3$, 400 MHz
0.99
0.78
3.00
1.07
9.01
f1 (ppm)

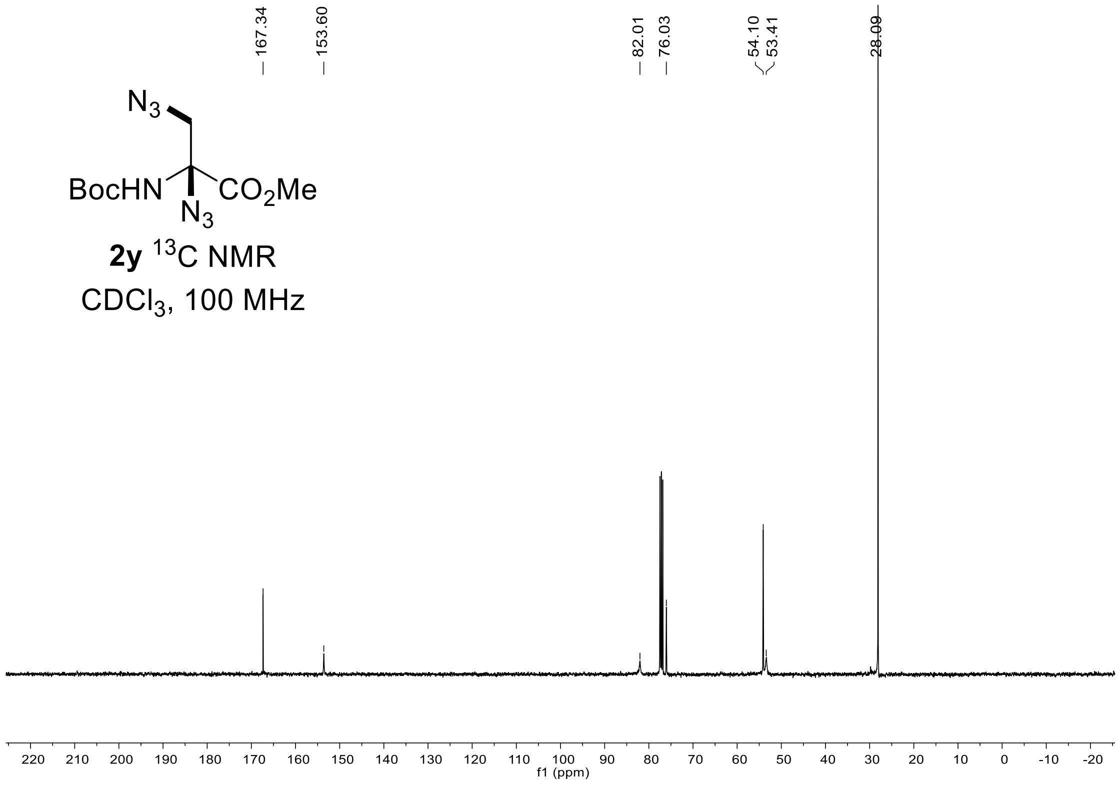
167.34
153.60
82.01
76.03
54.10
53.41
28.09
N_3
BocHN
CO_2Me
N_3
2y ^{13}C NMR
$CDCl_3$, 100 MHz
f1 (ppm)

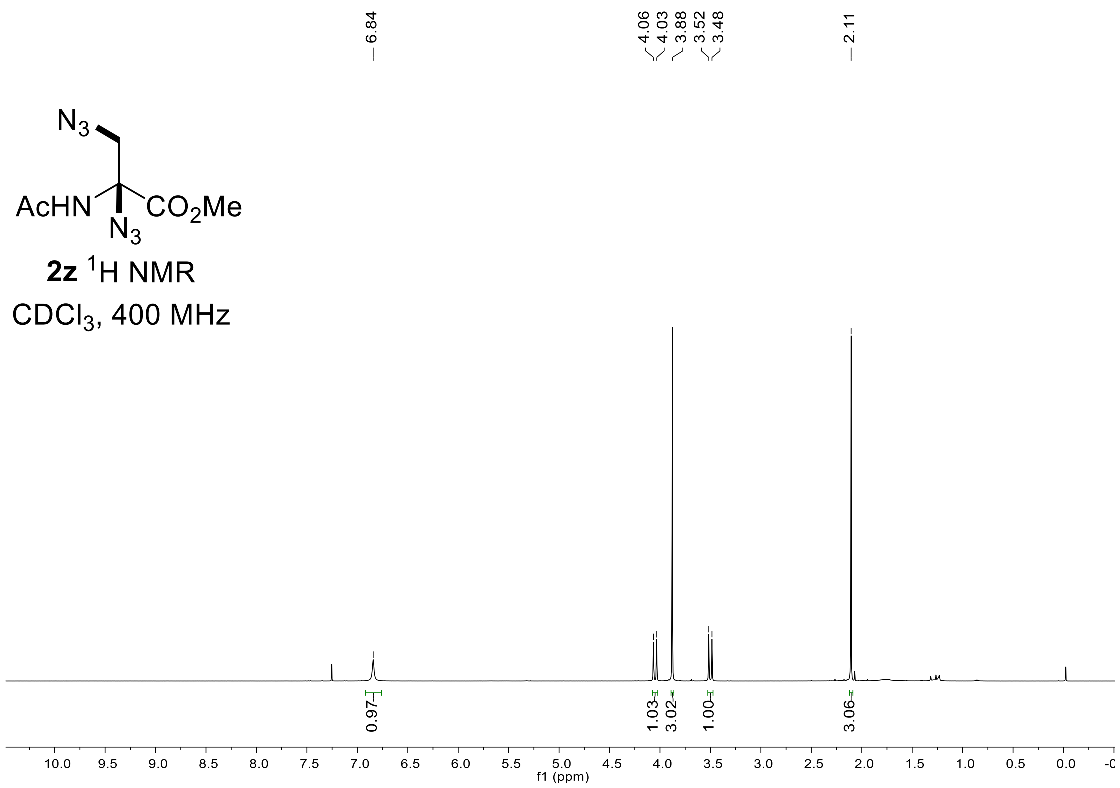
6.84
4.06
4.03
3.88
3.52
3.48
2.11
N_3
AcHN
CO_2Me
N_3
2z ^{1}H NMR
$CDCl_3$, 400 MHz
0.97
1.03
3.02
1.00
3.06
10.0 9.5 9.0 8.5 8.0 7.5 7.0 6.5 6.0 5.5 5.0 4.5 4.0 3.5 3.0 2.5 2.0 1.5 1.0 0.5 0.0
f1 (ppm)

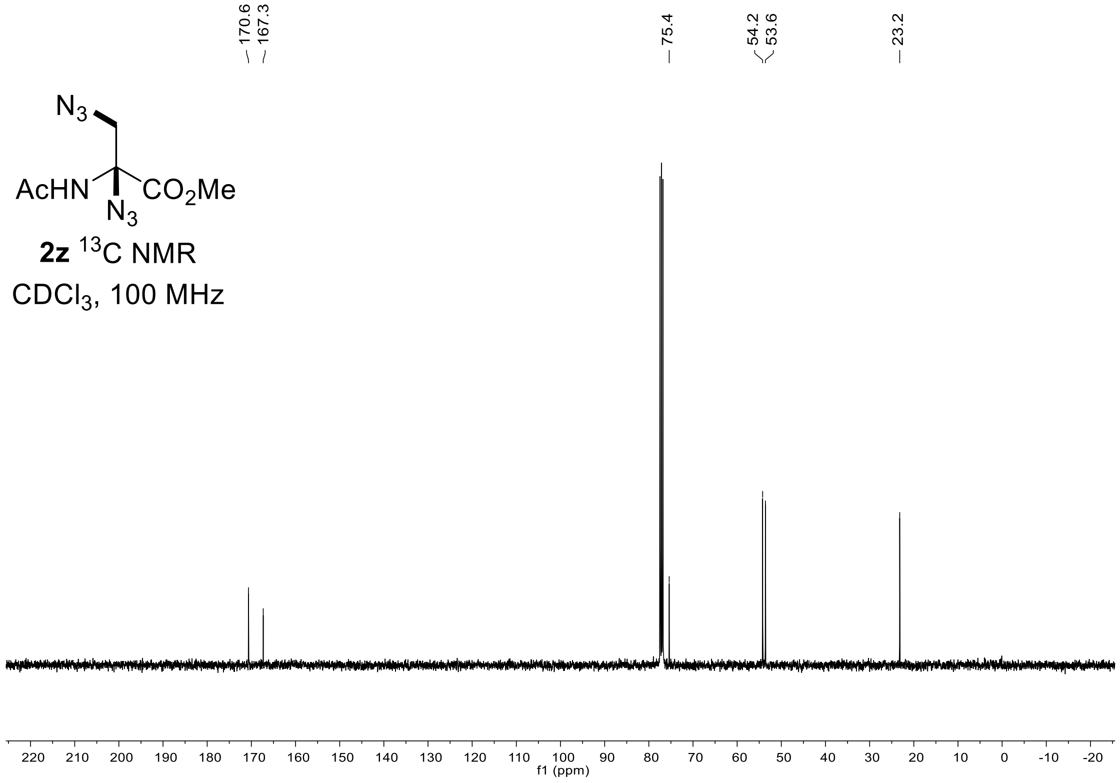
170.6
167.3
75.4
54.2
53.6
23.2
N_3
AcHN
CO_2Me
N_3
2z ^{13}C NMR
$CDCl_3$, 100 MHz
220 210 200 190 180 170 160 150 140 130 120 110 100 90 80 70 60 50 40 30 20 10 0 -10 -20
f1 (ppm)

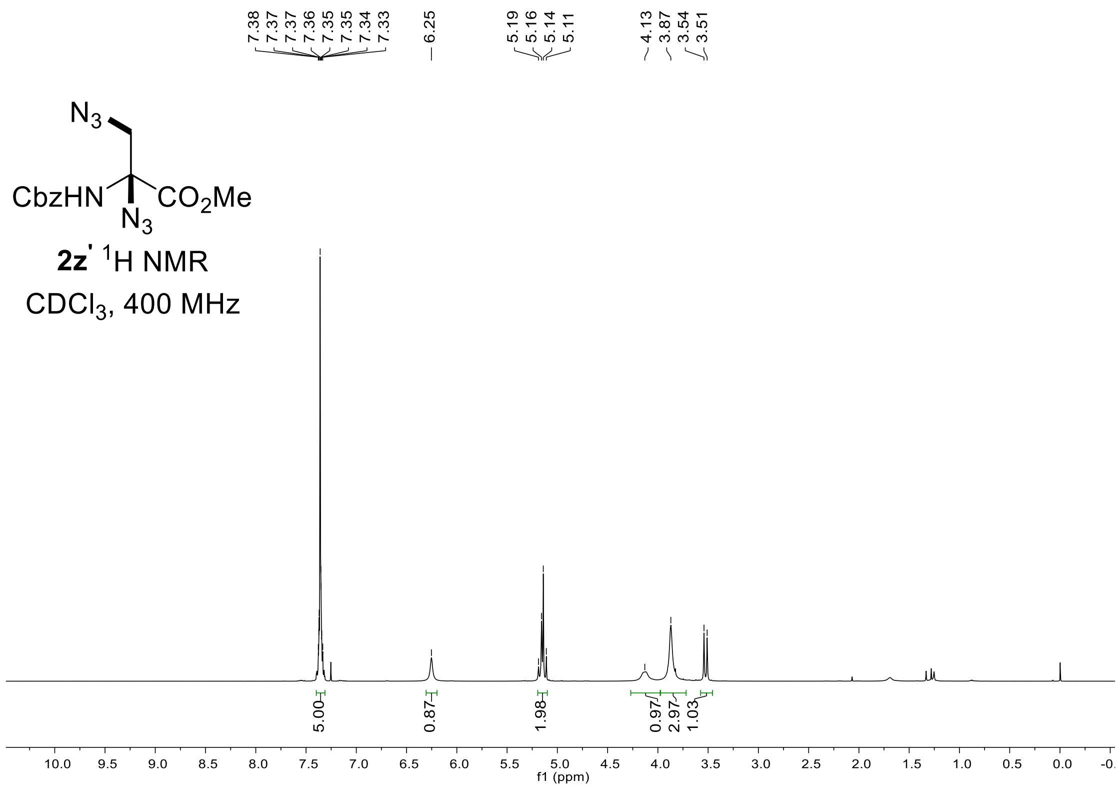
7.38
7.37
7.37
7.36
7.35
7.35
7.34
7.33
6.25
5.19
5.16
5.14
5.11
4.13
3.87
3.54
3.51
N3
CbzHN
CO2Me
N3
2z' 1H NMR
CDCl3, 400 MHz
5.00
0.87
1.98
0.97
2.97
1.03
f1 (ppm)

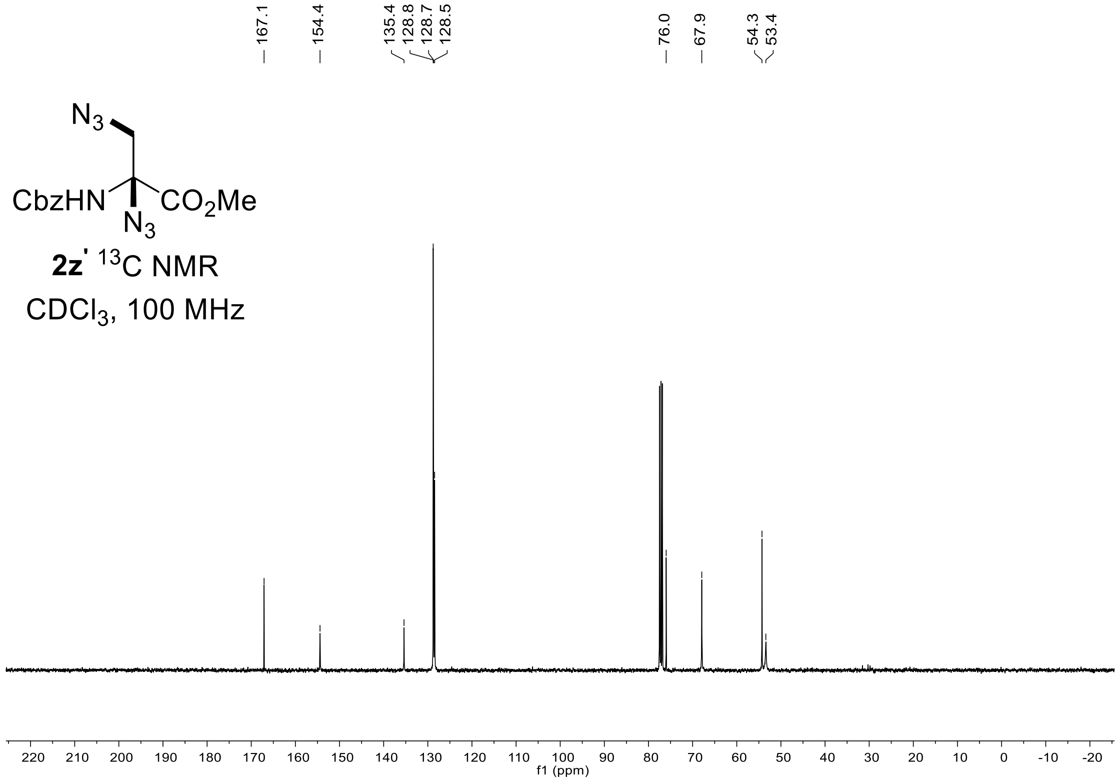
167.1
154.4
135.4
128.8
128.7
128.5
76.0
67.9
54.3
53.4
N3
CbzHN
CO2Me
N3
2z' 13C NMR
CDCl3, 100 MHz
f1 (ppm)

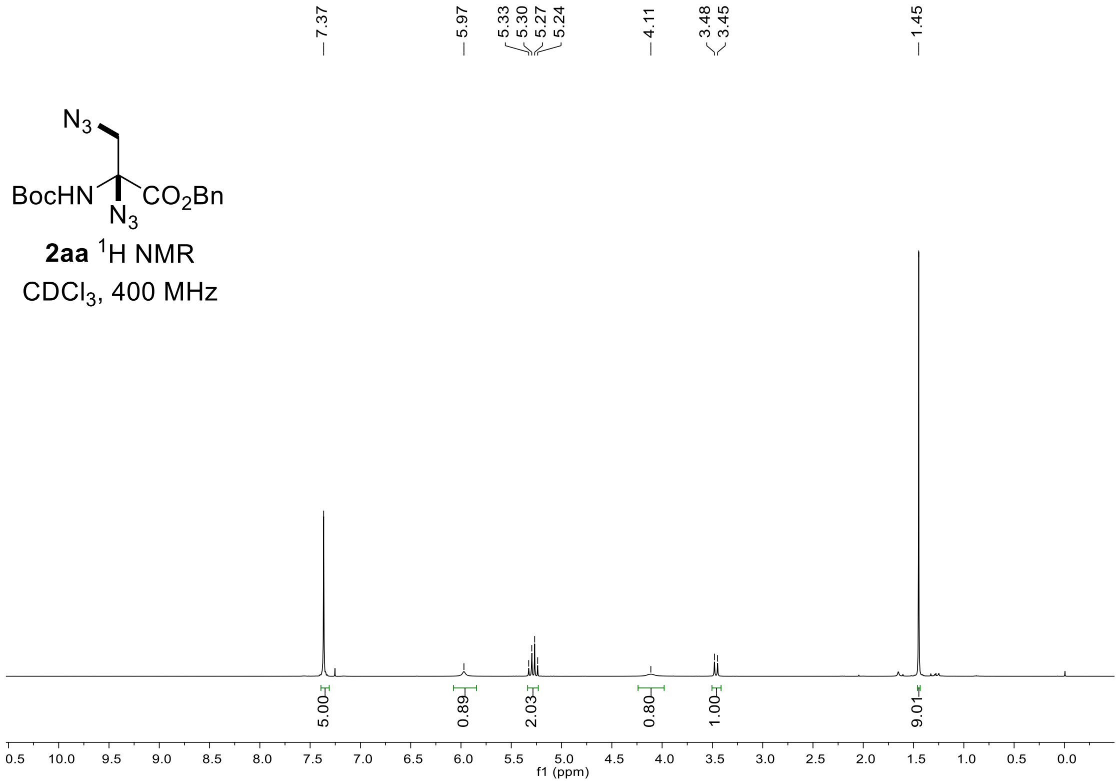

2aa ^{1}H NMR
$CDCl_3$, 400 MHz

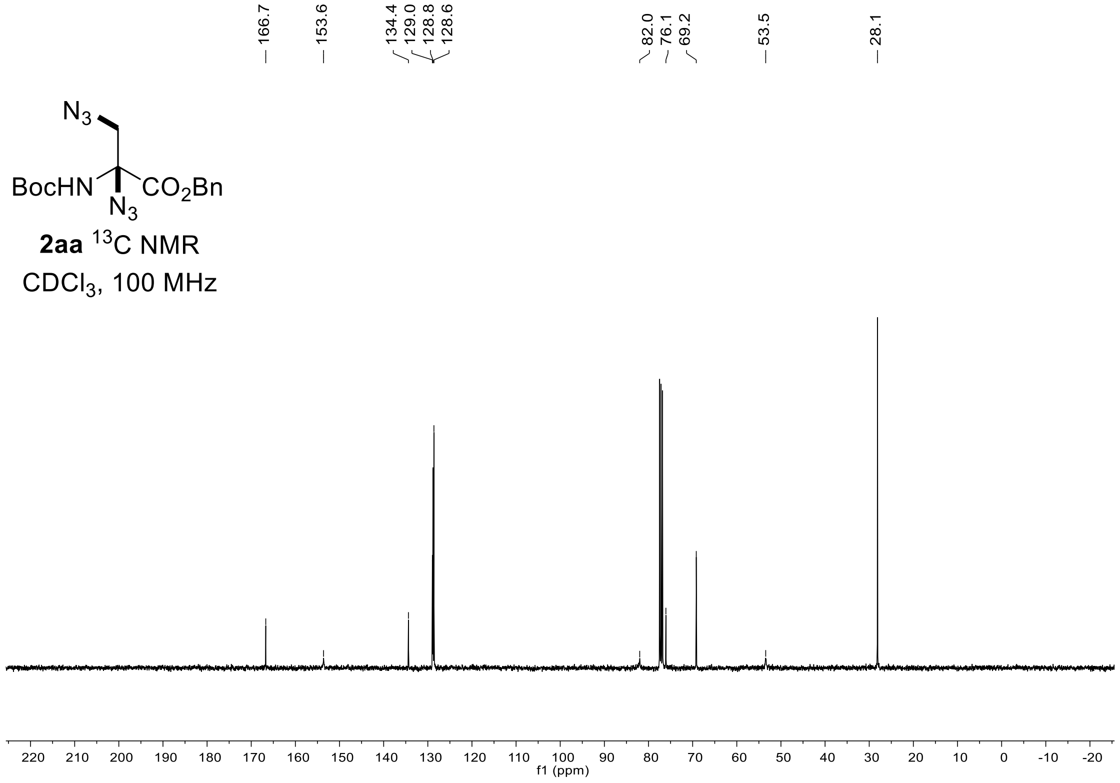

2aa ^{13}C NMR
$CDCl_3$, 100 MHz

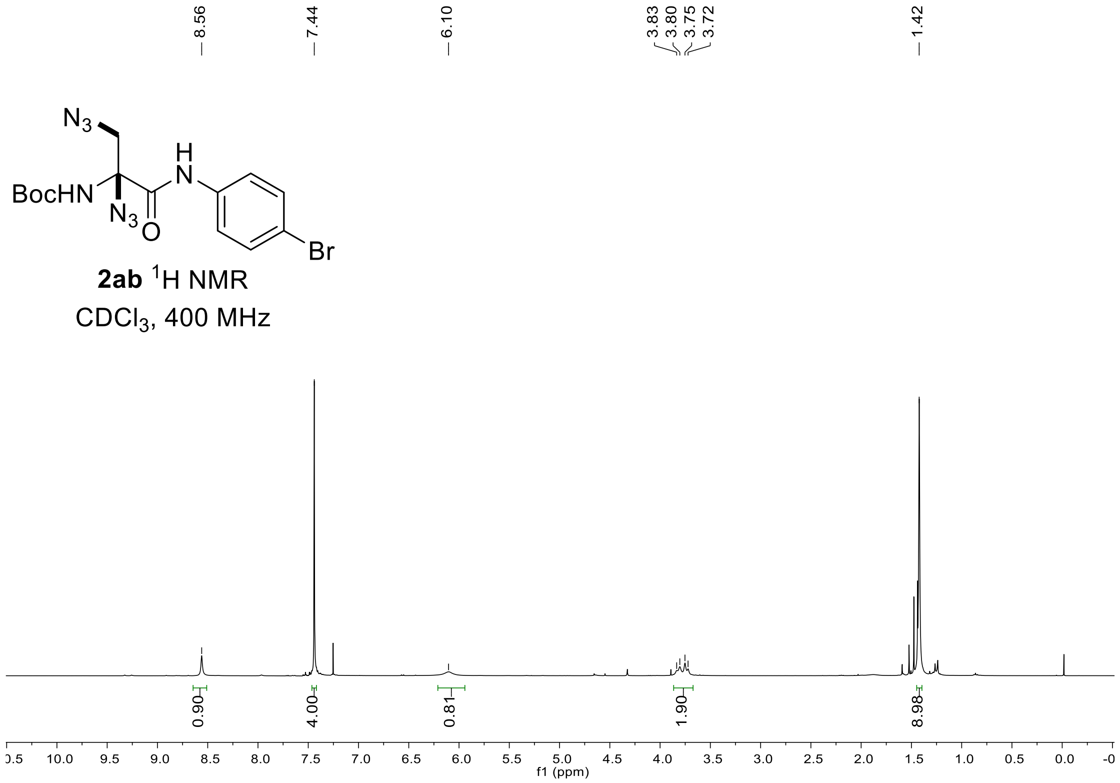

8.56
7.44
6.10
3.83
3.80
3.75
3.72
1.42
N3
H
N
BocHN
N3
O
Br
2ab 1H NMR
CDCl3, 400 MHz
0.90
4.00
0.81
1.90
8.98
0.5 10.0 9.5 9.0 8.5 8.0 7.5 7.0 6.5 6.0 5.5 5.0 4.5 4.0 3.5 3.0 2.5 2.0 1.5 1.0 0.5 0.0 -0
f1 (ppm)

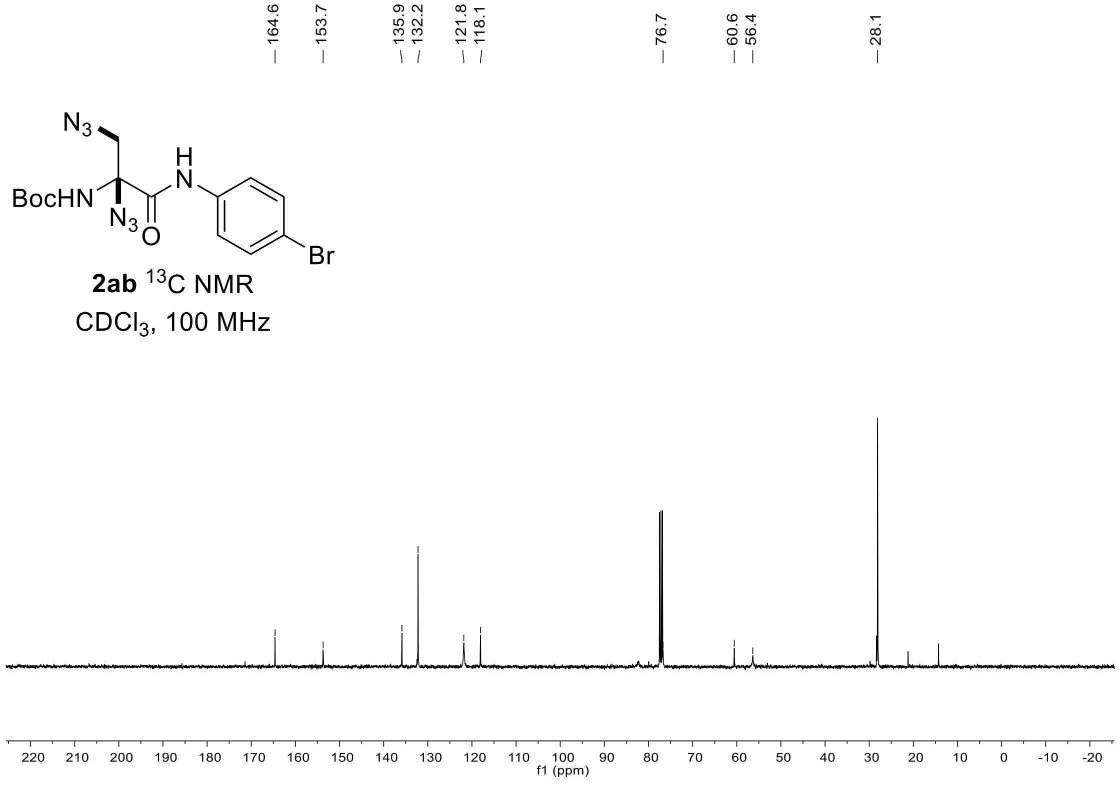

164.6
153.7
135.9
132.2
121.8
118.1
76.7
60.6
56.4
28.1
N3
H
N
BocHN
N3
O
Br
2ab 13C NMR
CDCl3, 100 MHz
220 210 200 190 180 170 160 150 140 130 120 110 100 90 80 70 60 50 40 30 20 10 0 -10 -20
f1 (ppm)

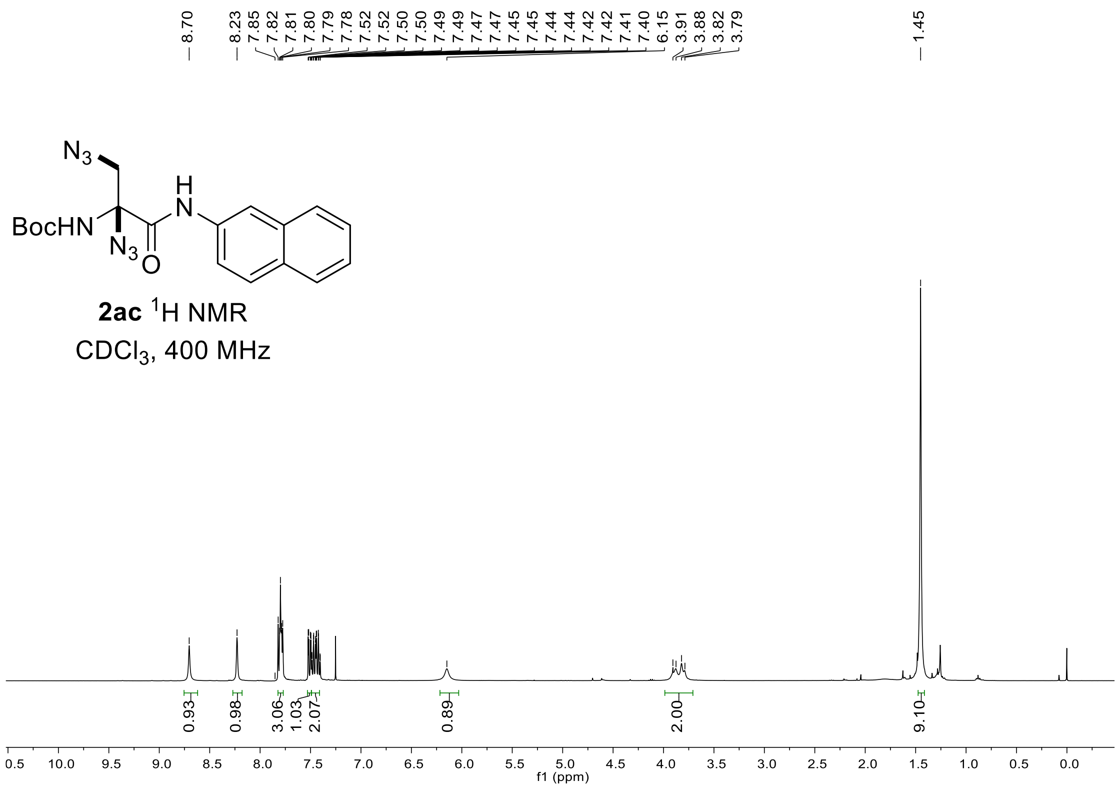

2ac ^{1}H NMR

$CDCl_3$, 400 MHz

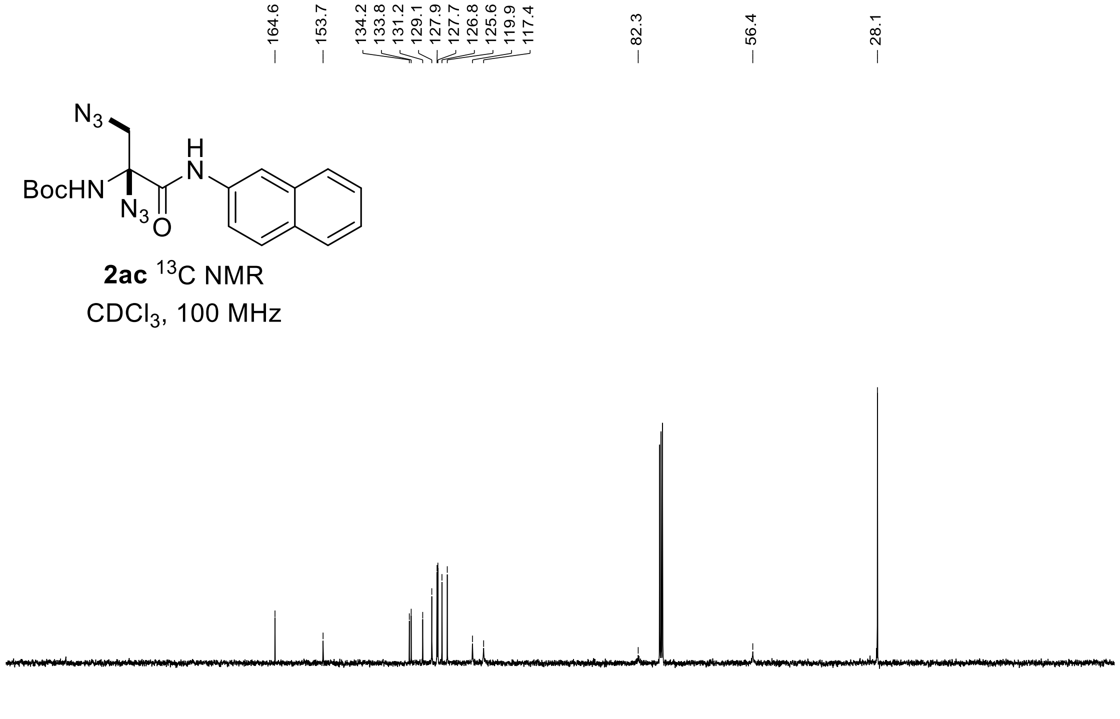

2ac ^{13}C NMR

$CDCl_3$, 100 MHz

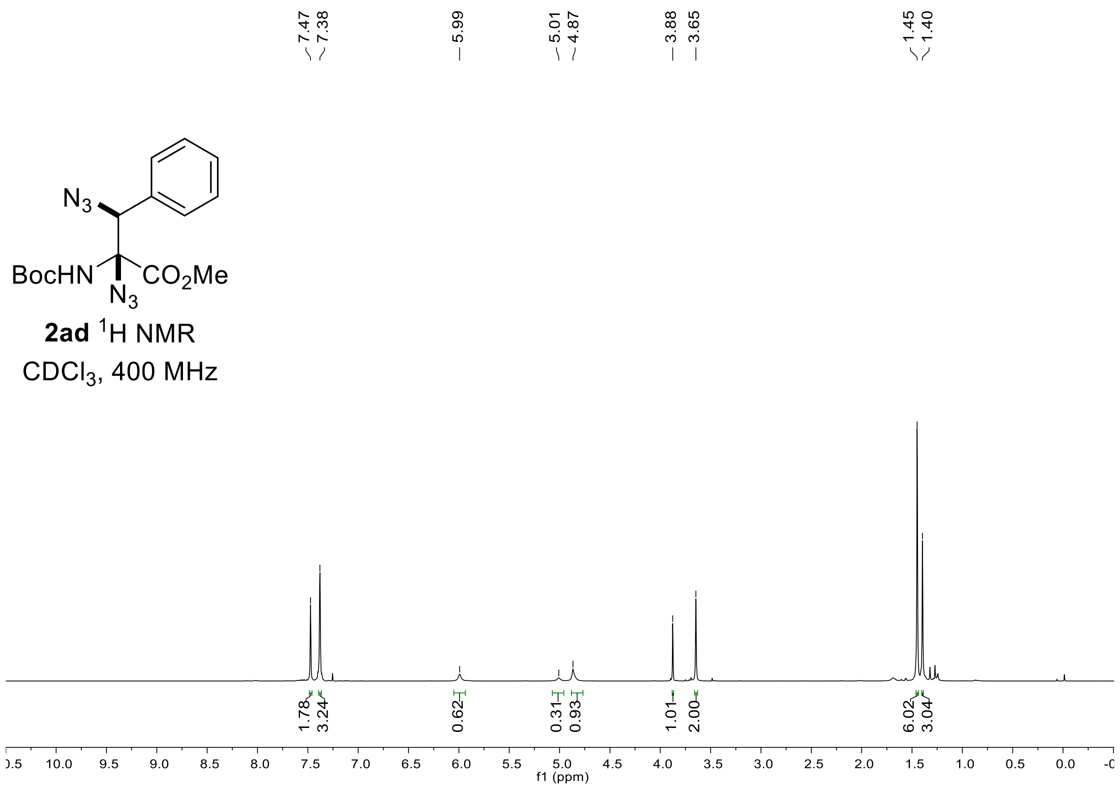
7.47
7.38
5.99
5.01
4.87
3.88
3.65
1.45
1.40
N3
BocHN
CO2Me
N3
2ad 1H NMR
CDCl3, 400 MHz
1.78
3.24
0.62
0.31
0.93
1.01
2.00
6.02
3.04
f1 (ppm)

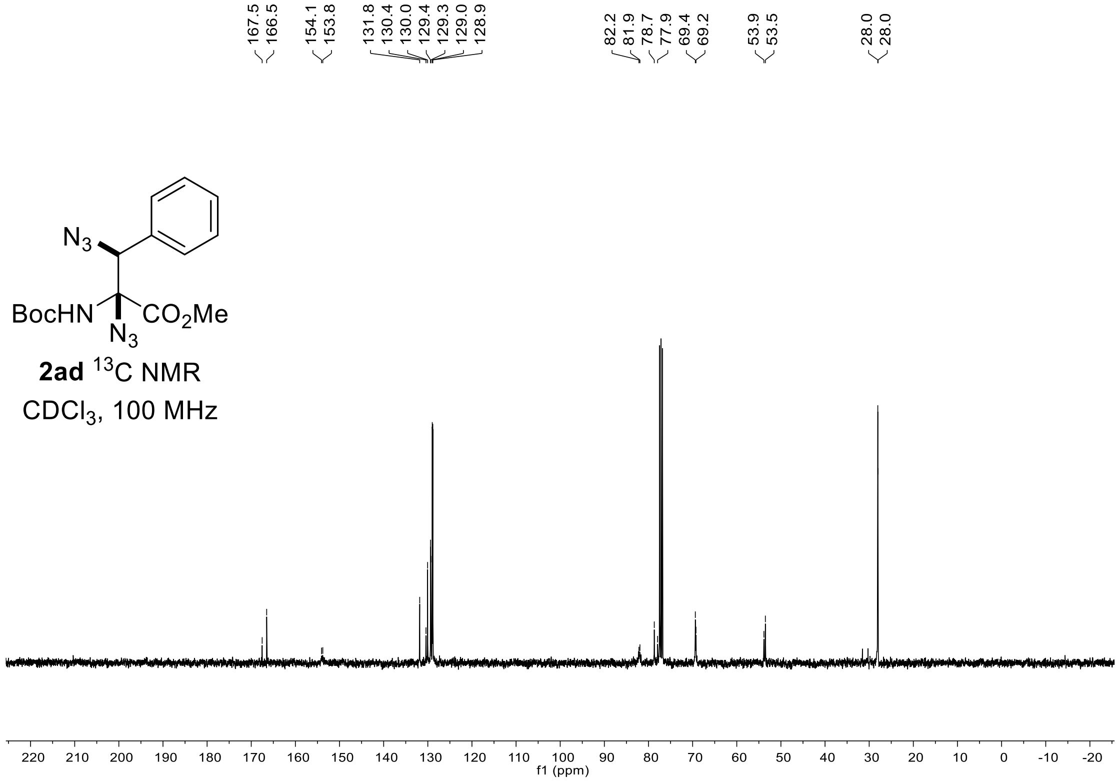
167.5
166.5
154.1
153.8
131.8
130.4
130.0
129.4
129.3
129.0
128.9
82.2
81.9
78.7
77.9
69.4
69.2
53.9
53.5
28.0
28.0
N3
BocHN
CO2Me
N3
2ad 13C NMR
CDCl3, 100 MHz
f1 (ppm)

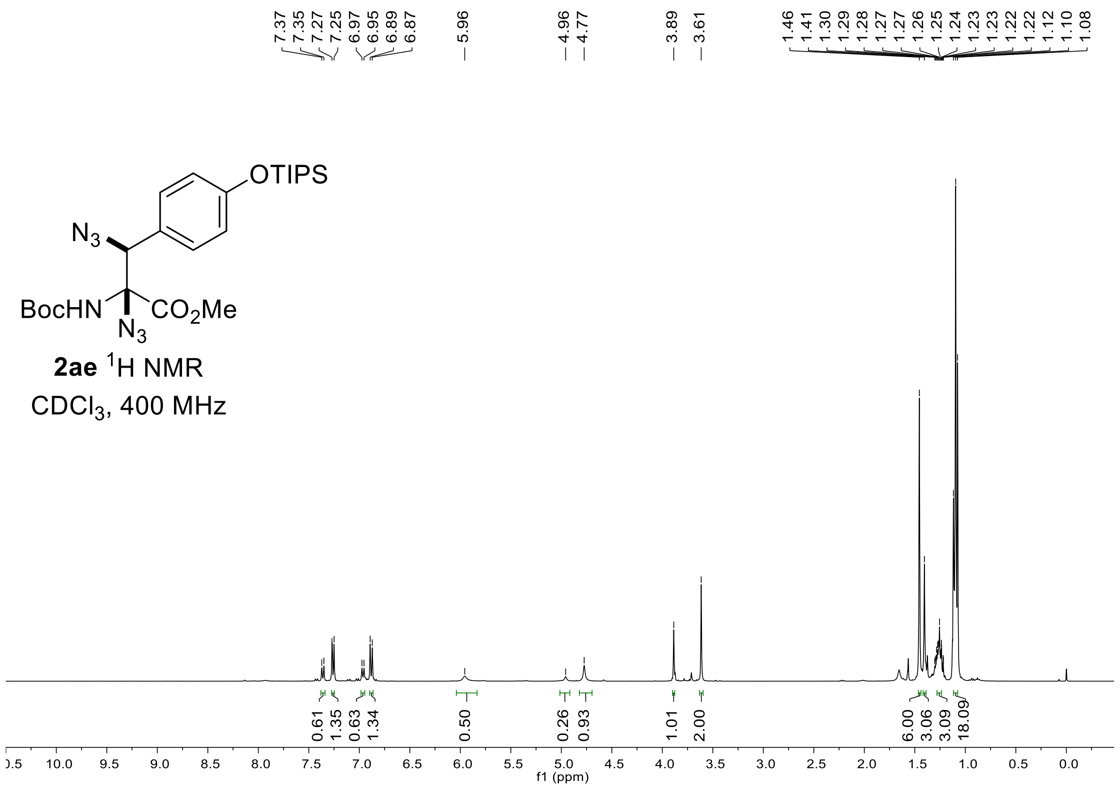
OTIPS
N_3
BocHN
CO_2Me
N_3
2ae ^{1}H NMR
$CDCl_3$, 400 MHz
7.37 7.35 7.27 7.25 6.97 6.95 6.89 6.87
5.96
4.96 4.77
3.89 3.61
1.46 1.41 1.30 1.29 1.28 1.27 1.27 1.26 1.25 1.24 1.23 1.23 1.22 1.22 1.12 1.10 1.08
0.61 1.35 0.63 1.34 0.50 0.26 0.93 1.01 2.00 6.00 3.06 3.09 18.09
f1 (ppm)

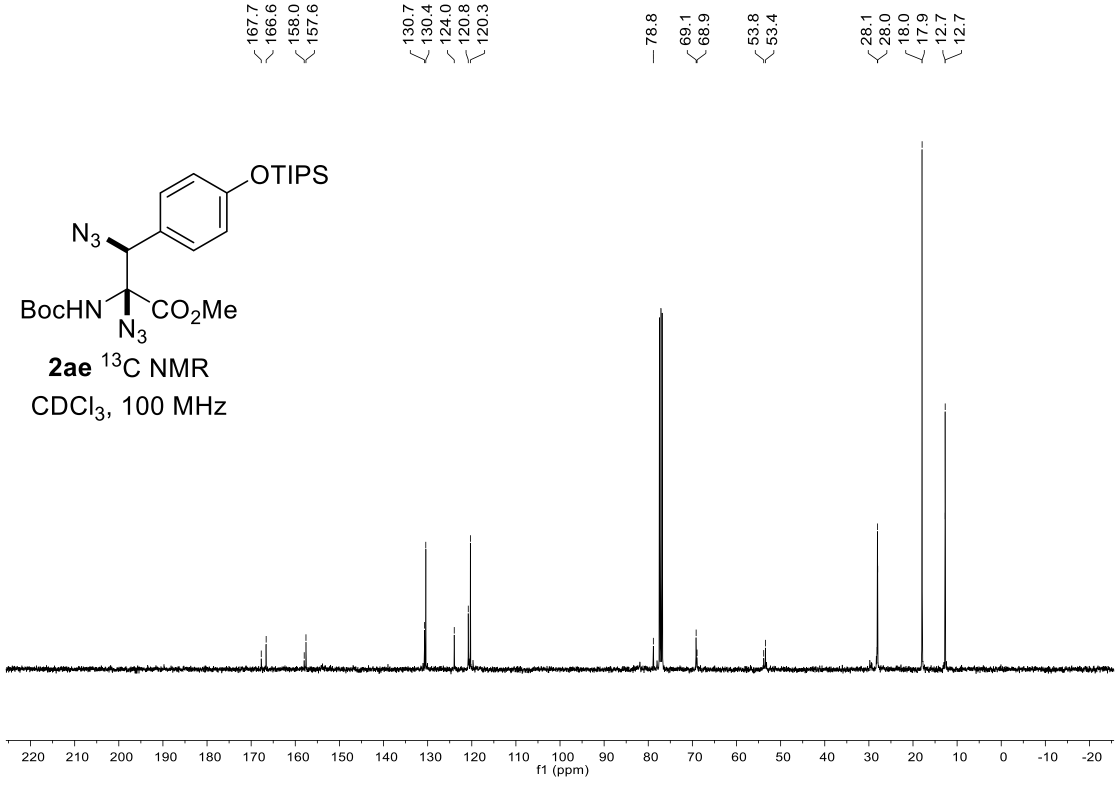
OTIPS
N_3
BocHN
CO_2Me
N_3
2ae ^{13}C NMR
$CDCl_3$, 100 MHz
167.7 166.6 158.0 157.6
130.7 130.4 124.0 120.8 120.3
78.8
69.1 68.9
53.8 53.4
28.1 28.0 18.0 17.9 12.7 12.7
f1 (ppm)

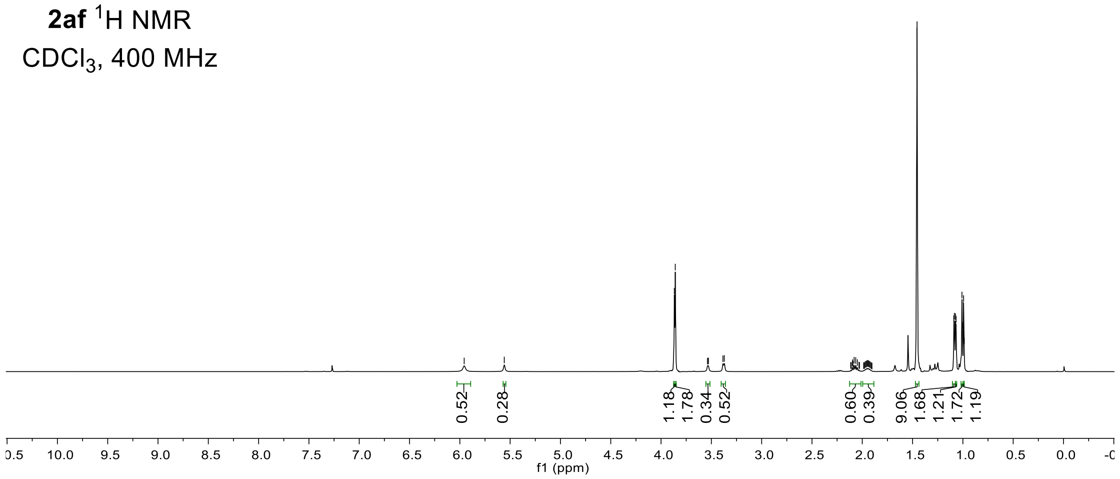

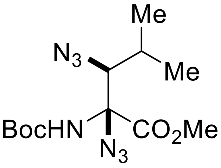

2af ^{1}H NMR

$CDCl_3$, 400 MHz

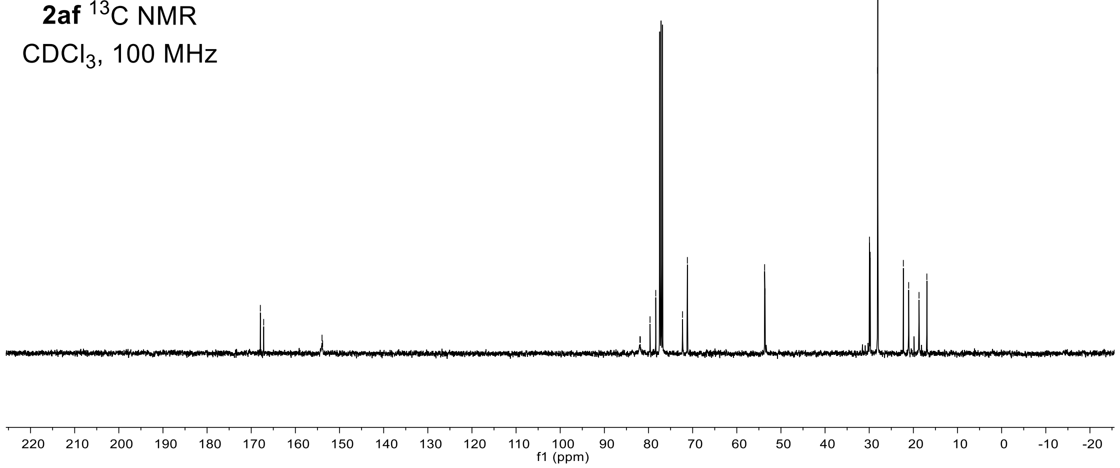

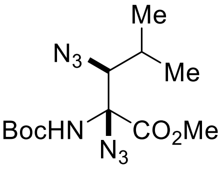

2af ^{13}C NMR

$CDCl_3$, 100 MHz

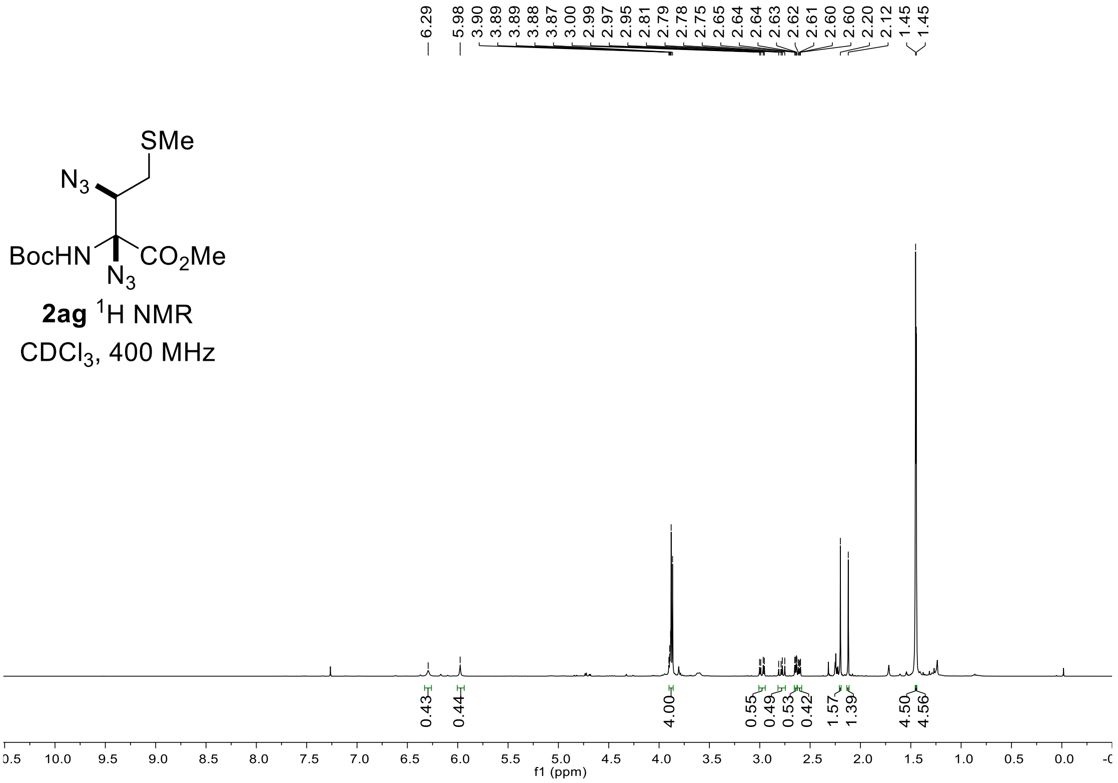
6.29
5.98
3.90 3.89 3.89 3.88 3.87 3.00 2.99 2.97 2.95 2.81 2.79 2.78 2.75 2.65 2.64 2.64 2.63 2.62 2.61 2.60 2.60 2.20 2.12 1.45 1.45
SMe
N3
BocHN
CO2Me
N3
2ag 1H NMR
CDCl3, 400 MHz
0.43 0.44 4.00 0.55 0.49 0.53 0.42 1.57 1.39 4.50 4.56
f1 (ppm)

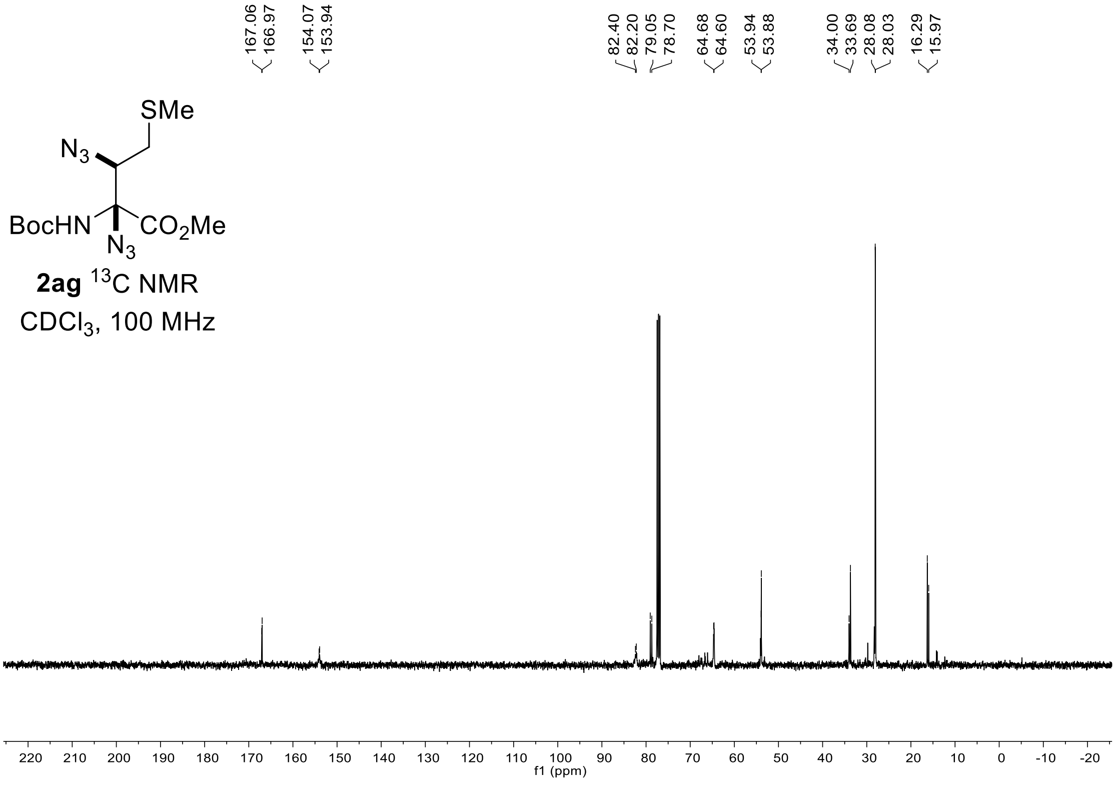
167.06 166.97 154.07 153.94 82.40 82.20 79.05 78.70 64.68 64.60 53.94 53.88 34.00 33.69 28.08 28.03 16.29 15.97
SMe
N3
BocHN
CO2Me
N3
2ag 13C NMR
CDCl3, 100 MHz
f1 (ppm)

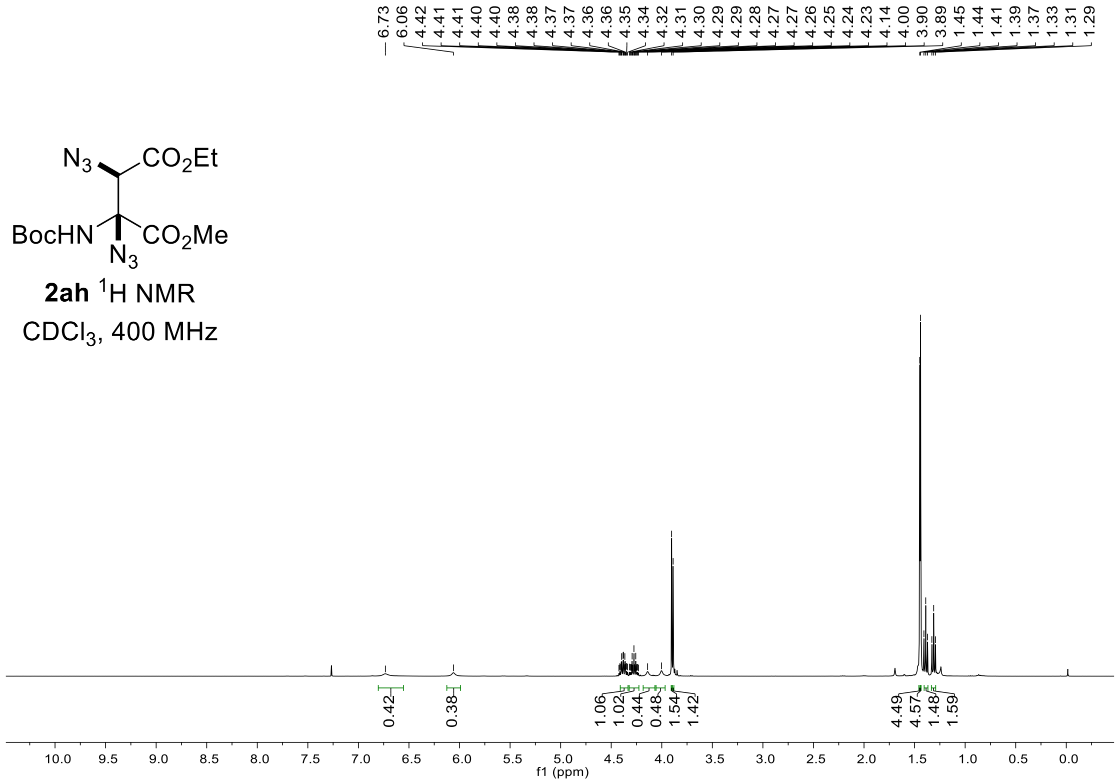

2ah ^{1}H NMR
CDCl$_3$, 400 MHz
f1 (ppm)

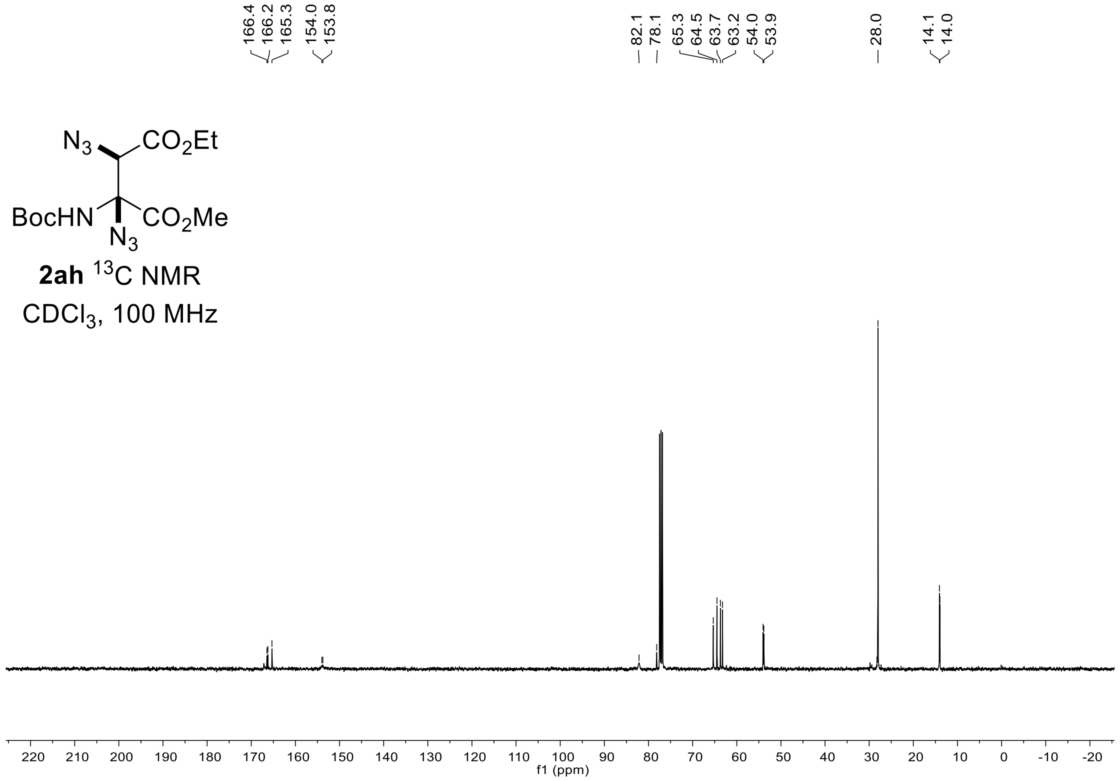

2ah ^{13}C NMR
CDCl$_3$, 100 MHz
f1 (ppm)

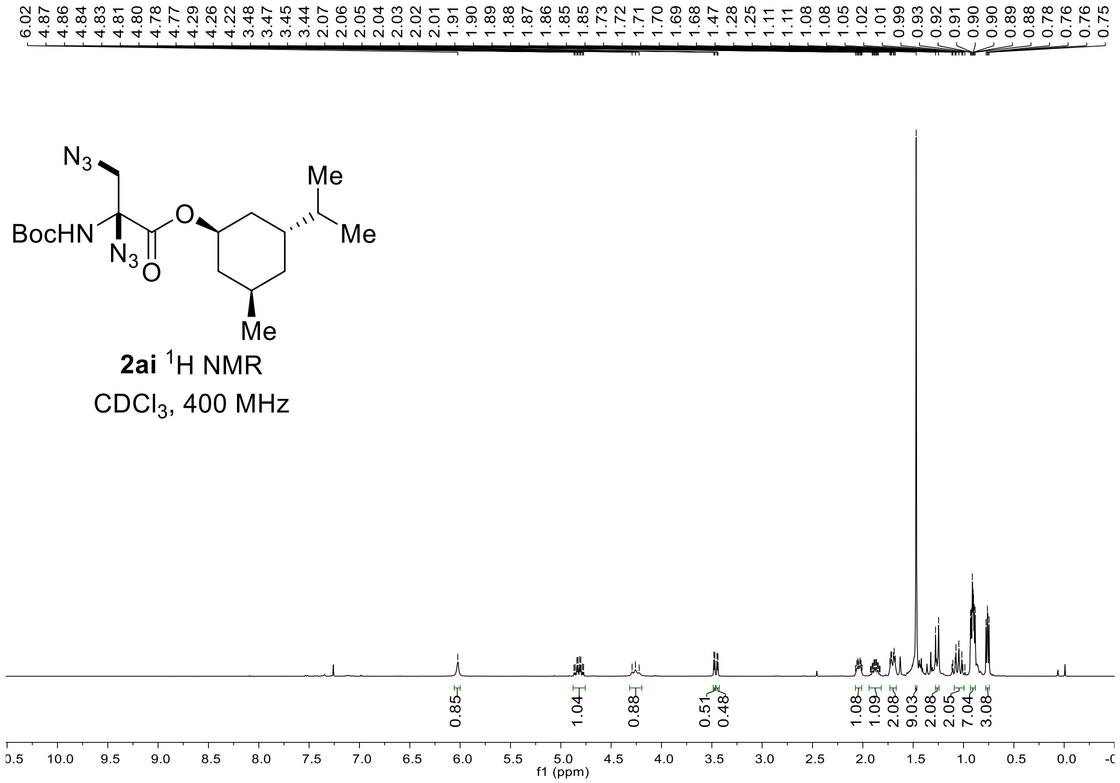

2ai ^{1}H NMR

$CDCl_3$, 400 MHz

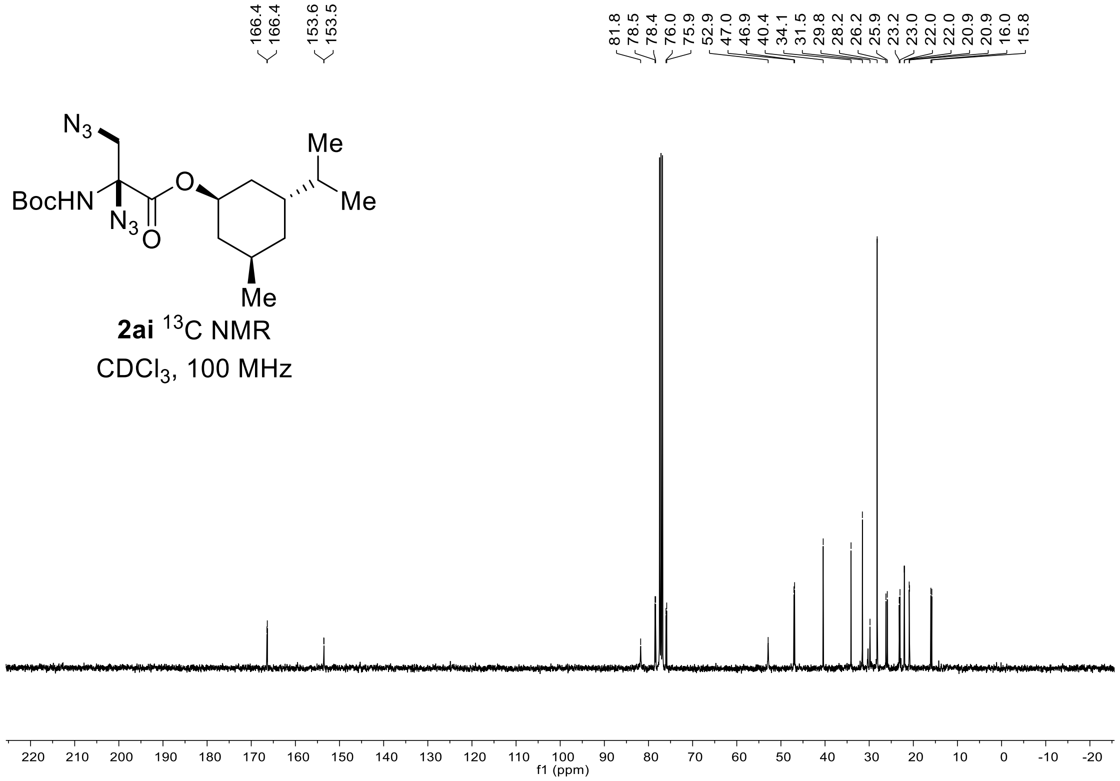

2ai ^{13}C NMR

$CDCl_3$, 100 MHz

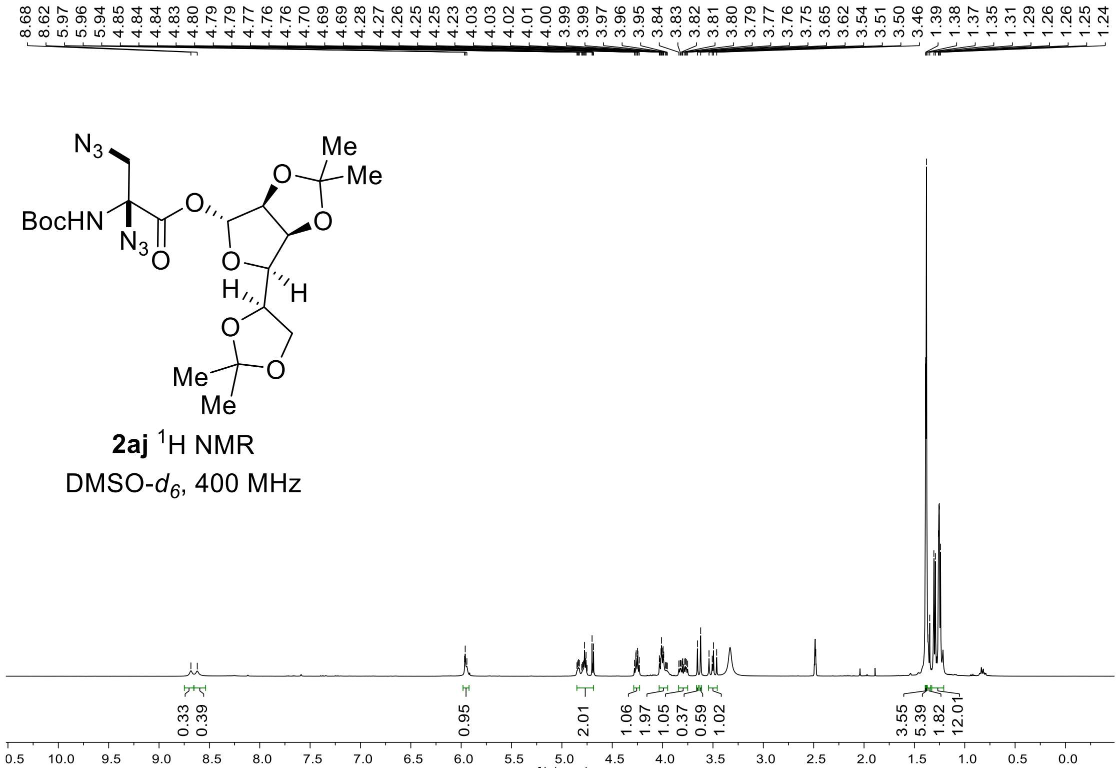
8.68 8.62 5.97 5.96 5.94 4.85 4.84 4.84 4.83 4.80 4.79 4.77 4.76 4.70 4.69 4.69 4.28 4.27 4.26 4.25 4.25 4.23 4.03 4.03 4.02 4.01 4.00 3.99 3.99 3.97 3.96 3.95 3.84 3.83 3.82 3.81 3.80 3.79 3.77 3.76 3.75 3.65 3.62 3.54 3.51 3.50 3.46 1.39 1.38 1.37 1.35 1.31 1.29 1.26 1.25 1.24
N3
Me
Me
BocHN
N3
O
H
H
Me
Me
2aj 1H NMR
DMSO-d6, 400 MHz
0.33 0.39 0.95 2.01 1.06 1.97 1.05 0.37 0.59 1.02 3.55 5.39 1.82 12.01
0.5 10.0 9.5 9.0 8.5 8.0 7.5 7.0 6.5 6.0 5.5 5.0 4.5 4.0 3.5 3.0 2.5 2.0 1.5 1.0 0.5 0.0
f1 (ppm)

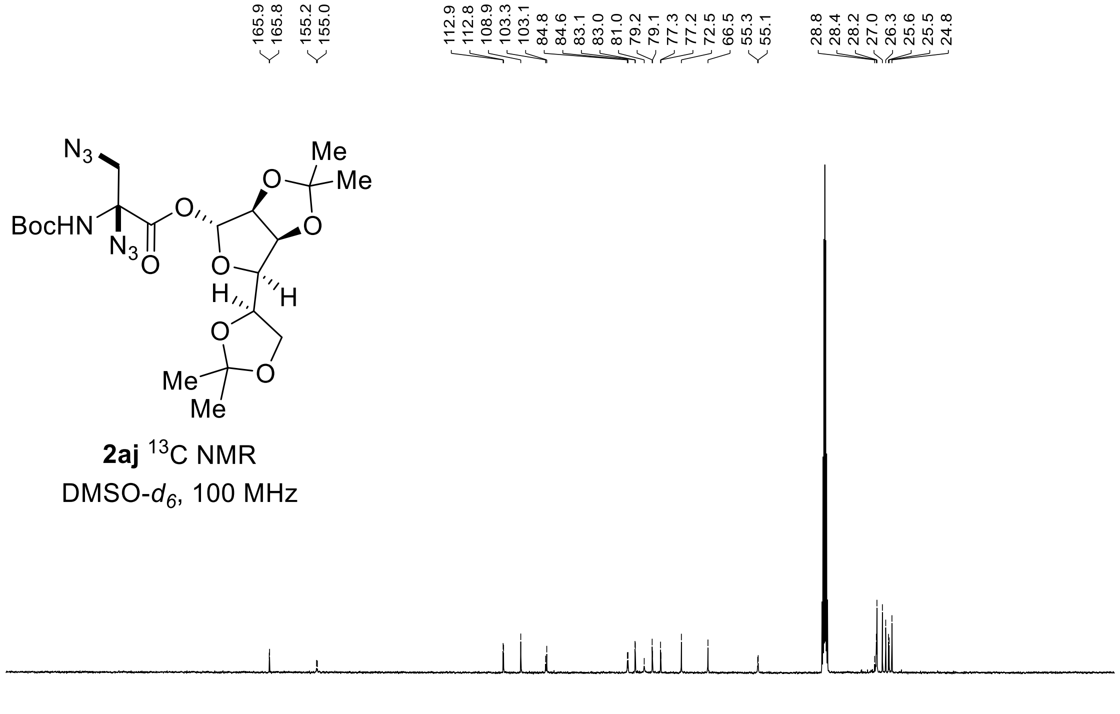
165.9 165.8 155.2 155.0 112.9 112.8 108.9 103.3 103.1 84.8 84.6 83.1 83.0 81.0 79.2 79.1 77.3 77.2 72.5 66.5 55.3 55.1 28.8 28.4 28.2 27.0 26.3 25.6 25.5 24.8
N3
Me
Me
BocHN
N3
O
H
H
Me
Me
2aj 13C NMR
DMSO-d6, 100 MHz
220 210 200 190 180 170 160 150 140 130 120 110 100 90 80 70 60 50 40 30 20 10 0 -10 -20
f1 (ppm)

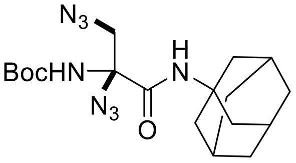

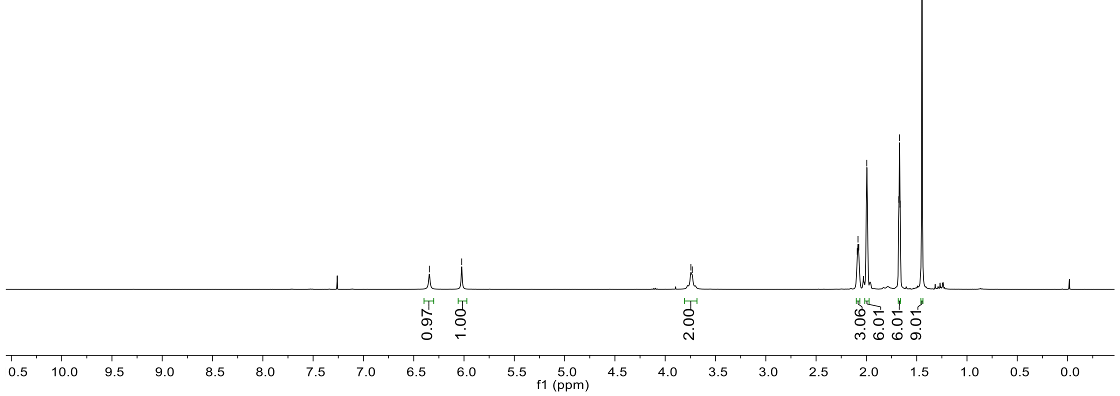

2ak ^{1}H NMR
$CDCl_3$, 400 MHz

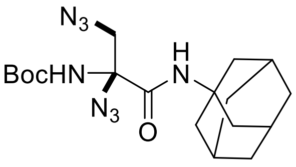

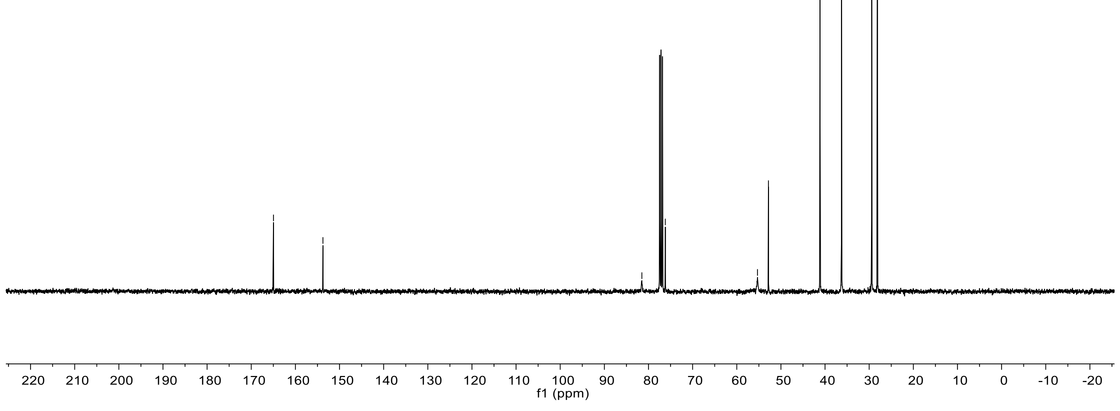

2ak ^{13}C NMR
$CDCl_3$, 100 MHz

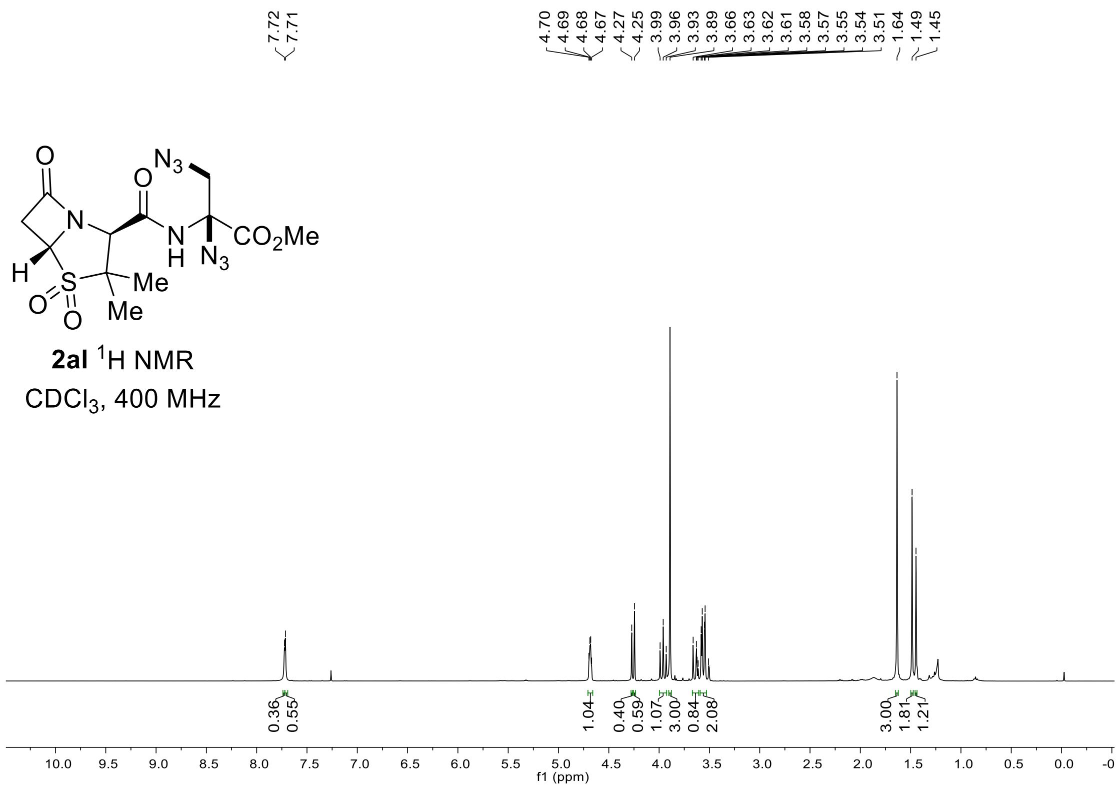
7.72
7.71
4.70
4.69
4.68
4.67
4.27
4.25
3.99
3.96
3.93
3.89
3.66
3.63
3.62
3.61
3.58
3.57
3.55
3.54
3.51
1.64
1.49
1.45
2al 1H NMR
CDCl3, 400 MHz
0.36
0.55
1.04
0.40
0.59
1.07
3.00
0.84
2.08
3.00
1.81
1.21
f1 (ppm)

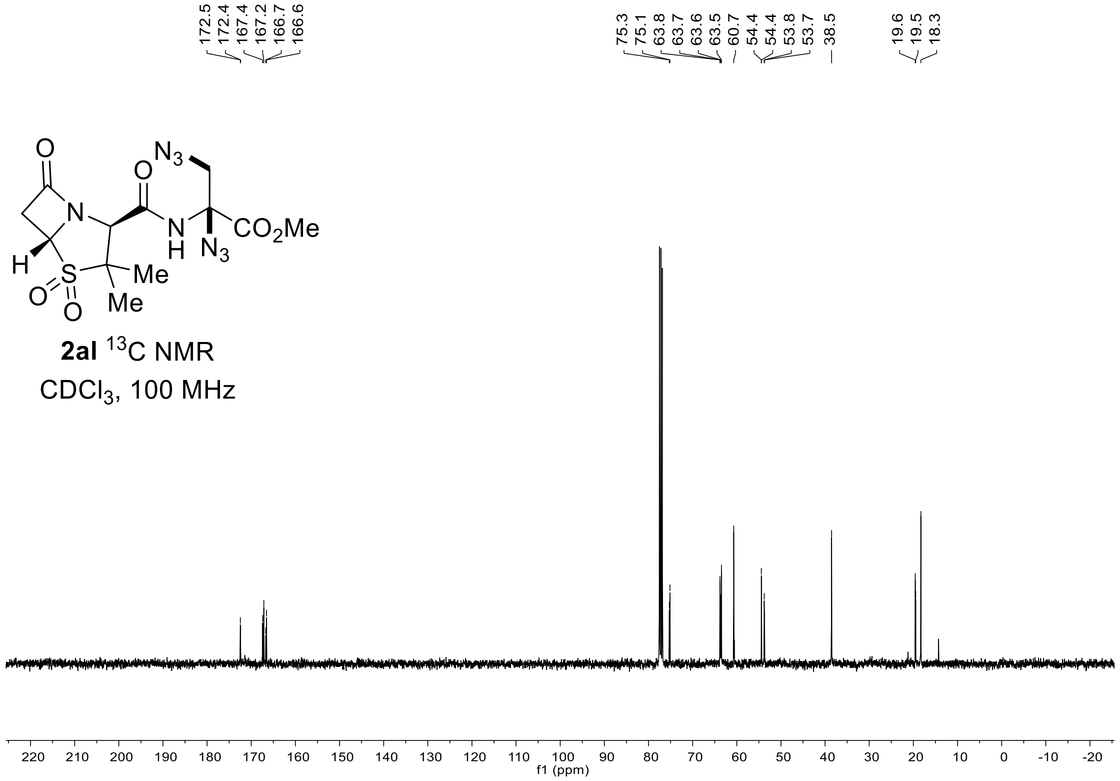
172.5
172.4
167.4
167.2
166.7
166.6
75.3
75.1
63.8
63.7
63.6
63.5
60.7
54.4
54.4
53.8
53.7
38.5
19.6
19.5
18.3
2al 13C NMR
CDCl3, 100 MHz
f1 (ppm)

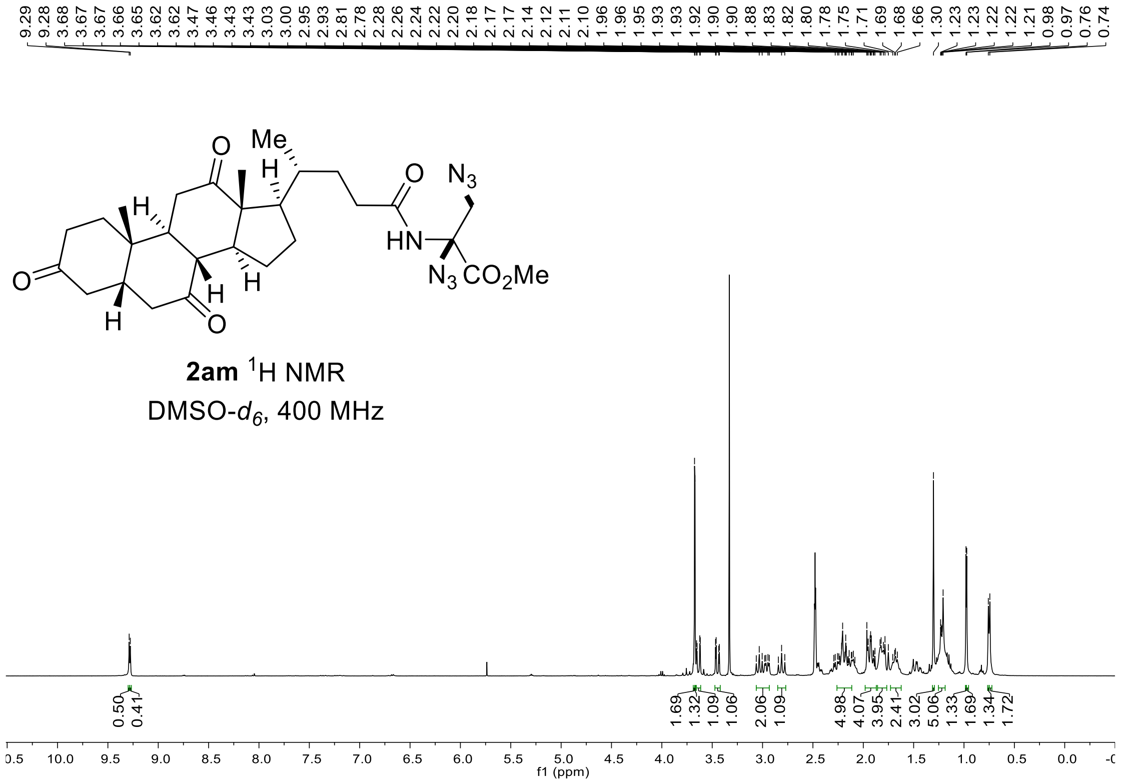

9.29 9.28 3.68 3.67 3.67 3.66 3.65 3.62 3.62 3.47 3.46 3.43 3.43 3.03 3.00 2.95 2.93 2.81 2.78 2.28 2.26 2.24 2.22 2.20 2.18 2.17 2.17 2.14 2.12 2.11 2.10 1.96 1.96 1.95 1.93 1.93 1.92 1.90 1.90 1.88 1.83 1.82 1.80 1.78 1.75 1.71 1.69 1.68 1.66 1.30 1.23 1.23 1.22 1.22 1.21 0.98 0.97 0.76 0.74
Me
O
H
N3
HN
N3 CO2Me
2am 1H NMR
DMSO-d6, 400 MHz
0.50 0.41 1.69 1.32 1.09 1.06 2.06 1.09 4.98 4.07 3.95 2.41 3.02 5.06 1.33 1.69 1.34 1.72
10.0 9.5 9.0 8.5 8.0 7.5 7.0 6.5 6.0 5.5 5.0 4.5 4.0 3.5 3.0 2.5 2.0 1.5 1.0 0.5 0.0
f1 (ppm)

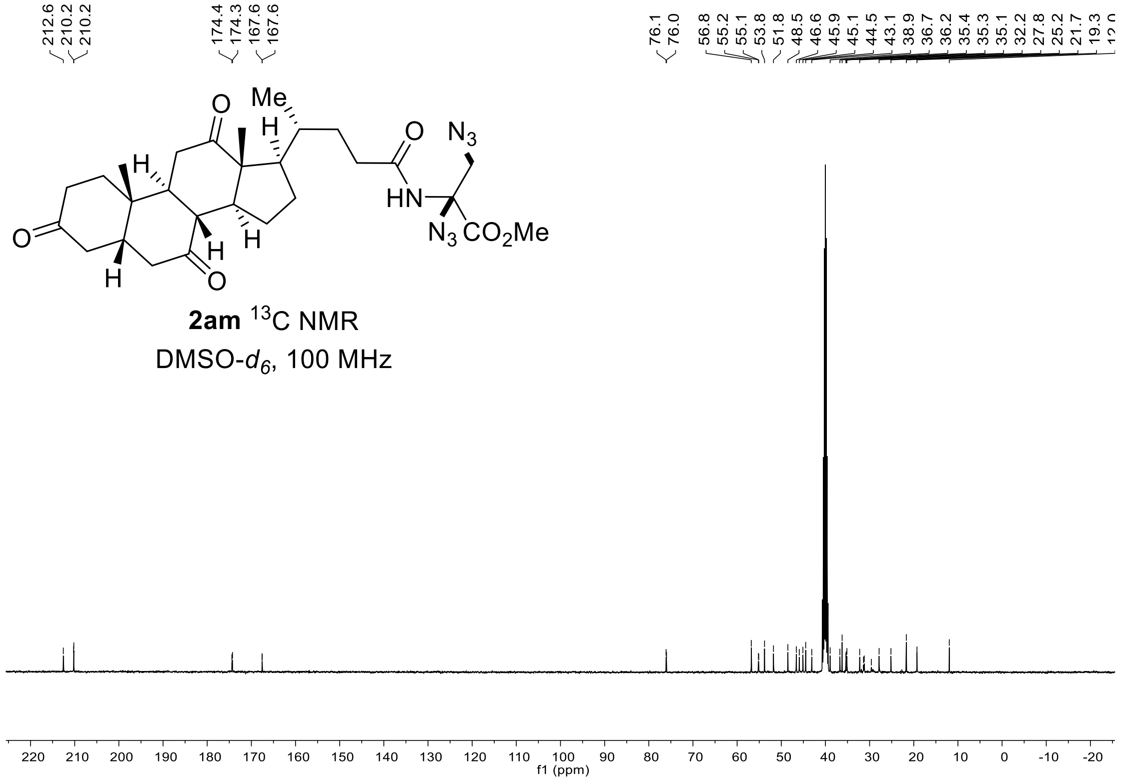

212.6 210.2 210.2 174.4 174.3 167.6 167.6 76.1 76.0 56.8 55.2 55.1 53.8 51.8 48.5 46.6 45.9 45.1 44.5 43.1 38.9 36.7 36.2 35.4 35.3 35.1 32.2 27.8 25.2 21.7 19.3
Me
O
H
N3
HN
N3 CO2Me
2am 13C NMR
DMSO-d6, 100 MHz
220 210 200 190 180 170 160 150 140 130 120 110 100 90 80 70 60 50 40 30 20 10 0 -10 -20
f1 (ppm)

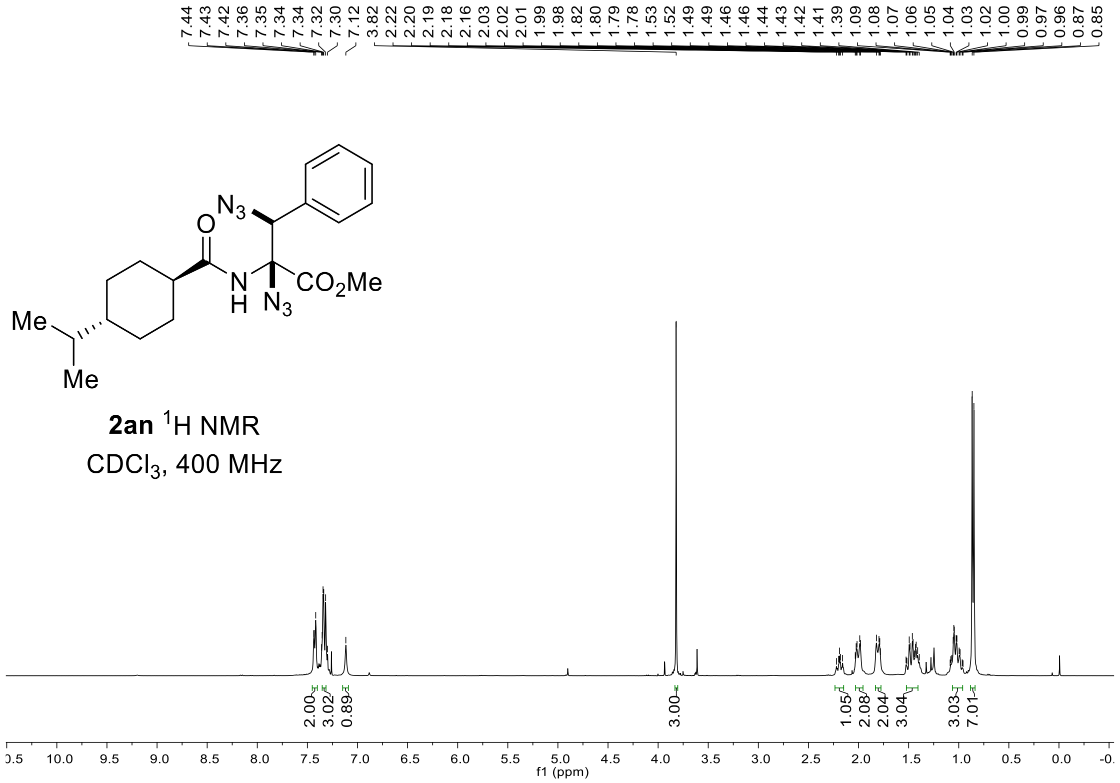
7.44 7.43 7.42 7.36 7.35 7.34 7.34 7.32 7.30 7.12 3.82 2.22 2.20 2.19 2.18 2.16 2.03 2.02 2.01 1.99 1.98 1.82 1.80 1.79 1.78 1.53 1.52 1.49 1.49 1.46 1.46 1.44 1.43 1.42 1.41 1.39 1.09 1.08 1.07 1.06 1.05 1.04 1.03 1.02 1.00 0.99 0.97 0.96 0.87 0.85
N_3
O
N
H
N_3
CO_2Me
Me
Me
2an ^{1}H NMR
$CDCl_3$, 400 MHz
2.00 3.02 0.89 3.00 1.05 2.08 2.04 3.04 3.03 7.01
10.0 9.5 9.0 8.5 8.0 7.5 7.0 6.5 6.0 5.5 5.0 4.5 4.0 3.5 3.0 2.5 2.0 1.5 1.0 0.5 0.0
f1 (ppm)

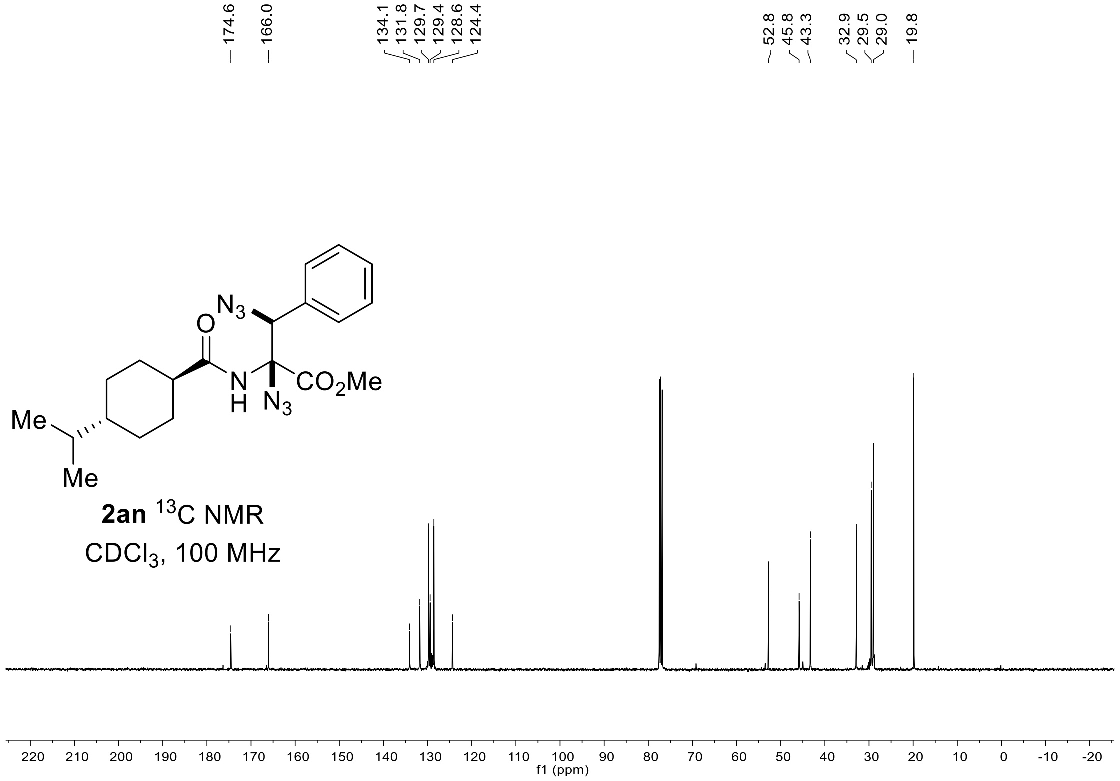
174.6 166.0 134.1 131.8 129.7 129.4 128.6 124.4 52.8 45.8 43.3 32.9 29.5 29.0 19.8
N_3
O
N
H
N_3
CO_2Me
Me
Me
2an ^{13}C NMR
$CDCl_3$, 100 MHz
220 210 200 190 180 170 160 150 140 130 120 110 100 90 80 70 60 50 40 30 20 10 0 -10 -20
f1 (ppm)

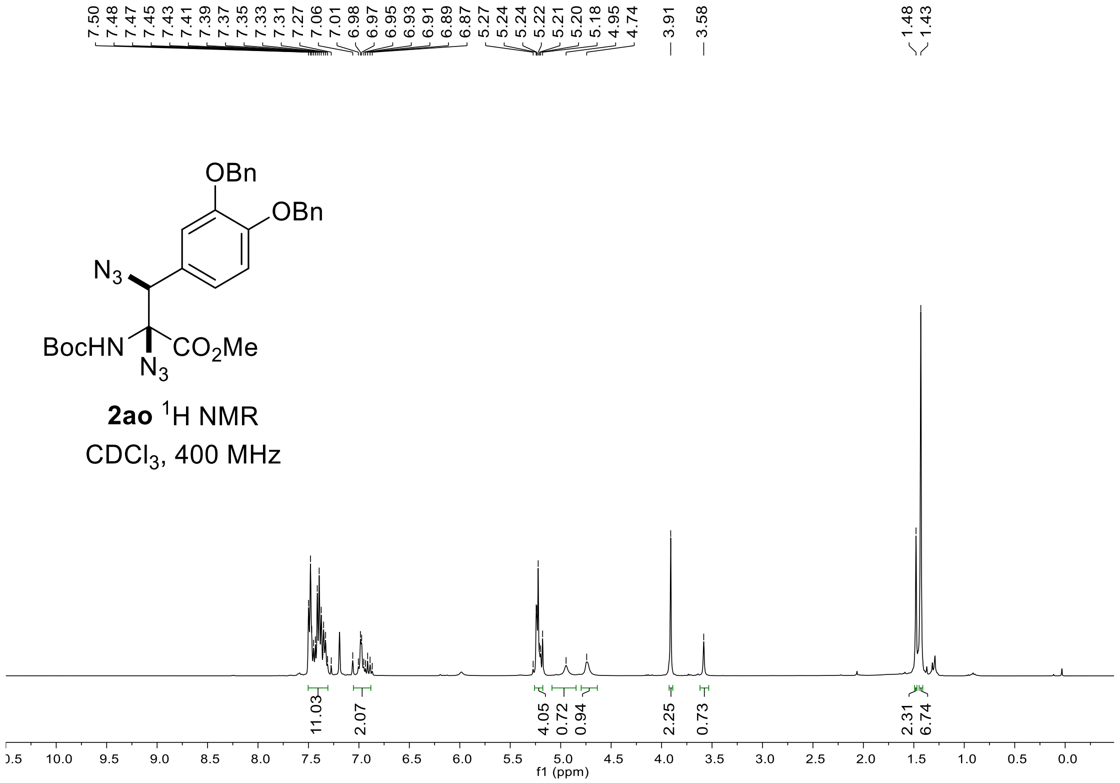
7.50 7.48 7.47 7.45 7.43 7.41 7.39 7.37 7.35 7.33 7.31 7.27 7.06 7.01 6.98 6.97 6.95 6.93 6.91 6.89 6.87 5.27 5.24 5.22 5.21 5.20 5.18 4.95 4.74
3.91
3.58
1.48 1.43
OBn
OBn
N3
BocHN
CO2Me
N3
2ao 1H NMR
CDCl3, 400 MHz
11.03
2.07
4.05
0.72
0.94
2.25
0.73
2.31
6.74
10.0 9.5 9.0 8.5 8.0 7.5 7.0 6.5 6.0 5.5 5.0 4.5 4.0 3.5 3.0 2.5 2.0 1.5 1.0 0.5 0.0
f1 (ppm)

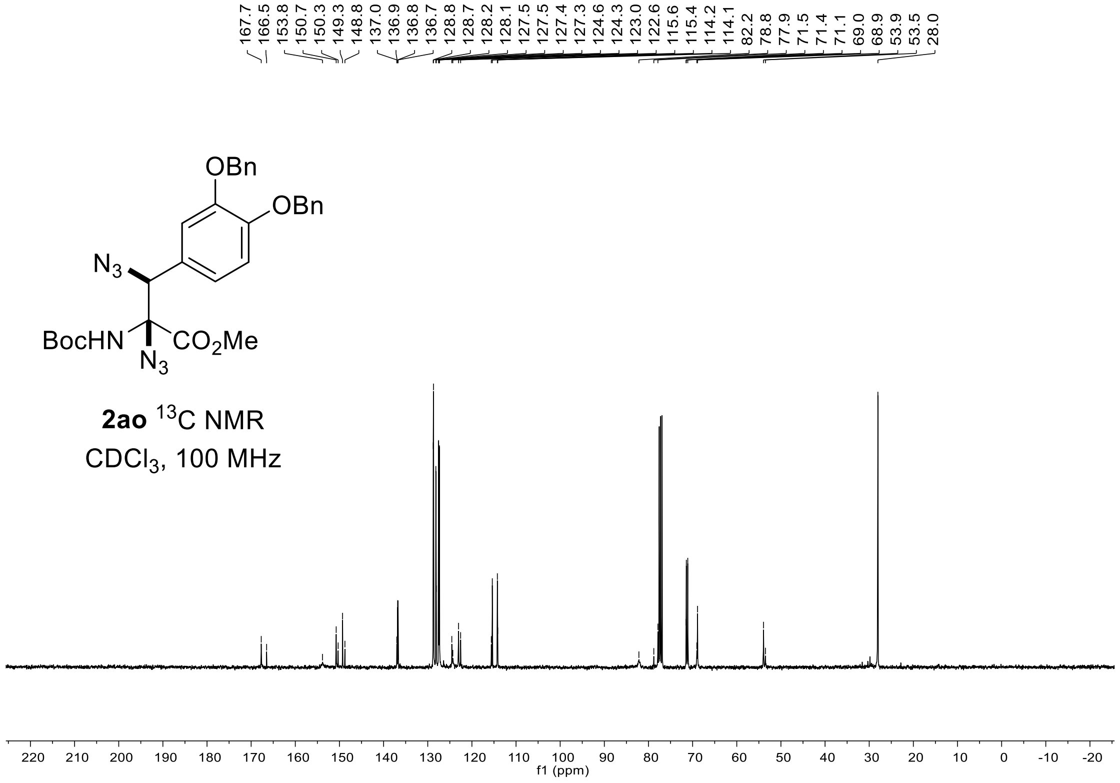
167.7 166.5 153.8 150.7 150.3 149.3 148.8 137.0 136.9 136.8 136.7 128.8 128.7 128.2 128.1 127.5 127.5 127.4 127.3 124.6 124.3 123.0 122.6 115.6 115.4 114.2 114.1 82.2 78.8 77.9 71.5 71.4 71.1 69.0 68.9 53.9 53.5 28.0
OBn
OBn
N3
BocHN
CO2Me
N3
2ao 13C NMR
CDCl3, 100 MHz
220 210 200 190 180 170 160 150 140 130 120 110 100 90 80 70 60 50 40 30 20 10 0 -10 -20
f1 (ppm)

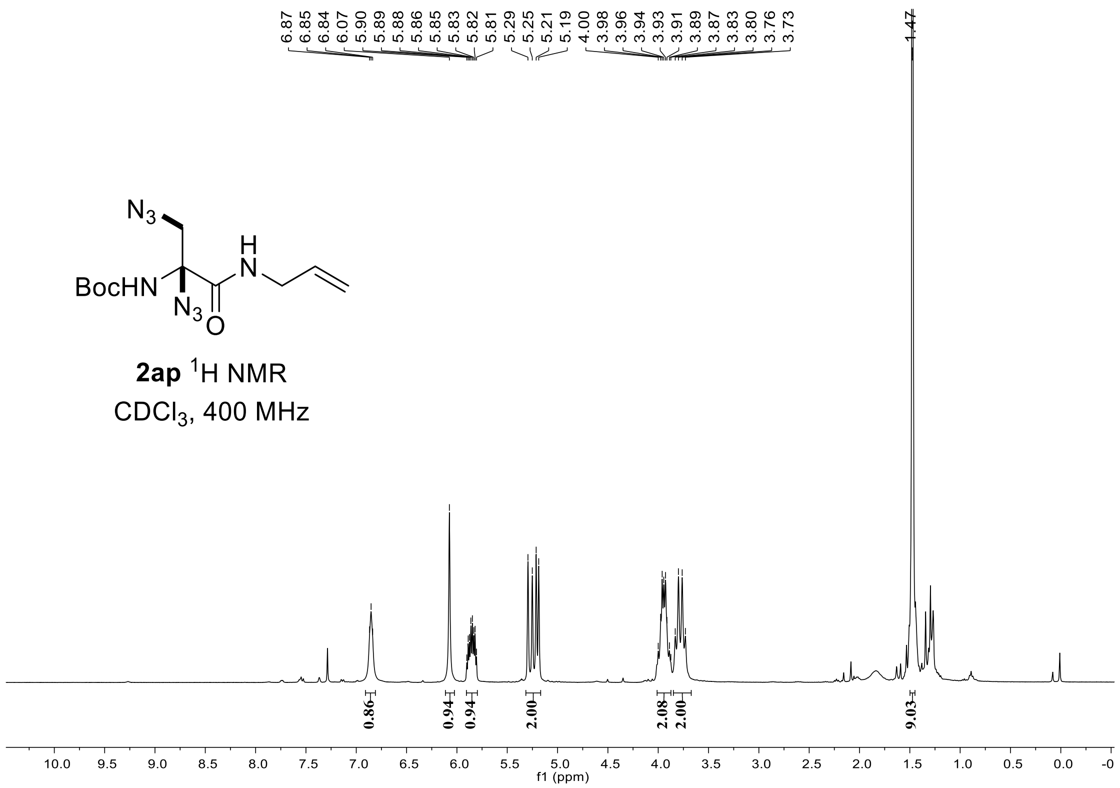
6.87
6.85
6.84
6.07
5.90
5.89
5.88
5.86
5.85
5.83
5.82
5.81
5.29
5.25
5.21
5.19
4.00
3.98
3.96
3.94
3.93
3.91
3.89
3.87
3.83
3.80
3.76
3.73
1.47
N3
H
N
BocHN
N3
O
2ap 1H NMR
CDCl3, 400 MHz
0.86
0.94
0.94
2.00
2.08
2.00
9.03
10.0 9.5 9.0 8.5 8.0 7.5 7.0 6.5 6.0 5.5 5.0 4.5 4.0 3.5 3.0 2.5 2.0 1.5 1.0 0.5 0.0 -0
f1 (ppm)

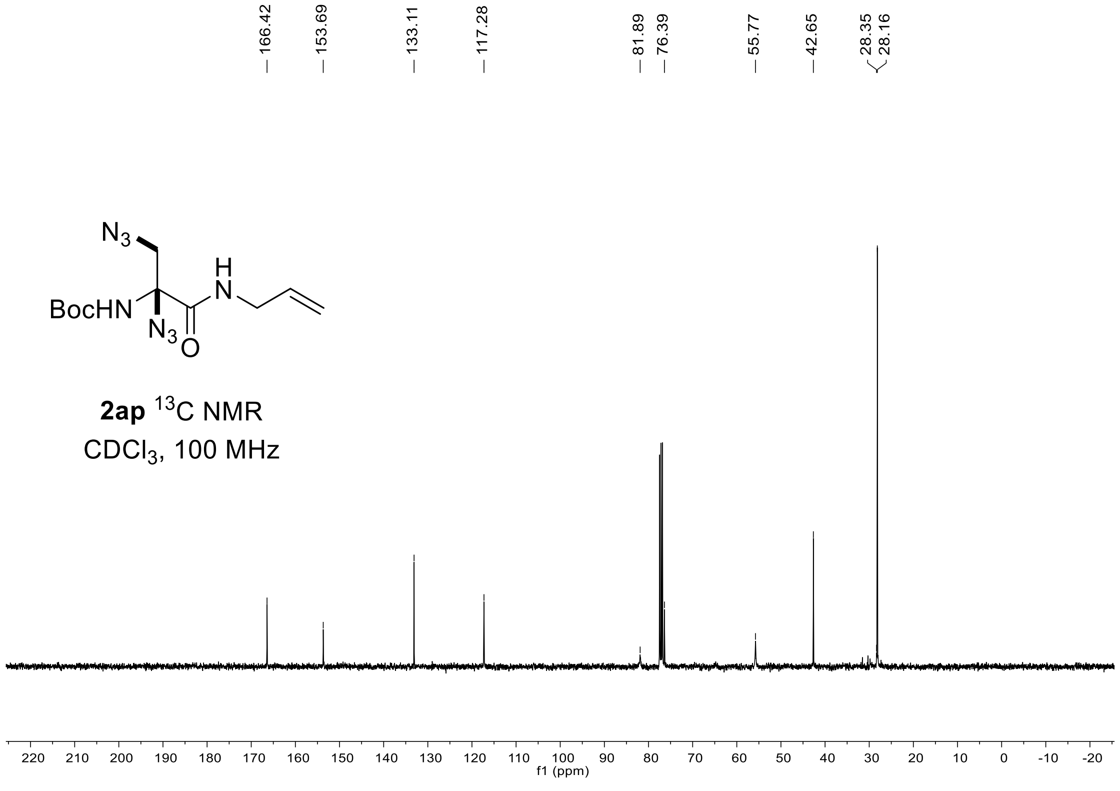
166.42
153.69
133.11
117.28
81.89
76.39
55.77
42.65
28.35
28.16
N3
H
N
BocHN
N3
O
2ap 13C NMR
CDCl3, 100 MHz
220 210 200 190 180 170 160 150 140 130 120 110 100 90 80 70 60 50 40 30 20 10 0 -10 -20
f1 (ppm)

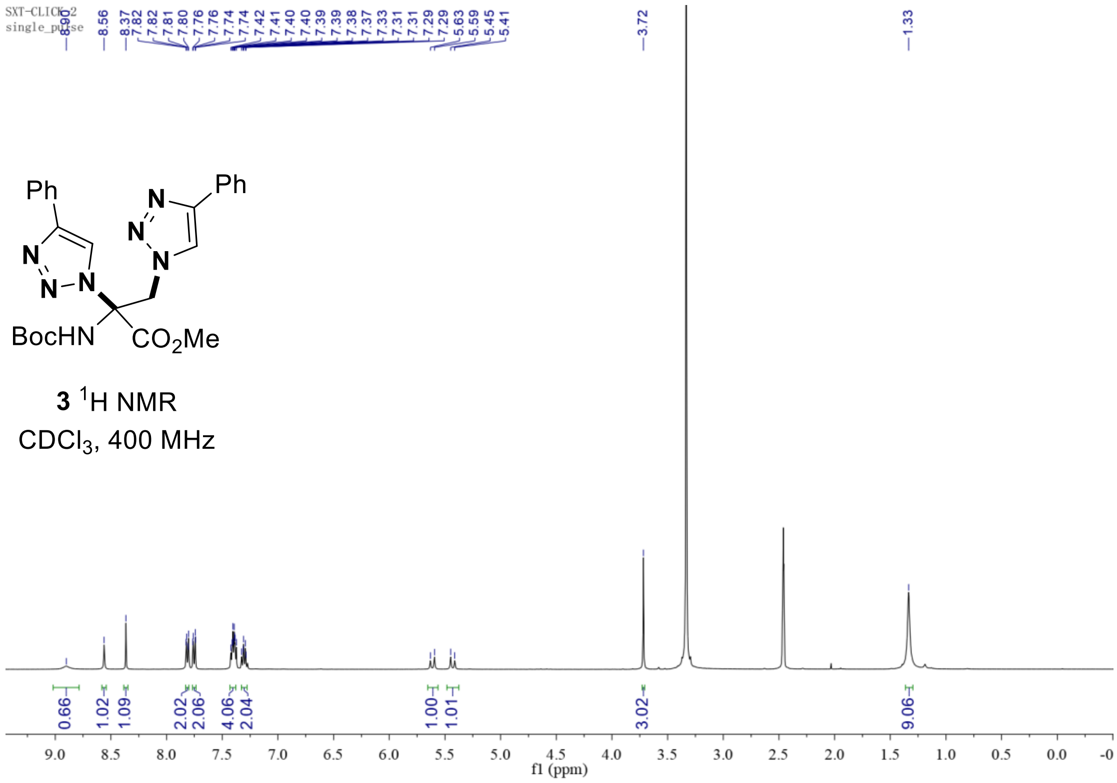

SXT-CLICK-2
single_pulse
8.90
8.56
8.37
7.82
7.81
7.80
7.76
7.76
7.74
7.74
7.42
7.41
7.40
7.40
7.39
7.39
7.38
7.37
7.33
7.31
7.31
7.29
7.29
5.63
5.59
5.45
5.41
3.72
1.33
Ph
N
N
N
Ph
N
N
N
BocHN
CO2Me
3 1H NMR
CDCl3, 400 MHz
0.66
1.02
1.09
2.02
2.06
4.06
2.04
1.00
1.01
3.02
9.06
9.0
8.5
8.0
7.5
7.0
6.5
6.0
5.5
5.0
4.5
4.0
3.5
3.0
2.5
2.0
1.5
1.0
0.5
0.0
-0
f1 (ppm)

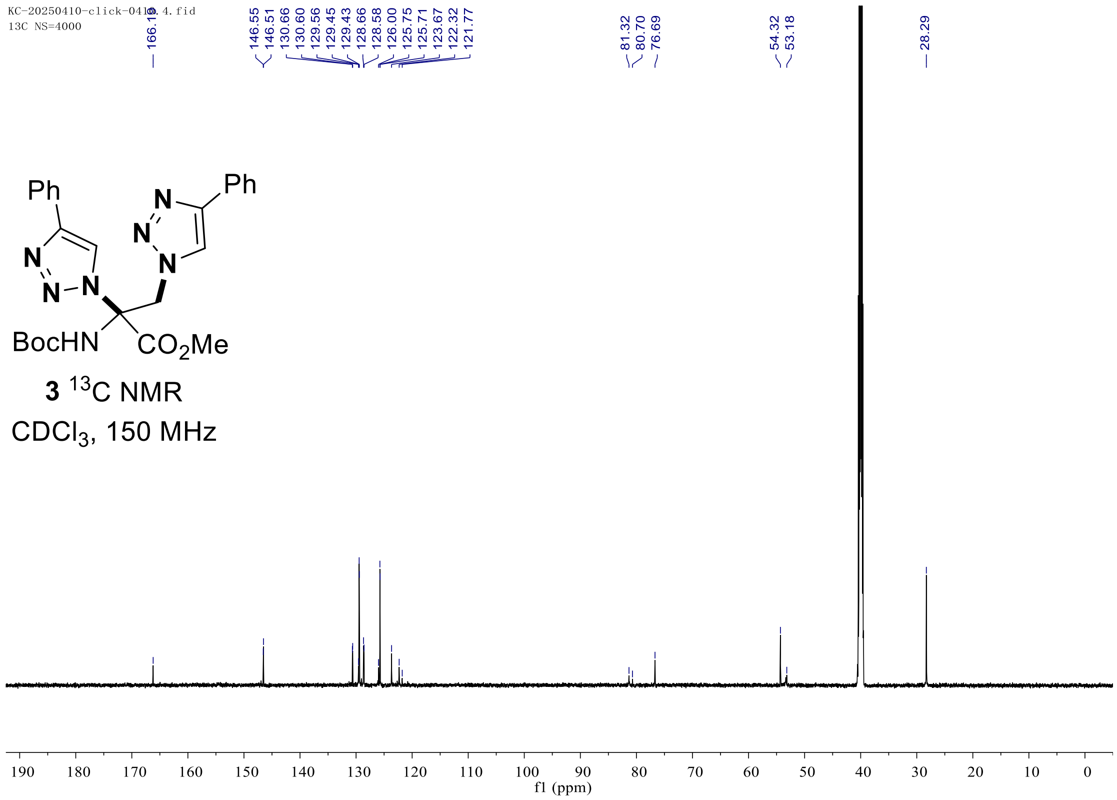

KC-20250410-click-0410.4.fid
13C NS=4000
166.19
146.55
146.51
130.66
130.60
129.56
129.45
129.43
128.66
128.58
126.00
125.75
125.71
123.67
122.32
121.77
81.32
80.70
76.69
54.32
53.18
28.29
Ph
N
N
N
Ph
N
N
N
BocHN
CO2Me
3 13C NMR
CDCl3, 150 MHz
190
180
170
160
150
140
130
120
110
100
90
80
70
60
50
40
30
20
10
0
f1 (ppm)
